# Efficacy and safety of incretin-based therapies in patients with type 2 diabetes mellitus: a network meta-analysis based on clinical trials

**DOI:** 10.3389/fphar.2026.1846714

**Published:** 2026-06-18

**Authors:** Xichao Wu, Yujiao Yang, Xueyan Cui, Xiao Li, Yan Li

**Affiliations:** 1 Shandong Medicine and Health Key Laboratory of Clinical Pharmacy, Shandong Engineering and Technology Research Center for Pediatric Drug Development, The First Affiliated Hospital of Shandong First Medical University / Shandong Qianfoshan Hospital, Jinan, China; 2 School of Pharmacy, Shandong University of Traditional Chinese Medicine, Jinan, Shandong, China

**Keywords:** adverse reactions, cardiorenal protection, efficacy, glucagon-like peptide-1 receptor agonists, network meta-analysis

## Abstract

**Objectives:**

This study systematically assessed the efficacy and safety of 15 incretin-based therapies (IBTs) for type 2 diabetes mellitus (T2DM), including mono- (GLP-1RA), dual- (GIP/GLP-1RA or GLP-1/GCGR), and triple- (GIP/GLP-1/GCGR) receptor agonists, and explored how dosage and treatment duration affect clinical outcomes to support individualized treatment decisions.

**Methods:**

A systematic review and Bayesian network meta-analysis were performed following PRISMA 2020 and PRISMA-NMA guidelines (PROSPERO: CRD420251152746). We searched MEDLINE, Cochrane Library, and Embase up to 1 August 2025, for randomized controlled trials (RCTs). Study selection, data extraction, risk-of-bias assessment (Cochrane RoB 2.0) and CINeMA framework were conducted. Analyses included SUCRA ranking, sensitivity, subgroup, and meta-regression in R Studio 4.4.3.

**Results:**

102 RCTs (98,693 T2DM patients) with low overall bias were included. IBTs were superior to placebo in glycemic control (tirzepatide, orforglipron, semaglutide best), weight loss (retatrutide best), lipid, blood pressure, cardiorenal, and insulin function improvement. The most common adverse events were mild, transient gastrointestinal reactions, and the risk of hypoglycemia was low. Higher doses improved efficacy but increased adverse events; 45 mg orforglipron balanced benefits and tolerability. Optimal duration and combination efficacy varied by agent.

**Conclusion:**

IBTs provide comprehensive benefits in T2DM. Efficacy and safety differ across agents, doses, durations, and combinations, supporting individualized therapy. Long-term high-quality RCTs are needed. Cardiovascular protection may stem from synergistic improvements in lipids, blood pressure, renal function, and insulin sensitivity.

**Systematic Review Registration:**

https://www.crd.york.ac.uk/prospero/, identifier CRD420251152746.

## 1 Introduction

Type 2 diabetes mellitus (T2DM) is a major global public health challenge, with its increasing prevalence placing substantial strain on healthcare systems and markedly impairing patients’ quality of life (Chen et al., 2025). Beyond glycemic dysregulation, T2DM is frequently associated with comorbidities such as obesity, dyslipidemia, hypertension (Singh et al., 2025), as well as elevated risks of target organ damage—including atherosclerotic cardiovascular disease (ASCVD) (Luciani et al., 2024), chronic kidney disease (CKD) (Thomas et al., 2016; Liu et al., 2024), and metabolic dysfunction-associated steatotic liver disease (MASLD) (Theodorakis and Nikolaou, 2025)--all of which complicate clinical management. Incretin-Based Therapies (IBTs), including mono- (glucagon-like peptide-1 receptor agonist, GLP-1RA), dual (glucose-dependent insulinotropic polypeptide/GLP-1 receptor agonist, GIP/GLP-1RA; or GLP-1/glucagon receptor agonist, GLP-1/GCGR), and triple (GIP/GLP-1/GCGR) receptor agonists, have emerged as cornerstone therapies in T2DM due to their pleiotropic benefits, including glycemic control, weight reduction, lipid regulation, and cardiorenal protection.

Recent clinical guidelines have updated T2DM treatment strategies to reflect the central role of IBTs. The Guidelines for the Prevention and Treatment of Diabetes Mellitus in China (2024 Edition) no longer list metformin as an absolute first-line therapy. Instead, they propose a risk-based hierarchical approach, recommending GLP-1RAs as first-line options for T2DM patients with established ASCVD (or high risk), CKD, overweight, obesity or MASLD—especially those with a history of ischemic stroke (National Health Commission of the People’s Republic of China, 2025). Similarly, the 2025 American Diabetes Association (ADA) Standards of Medical Care in Diabetes emphasize patient-centered, individualized treatment strategies, prioritizing GLP-1RAs or GIP/ GLP-1 dual agonists for T2DM patients with MASLD, overweight, obesity or metabolic dysfunction-associated steatohepatitis (MASH). Furthermore, GLP-1RAs are strongly recommended for individuals with CKD and eGFR < 30 mL/min/1.73 m^2^ due to their favorable safety profile and proven cardiovascular benefits (American Diabetes Association Professional Practice Committee, 2025).

IBTs vary in molecular structure, medication regimens, and administration cycles. With the continuous development of these drugs, there remains a lack of comprehensive and unified evaluations regarding their comparative efficacy, safety profiles, and applicable populations across different clinical scenarios. Most previous studies have only analyzed a single class of drugs, routine indicators, or individual outcome measures. For instance, a network meta-analysis conducted by Wu et al., which included 360 randomized controlled trials (RCTs) involving a total of 157,696 patients, confirmed that incretin drugs can significantly improve HOMA-βand HOMA-IR (Wu et al., 2019). Stefanou et al. performed a systematic review and meta-analysis of 16 RCTs with 28,168 patients, verifying that GLP-1 RAs and dual GIP/GLP-1 RAs can reduce the incidence of MACE and all-cause mortality (Stefanou et al., 2024). A meta-analysis by Mendonça et al., based on 8 RCTs enrolling 68,572 patients, found that GLP-1 RAs could lower the risk of renal function deterioration by 16% (Mendonça et al., 2025). Furthermore, in clinical practice and previous trials, incretin drugs are frequently combined with other oral antidiabetic drugs (OADs) or insulin. However, prior meta-analyses failed to thoroughly explore the impacts of background medications and active control groups. Waldrop et al. integrated trial data from 2008 onwards, stratified the results according to control regimens such as placebo and monotherapy, and comprehensively assessed the hypoglycemic and weight-loss effects of the drugs. They also analyzed the influences of baseline combined use of OADs and the intragroup efficacy differences within the same drug classes on glycemic control (Waldrop et al., 2018). In contrast, the present study included 15 types of incretin drugs. It comprehensively analyzed pancreatic function indicators (HOMA-β, HOMA-IR, and fasting C-peptide) as well as cardiac and renal function indicators (MACE, eGFR, and UACR). The study also updated data after 2016 and conducted stratified analyses on treatment regimens and control regimens in the subgroup analysis, addressing the lack of comprehensive multi-dimensional and multi-target evaluation in existing research.

As a robust tool for synthesizing evidence from multiple trials, network meta-analysis enables both direct and indirect comparisons of interventions, facilitating comprehensive efficacy-safety ranking. In accordance with the PRISMA 2020 and PRISMA-NMA guidelines, this study constructed a systematic literature search and rigorous screening of RCTs evaluating 15 IBTs for T2DM. It comprehensively evaluated their performance in key domains including glycemic control, weight management, lipid and blood pressure regulation, cardiorenal protection, and insulin function improvement, while also exploring the impacts of dose, treatment duration and combination therapy on outcomes. Based on receptor targeting profiles, the 15 agents were categorized into three main groups: GLP-1RAs (semaglutide, liraglutide, dulaglutide, exenatide, lixisenatide, orforglipron, polyethylene glycol loxenatide, efpeglenatide, ITCA 650); dual receptor agonists, including the GIP/GLP-1R agonist (tirzepatide) and the GLP-1R/GCGR agonist (mazdutide, survodutide); and the triple GIP/GLP-1R/GCGR agonist (retatrutide). The primary objective is to provide high-quality, actionable evidence to inform guideline-concordant clinical decision-making and optimize the benefit-risk balance in the management of T2DM.

## 2 Methods

The protocol of this systematic review and network meta-analysis was registered with PROSPERO (registration number: CRD420251152746). This study was conducted in accordance with the Preferred Reporting Items for Systematic Reviews and Meta-Analyses (PRISMA) 2020 edition (Page et al., 2021) and the PRISMA Extension Statement for Network Meta-Analyses (PRISMA-NMA) (Hutton et al., 2015).

### 2.1 Search strategy

A comprehensive literature search was conducted in Medline, the Cochrane Library, Embase, and selected Chinese databases to identify RCTs evaluating IBTs in T2DM patients, from database inception to 1 August 2025. An additional supplementary search focusing on RCTs involving cardiorenal outcomes and insulin function parameters was performed on 11 September 2025, to minimize the risk of missing relevant studies. Three reviewers independently conducted the search and screening processes, with discrepancies resolved through consultation with a fourth reviewer. Furthermore, manual searches were carried out on the official websites of the European Association for the Study of Diabetes (EASD) and the ADA scientific meetings, as well as on ClinicalTrials.gov. The reference list of included studies and previously published systematic reviews were also screened to identify potentially eligible trials.

The search terms of this study are as follows. (“tirzepatide” OR “orforglipron” OR “semaglutide” OR “retatrutide” OR “liraglutide” OR “dulaglutide” OR “exenatide” OR “lixisenatide” OR “mazdutide” OR “survodutide” OR “visepegenatide” OR “polyethylene glycol loxenatide” OR “efpeglenatide” OR “ITCA 650″) AND (“type 2 diabetes” OR “type 2 diabetes mellitus” OR “T2DM”) AND (“randomized controlled trial” OR “RCT” OR “randomized” OR “placebo-controlled”). Complete search strategies are provided in Supplementary Appendix 1.

### 2.2 Eligibility criteria

Studies that enrolled adult participants with T2DM and its associated complications were considered eligible if they involved treatment with IBTs and included a minimum follow-up duration of 12 weeks. Interventions consisted of either IBTs monotherapy or combination therapy with non-randomized background antihyperglycemic agents. Control groups received placebo, a different IBTs, or other antidiabetic drugs. Only peer-reviewed, published RCTs were included; studies presented solely as conference abstracts, crossover designs, and trials involving discontinued or withdrawn drugs were excluded.

### 2.3 Screening process

The retrieved database records were imported into EndNote X9 to remove duplicates and were then merged with results from other sources for comprehensive comparison. The screening process consisted of three stages. Firstly, three reviewers independently screened articles based on titles, retaining any studies with uncertain eligibility for further assessment. Secondly, all articles retained in the initial stage underwent abstract review, during which discrepancies among reviewers were resolved through consensus discussions, with arbitration by a fourth reviewer when necessary. Thirdly, full-text version of articles that met the preliminary inclusion criteria based on titles and abstracts were assessed against predefined eligibility criteria to determine final inclusion.

The criteria for full-text review were as follows: the outcome indicators of the article met the study’s requirements, there was no substantial missing data, double-blind design RCTs were preferred, and open-label trials were eligible for inclusion if their outcome indicators fulfilled the study’s objectives.

### 2.4 Data extraction

For each eligible study, data were independently extracted by two reviewers using a standardized, pre-designed form. The extracted information included study characteristics (e.g., publication year, treatment duration), study population demographics (e.g., age, gender, sample size, diabetes duration, body mass index), intervention details (e.g., agent name and dosage), and outcome indicators. The following efficacy outcomes were collected: mean change from baseline in glycated hemoglobin (HbA_1c_), change in fasting plasma glucose (FPG) concentration from baseline, proportion of patients achieving glycemic targets of HbA_1c_ <7.0% and <6.5%, change in body weight from baseline, as well as changes in blood pressure systolic blood pressure (SBP) and diastolic blood pressure (DBP)) and serum lipid profiles (such as high-density lipoprotein (HDL), low-density lipoprotein (LDL), total cholesterol (TC), and triglyceride (TG) levels). In addition, key clinical endpoints were extracted to assess cardiorenal effects and insulin function, including all-cause mortality (ACM), non-fatal stroke (NFS), non-fatal myocardial infarction (NFM), cardiovascular mortality (CVM), major adverse cardiovascular events (MACE), estimated glomerular filtration rate (eGFR), urinary albumin/creatinine ratio (UACR), fasting insulin level (FIL), homeostasis model assessment of β-cell function (HOMA-β), homeostasis model assessment of insulin resistance (HOMA-IR), and C-peptide levels during treatment. Safety data were comprehensively evaluated by extracting all reported adverse events without predefined restrictions. For studies presenting data graphically, numerical values were estimated using OriginPro software. All extractions were performed by two independent reviewers and subsequently verified, with discrepancies resolved through discussion or adjudication by a third reviewer. Baseline characteristics of the included RCTs are summarized in Supplementary Appendix 3.

### 2.5 Quality assessment of evidence

The Cochrane Risk of Bias Tool for Randomized Trials (RoB 2.0) was used to assess the risk of bias in all included trials across the following domains: random sequence generation, allocation concealment, blinding, incomplete outcome data, and selective reporting of results (Sterne et al., 2019). If the risk of bias was judged to be low in all domains, the trial was assigned an overall low risk of bias (score 1). If the risk of bias was high in at least one domain, the trial was classified as having a high overall risk of bias (score 3). In all other cases--such as when some concerns were identified in one or more domains--the overall risk of bias was rated as having some concerns (score 2). Risk of bias assessments were conducted independently by two reviewers, with disagreements resolved by consensus discussion. To quantitatively assess funnel plot symmetry, Egger’s regression test and Begg’s rank correlation test were performed (Sterne et al., 2001). Funnel plots were generated using estimates from direct evidence to visually inspect small-study effects, with each comparison evaluated separately. We further applied the Confidence in Network Meta-Analysis (CINeMA) framework to comprehensively assess the certainty of evidence generated by network meta-analysis, covering six core key domains: within-study bias, reporting bias, indirectness, imprecision, heterogeneity, and inconsistency. To evaluate intransitivity (a key component of indirectness assessment in the CINeMA framework), we systematically compared potential effect modifiers, including baseline age, baseline HbA_1c_ concentration, diabetes duration, and body mass index (BMI), between studies that provided direct and indirect evidence for each pair of treatment comparisons. In accordance with standardized CINeMA assessment criteria, each domain of the CINeMA assessment was rated on a hierarchical scale (very low, low, moderate, high), and the overall certainty of evidence for each primary and secondary outcome measure was synthesized based on the ratings of the individual domains. The CINeMA assessment was conducted by two independent reviewers, with any discrepancies resolved through discussion and, if necessary, adjudicated by a third senior reviewer.

### 2.6 Data synthesis and analysis

We employed an evidence-based evaluation framework integrating Bayesian network meta-analysis and frequentist methods, using R software (version 4.4.3) as the analytical platform. A comprehensive analytical system was constructed using the GeMTC, R JAGS, and netmeta packages. Within the Bayesian framework, non-informative priors, a consistent random-effects model, and a normal likelihood-identity link function were set (Noma, 2023). Parameter estimation was performed via the Markov Chain Monte Carlo (MCMC) algorithm, and model convergence was assessed using the Potential Scale Reduction Factor (PSRF), along with trace plots and density plots (Liu et al., 2023). Mean differences (MD) were used for continuous outcomes description; for binary outcomes, odds ratios (OR) were used to quantify effect sizes. Relative effects of interventions were presented through league tables and forest plots, while efficacy ranking was conducted using rank probability analysis and Surface Under the Cumulative Ranking (SUCRA) values. Network consistency was evaluated using the node-splitting method (Van Valkenhoef et al., 2016). Heterogeneity was assessed using the Bayesian I^2^ statistic--the proportion of between-study variance to total variance (Higgins et al., 2003). Missing data were supplemented by searching clinical trial registries or referring to comparable studies. Additionally, network meta-regression analyses were conducted to incorporate baseline characteristics (e.g., disease duration, age, glycosylated hemoglobin, etc.) and intervention-related factors (e.g., dosage, follow-up duration, concomitant treatment regimens) to explore potential effect modifiers, and subgroup analyses were performed according to the above intervention factors. Under the frequentist framework, the netmeta package was utilized to assess publication bias through Egger’s test and corrected funnel plots.

## 3 Results

### 3.1 Literature selection and study characteristics

**FIGURE 1:**
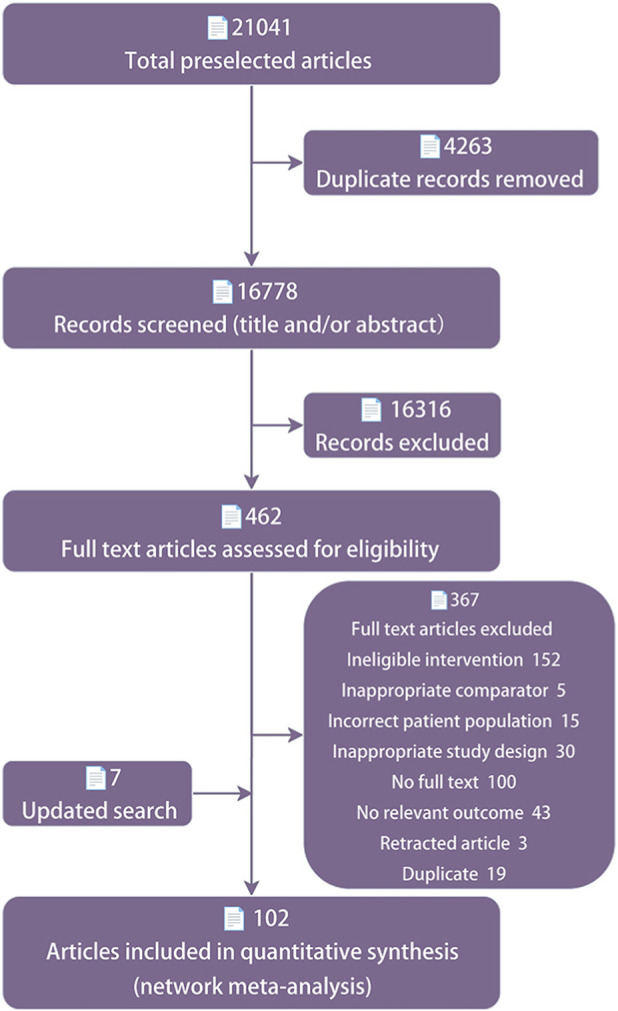
PRISMA flow diagram showing study identification, inclusion, and exclusion.

A total of 21,041 citations were identified through the initial literature search, of which 462 full-text articles were assessed for eligibility. The literature search was updated to ensure inclusion of the most current evidence. Based on the predefined inclusion criteria, 102 RCTs, involving 98,693 adult participants, were included in the analyses (Figure 1). The sample size of individual studies ranged from 43 to 14,752 participants. Intervention duration varied from 12 to 280 weeks. The mean age across studies was 57.13 years (standard deviation [SD] 9.86), with a mean proportion of male participants at 45.29%. The mean duration of diabetes was 8.57 years (SD 6.52). At baseline, the mean body mass index was 31.30 kg/m^2^ (SD 6.47), and mean HbA_1c_ level was 8.70% (SD 0.98) (Supplementary Appendix 2). No evidence of asymmetry in the funnel plot was observed, suggesting low risk of publication bias (Supplementary Appendix 9).

### 3.2 Risk of bias, certainty of evidence, and consistency

The risk of bias for each included trial is presented in Supplementary Appendix 4. A key limitation identified was incomplete reporting of blinding procedures for participants, researchers, and outcome assessors. Among the 102 trials, all were assessed as having a low risk of bias in random sequence generation, 101 (99.01%) in deviation from intended interventions, 101 (99.01%) in missing outcome data, and 92 (90.20%) in outcome measurement. All 102 studies (100%) exhibited a low risk of selective reporting bias. Overall, only one study (0.98%) was judged to have a high overall risk of bias.

Although some comparisons showed potential inconsistency, for most outcomes there was no statistically significant evidence of network inconsistency. Given that this review strictly included RCTs according to predetermined eligibility criteria, inter−study heterogeneity was generally low. Heterogeneity test using the I^2^ statistic revealed no substantial heterogeneity across the network, with most comparisons showing low or low−to−moderate levels of heterogeneity (Supplementary Appendix 5).

The results of the evidence certainty assessment based on the CINeMA framework show that after assessing the level of evidence using CINeMA, most of the results of the pairwise comparisons were of moderate or high confidence (Supplementary Appendix 10). All networks met the principle of transitivity, endowing the validity of indirect comparisons (Supplementary Appendix 10, Supplementary Table S10.1).

### 3.3 Sensitivity analyses and meta−regressions

To further evaluate the robustness of the results, sensitivity analyses were conducted by sequentially excluding all previously included trials comparing IBTs with other classes of antihyperglycemic−agents −− retaining only RCTs with placebo as control −− along with additional assessments stratified by dosage, exclusion of open−label trials, and removal of RCTs employing combination therapy designs. As illustrated in Supplementary Appendix 10, the results of sensitivity analysis were consistent with the primary outcomes, thereby supporting the stability and reliability of the study findings.

Meta−regression analysis was performed to assess the potential impact of baseline factors on the main outcomes, including age, proportion of male participants, duration of diabetes, BMI, baseline HbA_1c_, and combination therapy. The analysis revealed that combination therapy was significantly associated with greater treatment effects on the primary outcome measure (P < 0.05; see Supplementary Appendix 11).

### 3.4 Glycemic control

**FIGURE 2:**
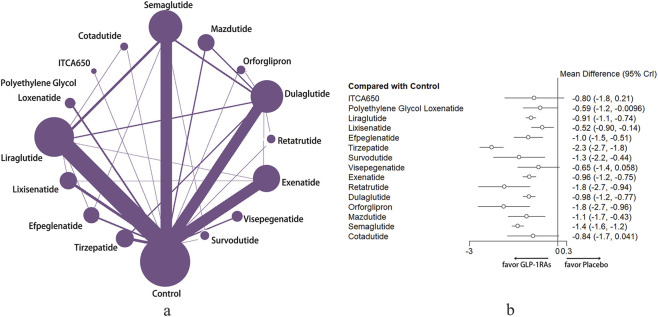
Network meta−analysis of IBTs versus placebo for HbA_1c_. **(a)** Network of available comparisons. The size of each node is proportional to the number of trial participants, and the thickness of the lines connecting nodes is proportional to the number of randomized participants directly comparing the two treatments. **(b)** Forest plot of network effect sizes. Effect sizes (with 95% confidence intervals) for each IBTs compared with placebo are shown.

The network meta−analysis on HbA_1c_ reduction included 102 trials involving 98,693 participants. Compared with placebo, all 15 IBTs demonstrated statistically significant efficacy in reducing HbA_1c_ levels in adult T2DM patients (Figure 2). Tirzepatide exhibited the greatest effect size, with a MD of −2.30% ([95% CrI −2.70 to −1.80], SUCRA 99.72%), followed by orforglipron (MD −1.80% [95% CrI −2.70 to −0.96], SUCRA 91.71%). Full ranking results based on SUCRA values are shown in Supplementary Appendix 7, Supplementary Table S7.1.

**FIGURE 3:**
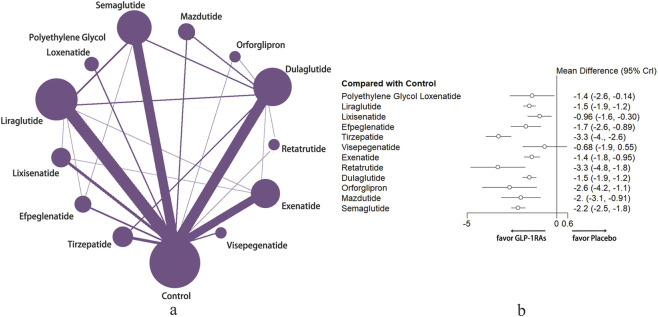
Network meta−analysis of IBTs versus placebo for FPG. **(a)** Network of available comparisons. The size of each node is proportional to the number of trial participants, and the thickness of the lines connecting nodes is proportional to the number of randomized participants directly comparing the two treatments. **(b)** Forest plot of network effect sizes. Effect sizes (with 95% confidence intervals) for each IBTs compared with placebo are shown.

As assessed by FPG, the network meta−analysis included 87 RCTs involving 45,510 participants, confirming the placebo−controlled efficacy of all 12 IBTs (Figure 3). Tirzepatide showed the greatest reduction, with a MD of −3.30 mmol/L ([95% CrI −4.00 to −2.60], SUCRA 98.73%), followed by orforglipron (MD −2.60 mmol/L [95% CrI −4.20 to −1.10], SUCRA 87.54%). Complete ranking results based on SUCRA are presented in Supplementary Appendix 1, Supplementary Table S7.2.

For the proportion of T2DM patients achieving the HbA_1c_ target of 7.0%, the network meta−analysis included 73 RCTs involving 41,660 participants, demonstrating the statistically significant efficacy of all 12 IBTs compared with placebo (Supplementary Appendix 6, Supplementary Table 6.1). Orforglipron showed the greatest effect, with a MD of 59.00% ([95% CrI 49.00 to 69.00], SUCRA 96.03%), followed by tirzepatide (MD 58.00% [95% CrI 52.00 to 65.00], SUCRA 95.64%). (The SUCRA ranking is shown in the Supplementary Appendix 7, Supplementary Table 7.4).

Regarding the proportion of T2DM patients achieving the more stringent HbA_1c_ target of 6.5%, the analysis included 67 RCTs involving 37,559 participants, confirming the placebo−controlled efficacy of all 12 IBTs (Supplementary Appendix 6, Supplementary Table 6.2). Tirzepatide ranked first (MD 60.00% [95% CrI 53.00 to 66.00], SUCRA 96.41%), followed by orforglipron (MD 59.00% [95% CrI 49.00 to 70.00], SUCRA 95.80%). Full SUCRA−based rankings are presented in Supplementary Appendix 7, Supplementary Table 7.5.

### 3.5 Weight loss

**FIGURE 4:**
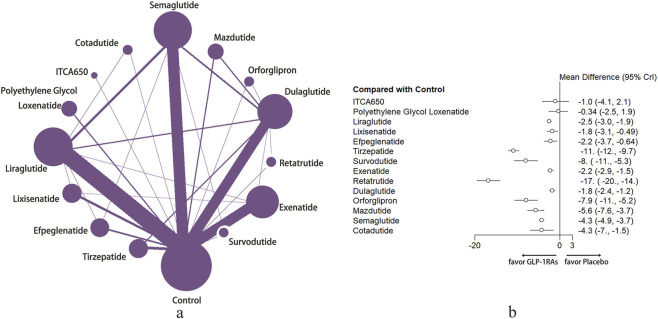
Network meta−analysis of IBTs versus placebo for Weight loss. **(a)** Network of available comparisons. The size of each node is proportional to the number of trial participants, and the thickness of the lines connecting nodes is proportional to the number of randomized participants directly comparing the two treatments. **(b)** Forest plot of network effect sizes. Effect sizes (with 95% confidence intervals) for each IBTs compared with placebo are shown.

This analysis included 98 trials involving 54,156 participants for body weight change. Retatrutide demonstrated the greatest efficacy in weight reduction, with a MD of −17.00 kg ([95% CrI −20.00 to −14.00], SUCRA 99.81%), and was ranked as the most effective IBTs (Figure 4). Compared with placebo, tirzepatide, orforglipron, mazdutide, semaglutide, and survodutide also showed significant reductions in body weight. Complete ranking results based on SUCRA values are provided in Supplementary Appendix 7, Supplementary Table S7.3.

### 3.6 Lipid profiles

For HDL elevation, liraglutide demonstrated the greatest effect, with a MD of 0.03 mmol/L ([95% CrI −0.003 to 0.06], SUCRA 90.96%). For LDL−C reduction, visepegenatide showed the largest decrease (MD −0.31 mmol/L [95% CrI −0.78 to 0.16], SUCRA 85.93%). For TC reduction, exenatide was the most effective (MD −0.24 mmol/L [95% CrI −0.33 to −0.16], SUCRA 93.33%). For TG reduction, visepegenatide again ranked highest (MD −0.31 mmol/L [95% CrI −0.78 to 0.16], SUCRA 84.07%. Detailed results are summarized in Supplementary Appendix 6, Supplementary Tables S6.2–S6.6. Complete SUCRA−based rankings are presented in Supplementary Appendix 7, Supplementary Tables S7.6–S7.9.

### 3.7 Blood pressure

For SBP reduction, eight IBTs demonstrated statistically significant efficacy. Orforglipron showed the greatest effect, with a MD of −8.50 mmHg ([95% CrI −10.00 to −6.90], SUCRA 99.99%), followed by liraglutide (MD −3.50 mmHg [95% CrI −4.50 to −2.40], SUCRA 77.66%). For DBP reduction, visepegenatide was the most effective (MD −2.10 mmHg [95% CrI −5.20 to 0.98], SUCRA 79.08%). Detailed results are provided in Supplementary Appendix 6, Supplementary Tables S6.7, S6.8. Complete SUCRA−based rankings are shown in Supplementary Appendix 7, Supplementary Tables S7.10, S7.11.

### 3.8 MACE

Tirzepatide demonstrated the most favorable overall profile, showing the greatest efficacy across four outcomes: MACE (OR 0.57 [95% CrI 0.39 to 0.79], SUCRA 96.21%), non−fatal myocardial infarction (OR 0.62 [95% CrI 0.33 to 1.1], SUCRA 88.39%), cardiovascular death (OR 0.64 [95% CrI 0.10 to 3.7], SUCRA 67.16%), and all−cause mortality (OR 0.58 [95% CrI 0.08 to 3.8], SUCRA 73.65%). It also ranked highly for non−fatal stroke (OR 0.69 [95% CrI 0.28 to 1.60], SUCRA 69.62%). Subgroup analyses indicated that ITCA650 did not demonstrate statistically significant benefits for MACE, cardiovascular death, and all−cause mortality. Similarly, lixisenatide did not show significant efficacy in reducing non−fatal stroke and non−fatal myocardial infarction. Detailed results are presented in Supplementary Appendix 6, Supplementary Tables S6.9–S6.13. Complete SUCRA−based rankings are provided in Supplementary Appendix 7, Supplementary Tables 7.12–S7.16.

### 3.9 Renal function

Regarding the changes in eGFR, polyethylene glycol loxenatide showed the greatest improvement compared with the placebo (MD 2.30 mL/min/1.73 m^2^ [95% CrI −4.50 to 9.20]). For UACR reduction, exenatide demonstrated the most favorable effect (MD −17.00 mg/g [95% CrI −43.00 to −8.20]). Detailed results are provided in Supplementary Appendix 6, Supplementary Tables S6.14, S6.15.

### 3.10 Function of insulin

For the outcome of reduced FIL levels, tirzepatide showed the greatest efficacy (MD −23.00 pmol/L [95% CrI −32.00 to −14.00], SUCRA 98.55%). For reduced C−peptide levels, tirzepatide again the most effective (MD −0.20 ng/mL [95% CrI −0.36 to −0.05], SUCRA 97.33%). For improvement in insulin resistance as measured by HOMA−IR, liraglutide ranked highest (MD −0.58 [95% CrI −0.90 to −0.25], SUCRA 82.94%). For enhancement of beta−cell function assessed by HOMA−β, tirzepatide demonstrated optimal performance (MD 31.00 [95% CrI 21.00 to 41.00], SUCRA 86.86%). Detailed results are summarized in Supplementary Appendix 6, Supplementary Tables S6.16–S6.19. Complete SUCRA−based rankings are provided in Supplementary Appendix 7, Supplementary Table S7.1.

### 3.11 Adverse events

All evaluated IBTs were associated with a statistically significant increase in the risk of gastrointestinal adverse events compared with placebo. For nausea incidence, ITCA650 had the highest risk (OR 6.70 [95% CrI 2.50 to 18.00]), followed by visepegenatide (OR 5.30 [95% CrI 2.30 to 12.00]), polyethylene glycol loxenatide (OR 4.90 [95% CrI 1.60 to 17.00]) and liraglutide (OR 3.60 [95% CrI 2.80 to 4.80]). In terms of the incidence of vomiting, ITCA650 had the highest risk (OR 12.00 [95% CrI 5.50 to 27.00]), followed by visepegenatide (OR 4.20 [95% CrI 2.00 to 9.60]), polyethylene glycol loxenatide (OR 3.60 [95% CrI 1.30 to 11.00]). In terms of the incidence of diarrhea, mazdutide had the highest risk (OR 4.70 [95% CrI 2.90 to 7.80]), and the risks of tirzepatide (OR 2.30 [95% CrI 1.80 to 2.90]) and retatrutide (OR 2.40 [95% CrI 1.30 to 4.20]) were similar. In terms of the incidence of constipation, retatrutide had the highest risk (OR 4.40 [95% CrI 1.90 to 9.90]), followed by tirzepatide (OR 2.60 [95% CrI 1.50 to 4.50]) (Supplementary Appendix 6, Supplementary Tables S6.20–S6.23).

### 3.12 Subgroup analyses of each GLP−1RA by doses and administration regimen

All IBTs at varying dosages significantly reduced HbA_1_c, FBG, and body weight: retatrutide 12 mg achieves the greatest FBG reduction (MD −3.20 mmol/L [95% CrI −4.30 to −2.10]) and weight loss (MD −15.00 kg [95% CrI −17.00 to −13.00]), while tirzepatide 15 mg induces the most notable HbA_1_c decrease (MD −2.00% [95% CrI to 2.20 to −1.80]). Most IBTs are linked to increased gastrointestinal adverse events: efpeglenatide 12 mg (MD 44.00% [95% CrI 28.00 to 61.00]) and orforglipron 24 mg (MD 28.00% [95% CrI: 18.00 to 39.00]) carry the highest nausea risk; efpeglenatide 14 mg (MD 28.00% [95% CrI 12.00 to 45.00]) and survodutide 1.8 mg (MD 19.00% [95% CrI 8.60 to 29.00]) present notable vomiting risk; mazdutide 4.5 mg (MD 32.00% [95% CrI 24.00 to 40.00]) and 3 mg (MD 28.00% [95% CrI 20.00 to 36.00]) pose higher diarrhea risk; orforglipron 24 mg (MD 13.00% [95% CrI 7.20 to 20.00]) and 12 mg (MD 11.00% [95% CrI 4.50 to 17.00]) have elevated constipation risk (see Supplementary Appendix 12).

### 3.13 Subgroup analyses of individual GLP−1RA by treatment duration

All IBTs achieved significant HbA_1_c reduction across all durations, with tirzepatide exhibiting optimal long−term efficacy (MD −1.80% [95% CrI −2.10 to −1.50]). Most IBTs also yielded significant FPG reduction, with dulaglutide demonstrating the best mid−term efficacy (MD −4.40 mmol/L [95% CrI −7.70 to −1.10]) most IBTs induced significant weight loss, with tirzepatide exerting the strongest long−term effect (MD −9.90 kg [95% CrI −12.00 to −7.90]); regarding safety, most IBTs elevated the risks of nausea, vomiting, diarrhea and constipation across durations, with semaglutide having the highest long−term nausea risk (OR 11.00 [95% CrI 6.20 to 22.00]) and mid−term vomiting risk (OR 8.30 [95% CrI 5.60 to 12.00]), and liraglutide showing the greatest long−term risks of diarrhea (OR 26.00 [95% CrI 8.60 to 85.00]) and constipation (OR 22.00 [95% CrI 12.00 to 51.00]) (see Supplementary Appendix 13).

### 3.14 Subgroup analysis of combination therapy regimens

All IBTs combination regimens achieved significant reductions in HbA_1_c, with the optimal hypoglycemic effect observed in the regimens of tirzepatide 15 mg (MD −2.80% [95% CrI −3.40 to −2.20]) and 10 mg (MD −2.80% [95% CrI −3.30 to −2.20]) in combination with an oral hypoglycemic agent at a stable dose; regimens involving “combination with insulin” or “high−dose monotherapy” generally exerted more pronounced HbA_1_c−lowering effects (e.g., tirzepatide 15 mg plus insulin: MD −2.40%; semaglutide 1 mg monotherapy: MD −1.90%); FPG reduction, most regimens had significant effects, with the liraglutide 1.8 mg plus multiple antidiabetic drugs regimen being the most effective (MD −2.90 mmol/L [95% CrI −5.30 to −0.46]), high−dose tirzepatide (15 mg) across all regimens consistently demonstrating substantial FPG−lowering potential (monotherapy: MD −2.50 mmol/L; plus insulin: MD −2.40 mmol/L); in terms of weight reduction, most regimens produced significant effects, with the tirzepatide 15 mg plus stable oral antidiabetic drugs regimen showing the strongest efficacy (MD −10.00 kg [95% CrI −13.00 to −7.80]), high−dose tirzepatide (15 mg) across all regimens exhibiting marked weight−lowering effects (monotherapy: MD −9.40 kg; plus insulin: MD −8.10 kg) and the semaglutide 1 mg plus insulin regimen also having robust efficacy (MD −5.00 kg [95% CrI −7.00 to −3.00]), while the liraglutide 0.6 mg monotherapy regimen even showed a trend toward weight gain (MD 0.37 kg [95% CrI −2.10 to 2.90]) (see Supplementary Appendix 14).

## 4 Discussion

This network meta-analysis shows that IBTs deliver comprehensive benefits for T2DM. Tirzepatide, orforglipron and semaglutide achieve excellent glycemic control, while retatrutide is superior in weight reduction. Their effects on blood lipids and blood pressure vary by drug: liraglutide raises HDL, visepegenatide lowers LDL-C and TG, and exenatide reduces TC; orforglipron works best for SBP reduction, and visepegenatide is optimal for DBP management. In addition, tirzepatide exerts the strongest cardioprotective effects, and all IBTs have renal protective properties—polyethylene glycol loxenatide improves eGFR and exenatide decreases UACR. Tirzepatide and liraglutide also help optimize insulin function. Common adverse reactions included mild pulse rate elevation, nasopharyngitis, constipation, and injection site reactions (exenatide, lixisenatide, etc.) (Seino et al., 2012) Low-titer anti-drug antibodies were detected for some agents without impairing efficacy (Blonde et al., 2015). Hypoglycemia risk was low overall. Rare adverse events involved acute kidney injury (liraglutide, exenatide) (Gerstein et al., 2019), cholelithiasis and malignant neoplasms (semaglutide, liraglutide, dulaglutide) (Marre et al., 2009; Ahmann et al., 2015), and diabetic ketoacidosis (retatrutide) (Rosenstock et al., 2023). Despite minor agent-specific differences in adverse reaction incidence and manifestations, the overall safety profiles of IBTs remained consistent. In addition, safety shows clear dose-response relationships—higher doses enhance efficacy but increase adverse event risks, with orforglipron 45 mg achieving an optimal benefit-tolerability balance, while semaglutide, dulaglutide, and other IBTs also exhibit dose-dependent efficacy and formulation-related disparities (e.g., oral orforglipron 36 mg matches injectables in HbA_1_c or weight loss effects, and 45 mg reduces FBG close to injectables). Treatment duration impacts outcomes: most agents improve efficacy long-term, with divergent trends in gastrointestinal adverse events (e.g., exenatide lowers nausea or vomiting risks, tirzepatide reduces diarrhea risk) and varying short-/mid-/long-term efficacy across agents, while IBTs combination therapies yield drug-specific synergistic effects superior to monotherapy, further supporting individualized selection based on patient comorbidities (e.g., obesity, cardiorenal disease) to maximize therapeutic benefits while ensuring safety.

From the perspective of pathophysiological mechanisms, the differences in therapeutic efficacy among various incretin drugs can be partially attributed to the synergistic effects of receptor co-activation. Although the insulinotropic effect of GIP in patients with T2DM is significantly diminished due to impaired β-cell function (Ciardullo et al., 2024), resulting in limited hypoglycemic efficacy when used alone, complementary benefits are generated when it is co-activated with GLP-1RAs (Liu, 2024). As a dual GIP/GLP-1RAs, tirzepatide acts on two incretin signaling pathways simultaneously. It not only promotes insulin secretion in a glucose-dependent manner but also synergistically enhances satiety, suppresses appetite, and improves lipid metabolism. Furthermore, the addition of glucagon receptor agonism forms a triple receptor co-activation strategy. Against the backdrop of hypoglycemic and weight-lowering effects mediated by GLP-1RAs, moderate activation of glucagon receptors yields additional metabolic benefits by boosting energy expenditure, enhancing lipolysis, and improving hepatic insulin sensitivity (Elmendorf et al., 2026). Retatrutide, as a GIP/GLP-1/GCGR triple receptor agonist, relies precisely on this mechanism to demonstrate superimposed advantages in weight loss and glycemic control (Katsi et al., 2025). While glucagon activation itself does not directly lower blood glucose, its indirect effects mediated by improving insulin resistance and promoting adipose tissue remodeling produce synergistic benefits with the GIP and GLP-1 pathways (Reimann and Gribble, 2016). Therefore, the efficacy gradient among different drugs observed in this study essentially reflects the pathophysiological progressive logic from single-receptor activation to dual-receptor and even triple-receptor activation.

By comprehensively evaluating the benefit-risk profiles of various incretin-based drugs, this study found that although medications including tirzepatide, orforglipron and semaglutide statistically significant advantages in reducing HbA_1_c, their incidence of gastrointestinal adverse events (nausea, vomiting, diarrhea, constipation) increases correspondingly with a clear dose-response relationship. Specifically, as the administered dose escalates, the hypoglycemic efficacy is further improved, while the risk and severity of adverse events such as nausea and vomiting rise simultaneously, directly leading to treatment discontinuation in some patients. Subgroup analysis further confirmed that high-dose regimens (e.g., orforglipron 24 mg, efpeglenatide 12 mg) can achieve superior glycemic control but are associated with markedly reduced gastrointestinal tolerability and an elevated discontinuation rate. Notably, orforglipron 45 mg achieves a relatively optimal balance between efficacy and tolerability, whereas some drugs with high SUCRA rankings do not demonstrate an equivalent level of overall acceptability. This finding suggests that in real clinical practice, selecting the so-called “optimal” drugs solely based on the reduction in glycated hemoglobin or SUCRA ranking may overlook the critical impact of patient tolerability on treatment persistence. If patients discontinue medication early due to intolerance to gastrointestinal reactions, the net clinical benefit of drugs with potent hypoglycemic effects demonstrated in clinical trials will be greatly diminished in practical application. Therefore, clinical decision-making should comprehensively weigh hypoglycemic efficacy, dose-dependent side effects and anticipated discontinuation risks, and formulate individualized treatment regimens based on patients’ personal tolerability, comorbidities and dose titration strategies, rather than selecting drugs simply according to efficacy rankings.

In the meta-regression analysis of this study, diabetes duration and baseline HbA_1_c level were identified as important effect modifiers influencing treatment effects. The analysis results indicated that patients with longer disease duration and higher baseline glycated hemoglobin levels achieved a more significant absolute reduction in glycated hemoglobin after treatment with incretin-based medications, suggesting that patients with a heavier baseline disease burden may obtain greater net glycemic-lowering benefits. However, it is noteworthy that such patients often have a prolonged disease course, a heavier burden of complications, and may be accompanied by multiple cardiovascular and metabolic abnormalities simultaneously. They may also have lower gastrointestinal tolerance to high-dose regimens, which may affect treatment persistence and overall benefits. Therefore, when extrapolating the efficacy conclusions of this study to different clinical subgroups, full consideration should be given to the heterogeneity of baseline characteristics.

Existing systematic reviews and meta-analyses are often restricted to limited number of agent types or focus on single outcomes, thereby failing to address the guideline-recommended multi-target therapeutic goals or adequately support individualized treatment strategies for patients with multiple comorbidities. This network meta-analysis has distinct strengths. First, it boasts an exceptionally comprehensive scope, solving problems such as the single research index and few types of drugs in similar studies. Second, stratified subgroup analyses of dosages, treatment courses, and combination therapies were conducted to address individualized clinical needs, clarifying efficacy-safety balancing strategies. Third, outcome measures spanned glycemic control, weight regulation, lipid metabolism, cardiovascular events, and renal function, enabling a holistic assessment of drug benefits. Fourth, strict adherence to PRISMA 2020 and PRISMA-NMA guidelines, PROSPERO registration, rigorous bias risk assessment, and robustness verification collectively ensure high result reliability. Notwithstanding these merits, the study has several limitations. The paucity of RCTs for some IBTs restricts analyzable indicators, and scarce head-to-head trials between specific agents necessitate heavy reliance on indirect evidence. The predominantly adult cohort excludes pediatric, adolescent, and severely comorbid patients, limiting conclusion generalizability. Additionally, data on rare adverse events and long-term efficacy stability beyond 2 years remain insufficient; subjective outcomes (e.g., quality of life) were not assessed, constraining comprehensive clinical decision support. Notably, unanalyzed agents do not equate to ineffectiveness but reflect evidence gaps warranting further trials.

Clinical trial data for some novel drugs remain relatively limited. In such cases, high SUCRA rankings should be interpreted with caution. As a probabilistic ranking indicator, the stability of SUCRA is highly dependent on the quality and precision of the evidence included in the analysis. Small sample sizes, large variances or sparse network structures in primary studies can render ranking results vulnerable to random fluctuations, model assumptions and potential biases, which may lead to inflated rankings and overestimation of the actual efficacy of drugs. Therefore, for novel drugs with insufficient supporting evidence, SUCRA analysis results can only serve as exploratory conclusions rather than definitive judgments.

Given the exploratory nature of network meta-analysis, and the fact that most comparisons rely on indirect evidence, surrogate endpoints or lack of direct head-to-head trials, the drug treatment recommendations based on different comorbidities presented in this study should be regarded as hypothesis-generating preliminary findings instead of definitive clinical guidance. For rankings derived from limited evidence or a small number of studies, the seemingly superior efficacy of certain drugs may not be robust and needs to be verified in well-powered randomized controlled trials with direct comparisons.

The preliminary findings are summarized as follows: Among obese patients, retatrutide exhibits the best weight loss effect, yet direct comparisons with other dual or triple agonists are lacking. In terms of dyslipidemia, the rankings indicating that liraglutide increases HDL, visepegenatide reduces LDL-C and TG, and exenatide lowers TC are all based on limited evidence, with visepegenatide having the weakest evidence base and its results deemed exploratory. For hypertension management, the findings that orforglipron reduces systolic blood pressure and visepegenatide decreases diastolic blood pressure are solely derived from indirect comparisons and surrogate endpoints, and require confirmation via dedicated direct antihypertensive studies. In patients with concomitant ASCVD, tirzepatide demonstrates superior benefits in MACE, while hard endpoint data for many other drugs remain inadequate. Among patients with CKD, the conclusions that pegylated lixisenatide improves eGFR and exenatide reduces UACR come from studies with small sample sizes and wide confidence intervals, and should be recognized as preliminary results. Regarding impaired insulin function, the benefits of tirzepatide on HOMA-β and liraglutide on HOMA-IR still need further validation using clinically relevant indicators. For patients with multiple comorbidities, tirzepatide or 45 mg orforglipron may be considered for their broad-spectrum efficacy, though direct comparisons with combination therapy regimens are unavailable. Clinicians shall combine the above exploratory findings with patients’ individual tolerability, treatment history and the strength of direct evidence for each drug. Dose titration, monitoring of gastrointestinal tolerance and lifestyle interventions remain essential components of clinical management. There is an urgent need to conduct head-to-head randomized controlled trials focusing on clinically relevant endpoints in the future to validate or refute these exploratory rankings. Furthermore, this study further defines the applicable populations for different incretin-based therapies. It is suggested that subsequent guidelines incorporate stratified recommendations for various preparations and clarify the preferred indications of each drug according to comorbidity types, so as to improve the clinical practicality of the guidelines. In terms of health policies, the medical insurance reimbursement policies for IBTs should be further optimized to improve drug accessibility, especially for oral IBTs and dual-receptor or triple-receptor agonists, so that more T2DM patients can receive high-quality glucose-lowering treatment. Meanwhile, efforts should be made to strengthen the construction of a monitoring system for adverse drug reactions of IBTs, establish a national adverse reaction database, and achieve early identification and warning of rare adverse reactions. In addition, more investment should be increased in clinical research on IBTs to support studies on special populations, long-term efficacy, and pharmacoeconomics, fill evidence gaps, and provide a more comprehensive evidence-based basis for the precise treatment of T2DM.

The findings of this network meta-analysis are generally consistent with major international guidelines for the management of T2DM, including the ADA 2025, EASD/ADA 2025 Consensus, IDF 2025 and CDS 2024. All these guidelines endorse the multi-dimensional benefits of IBTs in glycemic control, weight management and cardiorenal protection. Moreover, the efficacy of top-ranked agents such as tirzepatide provides quantitative evidence for the “cardiorenal priority” strategy recommended in the guidelines. Nevertheless, this study presents discrepancies from existing guidelines. It reveals that the high SUCRA rankings of several novel agents including orforglipron and retatrutide are derived from limited indirect evidence, suggesting that guidelines should interpret purely statistical rankings with caution. Additionally, the dose-subgroup analyses in this study, such as the favorable balance between benefits and tolerability of 45 mg orforglipron, as well as the substantial heterogeneity in gastrointestinal tolerability across different IBTs, call for the inclusion of more refined dose stratification and individualized tolerability recommendations in future guidelines.

## 5 Conclusion

In conclusion, this large-scale and comprehensive network meta-analysis confirms that IBTs yield significant multi-dimensional benefits in the treatment of T2DM, with marked heterogeneity in efficacy and safety among different drugs. Dosage, treatment duration and combination regimens exert important modulating effects on clinical outcomes. Nevertheless, the drug rankings and comparative results derived from this analysis are mainly based on indirect evidence and surrogate endpoints such as glycated hemoglobin, body weight and blood pressure. Some drugs are even evaluated relying on sparse data or limited head-to-head trials. Accordingly, the aforementioned rankings should be regarded as exploratory findings rather than definitive conclusions. In clinical practice, any preference for a specific IBT based on these results needs to be balanced against direct evidence, patients’ comorbidities, drug tolerability and shared decision-making. There is an urgent need for high-quality, long-term head-to-head randomized controlled trials focusing on hard clinical endpoints including MACE, mortality and renal failure, especially for newly developed drugs. Until such evidence becomes available, treatment decisions should prioritize a comprehensive assessment of the overall weight of both direct and indirect evidence and strictly adhere to the principle of individualized treatment.

## Data Availability

The original contributions presented in the study are included in the article/Supplementary Material, further inquiries can be directed to the corresponding author.

## Glossary

T2DM: type 2 diabetes mellitus
GLP-1RA: glucagon-like peptide-1 receptor agonist
GIP: glucose-dependent insulinotropic polypeptide
GCGR: glucagon receptor
GLP-1/GCGR: GLP-1/glucagon receptor agonist
GIP/GLP-1RA: GIP/GLP-1 receptor agonist
GIP/GLP-1/GCGR: GIP/GLP-1/glucagon receptor agonist
IBTs: incretin-based therapies
ASCVD: atherosclerotic cardiovascular disease
CKD: chronic kidney disease
MASLD: metabolic dysfunction-associated steatotic liver disease
MASH: metabolic dysfunction-associated steatohepatitis
ADA: American Diabetes Association
RCTs: randomized controlled trials
PRISMA: Preferred Reporting Items for Systematic Reviews and Meta-Analyses
NMA: network meta-analysis
PROSPERO: International Prospective Register of Systematic Reviews
RoB 2.0: Cochrane Risk of Bias Tool for Randomized Trials (version 2.0)
CINeMA: Confidence in Network Meta-Analysis
SUCRA: Surface Under the Cumulative Ranking
MCMC: Markov Chain Monte Carlo
PSRF: Potential Scale Reduction Factor
MD: mean difference
OR: odds ratio
CrI: credible interval
SD: standard deviation
**HbA**_**1c**_: glycated hemoglobin
FPG: fasting plasma glucose
SBP: systolic blood pressure
DBP: diastolic blood pressure
HDL: high-density lipoprotein
LDL: low-density lipoprotein
TC: total cholesterol
TG: triglyceride
ACM: all-cause mortality
NFS: non-fatal stroke
NFM: non-fatal myocardial infarction
CVM: cardiovascular mortality
MACE: major adverse cardiovascular events
eGFR: estimated glomerular filtration rate
UACR: urinary albumin/creatinine ratio
FIL: fasting insulin level
HOMA-β: homeostasis model assessment of β-cell function
HOMA-IR: homeostasis model assessment of insulin resistance and
EASD: European Association for the Study of Diabetes

## References

[B1] Ahmann A. Rodbard H. W. Rosenstock J. Lahtela J. T. de Loredo L. Tornøe K. (2015). Efficacy and safety of liraglutide versus placebo added to basal insulin analogues (with or without metformin) in patients with type 2 diabetes: a randomized, placebo-controlled trial. Diabetes Obes. Metab. 17 (11), 1056–1064. 10.1111/dom.12539 26179619 PMC5054929

[B2] American Diabetes Association Professional Practice Committee (2025). Standards of care in Diabetes—2025. Diabetes Care 48 (Suppl. 1), S1–S352. 10.2337/dc25-S011 39651982

[B3] Blonde L. Jendle J. Gross J. Woo V. Jiang H. Fahrbach J. L. (2015). Once-weekly dulaglutide versus bedtime insulin glargine, both in combination with prandial insulin lispro, in patients with type 2 diabetes (AWARD-4): a randomised, open-label, phase 3, non-inferiority study. Lancet 385 (9982), 2057–2066. 10.1016/S0140-6736(15)60936-9 26009229

[B4] Chen X. Zhang L. Chen W. (2025). Global, regional, and national burdens of type 1 and type 2 diabetes mellitus in adolescents from 1990 to 2021, with forecasts to 2030: a systematic analysis of the global burden of disease study 2021. BMC Med. 23 (1), 48. 10.1186/s12916-025-03890-w 39876009 PMC11776159

[B5] Ciardullo S. Morieri M. L. Daniele G. Fiorentino T. V. Mezza T. Tricò D. (2024). GLP1-GIP receptor co-agonists: a promising evolution in the treatment of type 2 diabetes. Acta Diabetol. 61 (8), 941–950. 10.1007/s00592-024-02300-6 38831203 PMC11329401

[B6] Elmendorf A. J. Yousefian M. Kim I. M. Hardaway J. A. Habegger K. Flak J. N. (2026). IUPHAR review: from foe to friend: repurposing glucagon to treat obesity and type 2 diabetes. Pharmacol. Res. 223, 108077. 10.1016/j.phrs.2025.108077 41478576 PMC12910462

[B7] Gerstein H. C. Colhoun H. M. Dagenais G. R. Diaz R. Lakshmanan M. Pais P. (2019). Dulaglutide and cardiovascular outcomes in type 2 diabetes (REWIND): a double-blind, randomised placebo-controlled trial. Lancet 394 (10193), 121–130. 10.1016/S0140-6736(19)31149-3 31189511

[B8] Higgins J. P. Thompson S. G. Deeks J. J. Altman D. G. (2003). Measuring inconsistency in meta-analyses. BMJ 327 (7414), 557–560. 10.1136/bmj.327.7414.557 12958120 PMC192859

[B9] Hutton B. Salanti G. Caldwell D. M. Chaimani A. Schmid C. H. Cameron C. (2015). The PRISMA extension statement for reporting of systematic reviews incorporating network meta-analyses of health care interventions: checklist and explanations. Ann. Intern Med. 162 (11), 777–784. 10.7326/M14-2385 26030634

[B10] Katsi V. Koutsopoulos G. Fragoulis C. Dimitriadis K. Tsioufis K. (2025). Retatrutide—A game changer in obesity pharmacotherapy. Biomolecules 15 (6), 796. 10.3390/biom15060796 40563436 PMC12190491

[B11] Liu Q. K. (2024). Mechanisms of action and therapeutic applications of GLP-1 and dual GIP/GLP-1 receptor agonists. Front. Endocrinol. (Lausanne) 15, 1431292. 10.3389/fendo.2024.1431292 39114288 PMC11304055

[B12] Liu Y. Béliveau A. Wei Y. Chen M. Y. Record-Lemon R. Kuo P. L. (2023). A gentle introduction to Bayesian network meta-analysis using an automated R package. Multivar. Behav. Res. 58 (4), 706–722. 10.1080/00273171.2022.2115965 36254763

[B13] Liu B. Li L. Cui H. Zhao Q. Chen S. (2024). Analysis of the global burden of CKD-T2DM in young and middle-aged adults in 204 countries and territories from 2000 to 2019: a systematic study of the global burden of disease in 2019. Diabetes Res. Clin. Pract. 217, 111884. 10.1016/j.diabres.2024.111884 39389473

[B14] Luciani L. Pedrelli M. Parini P. (2024). Modification of lipoprotein metabolism and function driving atherogenesis in diabetes. Atherosclerosis 394, 117545. 10.1016/j.atherosclerosis.2024.117545 38688749

[B15] Marre M. Shaw J. Brändle M. Bebakar W. M. W. Kamaruddin N. A. Strand J. (2009). Liraglutide, a once-daily human GLP-1 analogue, added to a sulphonylurea over 26 weeks produces greater improvements in glycaemic and weight control compared with adding rosiglitazone or placebo in subjects with type 2 diabetes (LEAD-1 SU). Diabet. Med. 26 (3), 268–278. 10.1111/j.1464-5491.2009.02666.x 19317822 PMC2871176

[B16] Mendonça L. Moura H. Chaves P. C. Neves J. S. Ferreira J. P. (2025). The impact of glucagon-like peptide-1 receptor agonists on kidney outcomes: a meta-analysis of randomized placebo-controlled trials. Clin. J. Am. Soc. Nephrol. 20 (2), 159–168. 10.2215/CJN.0000000584 39480988 PMC11835157

[B17] National Health Commission of the People's Republic of China (2025). Guidelines for the prevention and treatment of diabetes in China (2024 edition). Chin. J. Diabetes Mellit. 17 (1), 16–139. 10.3760/cma.j.cn112138-20250924-00572

[B18] Noma H. (2023). Bayesian estimation and prediction for network meta-analysis with contrast-based approach. Int. J. Biostat. 20 (2), 661–676. 10.1515/ijb-2022-0121 37401787

[B19] Page M. J. McKenzie J. E. Bossuyt P. M. Boutron I. Hoffmann T. C. Mulrow C. D. (2021). The PRISMA 2020 statement: an updated guideline for reporting systematic reviews. BMJ 372, n71. 10.1136/bmj.n71 33782057 PMC8005924

[B20] Reimann F. Gribble F. M. (2016). Mechanisms underlying glucose-dependent insulinotropic polypeptide and glucagon-like peptide-1 secretion. J. Diabetes Investig. 7 (Suppl. 1), 13–19. 10.1111/jdi.12478 27186350 PMC4854499

[B21] Rosenstock J. Frias J. Jastreboff A. M. Du Y. Lou J. Gurbuz S. (2023). Retatrutide, a GIP, GLP-1 and glucagon receptor agonist, for people with type 2 diabetes: a randomised, double-blind, placebo and active-controlled, parallel-group, phase 2 trial conducted in the USA. Lancet 402 (10401), 529–544. 10.1016/S0140-6736(23)01053-X 37385280

[B22] Seino Y. Min K. W. Niemoeller E. Takami A. EFC10887 GETGOAL-L Asia Study Investigators (2012). EFC10887 GETGOAL-L Asia study investigators. Randomized, double-blind, placebo-controlled trial of the once-daily GLP-1 receptor agonist lixisenatide in Asian patients with type 2 diabetes insufficiently controlled on basal insulin with or without a sulfonylurea (GetGoal-L-Asia). Diabetes Obes. Metab. 14 (10), 910–917. 10.1111/j.1463-1326.2012.01618.x 22564709 PMC3466411

[B23] Singh A. Shadangi S. Gupta P. K. Rana S. (2025). Type 2 diabetes mellitus: a comprehensive review of pathophysiology, comorbidities, and emerging therapies. Compr. Physiol. 15 (1), e70003. 10.1002/cph4.70003 39980164

[B24] Stefanou M. I. Palaiodimou L. Theodorou A. Safouris A. Fischer U. Kelly P. J. (2024). Risk of major adverse cardiovascular events and all-cause mortality under treatment with GLP-1 RAs or the dual GIP/GLP-1 receptor agonist tirzepatide in overweight or Obese adults without diabetes: a systematic review and meta-analysis. Ther. Adv. Neurol. Disord. 17, 17562864241281903. 10.1177/17562864241281903 39345822 PMC11437580

[B25] Sterne J. A. Egger M. Smith G. D. (2001). Systematic reviews in health care: investigating and dealing with publication and other biases in meta-analysis. BMJ 323 (7304), 101–105. 10.1136/bmj.323.7304.101 11451790 PMC1120714

[B26] Sterne J. A. C. Savović J. Page M. J. Elbers R. G. Blencowe N. S. Boutron I. (2019). RoB 2: a revised tool for assessing risk of bias in randomised trials. BMJ 366, l4898. 10.1136/bmj.l4898 31462531

[B27] Theodorakis N. Nikolaou M. (2025). From cardiovascular-kidney-metabolic syndrome to cardiovascular-renal-hepatic-metabolic syndrome: proposing an expanded framework. Biomolecules 15 (2), 213. 10.3390/biom15020213 40001516 PMC11853431

[B28] Thomas M. C. Cooper M. E. Zimmet P. (2016). Changing epidemiology of type 2 diabetes mellitus and associated chronic kidney disease. Nat. Rev. Nephrol. 12 (2), 73–81. 10.1038/nrneph.2015.173 26553517

[B29] van Valkenhoef G. Dias S. Ades A. E. Welton N. J. (2016). Automated generation of node-splitting models for assessment of inconsistency in network meta-analysis. Res. Synth. Methods 7 (1), 80–93. 10.1002/jrsm.1167 26461181 PMC5057346

[B30] Waldrop G. Zhong J. Peters M. Goud A. Chen Y. H. Davis S. N. (2018). Incretin-based therapy in type 2 diabetes: an evidence based systematic review and meta-analysis. J. Diabetes Complicat. 32 (1), 113–122. 10.1016/j.jdiacomp.2016.08.018 29074120

[B31] Wu S. Gao L. Cipriani A. Huang Y. Yang Z. Yang J. (2019). The effects of incretin-based therapies on β-cell function and insulin resistance in type 2 diabetes: a systematic review and network meta-analysis combining 360 trials. Diabetes Obes. Metab. 21 (4), 975–983. 10.1111/dom.13613 30536884

