# Supplementary material for: Efficacy and safety of incretin-based therapies in patients with type 2 diabetes mellitus: a network meta-analysis based on clinical trials
Source: Front Pharmacol. 2026 Jun 18;17:1846714. doi: 10.3389/fphar.2026.1846714 (PMC13322860; doi:10.3389/fphar.2026.1846714)

Supplementary

Efficacy and Safety of Incretin-Based Therapies in patients with Type 2 Diabetes Mellitus: A Network Meta-Analysis Based on Clinical Trials

Table of contents

Supplementary 1: Search strategy Supplementary Material *2*

Supplementary 2: Characteristics of included studies *7*

Supplementary 3: List of data extracted from the included randomized clinical trials *56*

Supplementary 4: Risk of bias of randomized clinical trials *57*

Supplementary 5: Evaluation of inconsistency and heterogeneity *61*

Supplementary 6: Network maps and forest plots of secondary outcome *73*

Supplementary 7: SUCRA and stacked sort charts *94*

Supplementary 8: league table of Summary Estimates for GLP-1RAs on T2D Derived from Network Meta-analysis of 102 Trials *122*

Suplementary 9: Funnel plots and CINeMA *149*

Suplementary 10: Sensitivity analyses *192*

Appendix 11: The meta-regression of the factors that may lead to differences to the main outcome indicators *194*

Suplementary 12: Effects of different doses of GLP-1RAs on various indicators of T2DM. *195*

Suplementary 13: Effects of treatment durations of different GLP-1 receptor agonists on various indicators in T2DM. *202*

Suplementary14: Effects of different combination therapy regimens of GLP-1 receptor agonists on various indicators of T2DM. *209*

Supplementary 1: Search strategy Supplementary Material

Table S1. Search strategy of Medline

**1**	(GLP-1 receptor agonis*[Title/Abstract]) OR (Glucagon-like peptide-1 agonis*[Title/Abstract]) OR (Incretin mimeti*[Title/Abstract]) OR (GLP-1 analo*[Title/Abstract]) OR ("Glucagon-Like Peptide 1"[MeSH Terms]) OR ("glucagon like peptide 1 receptor"[MeSH Terms])
**2**	(liraglutide[Title/Abstract] OR Victoza[Title/Abstract] OR Saxenda[Title/Abstract] OR SAXE[Title/Abstract]) OR (semaglutide[Title/Abstract] OR Ozempic[Title/Abstract] OR Wegovy[Title/Abstract] OR Rybelsus[Title/Abstract]) OR (tirzepatide[Title/Abstract] OR Mounjaro[Title/Abstract] OR NN-9535[Title/Abstract] OR NN-9536[Title/Abstract] OR NN-9931[Title/Abstract] OR NN9924[Title/Abstract] OR NNC-0113-0217[Title/Abstract]) OR (exenatide[Title/Abstract] OR Byetta[Title/Abstract] OR Bydureon[Title/Abstract] OR "Exendin 4"[Title/Abstract] OR EX-4[Title/Abstract] OR AC-2993[Title/Abstract] OR "AC 2993-LAR"[Title/Abstract] OR LY-2148568[Title/Abstract] OR LY2148568[Title/Abstract] OR "des-38-proline-exendine-4"[Title/Abstract]) OR (dulaglutide[Title/Abstract] OR Trulicity[Title/Abstract] OR NN-2211[Title/Abstract] OR NN-9211[Title/Abstract] OR "NNC-90 1170"[Title/Abstract] OR NN-8022[Title/Abstract] OR NNC-901170[Title/Abstract] OR "NNC 90-1170"[Title/Abstract] OR NN 9211[Title/Abstract] OR NN 2211[Title/Abstract]) OR (lixisenatide[Title/Abstract] OR Lyxumia[Title/Abstract] OR Adlyxin[Title/Abstract]) OR efpeglenatide[Title/Abstract] OR ("polyethylene glycol loxenatide"[Title/Abstract] OR "PEG loxenatide"[Title/Abstract] OR pegloxenatide[Title/Abstract] OR PEX-168[Title/Abstract] OR PEX168[Title/Abstract] OR loxenatide[Title/Abstract]) OR (mazdutide[Title/Abstract] OR IBI-362[Title/Abstract] OR IBI362[Title/Abstract] OR OXM-3[Title/Abstract] OR OXM3[Title/Abstract]) OR "ITCA 650"[Title/Abstract] OR (orforglipron[Title/Abstract] OR LY3502970[Title/Abstract] OR LY-3502970[Title/Abstract] OR "OWL 833"[Title/Abstract]) OR (retatrutide[Title/Abstract] OR LY3437943[Title/Abstract] OR LY-3437943[Title/Abstract]) OR vispegenatide[Title/Abstract] OR cotadutide[Title/Abstract] OR survodutide[Title/Abstract] OR BI 456906[Title/Abstract] OR GZR18[Title/Abstract] OR "Bofanglutide"[Title/Abstract]
**3**	(diabetes[Title/Abstract] OR T2D[Title/Abstract] OR NIDDM[Title/Abstract] OR hyperglyc*[Title/Abstract] OR prediabet*[Title/Abstract] OR "type 2"[Title/Abstract] OR "type II"[Title/Abstract] OR "Diabetes Mellitus"[MeSH Terms] OR "Diabetes Mellitus, Type 2"[MeSH Terms] OR "Hyperglycemia"[MeSH Terms] OR "Prediabetic State"[MeSH Terms])

**4**	(IFG[Title/Abstract] OR "impaired FPG"[Title/Abstract] OR "glucose intolerance"[Title/Abstract] OR IGT[Title/Abstract] OR "impaired glucose"[Title/Abstract])
**5**	("major adverse cardiovascular events"[Title/Abstract] OR myocardial infarction[Title/Abstract] OR "heart failure"[Title/Abstract] OR stroke[Title/Abstract] OR "chronic kidney disease"[Title/Abstract] OR "estimated glomerular filtration rate"[Title/Abstract] OR albuminuria[Title/Abstract] OR "insulin secretion"[Title/Abstract] OR "insulin sensitivity"[Title/Abstract])
**6**	#1 OR #2
**7**	#3 OR #4 OR #5
**8**	#6 AND #7
**9**	("Meta-Analysis"[MeSH Terms] OR "Meta-Analysis as Topic"[MeSH Terms] OR "Meta-Analysis"[Publication Type] OR "Review"[Publication Type] OR "meta analysis"[Title/Abstract])
**10**	#8 NOT #9
**11**	("Randomized Controlled Trial"[Publication Type] OR "Controlled Clinical Trial"[Publication Type] OR "Clinical Trials as Topic"[MeSH Terms] OR "Random Allocation"[MeSH Terms] OR "Randomized Controlled Trials as Topic"[MeSH Terms] OR "Clinical Trial"[Publication Type] OR "clinical trial"[Text Word] OR "singl*"[Text Word] OR "doubl*"[Text Word] OR "trebl*"[Text Word] OR "tripl*"[Text Word] OR "randomly"[Text Word] OR "trial"[Text Word])
**12**	#10 AND #11

Table S2. Search strategy of Cochrane Central Register of Controlled Trials (CENTRAL)

**1**	("GLP-1 receptor agonist*"):ti,ab,kw OR ("Glucagon-like peptide-1 agonist*"):ti,ab,kw OR ("Incretin mimetic*"):ti,ab,kw OR ("GLP-1 analog*"):ti,ab,kw OR ("Glucagon-Like Peptide 1"):ti,ab,kw OR ("glucagon like peptide 1 receptor*"):ti,ab,kw OR ("GLP-1"):ti,ab,kw
**2**	("liraglutide"):ti,ab,kw OR ("Victoza"):ti,ab,kw OR ("Saxenda"):ti,ab,kw OR ("SAXE"):ti,ab,kw
**3**	("semaglutide"):ti,ab,kw OR ("Ozempic"):ti,ab,kw OR ("Wegovy"):ti,ab,kw OR ("Rybelsus"):ti,ab,kw
**4**	("tirzepatide"):ti,ab,kw OR ("Mounjaro"):ti,ab,kw OR ("NN-9535"):ti,ab,kw OR ("NN-9536"):ti,ab,kw OR ("NN-9931"):ti,ab,kw OR ("NN9924"):ti,ab,kw OR ("NNC-0113-0217"):ti,ab,kw
**5**	("exenatide"):ti,ab,kw OR ("Byetta"):ti,ab,kw OR ("Bydureon"):ti,ab,kw OR ("Exendin 4"):ti,ab,kw OR ("EX-4"):ti,ab,kw OR ("AC-2993"):ti,ab,kw OR ("AC 2993-LAR"):ti,ab,kw OR ("LY-2148568"):ti,ab,kw OR ("LY2148568"):ti,ab,kw OR ("des-38-proline-exendin-4"):ti,ab,kw
**6**	("dulaglutide"):ti,ab,kw OR ("Trulicity"):ti,ab,kw OR ("NN-2211"):ti,ab,kw OR ("NN-9211"):ti,ab,kw OR ("NNC-901170"):ti,ab,kw OR ("NN-8022"):ti,ab,kw OR ("NN 2211"):ti,ab,kw OR ("NN 9211"):ti,ab,kw

**7**	("lixisenatide"):ti,ab,kw OR ("Lyxumia"):ti,ab,kw OR ("Adlyxin"):ti,ab,kw OR ("efpeglenatide"):ti,ab,kw
**8**	("polyethylene glycol loxenatide"):ti,ab,kw OR ("PEG loxenatide"):ti,ab,kw OR ("pegloxenatide"):ti,ab,kw OR ("PEX-168"):ti,ab,kw OR ("PEX168"):ti,ab,kw OR ("loxenatide"):ti,ab,kw
**9**	("mazdutide"):ti,ab,kw OR ("IBI-362"):ti,ab,kw OR ("IBI362"):ti,ab,kw OR ("OXM-3"):ti,ab,kw OR ("OXM3"):ti,ab,kw OR ("ITCA 650"):ti,ab,kw
**10**	("orforglipron"):ti,ab,kw OR ("LY3502970"):ti,ab,kw OR ("LY-3502970"):ti,ab,kw OR ("OWL 833"):ti,ab,kw
**11**	("retatrutide"):ti,ab,kw OR ("LY3437943"):ti,ab,kw OR ("LY-3437943"):ti,ab,kw OR ("vispegenatide"):ti,ab,kw
**12**	("cotadutide"):ti,ab,kw OR ("survodutide"):ti,ab,kw OR ("BI 456906"):ti,ab,kw
**13**	#1 or #2 or #3 or #4 or #5 or #6 or #7 or #8 or #9 or #10 or #11 or #12
**14**	("type 2 diabetes"):ti,ab,kw OR ("diabetes mellitus, type 2"):ti,ab,kw OR ("diabetes, type 2"):ti,ab,kw OR ("type 2 diabetes mellitus"):ti,ab,kw OR ("type II diabetes"):ti,ab,kw OR ("type II diabetes mellitus"):ti,ab,kw
**15**	("T2D"):ti,ab,kw OR ("NIDDM"):ti,ab,kw OR ("hyperglyc*"):ti,ab,kw OR ("prediabet*"):ti,ab,kw OR ("type 2 diabetes"):ti,ab,kw OR ("type 2 diabetes mellitus"):ti,ab,kw
**16**	("IFG"):ti,ab,kw OR ("impaired FPG"):ti,ab,kw OR ("glucose intolerance"):ti,ab,kw OR ("IGT"):ti,ab,kw OR ("impaired glucose tolerance"):ti,ab,kw OR ("impaired fasting glucose"):ti,ab,kw
**17**	("major adverse cardiovascular events"):ti,ab,kw OR ("myocardial infarction"):ti,ab,kw OR ("heart failure"):ti,ab,kw OR ("stroke"):ti,ab,kw OR ("chronic kidney disease"):ti,ab,kw OR ("estimated glomerular filtration rate"):ti,ab,kw OR ("albuminuria"):ti,ab,kw OR ("insulin secretion"):ti,ab,kw OR ("insulin sensitivity"):ti,ab,kw OR ("MACE"):ti,ab,kw OR ("eGFR"):ti,ab,kw OR ("glomerular filtration rate"):ti,ab,kw
**18**	#14 or #15 or #16 #17
**19**	#13 and #18
**20**	("meta-analysis"):ti,ab,kw OR ("systematic review"):ti,ab,kw OR ("review"):ti,ab,kw OR ("meta analysis"):ti,ab,kw
**21**	#19 not #20
**22**	("randomized clinical trial"):ti,ab,kw OR ("controlled clinical trial"):ti,ab,kw OR ("clinical trial"):ti,ab,kw OR ("random allocation"):ti,ab,kw OR ("randomised clinical trial"):ti,ab,kw OR ("RCT"):ti,ab,kw
**23**	("clinical trial"):ti,ab,kw OR ("single-blind"):ti,ab,kw OR ("double-blind"):ti,ab,kw OR ("triple-blind"):ti,ab,kw OR ("randomly assigned"):ti,ab,kw OR ("randomly allocated"):ti,ab,kw
**24**	("trial"):ti,ab,kw OR ("clinical trial"):ti,ab,kw OR ("study group"):ti,ab,kw OR ("treatment group"):ti,ab,kw OR ("control group"):ti,ab,kw
**25**	#22 or #23 or #24
**26**	#21 and #25

Table S3. Search strategy of Embase

**1**	('GLP-1 receptor agonis*':ti,ab,kw OR 'Glucagon-like peptide-1 agonis*':ti,ab,kw OR 'Incretin mimeti*':ti,ab,kw OR 'GLP-1 analo*':ti,ab,kw OR 'Glucagon-Like Peptide 1*':ti,ab,kw OR 'glucagon like peptide 1 receptor*':ti,ab,kw)
**2**	('liraglutide':ti,ab,kw OR 'Victoza':ti,ab,kw OR 'Saxenda':ti,ab,kw OR 'SAXE':ti,ab,kw) OR ('semaglutide':ti,ab,kw OR 'Ozempic':ti,ab,kw OR 'Wegovy':ti,ab,kw OR 'Rybelsus':ti,ab,kw) OR ('tirzepatide':ti,ab,kw OR 'Mounjaro':ti,ab,kw OR 'NN-9535':ti,ab,kw OR 'NN-9536':ti,ab,kw OR 'NN-9931':ti,ab,kw OR 'NN9924':ti,ab,kw OR 'NNC-0113-0217':ti,ab,kw) OR ('exenatide':ti,ab,kw OR 'Byetta':ti,ab,kw OR 'Bydureon':ti,ab,kw OR 'Exendin 4':ti,ab,kw OR 'EX-4':ti,ab,kw OR 'AC-2993':ti,ab,kw OR 'AC 2993-LAR':ti,ab,kw OR 'LY-2148568':ti,ab,kw OR 'LY2148568':ti,ab,kw OR 'des-38-proline-exendine-4':ti,ab,kw) OR ('dulaglutide':ti,ab,kw OR 'Trulicity':ti,ab,kw OR 'NN-2211':ti,ab,kw OR 'NN-9211':ti,ab,kw OR 'NNC-90 1170':ti,ab,kw OR 'NN-8022':ti,ab,kw OR 'NNC-901170':ti,ab,kw OR 'NNC 90-1170':ti,ab,kw OR 'NN 9211':ti,ab,kw OR 'NN 2211':ti,ab,kw) OR ('Lixisenatide':ti,ab,kw OR 'Lyxumia':ti,ab,kw OR 'Adlyxin':ti,ab,kw OR 'Efpeglenatide':ti,ab,kw OR 'polyethylene glycol loxenatide':ti,ab,kw OR 'PEG loxenatide':ti,ab,kw OR 'pegloxenatide':ti,ab,kw OR 'PEX-168':ti,ab,kw OR 'PEX168':ti,ab,kw OR 'loxenatide':ti,ab,kw) OR ('Mazdutide':ti,ab,kw OR 'IBI-362':ti,ab,kw OR 'IBI362':ti,ab,kw OR 'OXM-3':ti,ab,kw OR 'OXM3':ti,ab,kw) OR ('ITCA 650':ti,ab,kw OR 'Orforglipron':ti,ab,kw OR 'LY3502970':ti,ab,kw OR 'LY-3502970':ti,ab,kw OR 'OWL 833':ti,ab,kw) OR ('Retatrutide':ti,ab,kw OR 'LY3437943':ti,ab,kw OR 'LY-3437943':ti,ab,kw) OR ('Vispegenatide':ti,ab,kw OR 'Cotadutide':ti,ab,kw) OR ('Survodutide':ti,ab,kw OR 'BI 456906':ti,ab,kw)
**3**	('liraglutide'/exp OR 'Victoza'/exp OR 'Saxenda'/exp OR 'SAXE'/exp) OR ('semaglutide'/exp OR 'Ozempic'/exp OR 'Wegovy'/exp OR 'Rybelsus'/exp) OR ('tirzepatide'/exp OR 'Mounjaro'/exp OR 'NN-9535'/exp OR 'NN-9536'/exp OR 'NN-9931'/exp OR 'NN9924'/exp OR 'NNC-0113-0217'/exp) OR ('exenatide'/exp OR 'Byetta'/exp OR 'Bydureon'/exp OR 'Exendin 4'/exp OR 'EX-4'/exp OR 'AC-2993'/exp OR 'AC 2993-LAR'/exp OR 'LY-2148568'/exp OR 'LY2148568'/exp OR 'des-38-proline-exendine-4'/exp) OR ('dulaglutide'/exp OR 'Trulicity'/exp OR 'NN-2211'/exp OR 'NN-9211'/exp OR 'NNC-90 1170'/exp OR 'NN-8022'/exp OR 'NNC-901170'/exp OR 'NNC 90-1170'/exp OR 'NN 9211'/exp OR 'NN 2211'/exp) OR ('Lixisenatide'/exp OR 'Lyxumia'/exp OR 'Adlyxin'/exp OR 'Efpeglenatide'/exp OR 'polyethylene glycol loxenatide'/exp OR 'PEG loxenatide'/exp OR 'pegloxenatide'/exp OR 'PEX-168'/exp OR 'PEX168'/exp OR 'loxenatide'/exp) OR ('Mazdutide'/exp OR 'IBI-362'/exp OR 'IBI362'/exp OR 'OXM-3'/exp OR 'OXM3'/exp) OR ('ITCA 650'/exp OR 'Orforglipron'/exp OR 'LY3502970'/exp OR 'LY-

	3502970'/exp OR 'OWL 833'/exp) OR ('Retatrutide'/exp OR 'LY3437943'/exp OR 'LY-3437943'/exp) OR ('Vispegenatide'/exp OR 'Cotadutide'/exp) OR ('Survodutide'/exp OR 'BI 456906'/exp)
**4**	('type 2 diabetes':ti,ab,kw OR 'diabetes mellitus, type 2':ti,ab,kw OR 'diabetes, type 2':ti,ab,kw OR 'type 2 diabetes mellitus':ti,ab,kw OR 't2d':ti,ab,kw OR 'niddm':ti,ab,kw OR 'hyperglycemia':ti,ab,kw OR 'prediabetes':ti,ab,kw)
**5**	('ifg':ti,ab,kw OR 'impaired glucose':ti,ab,kw OR 'impaired fpg':ti,ab,kw OR 'glucose intolerance':ti,ab,kw OR 'igt':ti,ab,kw)
**6**	('major adverse cardiovascular events':ti,ab,kw OR 'myocardial infarction':ti,ab,kw OR 'heart failure':ti,ab,kw OR 'stroke':ti,ab,kw) OR ('chronic kidney disease':ti,ab,kw OR 'estimated glomerular filtration rate':ti,ab,kw OR 'albuminuria':ti,ab,kw) OR ('insulin secretion':ti,ab,kw OR 'insulin sensitivity':ti,ab,kw)
**7**	#1 OR #2 OR #3
**8**	#4 OR #5 OR #6
**9**	#7 AND #8
**10**	('review':it OR 'meta analysis':ti,ab,kw)
**11**	#9 NOT #10
**12**	(('controlled clinical trial'/exp OR 'clinical study'/exp OR 'randomization'/exp OR 'single blind procedure'/exp OR 'double blind procedure'/exp OR 'placebo'/exp) OR ('randomized controlled trial':ti,ab,kw OR 'singl*':ti,ab,kw OR 'doubl*':ti,ab,kw OR 'trebl*':ti,ab,kw OR 'tripl*':ti,ab,kw OR 'randomly':ti,ab,kw))
**13**	#11 AND #12

Supplementary 2: Characteristics of included studies

Table S2.1: Baseline of characteristics of included studies

Unique ID	Study ID	Trial registration	Design	Follow-up duration (weeks)	Number of participants	Randomize treatments	Dose and frequency	Diabetes duration (mean ± SD), years	Age (mean ± SD), years	Male , %	BMI (mean ± SD), kg/m²	HbA_{1c} (mean ± SD), %	Background hypoglycemic intervention
C1	BERNARD 2009	NCT00333151	RCT	26	533	Liraglutide 178<br>Liraglutide 178<br>Placebo 177	1.2mg QD sc<br>1.8mg QD sc	9±6	55±10	57<br>51<br>62	33.2±5.4<br>33.5±5.1<br>33.9±5.2	8.5±1.2<br>8.6±1.2<br>8.4±1.2	All subjects received metformin (1 g, twice daily) and rosiglitazone (4 mg, twice daily) as the basic hypoglycemic intervention.
B1	Bernard 2019	NCT03086330	RCT	35	302	Semaglutide 151<br>Placebo 151	1.0 mg QW sc	9.8±6.3<br>9.6±5.9	57.5±8.9<br>56.6±10.1	58.9<br>57.6	31.1±6.2<br>32.7±6.9	8.0±0.8<br>8.1±0.8	All patients received a stable dose of SGLT-2 inhibitor therapy (either as monotherapy or in combination with metformin ≥ 1500 mg/day or sulfonylureas) for at least 90 days. Among them, 71.5% of patients were on combination therapy with metformin, and 12.9% with sulfonylureas.

B2	Bernard Zinman 2019	NCT03021187	RCT	57	731	Semaglutide 184<br>Semaglutide 182<br>Semaglutide 181<br>Placebo 184	3mg QD po<br>7mg QD po<br>14mg QD po	15.1±7.9<br>16.2±8.6<br>14.1±8.0<br>14.8±7.9	61±10	55.4<br>56.6<br>47.0<br>57.1	31.0±6.8<br>31.1±7.0<br>30.8±6.3<br>31.0±6.5	8.2±0.7	All patients received a stable dose of insulin therapy (basal insulin, basal-bolus insulin, or premixed insulin; ≥ 10 U/day; ≥ 90 days), and 67.2% of them were on combination therapy with metformin (≥ 1500 mg/day or maximum tolerated dose; ≥ 90 days).
C19	L 2016	NCT02008682	RCT	26	368	Liraglutide 184<br>Sitagliptin 184	1.8 mg QD sc	5.3±4.4<br>5.2±5.4	51.7±10.7<br>51.4±11.0	55.7<br>63.6	27.3±3.4<br>27.2±4.0	8.14±0.83<br>8.11±0.78	All patients received a stable dose of metformin therapy (≥ 1500 mg/day or maximum tolerated dose ≥ 1000 mg/day) for at least 60 days.
F1	W Yang	NCT01632163	RCT	24	448	Lixisenatide 224<br>Placebo 224	20 ug QD sc	10.3 ± 6.1<br>10.2 ± 6.2	53.9 ± 9.9<br>56.2 ± 9.1	46.9<br>43.8	27.5 ± 4.39<br>27.9 ± 4.48	8.6 ± 0.84<br>8.6 ± 0.86	All patients received a stable dose of basal insulin (BI) (e.g., insulin glargine, NPH insulin, etc.), among whom 88.6% were on combination therapy with metformin (≥ 1.0 g/day).
C2	W 2010	Not provided	RCT	16	929	Liraglutide 231<br>Liraglutide 233<br>Liraglutide 233<br>Glimepiride 231	0.6 mg QD sc<br>1.2 mg QD sc<br>1.8 mg QD sc	7.5 ± 5.6	53.3 ± 9.5	55.2	25.6 ± 3.9	8.5 ± 1.1	All patients received a stable dose of metformin (mandatorily titrated up to 2000 mg/day, with permission to reduce to 1500 mg/day). During the trial, the use of insulin and other oral hypoglycemic agents was prohibited (except for the randomly assigned study drug).

B3	WANG 2024	NCT04109547	RCT	31	774	Semaglutide 130<br>Semaglutide 130<br>Semaglutide 130<br>Placebo 131	3mg QD po<br>7mg QD po<br>14mg QD po	2.3 ± 3.0<br>1.7 ± 2.4<br>2.1 ± 3.3<br>2.7 ± 3.8	52 ± 11	63.7	27.8 ± 4.8<br>28.4 ± 5.1<br>28.2 ± 5.8<br>28.4 ± 5.0	8.0 ± 0.8	Before enrollment and during the run-in period, only dietary control and exercise intervention were adopted, and no hypoglycemic agents of any kind were used.
B19	Yuichiro 2020	NCT03018028	RCT	57	243	Semaglutide 49<br>Semaglutide 49<br>Semaglutide 48<br>Placebo 49<br>Liraglutide 48	3mg QD po<br>7mg QD po<br>14mg QD po<br>0.9mg QD sc	7.4 ± 5.5<br>7.4 ± 5.6<br>7.9 ± 5.9<br>8.4 ± 6.0<br>6.7 ± 5.2	59 ± 9	79	26.5 ± 4.6<br>26.3 ± 3.5<br>24.7 ± 4.1<br>25.1 ± 3.9<br>26.9 ± 4.8	8.0 ± 0.8	no hypoglycemic agents of any kind were used.
B22	Daisuke 2020	NCT03015220	RCT	57	458	Semaglutide 131<br>Semaglutide 132<br>Semaglutide 130<br>Dulaglutide 65	3mg QD po<br>7mg QD po<br>14mg QD po<br>0.75mg QW sc	9.4 ± 6.3	58 ± 10	74	26.2 ± 4.8	8.3 ± 0.9	All participants were on oral antidiabetic monotherapy (sulfonylurea, glinide, thiazolidinedione, alpha-glucosidase inhibitor, or SGLT2 inhibitor). DPP-4 inhibitors were excluded.
E13	Carol	NCT01652716	RCT	28	377	Exenatide 229<br>Exenatid	2.0 mg QW sc	9 ± 6<br>8 ± 6	56 ± 10<br>57 ± 9	65<br>63	33 ± 6<br>33 ± 5	8.5 ± 1.0	Diet/exercise or stable oral medication (metformin, sulfonylureas, pioglitazone, or

						e BID 146	10ug bid sc						combination of two drugs): Non-sulfonylurea users: Metformin ± pioglitazone Sulfonylurea users: Sulfonylureas ± metformin/pioglitazone
D1	Weins tock 2015	NCT007 34474	RC T	104	1098	Dulagluti de 304 Dulagluti de 302 Sitaglipti n 315 Placebo 177	1.5 mg QW sc 0.75 mg QW sc	7	54	47. 4	31	8.1 ± 0.9	All patients were on a stable dose of metformin (≥ 1500 mg/day) at baseline, with some in combination with other oral hypoglycemic agents (e.g., sulfonylureas).
D9	Wang	NCT016 48582	RC T	52	774	Dulagluti de 258 Dulagluti de 257 Insulin Glargine 259	1.5 mg QW sc 0.75 mg QW sc	7.9 ± 4.8 8.1 ± 5.3	55.0 ± 9.6 54.5 ± 10.0	53. 4 56. 7	26.6 ± 3.7 27.0 ± 3.8	8.5 ± 1.2 8.3 ± 1.1	Metformin and/or sulfonylureas (stable dose for ≥ 3 months)
C20	Wang 2022	NCT031 72494	RC T	26	720	IDegLira 361 Deglude c 179 Liragluti de 180	10u+0. 36mg QD sc 10u QD sc 1.8 mg sc	8.00 ± 5.33 8.63 ± 5.55 8.05 ± 5.28	54.5 ± 10.3 55.7 ± 10.2 54.1 ± 10.2	60. 7 55. 9 60. 0	27.0 ± 3.9 26.5 ± 3.6 26.5 ± 3.6	8.20 ± 0.83 8.31 ± 0.84 8.21 ± 0.77	Before screening, all patients received a stable dose of metformin (1500 mg or maximum tolerated dose) ± one additional oral hypoglycemic agent (α-glucosidase inhibitors, sulfonylureas, glinides, or thiazolidinediones, with the dose being ≥ 50% of the locally

												approved maximum dose) for ≥ 60 consecutive days.
C3	Anna 2016	NCT01505673	RCT	6 个	71	Liraglutide 35<br>Placebo 36	1.8 mg QD sc	17.9 ± 8.4<br>17.9 ± 8.4	52.8 ± 8.1<br>55.5 ± 6.6	34<br>39	40.7 ± 6.7<br>41.6 ± 10.4	9.0 ± 1.2<br>8.9 ± 1.0	High-dose insulin (> 1.5 U/kg/day) plus maximum tolerated dose of metformin were stably used for ≥ 3 months at baseline.
D2	Guillermo 2014	NCT01126580	RCT	52	807	Dulaglutide 269<br>Dulaglutide 270<br>Metformin 268	1.5 mg QW sc<br>0.75mg QW sc	3 ± 2	56 ± 10<br>56 ± 11<br>55 ± 10	42<br>44<br>45	34 ± 6<br>33 ± 6<br>33 ± 5	7.6 ± 0.9<br>7.6 ± 0.9<br>7.6 ± 0.8	Before screening, patients were either managed solely through dietary and exercise control, or were on a single oral antidiabetic medication (OAM; e.g., metformin) with a dose ≤ 50% of the approved maximum dose, and had been using it stably for ≥ 3 months. All OAMs were discontinued 2 weeks prior to randomization.
C4	Miyagawa 2015	NCT01558271	RCR	26	587	Dulaglutide 280<br>Liraglutide 137<br>Placebo 70	0.75 mg QW sc<br>0.9 mg QD sc	6.8 ± 5.6<br>6.3 ± 6.0<br>6.3 ± 5.1	57.2 ± 9.6<br>57.9 ± 10.4<br>57.7 ± 8.3	81<br>83<br>79	25.6 ± 3.6<br>25.5 ± 3.5<br>25.2 ± 3.2	8.15 ± 0.77<br>8.08 ± 0.89<br>8.20 ± 0.83	Patients who were OAM-naive (managed solely through dietary and exercise control) before enrollment: Directly randomized after a 2-week run-in period. Patients who received monotherapy with OAMs (thiazolidinediones excluded) before enrollment: Randomized after an 8-week washout period.

												The use of other hypoglycemic agents was prohibited during the study. Rescue therapy was only permitted for severe, persistent hyperglycemia (fasting blood glucose meeting the target yet persistently elevated for ≥ 2 weeks without a clear cause).
B4	Christopher 2017	NCT02054897	RCT	30	387	Semaglutide 128<br>Semaglutide 130<br>Placebo 129	0.5 mg QW sc<br>1.0 mg QW sc	4.81 ± 6.10<br>3.62 ± 4.88<br>4.06 ± 5.48	54.6 ± 11.1<br>52.7 ± 11.9<br>53.9 ± 11.0	47<br>62<br>54	32.46 ± 7.62<br>33.92 ± 8.43<br>32.40 ± 6.86	8.09 ± 0.89<br>8.12 ± 0.81<br>7.95 ± 0.85	Before screening, patients were managed solely through dietary and exercise control for at least 30 days, with no hypoglycemic agents of any kind used (except for short-term insulin therapy lasting ≤ 7 days).
G1	Shuai M 2020	NCT02477865	RCT	24	361	PEX168 124<br>PEX168 116<br>Placebo 121	100 ug QW sc<br>200 ug QW sc	NA	50.5 ± 10.4<br>52.4 ± 11.5<br>51.5 ± 10.9	66.9<br>55.2<br>72.7	27 ± 3.7<br>26.4 ± 3.3<br>26.3 ± 3.4	8.5 ± 0.9<br>8.5 ± 0.9<br>8.6 ±1.0	Before screening, patients were managed solely through dietary control and exercise for at least 8 weeks, with no hypoglycemic agents of any kind used.
B23	Yutaka	NCT02254291	RCT	30	308	Semaglutide 103<br>Semaglutide 102<br>Sitagliptin 103	0.5 mg QW sc<br>1.0 mg QW sc	8.0 ± 5.2<br>7.8 ± 6.9<br>8.1 ± 6.7	58.8 ± 10.4<br>58.1 ± 11.6<br>57.9 ± 10.1	76.7<br>73.5<br>78.6	25.1 ± 3.8<br>26.1 ± 5.2<br>25.1 ± 3.6	8.2 ± 1.0<br>8.0 ± 0.9<br>8.2 ± 0.9	Before screening, patients had stably undergone dietary and exercise control or monotherapy with a single oral antidiabetic agent (e.g., biguanides, sulfonylureas, etc.). An 8-week washout period was required (except for biguanides), and only

												dietary/exercise control or monotherapy with a single oral antidiabetic agent was permitted prior to randomization.
C5	Seino 2010	NCT00393718	RCT	24	400	Liraglutide 268<br>Glibenclamide 132	0.9 mg QD sc	8.1 ± 6.7<br>8.5 ± 6.8	58.2 ± 10.4<br>58.5 ± 10.4	68<br>65	24.5 ± 3.7<br>24.4 ± 3.8	8.92 ± 1.08<br>8.78 ± 0.97	Before screening, patients had stably undergone dietary control or monotherapy with a single oral antidiabetic agent (e.g., biguanides, sulfonylureas, α-glucosidase inhibitors, etc.) for at least 8 weeks. A 4-6 week washout period was required prior to enrollment, with the dose of sulfonylureas required to be ≤ 50% of the maximum approved dose during this period.
C21	Yutaka 2022	NCT02505334	RCT	26	466	Liraglutide 233<br>Liraglutide 233	0.9 mg QD sc<br>1.8 mg QD sc	9.15 ± 5.40<br>9.40 ± 5.70	54.96 ± 10.61<br>55.07 ± 10.27	70.8<br>67	27.20 ± 4.72<br>27.34 ± 4.80	8.10 ± 0.87<br>8.14 ± 1.02	Before screening, patients had been on stable monotherapy with a single oral antidiabetic drug (OAD; e.g., biguanides, sulfonylureas, etc.) for at least 3 months. Prior to enrollment, OADs were discontinued for a washout period, followed by a 12-week run-in period during which only liraglutide 0.9 mg was administered (titrated up from 0.3 mg to 0.9 mg).

C6	Russell-Jones	NCT00331851	RCT	26	576	Liraglutide 230<br>Insulin Glargine 232<br>Placebo 114	1.8 mg QD sc	9.2 ± 5.8<br>9.7 ± 6.4<br>9.4 ± 6.2	57.6 ± 9.5<br>57.5 ± 10.5<br>57.5 ± 9.6	57<br>60<br>49	30.4 ± 5.3<br>30.3 ± 5.3<br>31.3 ± 5.0	8.3 ± 0.9<br>8.2 ± 0.9<br>8.3 ± 0.9	Before screening, patients had been on stable combination therapy with metformin (1 g twice daily) and glimepiride (4 mg once daily) for at least 3 months, with HbA_1c ranging from 7.0% to 10.0% (on combination therapy) or 7.5% to 10.0% (on monotherapy).
E1	DAVID 2011	NCT00676338	RCT	26	820	Exenatide 248<br>Metformin 246<br>Pioglitazone 163<br>Sitagliptin 163	2.0 mg QW sc	2.7	54	59	NA	8.5 ± 0.9<br>8.4 ± 0.9<br>8.6 ± 0.9<br>8.5 ± 0.9	Before screening, patients were drug-naive (managed solely through dietary and exercise control) with no hypoglycemic agents of any kind used.
A1	Rosenstock 2021	NCT03954834	RCT	40	478	Tirzepatide 121<br>Tirzepatide 121<br>Tirzepatide 121<br>Placebo 115	5 mg QW sc<br>10mg QW sc<br>15 mg QW sc	4.6 ± 5.1<br>4.9 ± 5.6<br>4.8 ± 5.0<br>4.5 ± 5.9	54.1 ± 11.9<br>55.8 ± 10.4<br>52.9 ± 12.3<br>53.6 ± 12.8	46<br>60<br>52<br>49	32.2 ± 7.0<br>32.2 ± 7.6<br>31.5 ± 5.5<br>31.7 ± 6.1	7.97 ± 0.84<br>7.90 ± 0.78<br>7.85 ± 1.02<br>8.05 ± 0.80	Before screening, patients were either managed solely through dietary and exercise control, or had been on stable oral antidiabetic agents (e.g., metformin) for at least 3 months, with no injectable hypoglycemic agents used.

H1	Rosenstock 2019	NCT02057172	RCT	12	252	Efpeglenatide 37<br>Efpeglenatide 37<br>Efpeglenatide 33<br>Efpeglenatide36<br>Efpeglenatide 36<br>Liraglutide 36<br>Placebo 37	0.3 mg QW sc<br>1.0 mg QW sc<br>2.0 mg QW sc<br>3.0 mg QW sc<br>4.0 mg QW sc<br>1.8 mg QD sc	5.9±5.4<br>6.4±4.8<br>6.3±5.1	55.6±10.0<br>54±11<br>55.6±9.0	53<br>44<br>43	32±5<br>32±4<br>31±5	7.8±0.8<br>8.0±0.8<br>7.6±0.9	Eighty-nine percent of patients were on metformin (≥ 1500 mg/day) at baseline, while 11% were drug-naive (managed through dietary and exercise control).
F7	JULIO 2013	NCT00707031	RCT	24	634	Lixisenatide 318<br>Exenatide 316	20 ug QD sc<br>10 ug BID sc	6.8 ± 5.5<br>6.8 ± 4.9	57.3 ± 9.2<br>57.6 ± 10.7	47.5<br>59.2	33.7 ± 6.3<br>33.5 ± 6.5	8.03 ± 0.8<br>8.02 ± 0.8	Before screening, patients had been on a stable dose of metformin ≥ 1.5 g/day for at least 3 months, with no other hypoglycemic agents used (e.g., insulin, GLP-1 receptor agonists, etc.).
B5	Julio 2023	NCT02607865	RCT	78	1864	Semaglutide 466<br>Semaglutide 466<br>Semaglutide 465<br>Sitagliptin 467	3 mg QD po<br>7 mg QD po<br>14 mg QD po	8.4 ± 6.1<br>8.3 ± 5.8<br>8.7 ± 6.1<br>8.8 ± 6.0	58 ± 10.0<br>58 ± 10.0<br>57 ± 10.0<br>58 ± 10.0	54.5<br>52.7<br>53.1<br>51.0	32.6 ± 6.7<br>32.6 ± 6.4<br>32.3 ± 6.3<br>32.5 ± 6.2	8.3 ± 1.0<br>8.4 ± 1.0<br>8.3 ± 0.9<br>8.3 ± 0.9	Patients had been on a stable dose of metformin (≥ 1500 mg/day), which could be combined with sulfonylureas (e.g., glimepiride, gliclazide, etc.). The medications had been used stably for at least 3 months before screening.

B24	Helen a	NCT028 63328	RC T	52	822	Semaglut ide 412 Empaglif lozin 410	14 mg QD po	7.4 ± 6.1 7.7 ± 6.3	58	50. 1 51	32.8 ± 6.1 32.8 ± 5.9	8.1 ± 0.9 8.1 ± 0.9	Before screening, patients had been on a stable dose of metformin (≥ 1500 mg/day or maximum tolerated dose) for at least 3 months, with no other hypoglycemic agents used except for short-term insulin therapy lasting ≤ 14 days.
B6	Helen a 2018	NCT023 05381	RC T	30	396	Semaglut ide 132 Semaglut ide 131 Placebo 133	0.5 mg QW sc 1.0 mg QW sc	12.9 13.7 13.3	59.1 58.5 58.8	56. 1 58. 8 53. 4	32.8 32.0 31.8	8.4 ± 0.83 8.3 ± 0.71 8.4 ± 0.9	Before screening, patients had been on stable treatment with basal insulin (≥ 0.25 IU/kg/day or ≥ 20 IU/day for insulin glargine, insulin detemir, etc.) as monotherapy or in combination with metformin for at least 90 days, with HbA_{1c} ranging from 7.0% to 10.0%.
F9	Micha el 2016	NCT019 73231	RC T	26	404	Liragluti de 202 Lixisenat ide 202	1.8 mg QD sc 20 ug QD sc	6.5 ± 5.3 6.3 ± 5.0	56.3 ± 10.6 56.1 ± 10.0	65 55	34.5 ± 6.8 34.9 ± 6.6	8.4 ± 0.7 8.4 ± 0.8	All patients had been on stable treatment with metformin at the maximum tolerated dose (1000-3000 mg/day) for at least 90 days prior to enrollment.
F3	MAT THE W 2013	NCT007 15624	RC T	24	495	Lixisenat ide 328 Placebo 167	20 ug QD sc	12.5 ± 7.0 12.4 ± 6.3	57 ± 10	46	31.9 ± 6.2 32.6 ± 6.3	8.4 ± 0.8	All patients had been on stable basal insulin therapy (e.g., insulin glargine, insulin detemir, NPH insulin, etc.) for at least 3 months prior to enrollment, with a stable dose (±20%) and a daily dose ≥ 30 units; 79% of the patients were

													concurrently receiving metformin treatment (≥ 1.5 g/day, or ≥ 1.0 g/day for patients in South Korea), which had lasted for at least 3 months.
B25	Richard 2018	NCT02648204	RCT	40	1199	Semaglutide 301<br>Semaglutide 300<br>Dulaglutide 299<br>Dulaglutide 299	0.5 mg QW sc<br>1.0 mg QW sc<br>0.75 mg QW sc<br>1.5 mg QW sc	7.7 ± 5.9<br>7.3 ± 5.7<br>7.0 ± 5.5<br>7.6 ± 5.6	56 ± 10.9<br>55 ± 10.6<br>55 ± 10.4<br>56 ± 10.6	56<br>54<br>54<br>57	NA	NA	NA
B20	Richard Pratley, 2019	NCT02863419	RCT	52	711	Semaglutide 285<br>Liraglutide 284<br>placebo 142	14 mg QD po<br>1.8 mg QD sc	7.8 ± 5.7<br>7.3 ± 5.3<br>7.8 ± 5.5	56 ± 10<br>56 ± 10<br>57 ± 10	52	32.5 ± 5.9<br>33.4 ± 6.7<br>32.9 ± 6.1	8.0 ± 0.7<br>8.0 ± 0.7<br>7.9± 0.7	Prior to the trial, patients had been on a stable dose of metformin (≥ 1500 mg/day or maximum tolerated dose) for at least 90 days, which could be combined with an SGLT2 inhibitor. The use of other hypoglycemic agents (e.g., sulfonylureas, insulin, etc.) was prohibited.
D3	Paolo Pozzilli, 2016	NCT02152371	RCT	28	300	Dulaglutide 150<br>placebo 150	1.5 mg QW sc	13.0 ± 7.5<br>13.3 ± 7.7	60.2 ± 9.5<br>60.6 ± 10.1	56.7<br>58.7	32.8 ± 4.9<br>32.6 ± 4.9	8.4 ± 0.9<br>8.3 ± 0.8	Insulin glargine was titrated daily (basal dose: 39 ± 22 U), and could be combined with metformin (≥ 1500 mg/day). Prior to the trial, patients had been on a stable dose

												of insulin glargine (with or without concurrent metformin) for at least 3 months, with no other GLP-1 receptor agonists or insulin secretagogues used.
C7	A. Ahmann, 2015	NCT01617434	RCT	26	451	Liraglutide 226<br>placebo 225	1.8 mg QD sc	12.1 ± 7.1<br>12.1 ± 6.8	NA	53.3<br>60.4	32.3 ± 5.6<br>32.3 ± 5.7	8.2 ± 0.8<br>8.3 ± 0.9	Patients had been on a stable dose of basal insulin analogs (insulin glargine or insulin detemir, ≥ 20 U/day) with or without metformin (≥ 1500 mg/day) for at least 8 weeks prior to the trial. Specifically, they had maintained a stable dose of basal insulin (insulin glargine or insulin detemir) for no less than 8 weeks before the trial; concurrent use of metformin was permitted, while the use of other GLP-1 receptor agonists, insulin secretagogues, or intensive insulin therapy was prohibited.
C22	S.T. Azar, 2016	NCT01917656	RCT	33	343	Liraglutide 172<br>Sulfonylureas 171	1.8 mg QD sc	8.0 ± 5.26<br>7.2 ± 4.39	54.9 ± 9.27<br>54.0 ± 9.33	49.7<br>48.8	30.2 ± 5.37<br>31.4 ± 5.88	8.3 ± 0.94<br>8.2 ± 0.91	Stable doses of metformin plus a sulfonylurea (for ≥ 90 days prior to the trial). The use of insulin was prohibited before the trial (except for short-term use ≤ 7 days due to acute conditions), and other hypoglycemic agents were also forbidden.

C23	Francisco J, 2021	NCT01919489	RCT	26	277	Liraglutide 140<br>Insulin glargine 137	1.8 mg QD sc	9.5 ± 7.8<br>9.8 ± 9.1	56.1 ± 9.5<br>55.9 ± 11.2	65<br>55	33.5 ± 5.3<br>33.3 ± 5.3	8.3 ± 0.9<br>8.4 ± 0.8	Continuation of oral antidiabetic agents used prior to the trial (e.g., metformin, sulfonylureas) was permitted, but dose adjustments were required. Exclusion criteria included the use of GLP-1 receptor agonists within the recent 3 months, type 1 diabetes mellitus, severe hepatic or renal insufficiency, etc.
F4	Chang Yu Pan, 2014	NCT01169779	RCT	24	391	Lixisenatide 196<br>placebo 195	20 ug QD sc	6.5 ± 4.6<br>6.8 ± 4.8	54.5 ± 10.3<br>55.1 ± 10.5	51.5<br>46.9	26.8 ± 3.9<br>27.1 ± 3.8	7.95 ± 0.81<br>7.85 ± 0.71	Stable doses of metformin (1.0–1.5 g/day) with or without a sulfonylurea, which had been used stably for 3 months prior to the trial.
D4	Tomoaki, 2024	NCT04809220	RCT	52	591	Dulaglutide 395<br>Dulaglutide 196	1.5 mg QW sc<br>0.75 mg QW sc	10.7±6.6<br>10.4±6.7	60.1±10.7<br>60.7±10.7	71.7	26.6±3.9<br>26.1±3.7	8.6±0.6	All patients were on a stable dose of one oral antidiabetic medication (OAM; e.g., sulfonylureas, metformin, α-glucosidase inhibitors, etc.) prior to randomization, and this medication was continued until the end of the study (dipeptidyl peptidase-4 [DPP-4] inhibitors needed to be discontinued).
F5	Graydon S, 2017	NCT01798706	RCT	24	350	Lixisenatide 176<br>placebo 174	20 mg QD sc	13.6±7.3<br>14.6±7.9	74.0±4.0<br>74.4±3.8	52	29.9±3.7<br>30.1±4.5	8.1±0.7<br>8.1±0.7	Concurrent use of metformin, sulfonylureas (e.g., glimepiride ≤ 10 mg, gliclazide ≤ 160 mg), meglitinides (e.g., repaglinide ≤ 6

												mg), pioglitazone, or basal insulin was permitted.
C8	Melanie J, 2015	NCT01620489	RCT	26	279	Liraglutide 140<br>placebo 139	1.8 mg QD sc	15.9±8.9<br>14.2±7.5	68.0±8.3<br>66.3±8.0	53.6<br>47.4	33.4±5.4<br>34.5±5.4	8.08±0.79<br>8.00±0.85	Concurrent use of metformin, sulfonylureas (SU), pioglitazone, basal insulin, or premixed insulin was permitted. At baseline, 55% of patients were using insulin (19.1% on basal insulin and 36.1% on premixed insulin), and 45% were using oral antidiabetic drugs (OADs) (either as monotherapy or in combination with metformin, SU, or pioglitazone).
C9	M. Marre, 2009	NA	RCT	26	1041	Liraglutide 233<br>Liraglutide 228<br>Liraglutide 234<br>placebo 114<br>Rosiglitazone 232	0.6 mg QD sc<br>1.2 mg QD sc<br>1.8 mg QD sc	6.5	55.7–57.7 ± 9.0–10.0	45–54	29.4–30.3 ± 4.8–5.4	8.4–8.5 ± 0.9–1.1	All patients had received oral antidiabetic drugs (either as monotherapy or combination therapy) prior to enrollment. During the study, all patients were uniformly switched to glimepiride monotherapy (2–4 mg/day), and concurrent use of other hypoglycemic agents (e.g., metformin) was prohibited.)

E2	Chieh-Hsiang, 2013	NCT00324363	RCT	16	51	Exenatide 26 placebo 25	10 mg BID sc	6.7±3.9 8.5±5.7	50.5±9.0 51.3±9.9	54	27.2±3.3	8.1±1.0 8.1±1.0	Metformin monotherapy: 2 cases (7.7%) in the exenatide group and 3 cases (12.5%) in the placebo group. Metformin plus sulfonylurea: 24 cases (92.3%) in the exenatide group and 21 cases (87.5%) in the placebo group. Concurrent use of other hypoglycemic agents (e.g., thiazolidinediones, α-glucosidase inhibitors, etc.) was prohibited during the study.
B7	Ildiko Lingvay, 2018	NCT02461589	RCT	26	512	Semaglutide 0.05 mg（64）、0.1 mg（63）、0.2 mg（65）、0.3 mg（63）; Liraglutide 0.3 mg（64）、0.6 mg		7.2±5.6	56.7±9.9	53.8	32.8±4.4	8.1±0.8	Metformin: 61.3% of patients were on metformin at baseline (≥ 1500 mg/day or maximum tolerated dose), and the dose was maintained stable during the study.

						(64)、1.2 mg (64)、1.8 mg (65); placebo 129							
B8	Ildiko Lingvay, 2019	NCT03136484	RCT	52	788	Semaglutide 394 Canagliflozin 394	1.0 mg QW sc	7.5 ± 5.9 7.2 ± 5.4	55.7 ± 11.1 57.5 ± 10.7	57 51	32.2 ± 6.8 32.5 ± 6.9	8.3 ± 1.0 8.2 ± 1.0	All patients were on a stable dose of metformin (≥ 1500 mg/day or maximum tolerated dose) for at least 90 consecutive days prior to screening. No other hypoglycemic agents except metformin were permitted during the trial (with the exception of emergency medications).
C10	Marcus Lind, 2015	EudraCT 2012-001941-42	RCT	24	129	Liraglutide 64 placebo 60	1.8 mg QD sc	17.3 ± 7.6 17.0 ± 8.1	63.7 ± 8.2 63.5 ± 7.7	62.5 66.7	33.7 ± 4.3 33.5 ± 4.0	9.0 ± 1.0 9.0 ± 1.1	All patients received multiple daily insulin injections (including basal insulin and prandial insulin, with at least 2 doses of prandial insulin per day) and had been using this regimen stably for at least 8 weeks prior to screening. Concurrent use of metformin was permitted (68.8% of patients in the liraglutide group and 73.3% in

												the placebo group were on metformin at baseline).
B21	Kimura, 2023	UMIN000044264	RCT	24	120	Dulaglutide 59<br>Semaglutide 61	0.75 mg QW sc<br>1.0 mg QW sc	13.2 ± 7.0<br>14.6 ± 7.8	62.7 ± 11.4<br>62.7 ± 10.1	55<br>56	29.2 ± 5.9<br>29.4 ± 5.9	8.1 ± 0.6<br>7.9 ± 0.5	All patients were on a stable dose of oral antidiabetic drugs (OADs) (e.g., metformin, sulfonylureas, etc.) at baseline. Continuation of these medications was permitted during the trial, but adjustments to the doses of other hypoglycemic agents or introduction of new hypoglycemic agents (other than the study drug) were prohibited. No adjustments to insulin or other non-GLP-1 receptor agonist (non-GLP-1 RA) medications were allowed during the study.
E3	David M, 2005	NA	RCT	30	733	Exenatide 245<br>Exenatide 241<br>placebo 247	5 ug BID sc<br>10 ug BID sc	9.4 ± 6.2	55 ± 10	59	33.6 ± 5.7	8.5 ± 1.0	All patients were on a stable combination therapy of metformin (1500 mg/day) plus a sulfonylurea (at the maximum effective dose or the minimum recommended dose), which had been maintained for at least 3 months at baseline. During the trial, adjustments to the doses of metformin and sulfonylurea were prohibited unless hypoglycemia

												occurred (in which case the sulfonylurea dose needed to be reduced).
B26	Kohei Kaku , 2017	NCT02207374	RCT	56	601	Semaglutide 239<br>Semaglutide 241<br>OAD 121	0.5 mg QW sc<br>1.0 mg QW sc	8.1 ± 6.0<br>9.4 ± 6.5<br>9.3 ± 7.0	58.0 ± 10.6<br>58.7 ± 10.2<br>59.2 ± 10.1	69.5<br>72.2<br>74.2	26.2 ± 4.8<br>26.4 ± 4.7<br>26.7 ± 4.6	8.0 ± 0.9<br>8.1 ± 1.0<br>8.1 ± 0.9	Semaglutide group: It could be used as monotherapy or in combination with baseline oral antidiabetic drugs (OADs) (e.g., metformin, sulfonylureas, etc.), but adjustments to the doses of other OADs were prohibited. Additional OAD group: An oral antidiabetic drug with a different mechanism of action (e.g., dipeptidyl peptidase-4 [DPP-4] inhibitors, biguanides, etc.) was added to the baseline treatment.
C11	Kohei Kaku , 2010	NA	RCT	24	264	Liraglutide 88<br>Liraglutide 88<br>placebo 88	0.6 mg QD sc<br>0.9 mg QD sc	9.3 ± 5.8<br>11.6 ± 7.7<br>10.1 ± 7.3	59.1 ± 10.3<br>61.3 ± 11.0<br>58.6 ± 9.7	60<br>67<br>65	25.3 ± 3.6<br>24.4 ± 3.4<br>24.9 ± 4.0	8.60 ± 0.91<br>8.23 ± 0.78<br>8.45 ± 0.99	All patients were on sulfonylurea monotherapy (glimepiride, gliclazide, or glimepiride) for at least 8 weeks at baseline, with $HbA_{1}c$ between 7.0% [and the upper limit should be supplemented as per the original text, e.g., "and 10.0%"].
A5	Takashi , 2022	NCT03861039	RCT	52	443	Tirzepatide 148<br>Tirzepatide 147	5 mg QW sc<br>10 mg QW sc	8.6	57.0 ± 10.8	76	27.9 ± 4.8	8.6 ± 1.1	All patients were receiving monotherapy with a single oral antidiabetic drug (OAD) for at least 3 months at baseline, with

						Tirzepatide 148	15 mg QW sc						the dose maintained stable for no less than 8 weeks. During the trial: the dose of the baseline OAD was kept unchanged, and concurrent use of other hypoglycemic agents was prohibited (except in emergency situations).
E4	Michael Joubert 2021	NCT01140893	RCT	24	46	Exenatide 28 placebo 18	10 ug BID sc	18.4 ± 7.7 19.3 ± 7.9	58 ± 8 61 ± 7	50 39	35.1 ± 4.4 35.5 ± 4.2	8.9 ± 0.9 8.8 ± 0.8	Continuation of insulin pump therapy or multiple daily insulin injections (MDI) was permitted. Adjustments to the total daily insulin dose were prohibited unless hypoglycemia occurred (in which case the dose needed to be reduced); however, adjustments to the insulin infusion rate were allowed based on the blood glucose target ("treat-to-target" strategy).
B9	Bo Ahrén 2017	NCT01930188	RCT	56	1225	Semaglutide 409 Semaglutide 409 Sitagliptin 407	0.5 mg QW sc 1.0 mg QW sc	6.4 ± 4.7 6.7 ± 5.6 6.6 ± 5.1	56.2 ± 10.3	51	32.4 ± 6.2 32.5 ± 6.6 32.5 ± 5.8	8.0 ± 0.9 8.0 ± 0.9 8.2 ± 0.9	Metformin (≥ 1500 mg/day), thiazolidinediones (e.g., pioglitazone ≥ 30 mg/day or rosiglitazone ≥ 4 mg/day), or their combination. These medications had been used stably at the maximum tolerated dose for at least 90 days prior to screening.

E14	Linong Ji 2013	NCT00917267	RCT	26	678	EQW 340<br>EBID 338	2 mg QW sc<br>10 ug BID sc	7.7 ± 5.1<br>8.6 ± 6.0	56 ± 10<br>55 ± 11	54.4<br>53.8	26.4 ± 3.7<br>26.7 ± 3.4	8.7 ± 1.0<br>8.7 ± 1.0	Oral antidiabetic medications (OAMs), including metformin (MET), sulfonylureas (SU), thiazolidinediones (TZD), or their combinations. These medications had been used stably for at least 3 months prior to screening, and the dose of sulfonylureas (SU) was adjusted to the minimum recommended dose in respective countries during the study.
B10	Linong Ji 2024	NCT04017832	RCT	26	1441	Semaglutide 361<br>Semaglutide 360<br>Semaglutide 361<br>Sitagliptin 359	3 mg QD po<br>7 mg QD po<br>14 mg QD po	5.8±5.4<br>5.2±4.9<br>5.9±5.2<br>5.6±4.9	53	58.3	28.9±6.0<br>28.8±5.6<br>28.4±5.0<br>28.2±5.3	8.1±0.9	All patients were on a stable dose of metformin (≥ 1500 mg/day or the maximum tolerated dose), which had remained unchanged for at least 60 days prior to screening. No other hypoglycemic agents were used (except for short-term insulin use ≤ 14 days).
E5	Serge A. Jabbour 2018	NCT02229396	RCT	52	695	EQW+Dapagliflozin 231<br>EQW+Placebo 230<br>Dapagliflozin+Placebo 233	2 mg QW sc	7.4	54.2	47.9	32.7	9.3 ± 1.0	All patients were on stable monotherapy with metformin (≥ 1500 mg/day), which had remained unchanged for at least 3 months prior to screening.

A2	Nobuya Inagaki 2022	NCT03861052	RCT	52	636	Tirzepatide 159<br>Tirzepatide 158<br>Tirzepatide 160<br>Dulaglutide 159	5 mg QW sc<br>10 mg QW sc<br>15 mg QW sc<br>0.75 mg QW sc	4.8	56.6 ± 10.3	76	28.1 ± 4.4	8.2 ± 0.9	Treatment-naive patients: Managed solely through diet and exercise (baseline $HbA_{1}c$: 7.0%–10.0%).<br>Patients on oral antidiabetic drug (OAD) monotherapy: Received monotherapy with sulfonylureas, biguanides, or thiazolidinediones prior to screening (an 8-week washout period was required; baseline $HbA_{1}c$: 6.5%–9.0%).
B11	Juan P Frías 2021	NCT03989232	RCT	40	961	Semaglutide 480<br>Semaglutide 481	2.0 mg QW sc<br>1.0 mg QW sc	9.5 ± 6.2	58.0 ± 10.0	59	34.6 ± 7.0	8.9 ± 0.6	All patients: On a stable dose of metformin (≥ 1500 mg/day or the maximum tolerated dose), which could be combined with sulfonylureas (≤ 50% of the maximum dose).<br>Prohibited medications: Insulin (except for short-term use ≤ 14 days for acute illnesses), GLP-1 receptor agonists, DPP-4 inhibitors, SGLT2 inhibitors, etc.
D11	E. Araki 2015	NCT01584232	RCT	26		Dulaglutide 181<br>Glargine 180	0.75 mg QW sc	8.9 ± 6.7<br>8.8 ± 6.1	57.5 ± 10.5<br>56.1 ± 11.3	69<br>74	26.1 ± 3.6<br>25.9 ± 3.9	8.1 ± 0.8<br>8.0 ± 0.9	The baseline doses of sulfonylureas and/or biguanides were maintained, and the addition of other hypoglycemic agents (e.g., α-glucosidase inhibitors, thiazolidinediones, etc.) was prohibited.

B12	Vanita R Aroda 2023	NCT04707469	RCT	68	1596	Semaglutide 526<br>Semaglutide 535<br>Semaglutide 535	14 mg QD po<br>25 mg QD po<br>50 mg QD po	9.4 ± 5.9<br>9.7 ± 6.7<br>8.9 ± 5.9	58.4 ± 10.4<br>58.8 ± 10.7<br>57.6 ± 11.2	61<br>57<br>57	33.7 ± 6.1<br>34.1 ± 6.5<br>33.7 ± 6.2	8.9 ± 0.8<br>9.0 ± 0.8<br>8.9 ± 0.7	Dipeptidyl peptidase-4 (DPP-4) inhibitors were required to be discontinued at randomization. The dose of sulfonylureas needed to be halved to reduce the risk of hypoglycemia (at the investigator's discretion). The addition of glucagon-like peptide-1 (GLP-1) receptor agonists, DPP-4 inhibitors, or amylin analogs was prohibited; however, dose adjustments of other background hypoglycemic agents were permitted as needed.
B13	Vanita R. Aroda 2019	NCT02906930	RCT	26	703	Semaglutide 175<br>Semaglutide 175<br>Semaglutide 175<br>placebo 178	3 mg QD po<br>7 mg QD po<br>14 mg QD po	3.5 ± 4.9	55 ± 11	50.8	31.8 ± 6.6	8.0 ± 0.7	Managed solely through diet and exercise, with no use of any hypoglycemic agents (except for short-term insulin therapy ≤ 14 days; no hypoglycemic agents were received within 90 days prior to screening).
E6	Richard M. Bergenstal 2010	NCT00637273	RCT	26	514	Exenatide 170<br>Sitagliptin 172<br>Pioglitazone 172	2 mg QW sc	6 ± 5	52 ± 10	47	32 ± 5	8.5 ± 1.1	Only the study drug was permitted; the addition of any other hypoglycemic agents was prohibited.

E15	Thomas Blevins 2011	NA	RCT	24	252	Exenatide 129<br>Exenatide 123	2 mg QW sc<br>10 ug BID sc	7 ± 5	56 ± 11<br>55 ± 10	60<br>55	33.6 ± 5.5<br>33.0 ± 5.3	8.5 ± 1.1<br>8.4 ± 1.2	The doses of background hypoglycemic agents were maintained unchanged, and the use of other hypoglycemic agents (e.g., insulin, GLP-1 receptor agonists, etc.) was prohibited. Dose adjustments of sulfonylureas (SU) or thiazolidinediones (TZD) were permitted based on blood glucose levels, but such adjustments must be documented.
D10	Lawrence Blonde 2015	NCT01191268	RCT	52	884	Dulaglutide 295<br>Dulaglutide 293<br>Insulin glargine 296	1.5 mg QW sc<br>0.75 mg QW sc	12.8 ± 7.0<br>12.4 ± 6.9<br>13.0 ± 6.8	58.9 ± 9.6<br>59.3 ± 9.0<br>59.9 ± 9.1	53.7	32.0 ± 5.1<br>33.1 ± 5.2<br>32.4 ± 5.3	8.46 ± 1.08<br>8.40 ± 1.03<br>8.53 ± 1.03	All enrolled patients had received conventional insulin therapy (1–2 times per day, including basal insulin, basal + prandial insulin, or premixed insulin) and could be on combination therapy with oral antidiabetic drugs (e.g., metformin; during the trial, the metformin dose was required to be ≥ 1500 mg/day and stable for at least 6 weeks). During the trial, all other oral antidiabetic drugs were discontinued, with only metformin retained.

E7	John B. Buse 2011	NCT00765817	RCT	30	261	Exenatide 138<br>placebo 123	10 ug BID sc	12 ± 7	59 ± 9<br>59 ± 10	51<br>64	33.8 ± 5.8<br>33.1 ± 6.2	8.32 ± 0.85<br>8.50 ± 0.96	All patients had been on a stable dose of insulin glargine (≥ 20 U/day) prior to enrollment, which could be combined with metformin and/or pioglitazone (with stable doses maintained for at least 3 months). During the trial, the original oral medications were kept unchanged, and the dose of insulin glargine was adjusted based on the fasting blood glucose target (< 5.6 mmol/L).
E16	John B. Buse 2012	NCT01029886	RCT	26	912	Liraglutide 450<br>Exenatide 461	1.8 mg QD sc<br>2.0 mg QW sc	9 ± 6<br>8 ± 6	57 ± 9.6<br>57 ± 9.4	54<br>55	32.3 ± 5.5	8.4 ± 1.0<br>8.5 ± 1.0	All patients had received lifestyle interventions and oral antidiabetic drugs (OADs) (metformin, sulfonylureas, metformin + sulfonylureas, or metformin + pioglitazone) prior to enrollment, with stable doses maintained for at least 3 months. During the trial, continuation of the original oral medications was permitted, and the dose of sulfonylureas could be adjusted based on blood glucose levels.

A3	Dominik Dahl 2022	NCT04039503	RCT	40	475	Tirzepatide 116<br>Tirzepatide 119<br>Tirzepatide 120<br>placebo 120	5 mg QW sc<br>10 mg QW sc<br>15 mg QW sc	13.3	60.6 ± 9.9	56	33.4 ± 6.1	8.31 ± 0.85	All patients had been on a stable dose of insulin glargine (once daily, > 20 IU/day or > 0.25 IU/kg/day) for at least 8 weeks prior to enrollment, with metformin (≥ 1500 mg/day) permitted for combination use.
E12	Melanie Davies 2013	NCT01003184	RCT	26	222	Exenatide 111<br>Insulin Detemir 105	2.0 mg QW sc	8±6<br>7±5	59±10<br>58±10	64<br>69	33.7±4.7	8.37±0.85<br>8.35±0.88	All patients had been on stable monotherapy with metformin (≥ 1000 mg/day) or combination therapy with metformin plus sulfonylureas (SU) for at least 3 months prior to enrollment. During the trial, the metformin dose was maintained unchanged; the SU dose was halved at randomization and could be further adjusted based on blood glucose levels thereafter. The combination of other hypoglycemic agents (e.g., GLP-1 receptor agonists, DPP-4 inhibitors, etc.) was prohibited.
C13	Melanie J. Davies 2015	NCT01272232	RCT	56	846	Liraglutide 423<br>Liraglutide 211	3.0 mg QD sc<br>1.8 mg QD sc	7.5 ± 5.65<br>7.4 ± 5.16	55.0 ± 10.8<br>54.9 ± 10.7	52.0<br>51.2	37.1 ± 6.5<br>37.0 ± 6.9	7.9 ± 0.8<br>8.0 ± 0.8	All patients had been on a stable regimen of 0–3 oral antidiabetic drugs (OADs) (metformin, thiazolidinediones, sulfonylureas) prior to enrollment, with diet and

						placebo 212		6.7 ± 5.07	54.7 ± 9.8	45.8	37.4 ± 7.1	7.9 ± 0.8	exercise management permitted as adjunctive therapy. During the trial, the doses of the original oral medications were maintained; the dose of sulfonylureas could be halved to reduce the risk of hypoglycemia. The initiation of new hypoglycemic agents (e.g., insulin, GLP-1 receptor agonists, etc.) was prohibited.
H2	Stefano Del Prato 2020	NCT02081118	RCT	16	209	efpeglenatide 52<br>efpeglenatide 52<br>efpeglenatide 53<br>placebo 52	8.0 mg qm sc<br>12 mg qm sc<br>14 mg qm sc	7.9 ± 6.2<br>7.2 ± 5.3	56.4 ± 9.0<br>54.7 ± 9.9	45.9<br>46.0	32.0 ± 4.5<br>32.4 ± 4.2	7.81 ± 0.75<br>7.90 ± 0.68	All patients had been on a stable dose of metformin (≥ 1500 mg/day or the maximum tolerated dose) for at least 3 months prior to enrollment. During the trial, the metformin dose was maintained unchanged, and the combination of other hypoglycemic agents (e.g., insulin, GLP-1 receptor agonists, DPP-4 inhibitors, etc.) was prohibited. Dose adjustments of lipid-lowering drugs and antihypertensive drugs were permitted as clinically indicated.
E17	Daniel J Drucker 2008	NCT00308139	RCT	30	303	Exenatide 148<br>Exenatide 147	2.0 mg QW sc<br>10ug bid sc	6.7 ± 5.0	55 ± 10	53	35 ± 5	8.3 ± 1.0	All patients had received diet and exercise management or oral antidiabetic drugs (OADs) (monotherapy with metformin, sulfonylureas, thiazolidinediones,

												or their combinations) for at least 2 months prior to enrollment. During the trial, the original oral medication regimen was maintained (the sulfonylurea dose was required to be reduced to the minimum within the first 10 weeks, and could be adjusted thereafter). The combination of insulin, GLP-1 receptor agonists, DPP-4 inhibitors, α-glucosidase inhibitors, etc., was prohibited.
D12	Kathleen M Dungan 2014	NCT01624259	RCT	26	599	Dulaglutide 299<br>Liraglutide 300	1.5 mg QW sc<br>1.8 mg QD sc	7.1 ± 5.4<br>7.3 ± 5.4	56.5 ± 9.3<br>56.8 ± 9.9	46<br>50	33.5 ± 5.1<br>33.6 ± 5.2	8.1 ± 0.8	All patients had been on stable monotherapy with metformin (≥ 1500 mg/day) for at least 3 months prior to enrollment. During the trial, adjustments to other oral antidiabetic drugs (e.g., sulfonylureas, thiazolidinediones) were prohibited. Rescue hypoglycemic agents (e.g., insulin) could only be initiated in cases of severe hyperglycemia, but such initiation must be documented and the relevant data excluded.

H3	Juan Pablo Frias 2022	NCT03353350	RCT	56	406	Efpeglenatide 100<br>Efpeglenatide 101<br>Efpeglenatide 103<br>placebo 102	2 mg QW sc<br>4 mg QW sc<br>6 mg QW sc	5.3 ± 5.3<br>4.9 ± 5.0<br>5.2 ± 5.2<br>5.0 ± 4.9	58.6 ± 10.5<br>56.3 ± 11.5<br>59.6 ± 10.7<br>59.5 ± 11.7	54.2	34.4 ± 6.4<br>33.8 ± 6.6<br>33.8 ± 6.9<br>34.8 ± 7.1	8.1 ± 0.9	Managed solely through diet and exercise; the use of any hypoglycemic agents was prohibited during the trial.
D7	Juan P. Frias 2019	NA	RCT	18	318	Dulaglutide 81<br>Dulaglutide 79<br>Dulaglutide 76<br>placebo 82	1.5 mg QW sc<br>3.0 mg QW sc<br>4.5 mg QW sc	8.1 ± 6.2	56.8 ± 9.7	50.8	33.0 ± 5.7	8.1 ± 0.8	All patients had been on stable monotherapy with metformin (dose ≥ 1500 mg/day) for at least 3 months prior to enrollment. During the trial, the combination of other hypoglycemic agents (e.g., insulin, GLP-1 receptor agonists, DPP-4 inhibitors, etc.) was prohibited. Rescue therapy (e.g., basal insulin or other non-GLP-1/non-DPP-4 agents) could be initiated in cases of severe hyperglycemia, but such initiation must be documented and the relevant data excluded.
E18	Kishore M. Gadde 2017	NCT01652729	RCT	28	365	Exenatide 181<br>Sitagliptin 122<br>placebo 61	2.0 mg QW sc	8.3 ± 5.6	53.7 ± 9.4	52.6	32.0 ± 5.6	8.4 ± 1.0	All patients had been on stable monotherapy with metformin (dose ≥ 1500 mg/day) for at least 2 months prior to enrollment. During the trial, the combination of other hypoglycemic agents

													(e.g., insulin, GLP-1 receptor agonists, sulfonylureas, etc.) was prohibited. Rescue therapy (e.g., basal insulin or other non-GLP-1/non-DPP-4 agents) could be initiated in cases of severe hyperglycemia, but such initiation must be documented and the relevant data excluded.
C14	A. Garber 2011	NCT00294723	RCT	104	746	Liraglutide 251 Liraglutide 246 Sulfonylureas 248	1.2 mg QD sc 1.8 mg QD sc	5.4 ± 5.3	53.4 ± 10.9	49.2	33.0 ± 5.8	8.2 ± 1.1	All patients had received diet/exercise management or monotherapy (e.g., metformin, sulfonylureas, etc.) for at least 3 months prior to enrollment. During the trial, monotherapy was maintained, and the combination of other hypoglycemic agents was prohibited. Rescue therapy could be initiated in cases of severe hyperglycemia, but such initiation must be documented and the relevant data excluded.
C15	W. Timothy Garvey 2020	NCT02963922	RCT	56	396	Liraglutide 198 placebo 198	3.0 mg QD sc	11.4 ± 6.8 12.8 ± 6.9	55.9 ± 11.3 57.6 ± 10.4	45.5 50.0	35.9 ± 6.5 35.3 ± 5.8	7.9 ± 1.1 8.0 ± 1.0	All patients had been receiving basal insulin (e.g., long-acting insulin analogs) therapy for at least 90 days prior to enrollment, combined with no more than two oral antidiabetic drugs (OADs) (e.g., metformin, sulfonylureas,

												SGLT-2 inhibitors, etc.) at stable doses. During the trial, adjustments to the insulin dose were permitted to maintain the fasting blood glucose target (4–5 mmol/L), but the use of GLP-1 receptor agonists or DPP-4 inhibitors was prohibited.
E19	Robert J. Heine 2005	NCT00082381	RCT	26	551	Exenatide 282 Insulin glargine 269	10 ug BID sc	9.9 ± 6.0 9.2 ± 5.7	59.8 ± 8.8 58.0 ± 9.5	55.8	31.4 ± 4.4 31.3 ± 4.6	8.2 ± 1.0 8.3 ± 1.0	All patients had been on combination therapy with metformin and sulfonylureas for at least 3 months prior to enrollment, with doses stabilized at the maximum effective doses. During the trial, the use of other hypoglycemic agents (e.g., insulin, GLP-1 receptor agonists, etc.) was prohibited; only dose adjustments of sulfonylureas were permitted for managing hypoglycemic events.
E9	Zinman, 2007	NCT00099320	RCT	16	233	Exenatide 121 Placebo 112	10 ug BID sc	7.3±4.9 8.2±5.8	55.6±10.8	53.7% 57.1%	34.0±5.1	7.9±0.9% 7.9±0.8%	All patients had been on a stable dose of thiazolidinediones (TZDs) for at least 4 months prior to enrollment.
M2	Yan, 2024	NCT04504370	RCT	52	273	Visepegenatide 137	150ug QW sc	2.20±2.4 2.16±2.60	50.3±10.25 50.4±11.41	70.6% 75.7%	26.53±3.51 26.52±3.73	8.47±0.81 8.53±0.81	All patients had received only diet plus exercise interventions with no prior hypoglycemic agent

						Placebo 136							therapy for at least 8 weeks before enrollment.
M1	Cai, 2024	NCT04504396	RCT	52	620	Visepegenatide 310 Placebo 310	150ug QW sc	5.09±4.65 5.18±4.47	52.7±10.92 53.7±10.63	63.2% 57.4%	26.67±3.70 26.28±3.51	8.48±0.86 8.46±0.79	All patients had been on stable monotherapy with metformin for at least 8 weeks prior to enrollment.
C16	Sten, 2004	NA	RCT	12	193	Randomized 1:1 to 5 liraglutide dose groups (0.045mg, 0.225mg, 0.45mg, 0.60mg, 0.75mg), placebo group, and open-label glimepiride group.	Liraglutide: Subcutaneous injection once daily,administered in the morning before breakfast, at fixed doses of0.045 mg,0.225mg,0	3.8±3.4	57.0±9.2	68.40%	30.5±4.8	7.6±0.9	All patients had received diet control or oral hypoglycemic agent (OHA) therapy prior to screening.

							.45mg, 0.60mg, and 0.75mg respectively.						
I1	Rajaa, 2021	NCT03235050	RCT	54	834	Cotadutide 100µg 100<br>Cotadutide 200µg 256<br>Cotadutide 300µg 256<br>Liraglutide 1.8mg 110<br>Placebo 112	Cotadutide 100µg QD sc<br>Cotadutide 200µg QD sc<br>Cotadutide 300µg QD sc<br>Liraglutide 1.8mg QD sc	NA	NA	NA	NA	NA	All participants maintained a stable dose of metformin during the 2-week run-in period and throughout the entire study, with no dose adjustments permitted.
F6	Marc, 2015	NCT01147250	RCT	109	6068	Lixisenatide 3034<br>Placebo 3034	10 ug QW sc	9.2 ± 8.2<br>9.4 ± 8.3	59.9 ± 9.7<br>60.6 ± 9.6	69.6<br>69.1	30.1 ± 5.6<br>30.2 ± 5.8	7.7 ± 1.3<br>7.6 ± 1.3	Patients could have received prior hypoglycemic therapy (e.g., metformin, sulfonylureas, insulin, etc.), but they must have

												experienced an acute coronary event (myocardial infarction or hospitalization for unstable angina pectoris) within 180 days prior to enrollment. Patients scheduled for coronary revascularization within 90 days or with an eGFR < 30 mL/min/1.73m² were excluded.
E10	Rury, 2017	NCT01144338	RCT	167	14752	Exenatide 7356 Placebo 7396	2.0 mg QW sc	NA	NA	62 62	NA	NA	Patients could receive ≤ 3 oral antidiabetic drugs or insulin (either as monotherapy or combined with ≤ 2 oral antidiabetic drugs). Patients were excluded if they had: ≥ 2 episodes of severe hypoglycemia (requiring assistance from others) within the past 12 months, an eGFR < 30 mL/min/1.73m², or a prior history of GLP-1 receptor agonist use.
B14	Kenneth, 2024	NA	RCT	177	3533	Semaglutide 1767 Placebo 1766	1.0 mg QW sc	NA	NA	NA	NA	NA	All patients received a stable dose of a renin-angiotensin system (RAS) inhibitor (either ACEI or ARB) at the maximum tolerated dose or labeled dose. Concomitant use of other hypoglycemic agents (e.g., insulin, oral antidiabetic drugs)

													was permitted; however, patients not receiving a baseline RAS inhibitor were excluded at enrollment. At baseline, 15.6% of participants were using SGLT2 inhibitors, and no one was using finerenone (as the relevant trials for finerenone had not been completed when this study was initiated).
J1	Jiang, 2022	NCT04466904	RCT	23	43	15 patients in the 3.0mg cohort, 14 in the 4.5mg cohort, and 14 in the 6.0mg cohort. Each cohort included 8 cases of IBI362, 4 of Placebo,	IBI362 (3.0mg/ 4.5mg/ 6.0mg) QW sc, weekly subcutaneous placebo, and weekly subcutaneous dulaglutide (1.5mg, active reference).	2.1 4.7 6.0 3.3 3.1	58.6 ± 5.3 47.9 ± 8.9 54.6 ± 10.6 50.7 ± 4.8 50.2 ± 8.5	75 37.5 62.5 100 50	24.1 ± 1.4 25.7 ± 3.4 26.0 ± 2.2 26.6 ± 3.0 26.5 ± 2.7	8.9 ± 0.7 8.8 ± 1.0 8.5 ± 1.1 8.3 ± 1.4 8.3 ± 0.7	Prohibited medications: GLP-1 receptor agonists, SGLT2 inhibitors, DPP-4 inhibitors, and insulin (within 3 months prior to enrollment).

						and 2 of dulaglutide (1 dulaglutide case in the 6.0mg cohort was not dosed).							
J2	Zhang, 2024	NCT04965506	RCT	27	252	Mazdutide 3mg 51<br>Mazdutide 4.5mg 49<br>Mazdutide 6mg 49<br>Dulaglutide 1.5mg 50<br>Placebo 51	Mazdutide 3mg QW sc<br>Mazdutide 4.5mg QW sc<br>Mazdutide 6mg QW sc<br>Dulaglutide 1.5mg QW sc<br>Placebo QW sc	4.9	53.5	59.2	27.4	8.11 ± 0.84<br>8.11 ± 0.91<br>7.94 ± 0.93<br>7.94 ± 0.92<br>8.16 ± 0.91	Prohibited medications: GLP-1 receptor agonists, SGLT2 inhibitors, DPP-4 inhibitors, and insulin (within 3 months prior to enrollment).

K1	Juan, 2023	NCT05048719	RCT	30	383	Placebo 55 Orforglipron 3mg 51 Orforglipron 12mg 56 Orforglipron 24mg 47 Orforglipron 36mg 61 Orforglipron 45mg 63 Dulaglutide 1.5mg 50	9 groups (stratified by dose and administration regimen): Once-daily oral Orforglipron (3mg/12mg/24mg/36mg subgroup 1/36mg subgroup 2/45mg subgroup 1/45mg subgroup 2)	8	58.9	59	35.2	8.1 ± 0.9 8.0 ± 0.8 8.2 ± 0.9 8.2 ± 0.9 8.1 ± 0.9 8.1 ± 0.9 8.0 ± 0.7	Prohibited medications: GLP-1 receptor agonists, SGLT2 inhibitors, and DPP-4 inhibitors (within 3 months prior to enrollment).

							Once-daily oral placebo Once-weekly subcutaneous dulaglutide 1.5mg (active reference)						
K2	Sean, 2025	NCT05048719	RCT	30	383	Orforglipron 3mg 47 Orforglipron 12mg 50 Orforglipron 24mg 42 Orforglipron 36mg 59 Orforglip	Once-daily oral Orforglipron (3mg/12mg/24mg/36mg/45mg), once-weekly subcutaneous dulaglutide	8	59	60	35.3 ± 6.9	8.1 ± 0.8	Prohibited: GLP-1 receptor agonists, SGLT2 inhibitors, and DPP-4 inhibitors (within 3 months prior to enrollment).

						ron 45mg 59 Dulagluti de 1.5mg 50 Placebo 54	1.5mg (active referen ce), and placebo						
B16	Ofri , 2019	NCT028 27708	RC T	33	324	Semaglut ide 163 Placebo 161	14 mg QD po	14.1 ± 8.6 13.9 ± 7.4	71.0 ± 8.0 70.0 ± 8.0	51 45	32.2 ± 5.4 32.6 ± 5.5	8.0 ± 0.7 7.9 ± 0.7	Prohibited medications: GLP-1 receptor agonists, DPP-4 inhibitors, and SGLT2 inhibitors (within 3 months prior to enrollment).
B15	Mans oor, 2019	NCT026 92716	RC T	69	3183	Semaglut ide 1591 Placebo 1592	14 mg QD po	14.7 ± 8.5 15.1 ± 8.5	66 ± 7 66 ± 7	68. 1 68. 6	32.3 ± 6.6 32.3 ± 6.4	8.2 ± 1.6 8.2 ± 1.6	Prohibited medications: GLP-1 receptor agonists, DPP-4 inhibitors, and SGLT2 inhibitors (within 3 months prior to enrollment).
D8	Hertz el,201 9	NCT013 94952	RC T	280	9901	Dulagluti de 4949 Placebo 4952	1.5 mg QW sc	10.5 ± 7.3 10.6 ± 7.2	66.2 ± 6.5 66.2 ± 6.5	53. 7	32.3 ± 5.7 32.3 ± 5.8	7.3 ± 1.1 7.4 ± 1.1	NA
A6	Stefan o,202 1	NCT037 30662	RC T	85	2002	Tirzepati de 5mg 329 Tirzepati de 10mg 328 Tirzepati	5 mg QW sc 10 mg QW sc 15 mg QW sc 100U/	9.8 10.6 10.4 10.7	62.9±8. 6 63.7±8. 7 63.7±8. 6	62	32.6±6. 06 32.8±5. 51 32.5±5. 02	8.52±0. 049 8.60±0. 049 8.52±0. 048	Participants maintained their original baseline hypoglycemic therapy throughout the entire study, including any one or combination of metformin, sulfonylureas, and sodium-glucose cotransporter 2 (SGLT-2)

						de 15mg 338 Insulin Glargine 1000	mL QD sc		63.8±8.5		32.5±5.55[2]	8.51±0.028	inhibitors. The use of GLP-1 receptor agonists, DPP-4 inhibitors, and pramlintide was not permitted. In case of hypoglycemia, the baseline hypoglycemic agents could be reduced or discontinued. For persistent hyperglycemia, additional hypoglycemic agents could be used as rescue therapy in accordance with predefined criteria (or after early discontinuation of the study drug).
B17	Steven, 2016	NCT01720446	RCT	109	3297	Randomized 1:1:1:1 to 4 groups: 0.5mg semaglutide group 1.0mg semaglutide group 0.5mg placebo group (volume-matched	Both semaglutide and placebo were administered subcutaneously once weekly (QW sc), using a fixed	14.3±8.2<br>14.1±8.2<br>14.0±8.5<br>13.2±7.4	64.6±7.3<br>64.7±7.1<br>64.8±7.6<br>64.4±7.5	60.7	NA	8.7±1.4<br>8.7±1.5<br>8.7±1.5<br>8.7±1.5	Patients had either received no hypoglycemic agent therapy or were on no more than 2 oral antidiabetic drugs (with or without combination of basal insulin/premixed insulin). During the trial: Investigators adjusted the treatment in accordance with local guidelines to achieve effective glycemic control, and could add or modify non-study hypoglycemic agents (non-incretin-based agents). The frequency of adding additional hypoglycemic agents (including insulin) was higher in the placebo

	to 0.5mg semaglutide group) 1.0mg placebo group (volume-matched to 1.0mg semaglutide group)	dose-escalation regimen: initial dose of 0.25mg for 4 weeks, followed by 0.5mg for 4 weeks, and finally reaching the maintenance dose (0.5mg or 1.0mg); no adjustment of the mainten		group than in the semaglutide group.

							ance dose was allowed during treatme nt.						
B18	Vlado , 2024	NCT038 19153	RC T	163	3533	Semaglut ide 1767 Placebo 1766	1.0mg QW sc	NA	66.6±9. 0 66.7±9. 0	69. 70 %	31.9±6. 1 32.0±6. 5	7.8±1.3 7.8±1.3	Enrollment criteria: Patients must be receiving a stable dose of a renin-angiotensin system (RAS) inhibitor (angiotensin-converting enzyme inhibitor [ACEI] or angiotensin receptor blocker [ARB]) at the maximum labeled dose or the maximum tolerated dose without unacceptable adverse effects; patients who cannot use RAS inhibitors due to adverse effects are also eligible for enrollment. Permitted medications: During the trial, SGLT2 inhibitors,

													mineralocorticoid receptor antagonists (MRAs), lipid-lowering drugs, diuretics, insulin, and other medications may be used, with doses required to remain stable; at baseline, 15.6% (550 patients) were using SGLT2 inhibitors, 80.2% (2834 patients) were using lipid-lowering drugs, 50.4% (1780 patients) were using diuretics, and 61.4% (2168 patients) were using insulin. Prohibited/restricted medications: No other baseline hypoglycemic agents are prohibited, but doses must be maintained stable; the initiation or adjustment of medications that may affect the study outcomes is not allowed.
E20	Wang, 2020	NCT02690883	RCT	24	92	Exenatide + Insulin Glargine 37<br>Insulin Lispro + Insulin Glargine 36	10 ug BID sc	10.9±6.2<br>11.4±7.0	55.9±8.9<br>56.2±8.0	76.1<br>63.0	26.1±3.4<br>23.5±3.1	9.1±1.4<br>9.0±1.3	Medications prior to enrollment: 75.0% (69/92) of patients were using insulin, 38.0% (35/92) were using metformin, 15.2% (14/92) were using sulfonylureas, and 26.1% (24/92) were using aminoglycoside inhibitors. Restrictions during the study: After the run-in period, all hypoglycemic agents except the study drugs (exenatide / insulin

													lispro + insulin glargine) were discontinued; the use of antihypertensive drugs, lipid-lowering drugs, and antiplatelet drugs was permitted (with doses required to be maintained stable). Among these, 87.0% (80/92) of patients were using angiotensin-converting enzyme inhibitors (ACEIs) or angiotensin II receptor blockers (ARBs), 87.0% (80/92) were using statins, and 90.2% (83/92) were using at least one type of antihypertensive drug.
G3	Cao，2024	ChiCTR2300070919	RCT	24	88	Polyethylene Glycol Loxenatide 44<br>Dapagliflozin 44	0.2 mg QW sc	7.8±3.9<br>7.3±3.7	51.0±9.3<br>48.4±10.1	45.0<br>57.5	26.4±1.4<br>26.3±1.6	8.31±0.71<br>8.31±0.77	Medication requirements during the study: The doses of baseline hypoglycemic agents were maintained unchanged; the use of antihypertensive drugs (e.g., ACEI/ARB, with usage rates of 47.5% and 45.0% in the two groups, respectively), lipid-lowering drugs (usage rates of 47.5% and 57.5% in the two groups, respectively), and diuretics (usage rates of 7.5% and 5.0% in the two groups, respectively) was permitted, but doses were required to remain stable. Patients who had used

												GLP-1 receptor agonists or SGLT2 inhibitors within 12 weeks prior to enrollment were excluded.
C24	Jiang, 2020	NA	RCT	24	161	Dapagliflozin 81<br>Liraglutide 80	10mg QD po<br>1.8 mg QD sc	9.14±6.07<br>9.04±6.11	56.03±10.02<br>55.29±10.53	67<br>61	26.65±4.06<br>26.62±3.68	8.96±1.23<br>8.99±1.34	Medication requirements during the study: The dose of baseline triple therapy was maintained unchanged; the concurrent use of antihypertensive drugs (e.g., ACEI/ARB), lipid-lowering drugs, and other concomitant medications was permitted, but doses were required to remain stable. Patients with acute diabetic complications, severe cardio-cerebrovascular diseases, hepatic or renal insufficiency, or allergies to the study drug prior to enrollment were excluded.
E11	Christian,2022	NCT01455896	RCT	70	4156	ITCA 650 2075<br>Placebo 2081	60ug QD	10.4<br>10.2	63<br>63	62.5<br>64.1	32.4<br>31.9	NA	The use of GLP-1 receptor agonists, SGLT2 inhibitors, and DPP-4 inhibitors is prohibited. Adjustments to other hypoglycemic agents shall follow the principle of "maintaining target blood glucose levels," with no mandatory medication restrictions.

L1	Julio, 2023	NCT04867785	RCT	43	281	Retatrutide: 190<br><br>Dulaglutide 1.5mg 46<br>Placebo 45	Retatrutide (0.5mg/4mg escalation/4mg non-escalation/8mg slow escalation/8mg fast escalation/12mg escalation) QW scDulaglutide 1.5mg QW sc	7.2-10.5 年<br>8.7 ± 8.3<br>8.7 ± 8.3	52.0-57.7<br>56.2 ± 9.7<br>56.2 ± 9.7	44.1	33.8-36.3<br>35.5 ± 6.9<br>33.8 ± 4.9	8.0-8.4<br>8.2 ± 1.1<br>8.4 ± 1.1	Prohibited medications: GLP-1 receptor agonists, SGLT2 inhibitors, and DPP-4 inhibitors (within 3 months prior to enrollment).

N1	Matthias, 2023	NCT04153929	RCT	16	411	Survodutide：302 Semaglutide 50 Placebo 59	DG1 (0.3mg once weekly): Subcutaneous injection once weekly, no dose escalation, directly maintained at 0.3mg/week; DG2 (0.9mg once weekly): Dose escalation to 0.9mg/week for maintenance;	5.1±4.7（DG1）-8.8±7.1（DG5） 7.9±4.7 7.9±5.6	55.3±10.3（DG3）-59.6±8.5（DG4） 55.8±10.5 57.5±10.5	51.9（DG3）-66.0（DG4） 68.0 52.5%	33.0±5.0（DG5）-34.9±7.0（DG6） 33.4±6.1 33.4±5.9	7.89±0.80（DG2）-8.18±0.97（DG4） 8.03±0.82 8.15±0.85	All patients had been on stable monotherapy with metformin at a constant dose for at least 3 months prior to enrollment.

DG3 (1.8mg once weekly): Dose escalation to 1.8mg/week for maintenance; DG4 (2.7mg once weekly): Dose escalation to 2.7mg/week for maintenance; DG5 (1.2mg twice weekly): Dose escalati

	on to 1.2mg/ dose, twice weekly (total dose 2.4mg/ week) for mainten ance; DG6 (1.8mg twice weekly) : Dose escalati on to 1.8mg/ dose, twice weekly (total dose 3.6mg/ week) for mainten ance;		

	Semaglutide 1.0mg QW sc	

Supplementary 3: List of data extracted from the included randomized clinical trials

Data category	List of variables
Study	Primary author, year of publication, duration of study, total number of patients in each group
Patients	Sex, age, diabetes duration, HbA_{1c} and BMI at baseline
Interventions	Drug class, dose and duration of the primary intervention and strategies used for implementing them
Efficacy outcome	Mean of change in HbA1c (%), FPG (mmol/L), weight (kg), HDL (mmol/L), LDL (mmol/L), TC (mmol/L), TG (mmol/L), SBP (mmHg), DBP (mmHg), GFR (mL/min/1.73m²), UACR (mg/mmol), FIL (mIU/L), HOMA-β, HOMA-IR, and C-peptide (nmol/L) with respective standard deviation from baseline ; proportion of patients achieving blood glucose targets of 7.0% and 6.5%; ACM, NFS, NFM, CVM, MACE
Adverse events	Various adverse events reported in included trials

Abbreviations: FPG, fasting plasma glucose; HDL, highdensity lipoprotein; LDL, low-density lipoprotein; TC, total cholesterol; TG, triglyceride; SBP, systolic blood pressure; DBP, diastolic blood pressure, ACM, all-cause mortality; NFS, non-fatal stroke; NFM, non-fatal myocardial infarction; CVM, cardiovascular mortality; MACE, major adverse cardiovascular events; GFR, glomerular filtration rate; UACR, urinary albumin/creatinine ratio; FIL, fasting insulin level; HOMA-β, homeostasis model assessment of β-cell function; HOMA-IR, homeostasis model assessment of insulin resistance.

Supplementary 4: Risk of bias of randomized clinical trials

Figure S4: Overall risk of bias presented as percentage of each risk of bias item across all included studies.

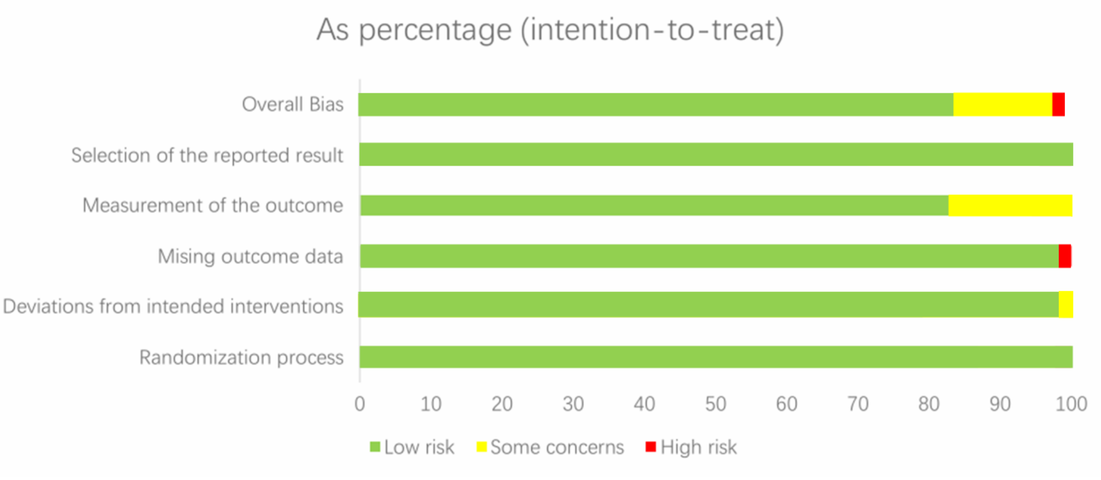

Green = Low risk, Red = High risk, Yellow = Some concerns

.

Table S4: Study level risk of bias assessment using Cochrane risk of bias tool 2.0 for assessing risk of bias of randomized clinical trials.

Unique ID	Study ID	Randomization process	Deviations from intended interventions	Mising outcome data	Measurement of the outcome	Selection of the reported result
C1	BERNARD 2009	Low	Low	High	Low	Low
B1	Bernard 2019	Low	Low	Low	Low	Low
B2	Zinman 2019	Low	Low	Low	Low	Low
C19	L 2016	Low	Low	Low	Low	Low
F1	W Yang	Low	Low	Low	Low	Low
C2	W 2010	Low	Low	Low	Low	Low
B3	WANG 2024	Low	Low	Low	Low	Low
B19	Yuichiro 2020	Low	Low	Low	Low	Low
B22	Daisuke 2020	Low	Low	Low	Low	Low
E13	Carol	Low	Low	Low	Low	Low
D1	Weinstock 2015	Low	Low	Low	Low	Low
D9	Wang	Low	Low	Low	Low	Low
C20	Wang 2022	Low	Low	Low	Low	Low
C3	Anna 2016	Low	Low	Low	Low	Low
D2	Guillermo 2014	Low	Low	Low	Low	Low
C4	Miyagawa 2015	Low	Low	Low	Low	Low

B4	Christopher 2017	Low	Low	Low	Low	Low
G1	ShuaiM 2020	Low	Low	Low	Low	Low
B23	Yutaka	Low	Low	Low	Some concerns	Low
C5	Seino 2010	Low	Low	Low	Low	Low
C21	Yutaka 2022	Low	Low	Low	Some concerns	Low
C6	Russell-Jones	Low	Low	Low	Low	Low
E1	DAVID 2011	Low	Low	Low	Low	Low
A1	Rosenstock 2021	Low	Low	Low	Low	Low
H1	Rosenstock 2019	Low	Low	Low	Low	Low
F7	JULIO 2013	Low	Low	Low	Some concerns	Low
B5	Julio 2023	Low	Low	Low	Low	Low
B24	Helena	Low	Low	Low	Some concerns	Low
B6	Helena 2018	Low	Low	Low	Low	Low
F9	Michael 2016	Low	Low	Low	Some concerns	Low
F3	MATTHEW 2013	Low	Low	Low	Low	Low
B25	Richard 2018	Low	Low	Low	Low	Low
B20	Richard 2019	Low	Some concerns	Low	Low	Low
D3	Pozzilli	Low	Low	Low	Low	Low
C7	Ahmann 2015	Low	Low	Low	Low	Low
C22	Azar 2016	Low	Low	Low	Some concerns	Low
C23	Francisco 2021	Low	Low	Low	Low	Low
F4	Pan 2014	Low	Low	Low	Low	Low
D4	Tomoaki 2024	Low	Low	Low	Low	Low
F5	Graydon 2017	Low	Low	Low	Low	Low
C8	Melanie 2015	Low	Low	Low	Low	Low
C9	Marre 2009	Low	Low	Low	Low	Low
E2	Lu 2012	Low	Low	Low	Low	Low
B7	Lingvay 2018	Low	Low	Low	Low	Low
B8	Ildiko 2019	Low	Low	Low	Low	Low
C10	Lind 2015	Low	Low	Low	Low	Low
B21	Tomohiko 2023	Low	Low	Low	Low	Low
E3	DAVID 2005	Low	Low	Low	Low	Low
B26	Kohei	Low	Low	Low	Low	Low
C11	Kaku 2010	Low	Low	Low	Low	Low
A5	Kadowaki 2022	Low	Low	Low	Some concerns	Low
E4	Michael 2020	Low	Low	Low	Low	Low
B9	Bo 2017	Low	Low	Low	Low	Low
E14	Linong 2013	Low	Low	Low	Low	Low
B10	Ji 2023	Low	Low	Low	Low	Low
E5	Serge 2018	Low	Low	Low	Low	Low

A2	Nobuya 2022	Low	Low	Low	Low	Low
B11	Frías 2021	Low	Low	Low	Low	Low
D11	Araki 2015	Low	Low	Low	Low	Low
B12	Aroda 2023	Low	Low	Low	Low	Low
B13	Vanita 2019	Low	Low	Low	Low	Low
E6	Bergenstal 2010	Low	Low	Low	Low	Low
E15	Thomas 2011	Low	Low	Low	Low	Low
D10	Lawrence 2015	Low	Low	Low	Low	Low
E7	John 2010	Low	Low	Low	Low	Low
E16	Buse 2012	Low	Low	Low	Some concerns	Low
A3	Dominik 2022	Low	Low	Low	Low	Low
E12	ELANIE 2013	Low	Low	Low	Some concerns	Low
C13	Melanie 2015	Low	Low	Low	Low	Low
H2	Stefano 2020	Low	Low	Low	Low	Low
E17	Daniel 2008	Low	Low	Low	Low	Low
D12	Kathleen 2014	Low	Low	Low	Low	Low
H3	Juan 2022	Low	Low	Low	Low	Low
D7	Juan	Low	Low	Low	Low	Low
E18	Kishore 2017	Low	Low	Low	Low	Low
C14	Garber 2010	Low	Low	Low	Low	Low
C15	Timothy 2019	Low	Low	Low	Low	Low
E19	Robert 2005	Low	Low	Low	Low	Low
E9	Bernard 2013	Low	Low	Low	Low	Low
M2	Yan 2024	Low	Low	Low	Low	Low
M1	Cai 2024	Low	Low	Low	Low	Low
C16	MADSBAD 2004	Low	Low	Low	Low	Low
I1	Rajaa 2021	Low	Low	Low	Low	Low
F6	Marc 2015	Low	Low	Low	Low	Low
E10	Rury 2017	Low	Low	Low	Low	Low
B14	Kenneth	Low	Low	Low	Low	Low
J1	Jiang 2022	Low	Low	Low	Low	Low
J2	Zhang 2023	Low	Low	Low	Low	Low
K1	Juan 2023	Low	Low	Low	Low	Low
B16	Ofri 2019	Low	Low	Low	Low	Low
B15	Mansoor 2019	Low	Low	Low	Low	Low
D8	Hertzel 2019	Low	Low	Low	Low	Low
A6	Stefano 2021	Low	Low	Low	Some concerns	Low
B17	Steven 2016	Low	Low	Low	Low	Low
B18	Vlado 2024	Low	Low	Low	Low	Low
E20	Wang 2020	Low	Low	Low	Low	Low
G3	Cao	Low	Low	Low	Low	Low
C24	Jiang 2020	Low	Low	Low	Low	Low
E11	Christian,2022	Low	Low	Low	Low	Low

K2	Sean，2025	Low	Low	Low	Low	Low
L1	Julio,2023	Low	Low	Low	Low	Low
N1	Matthias，2023	Low	Low	Low	Low	Low

Supplementary 5: Evaluation of inconsistency and heterogeneity

Table S5.1: I^2 value of each clinical outcome

Clinical outcome	I^2
HbA_{1c} change from baseline	35%
FPG change from baseline	30%
Body weight change from baseline	45%
The proportion of patients with HbA_{1c} reaching 7.0%	40%
The proportion of patients with HbA_{1c} reaching 6.5	45%
SBP change from baseline	50%
DBP change from baseline	48%
HDL change from baseline	40%
LDL change from baseline	55%
TG change from baseline	52%
TC change from baseline	50%
MACE	70%
NFS	35%
NFM	38%
ACM	65%
CVM	72%
GFR change from baseline	45%
UACR change from baseline	48%
HOMA-IR change from baseline	42%

HOMA-βchange from baseline	40%
C-peptide change from baseline	38%
FIL change from baseline	45%

Abbreviations: FPG, fasting plasma glucose; HDL, high density lipoprotein; LDL, low-density lipoprotein; TC, total cholesterol; TG, triglyceride; SBP, systolic blood pressure; DBP, diastolic blood pressure, ACM, all-cause mortality; NFS, non-fatal stroke; NFM, non-fatal myocardial infarction; CVM, cardiovascular mortality; MACE, major adverse cardiovascular events; GFR, glomerular filtration rate; UACR, urinary albumin/creatinine ratio; FIL, fasting insulin level; HOMA-β, homeostasis model assessment of β-cell function; HOMA-IR, homeostasis model assessment of insulin resistance; and C-peptide.

Table S5.2: splitting of HbA_{1c}. Inconsistency test between direct and indirect treatment comparisons in mixed treatment comparison.

Comparison	Direct		Indirect		Difference		
	Coef.	CrI	Coef.	CrI	Coef.	CrI	P>|z|
Dulaglutide v.s. Semaglutide	-0.27	(-0.62,0.091)	-0.30	(-0.47,-0.12)	-0.29	(-0.45,-0.14)	0.87467
Dulaglutide v.s. Exenatide	0.42	(-0.20,1.0)	0.018	(-0.19,0.23)	0.065	(-0.13,0.26)	0.23036
Dulaglutide v.s. Liraglutide	0.18	(-0.43,0.80)	-0.012	(-0.18,0.15)	0.0021	(-0.16,0.16)	0.54621
Dulaglutide v.s. Tirzepatide	-0.89	(-1.2,-0.55)	-0.97	(-1.3,-0.69)	-0.92	(-1.1,-0.71)	0.69418
Dulaglutide v.s. Placebo	1.0	(0.85 1.2)	0.97	(0.76 1.2)	1.0	(0.88 1.1)	0.75387
Dulaglutide v.s. others	0.25	(0.0074 0.49)	0.34	(0.15 0.54)	0.31	(0.16 0.46)	0.56101
Semaglutide v.s. Liraglutide	0.41	(0.12,0.71)	0.25	(0.070,0.43)	0.29	(0.14,0.44)	0.35131
Semaglutide v.s. Efpeglenatide	0.40	(-0.47,1.3)	0.54	(0.27,0.82)	0.53	(0.27,0.79)	0.76047
Exenatide v.s. Liraglutide	0.20	(-0.68,1.1)	-0.078	(-0.28,0.12)	-0.063	(-0.26,0.13)	0.54123
Exenatide v.s. Lixisenatide	0.15	(-0.72,1.0)	0.52	(0.13,0.91)	0.46	(0.096,0.82)	0044606
Liraglutide v.s. Lixisenatide	0.60	(-0.27,1.5)	0.51	(0.13,0.88)	0.52	(0.18,0.86)	0.84556
Liraglutide v.s. Efpeglenatide	0.22	(-0.17,0.62)	0.25	(-0.11,0.61)	0.24	(-0.017,0.49)	0.90927
Tirzepatide v.s. Placebo	1.9	(1.7,2.2)	2.0	(1.6,2.4)	1.9	(1.7,2.1)	0.81811
Tirzepatide v.s.	0.98	(0.47,1.5)	1.3	(1.0,1.5)	1.2	(1.2,1.4)	0.27745

others							
Semaglutide v.s. Placebo	1.2	(1.1,1.4)	1.4	(1.2,1.7)	1.3	(1.2,1.4)	0.10547
Exenatide v.s. Placebo	0.97	(0.76,1.2)	0.91	(0.60,1.2)	0.94	(0.77,1.1)	0.74107
PEG-Loxenatidev.s. Placebo	1.0	(0.39,1.6)	0.80	(0.17,1.4)	0.91	(0.47,1.4)	0.64711
Liraglutide v.s. Placebo	1.0	(0.89 1.2)	0.97	(0.74 1.2)	1.0	(0.89 1.1)	0.65327
Lixisenatide v.s. Placebo	0.49	(0.051 0.93)	0.48	(-0.031 1.0)	0.49	(0.15 0.82)	0.98741
Efpeglenatide v.s. Placebo	0.75	(0.49,1.0)	0.90	(0.013,1.8)	0.77	(0.53,1.0)	0.75160
Semaglutide v.s. others	0.74	(0.52,0.97)	0.49	(0.30,0.68)	0.60	(0.45,0.74)	0.09610
Exenatide v.s. others	0.22	(-0.14,0.58)	0.25	(0.021,0.48)	0.24	(0.050,0.43)	0.90105
PEG-Loxenatidev.s. others	0.11	(-0.51,0.73)	0.31	(-0.32,0.95)	0.21	(-0.23,0.65)	0.64648
Liraglutide v.s. others	0.34	(0.11,0.57)	0.28	(0.087,0.47)	0.30	(0.16,0.45)	0.69906
Lixisenatide v.s. others	-0.45	(-1.3,0.42)	-0.17	(-0.55,0.21)	-0.22	(-0.56,0.13)	0.56627

Table S5.3: splitting of FPG. Inconsistency test between direct and indirect treatment comparisons in mixed treatment comparison.

Comparison	Direct		Indirect		Difference		
	Coef.	CrI	Coef.	CrI	Coef.	CrI	P>|z|
Dulaglutide v.s. Semaglutide	-0.47	(-1.4,0.50)	-0.74	(1.1,-0.38)	-0.71	(-1.0,-0.37)	0.60450
Dulaglutide v.s. Exenatide	0.77	(-0.42,2.0)	0.045	(-0.40,0.49)	0.14	(-0.27,0.56)	0.26078
Dulaglutide v.s. Liraglutide	0.32	(-0.87,1.5)	-0.24	(-0.59,0.12)	-0.19	(-0.53,0.15)	0.37764
Dulaglutide v.s. Tirzepatide	-1.4	(-2.0,-0.75)	-1.1	(-1.7,-0.56)	-1.3	(-1.7,-0.88)	0.51113
Dulaglutide v.s. Placebo	1.5	(1.2 1.8)	1.1	(0.68 1.6)	1.4	(1.1 1.6)	0.19701
Dulaglutide v.s. others	-0.13	(-0.60 0.33)	0.38	(-0.023 0.78)	0.16	(-0.14 0.47)	0.10129
Semaglutide v.s. Liraglutide	1.1	(0.37,1.7)	0.33	(-0.068,0.73)	0.51	(0.17,0.86)	0.07323
Semaglutide v.s. Efpeglenatide	0.11	(-1.6,1.8)	0.71	(0.16,1.3)	0.65	(0.13,1.2)	0.50847

Exenatide v.s. Liraglutide	-0.36	(-2.0,1.3)	-0.33	(-0.78,0.11)	-0.34	(-0.76,0.092)	0.97543
Exenatide v.s. Lixisenatide	0.19	(-1.5,1.9)	0.088	(-0.69,0.87)	0.11	(-0.60,0.81)	0.91051
Liraglutide v.s. Lixisenatide	0.60	(-1.1,2.3)	0.41	(-0.332,1.1)	0.44	(-0.23,1.1)	0.83811
Liraglutide v.s. Efpeglenatide	-0.17	(-0.92,0.58)	0.63	(-0.072,1.3)	0.14	(-0.36,0.64)	0.12546
Tirzepatide v.s. Placebo	2.9	(2.5,3.4)	2.2	(1.5,2.9)	2.7	(2.3,3.0)	0.08499
Tirzepatide v.s. others	0.21	(-0.74,1.2)	1.8	(1.3,2.2)	1.4	(1.0,1.9)	0.00480
Semaglutide v.s. Placebo	2.0	(1.6,2.3)	2.2	(1.8,2.7)	2.1	(1.8,2.4)	0.37010
Exenatide v.s. Placebo	1.2	(0.77,2.7)	1.3	(0.66,2.0)	1.2	(0.87,1.6)	0.80726
PEG-Loxenatide v.s. Placebo	1.5	(0.27,2.7)	1.2	(-0.52,2.9)	1.4	(0.40,2.4)	0.80325
Liraglutide v.s. Placebo	1.6	(1.3 1.9)	1.7	(1.2 2.2)	1.6	(1.3 1.8)	0.72611
Lixisenatide v.s. Placebo	0.40	(-0.43 1.2)	2.1	(1.1 3.1)	1.1	(0.48 1.8)	0.00846
Efpeglenatidev.s. Placebo	1.4	(0.88,1.9)	2.0	(0.24,3.7)	1.4	(0.96,1.9)	0.52691
Semaglutide v.s. others	1.0	(0.54,1.5)	0.75	(0.32,1.2)	0.87	(0.55,1.2)	0.41630
Exenatide v.s. others	0.45	(-0.39,1.3)	-0.12	(-0.59,0.35)	0.018	(-0.39,0.43)	0.25035
PEG-Loxenatide v.s. others	-0.00030	(-1.7,1.7)	0.26	(-0.96,1.5)	0.17	(-0.82,1.2)	0.80577
Liraglutide v.s. others	0.63	(0.099,1.2)	0.19	(-0.22,0.60)	0.35	(0.029,0.68)	0.19963
Lixisenatide v.s. others	3.0	(1.4,4.7)	-0.69	(-1.4,0.022)	-0.088	(-0.76,0.58)	0.00002

Table S5.4: splitting of Weight loss. Inconsistency test between direct and indirect treatment comparisons in mixed treatment comparison.

Comparison	Direct		Indirect		Difference		
	Coef.	CrI	Coef.	CrI	Coef.	CrI	P>|z|
Dulaglutide v.s. Semaglutide	-2.4	(-4.0, -0.76)	-1.9	(-2.7, -1.1)	-2	(-2.7, -1.3)	0.56755
Dulaglutide v.s. Exenatide	-0.40	(-3.2, 2.4)	-0.88	(-1.9, 0.094)	-0.84	(-1.8, 0.070)	0.75542

Dulaglutide v.s. Liraglutide	-0.33	(-3.2, 2.5)	-0.58	(-1.4, 0.19)	-0.36	(-1.5, 0.82)0	0.86742
Dulaglutide v.s. Tirzepatide	-5.5	(-7., -4.)	-7.5	(-8.8, -6.2)	-6.6	(-7.6, -5.7)	0.04441
Dulaglutide v.s. Placebo	0.99	(0.40, 1.6)	1.1	(0.32, 1.9)	1.1	(0.53, 1.7)	0.79663
Dulaglutide v.s. others	2.2	(1.1, 3.3)	1.9	(0.97, 2.8)	2.0	(1.3, 2.7)	0.63097
Semaglutide v.s. Liraglutide	2.6	(1.2, 3.9)	0.96	(0.13, 1.8)	1.4	(0.70, 2.1)	0.04445
Semaglutide v.s. Efpeglenatide	0.80	(-0.3, 1.9)	1.9	(0.62, 3.1)	0.9	(0.5, 1.3)	0.64462
Exenatide v.s. Liraglutide	-0.89	(-4.9, 3.1)	0.34	(-0.60, 1.3)	0.27	(-0.64, 1.2)	0.55851
Exenatide v.s. Lixisenatide	0.50	(-3.5, 4.5)	0.48	(-1.5, 2.4)	0.48	(-1.3, 2.2)	0.99442
Liraglutide v.s. Lixisenatide	0.50	(-3.5, 4.5)	0.15	(-1.7, 2.0)	0.21	(-1.5, 1.9)	0.8748
Liraglutide v.s. Efpeglenatide	1.1	(-0.76, 2.9)	-0.20	(-1.8, 1.4	0.38)	(-0.77, 1.5)	0.31390
Tirzepatide v.s. Placebo	7.3	(6.2, 8.3)	9.7	(8.0, 11.)	7.7	(6.8, 8.6)	0.01668
Tirzepatide v.s. others	11	(9.1, 14.)	8.6	(6.9, 9.7)	8.8	(7.6, 9.1)	0.00985
Semaglutide v.s. Placebo	3.4	(2.7, 4.1)	2.1	(1.1, 3.1)	3.1	(2.5, 3.6)	0.03125
Exenatide v.s. Placebo	1.9	(1.0, 2.9)	2.	(0.56, 3.4)	1.9	(1.2, 2.7)	0.96037
PEG-Loxenatide v.s. Placebo	-0.29	(-3.1, 2.5)	-0.41	(-4.5, 3.6)	-0.33	(-2.7, 2.)	0.96136
Liraglutide v.s. Placebo	1.6	(0.88, 2.3)	1.9	(0.77, 3.)	1.7	(1.1, 2.2)	0.64525
Lixisenatide v.s. Placebo	1.2	(-1.1, 3.5)	1.7	(-0.65, 4.1)	1.5	(-0.19, 3.2)	0.76543
Efpeglenatidev.s. Placebo	1.3	(0.13, 2.6)	2.2	(-1.9, 6.2)	1.3	(-0.19, 2.4)	0.70476
Semaglutide v.s. others	2.5	(1.5, 3.5)	5.1	(4.2, 6.)	4.	(3.3, 4.7)	0.00016
Exenatide v.s. others	2.8	(1.2, 4.5)	2.9	(1.8, 3.9)	2.8	(2., 3.7)	0.98103
PEG-Loxenatide v.s. others	0.51	(-3.5, 4.5)	0.61	(-2.3, 3.5)	0.57	(-1.8, 2.9)	0.96471
Liraglutide v.s. others	2.9	(1.8, 4.)	2.3	(1.4, 3.2)	2.6	(1.9, 3.3)	0.40276

Lixisenatide v.s. others	3.4	(-0.60, 7.4)	2.1	(0.23, 4.0)	2.4	(0.65, 4.1)	0.57046

Table S5.5: splitting of the proportion of patients with HbA1c reaching 7.0%. Inconsistency test between direct and indirect treatment comparisons in mixed treatment comparison.

Comparison	Direct		Indirect		Difference		
	Coef.	CrI	Coef.	CrI	Coef.	CrI	P>|z|
Dulaglutide v.s. Semaglutide	13	(2.9,24)	17	(11,23)	16	(11,21)	0.56776
Dulaglutide v.s. Exenatide	-15	(-33,2.9)	-6	(-16,4.5)	-10	(-19,-1.3)	0.38742
Dulaglutide v.s. Liraglutide	-3.3	(-21,15)	0.36	(-5.4,6.2)	0.020	(-5.5,5.5)	0.70366
Dulaglutide v.s. Tirzepatide	21	(12,31)	24	(14,33)	23	(16,29)	0.75317
Dulaglutide v.s. Placebo	-37	(-42 -31)	-33	(-40 -27)	-36	(-40 -31)	0.44199
Dulaglutide v.s. others	-11	(-19 -2.8)	-19	(-26 -12)	-16	(-21 -10)	0.110000
Semaglutide v.s. Liraglutide	-22	(-30,-13)	-12	(-19,-5.8)	-16	(-21,-11)	0.08596
Semaglutide v.s. Efpeglenatide	-24	(-50,1.4)	-10	(-19,-0.87)	-12	(-21,-3.1)	0.31330
Liraglutide v.s. Lixisenatide	-29	(-54,-3.2)	-18	(-33,-2.1)	-20	(-34,-7.3)	0.45834
Liraglutide v.s. Efpeglenatide	5.1	(-6.5,17)	1.8	(-12,15)	4.1	(-4.2,12)	0.71746
Tirzepatide v.s. Placebo	-59	(-67,-51)	-60	(-71,-49)	-58	(-65,-52)	0.84725
Tirzepatide v.s. others	-36	(-50,-21)	-39	(-48,-31)	-38	(-46,-31)	0.66897
Semaglutide v.s. Placebo	-51	(-57,-45)	-52	(-59,-44)	-52	(-56,-47)	0.92788
Exenatide v.s. Placebo	-16	(-26,-6.0)	-45	(-60,-29)	-26	(-34,-17)	0.00259
Liraglutide v.s. Placebo	-37	(-41 -32)	-35	(-44 -27)	-36	(-40 -32)	0.83706
Lixisenatide v.s. Placebo	-18	(-33 -3.3)	-6.9	(-33 19)	-15	(-28 -2.5)	0.45789
Efpeglenatidev.s. Placebo	-41	(-51,-32)	-27	(-54,-1.1)	-40	(-48,-32)	0.31086
Semaglutide v.s. others	-31	(-37,24)	-33	(-41,-26)	-32	(-37,-27)	0.59540

Exenatide v.s. others	-22	(-37,-7.9)	3.9	(-7.0,15)	-5.6	(-15,3.3)	0.00505
Liraglutide v.s. others	-19	(-29,-9.7)	-14	(-21,-7.2)	-16	(-21,-10)	0.37517

Table S5.6: splitting of the proportion of patients with HbA1c reaching 6.5%. Inconsistency test between direct and indirect treatment comparisons in mixed treatment comparison.

Comparison	Direct		Indirect		Difference		
	Coef.	CrI	Coef.	CrI	Coef.	CrI	P>|z|
Dulaglutide v.s. Semaglutide	6.8	(-4.7,18)	22	(16,29)	18	(13,24)	0.02187
Dulaglutide v.s. Exenatide	-20	(-38,-1.5)	-5.8	(-18,6.7)	-12	(-22,-1.6)	0.21394
Dulaglutide v.s. Liraglutide	-5.5	(-24,13)	0.19	(-6.0,6.4)	-0.41	(-6.3,5.5)	0.56219
Dulaglutide v.s. Tirzepatide	24	(14,34)	33	(23,43)	28	(22,35)	0.21881
Dulaglutide v.s. Placebo	-31	(-36,-26)	-31	(-38,-24)	-31	(-36,-27)	0.95920
Dulaglutide v.s. others	-11	(-19,-3.2)	-26	(-34,-19)	-20	(-25,-14)	0.00725
Semaglutide v.s. Liraglutide	-19	(-30,-6.8)	-19	(-226,-12)	-19	(-25,-13)	0.94285
Semaglutide v.s. Efpeglenatide	-31	(-57,-4.6)	-16	(-26,-5.7)	-18	(-27,-8.3)	0.29068
Liraglutide v.s. Lixisenatide	-28	(-55,-2.2)	-17	(-33,-1.0)	-20	(-33,-6.4)	0.45591
Liraglutide v.s. Efpeglenatide	-7.8	(-19,3.8)	7.5	(-6.2,21)	1.2	(-7.4,9.8)	0.09445
Tirzepatide v.s. Placebo	-62	(-70,-54)	-59	(-70,-47)	-60	(-66,-53)	0.67785
Tirzepatide v.s. others	-42	(-57,-27)	-50	(-59,-41)	-48	(-56,-40)	0.39853
Semaglutide v.s. Placebo	-47	(-54,-39)	-53	(-61,-45)	-50	(-55,-44)	0.22670
Exenatide v.s. Placebo	-9.1	(-22,3.7)	-37	(-53,-21)	-20	(-29,-9.8)	0.00815
Liraglutide v.s. Placebo	-31	(-37,-26)	-28	(-37,-18)	-31	(-35,-26)	0.47972
Lixisenatide v.s. Placebo	-14	(-29,1.3)	-2.2	(-29,24)	-11	(-24,2.1)	0.45665
Efpeglenatidev.s. Placebo	-39	(-48,-29)	-18	(-44,8.6)	-32	(-40,-24)	0.13872

Semaglutide v.s. others	-44	(-52,-37)	-31	(-39,-23)	-38	(-44,-32)	0.01852
Exenatide v.s. others	-23	(-37,-7.9)	3.7	(-9.3,17)	-7.8	(-18,2.1)	0.00921
Liraglutide v.s. others	-17	(-27,-6.9)	-21	(-28,-13)	-19	(-25,-13)	0.5509

Table S5.7 splitting of HDL. Inconsistency test between direct and indirect treatment comparisons in mixed treatment comparison.

Comparison	Direct		Indirect		Difference		
	Coef.	CrI	Coef.	CrI	Coef.	CrI	P>|z|
Dulaglutide v.s. Semaglutide	-0.14	(-0.21,-0.070)	0.069	(0.024,0.11)	0.0080	(-0.043,0.059)	0.00002
Dulaglutide v.s. Placebo	0.080	(0.049,0.11)	-0.13	(-0.20,-0.054)	0.050	(0.011,0.089)	0.00003
Exenatide v.s. Liraglutide	6.2e-05	(-0.094,0.094)	0.056	(0.0084,0.10)	0.045	(0.0023,0.087)	0.28518
Semaglutide v.s. Placebo	0.0051	(-0.035,0.045)	0.13	(0.070,0.20)	0.042	(0.0024,0.082)	0.00160
Exenatide v.s. Placebo	0.026	(-0.016,0.068)	-0.010	(-0.078,0.057)	0.016	(-0.020,0.051)	0.36188
PEG-Loxenatide v.s. Placebo	-0.010	(-0.078,0.057)	0.028	(-0.079,0.14)	0.00096	(-0.056,0.057)	0.53804
Liraglutide v.s. Placebo	-0.033	(-0.070,0.0042)	-0.013	(-0.086,0.059)	-0.029	(-0.060,0.0028)	0.63215
Semaglutide v.s. others	0.040	(-0.056,0.14)	0.039	(-0.028,0.11)	0.039	(-0.014,0.093)	0.98374
Exenatide v.s. others	0.0099	(-0.057,0.078)	0.016	(-0.050,0.082)	0.013	(-0.033,0.060)	0.89893
PEG-Loxenatide v.s. others	0.020	(-0.076,0.12)	-0.018	(-0.10,0.065)	-0.0019	(-0.064,0.060)	0.53906
Liraglutide v.s. others	-0.040	(-0.11,0.027)	-0.024	(-0.088,0.039)	-0.032	(-0.077,0.014)	0.73308

Table S5.8: splitting of LDL. Inconsistency test between direct and indirect treatment comparisons in mixed treatment comparison.

Comparison	Direct		Indirect		Difference		
	Coef.	CrI	Coef.	CrI	Coef.	CrI	P>|z|
Dulaglutide v.s. Semaglutide	-0.46	(-0.75,-0.16)	-0.12	(-0.31,0.072)	-0.22	(-0.38,-0.051)	0.05502

Dulaglutide v.s. Placebo	-0.050	(-0.18,0.080)	-0.39	(-0.71,-0.075)	-0.098	(-0.23,0.028)	0.05070
Exenatide v.s. Liraglutide	0.040	(-0.27,0.35)	0.14	(-0.013,0.30)	0.12	(-0.012,0.25)	0.54554
Semaglutide v.s. Placebo	0.075	(-0.079,0.23)	0.14	(-0.013,0.30)	0.12	(-0.015,0.26)	0.29254
Exenatide v.s. Placebo	0.20	(0.065,0.33)	0.0081	(-0.21,0.22)	0.15	(0.029,0.26)	0.13395
PEG-Loxenatide v.s. Placebo	0.0047	(-0.22,0.23)	-0.0025	(-0.35,0.35)	0.0024	(-0.18,0.19)	0.96960
Liraglutide v.s. Placebo	-0.026	(-0.14,0.089)	0.21	(-0.016,0.43)	0.023	(-0.080,0.13)	0.06934
Semaglutide v.s. others	0.080	(-0.23,0.39)	0.18	(-0.031,0.40)	0.15	(-0.024,0.32)	0.57907
Exenatide v.s. others	0.085	(-0.13,0.30)	0.27	(0.055,0.47)	0.18	(0.026,0.33)	0.23165
PEG-Loxenatide v.s. others	0.030	(-0.28,0.34)	0.038	(-0.23,0.31)	0.035	(-0.17,0.24)	0.96696
Liraglutide v.s. others	0.19	(-0.025,0.40)	-0.060	(-0.26,0.14)	0.055	(-0.094,0.20)	0.09274

Table S5.9 splitting of TC. Inconsistency test between direct and indirect treatment comparisons in mixed treatment comparison.

Comparison	Direct		Indirect		Difference		
	Coef.	CrI	Coef.	CrI	Coef.	CrI	P>|z|
Exenatide v.s. Liraglutide	0.090	(-0.13,0.31)	0.16	(0.055,0.27)	0.15	(0.053,0.24)	0.54094
Semaglutide v.s. Placebo	0.070	(-0.041,0.18)	0.075	(-0.17,0.32)	0.071	(-0.028,0.17)	0.96797
Exenatide v.s. Placebo	0.30	(0.20,0.39)	0.11	(-0.031,0.26)	0.24	(0.16,0.33)	0.03965
PEG-Loxenatide v.s. Placebo	-0.025	(-0.18,0.13)	0.15	(-0.093,0.39)	0.025	(-0.11,0.15)	0.22667
Liraglutide v.s. Placebo	0.073	(-0.012,0.16)	0.17	(0.015,0.32)	0.095	(0.023,0.17)	0.26877
Semaglutide v.s. others	0.060	(-0.16,0.28)	0.055	(-0.100,0.21)	0.057	(-0.068,0.18)	0.97235
Exenatide v.s. others	0.13	(-0.018,0.28)	0.31	(0.18,0.45)	0.23	(0.13,0.33)	0.06961
PEG-Loxenatide v.s. others	0.11	(-0.11,0.33)	-0.062	(-0.25,0.12)	0.010	(-0.13,0.15)	0.22681

Liraglutide v.s. others	0.11	(-0.013,0.24)	0.037	(-0.11,0.18)	0.081	(-0.014,0.18)	0.42768

Table S5.10 splitting of TG. Inconsistency test between direct and indirect treatment comparisons in mixed treatment comparison.

Comparison	Direct		Indirect		Difference		
	Coef.	CrI	Coef.	CrI	Coef.	CrI	P>|z|
Dulaglutide v.s. Semaglutide	1.3	(1.1,1.5)	-0.047	(-0.18,0.086)	0.36	(0.097,0.61)	0
Dulaglutide v.s. Placebo	0.030	(-0.077,0.14)	1.3	(1.1,1.6)	0.25	(0.046,0.46)	0
Semaglutide v.s. Placebo	0.088	(-0.10,0.28)	-0.58	(-0.88,-0.28)	-0.10	(-0.30,0.094)	0.0007
Exenatide v.s. Placebo	0.031	(-0.24,0.30)	-0.12	(-0.53,0.29)	-0.015	(-0.24,0.21)	0.54263
PEG-Loxenatide v.s. Placebo	-0.16	(-0.48,0.17)	0.18	(-0.34,0.69)	-0.061	(-0.34,0.22)	0.27880
Liraglutide v.s. Placebo	0.11	(-0.079,0.29)	-0.061	(-0.47,0.35)	0.078	(-0.083,0.24)	0.45233
Placebo v.s. Orforglipron	-0.11	(-0.22,0.00015)	-1.3	(-1.7,-1.0)	-0.22	(-0.44,-0.0034)	0.0
Semaglutide v.s. others	0.050	(-0.42,0.52)	-0.16	(-0.47,0.16)	-0.091	(-0.35,0.17)	0.45880
Exenatide v.s. others	-0.070	(-0.40,0.26)	0.079	(-0.29,0.45)	-0.0030	(-0.25,0.24)	0.54392
PEG-Loxenatide v.s. others	0.14	(-0.32,0.60)	-0.19	(-0.59,0.21)	-0.049	(-0.35,0.25)	0.27893
Liraglutide v.s. others	0.023	(-0.25,0.29)	0.19	(-0.14,0.53)	0.089	(-0.12,0.30)	0.43085

Table S5.11 splitting of SBP. Inconsistency test between direct and indirect treatment comparisons in mixed treatment comparison.

Comparison	Direct		Indirect		Difference		
	Coef.	CrI	Coef.	CrI	Coef.	CrI	P>|z|
Dulaglutide v.s. Semaglutide	-0.27	(-2.5,1.9)	-1.1	(-2.5,0.19)	-0.91	(-2.0,0.22)	0.49469
Dulaglutide v.s. Exenatide	0.18	(-2.5,2.8)	-1.9	(-3.3,-0.44)	-1.4	(-2.7,-0.17)	0.17819
Dulaglutide v.s. Liraglutide	-1.2	(-3.9,1.4)	-1.7	(-3.0,-0.33)	-1.6	(-2.8,-0.40)	0.77333
Dulaglutide v.s. Placebo	1.6	(0.29,2.8)	2.2	(0.71,3.6)	1.9	(0.95,2.8)	0.51573

Dulaglutide v.s. others	1.3	(0.054,2.6)	1.5	(0.073,3.0)	1.4	(0.46,2.4)	0.80966
Exenatide v.s. Liraglutide	0.98	(-2.8,4.7)	-0.33	(-1.8,1.1)	-0.16	(-1.5,1.2)	0.51763
Semaglutide v.s. Placebo	2.7	(1.2,4.2)	2.9	(1.3,4.5)	2.8	(1.7,3.9)	0.85941
Exenatide v.s. Placebo	3.2	(2.,4.3)	4.7	(2.3,7.0)	3.3	(2.3,4.4)	0.24868
PEG-Loxenatide v.s. Placebo	0.14	(-2.5,2.8)	2.3	(-1.6,6.2)	0.81	(-1.4,3.0)	0.36264
Liraglutide v.s. Placebo	4.4	(3.1,5.7)	2.	(0.34,3.6)	3.5	(2.4,4.5)	0.02103
Placebo v.s. Orforglipron	-8.5	(-10,-6.6)	-8.	(-12.,-3.9)	-8.5	(-10,-6.9)	0.79389
Semaglutide v.s. others	2.7	(1.2,4.1)	1.8	(0.20,3.5)	2.3	(1.3,3.4)	0.44733
Exenatide v.s. Others	4.2	(1.6,6.8)	2.4	(0.90,3.9)	2.8	(1.5,4.1)	0.23752
PEG-Loxenatide v.s. others	1.7	(-2.1,5.5)	-0.45	(-3.3,2.4)	0.33	(-1.9,2.6)	0.36570
Liraglutide v.s. Others	1.6	(-0.24,3.4)	4.	(2.5,5.5)	3.	(1.8,4.2)	0.04896

Table S5.12 splitting of DBP. Inconsistency test between direct and indirect treatment comparisons in mixed treatment comparison.

Comparison	Direct		Indirect		Difference		
	Coef.	CrI	Coef.	CrI	Coef.	CrI	P>|z|
Dulaglutide v.s. Semaglutide	-0.27	(-2.4,1.9)	-1.1	(-2.5,0.18)	-0.91	(-2.0,0.22)	0.49349
Dulaglutide v.s. Exenatide	0.18	(-2.5,2.8)	-1.9	(-3.3,-0.44)	-1.4	(-2.7,-0.17)	0.17895
Dulaglutide v.s. Liraglutide	-1.3	(-3.9,1.4)	-1.7	(-3.0,-0.34)	-1.6	(-2.8,-0.40)	0.77326
Dulaglutide v.s. Placebo	1.5	(0.28,2.8)	2.2	(0.72,3.6)	1.9	(0.95,2.8)	0.51268
Dulaglutide v.s. others	1.3	(0.057,2.6)	1.5	(0.075,3.0)	1.4	(0.46,2.4)	0.80265
Exenatide v.s. Liraglutide	0.96	(-2.8,4.7)	-0.33	(-1.8,1.1)	-0.16	(-1.5,1.2)	0.52311
Semaglutide v.s. Placebo	2.7	(1.2,4.2)	2.9	(1.3,4.5)	2.8	(1.7,3.9)	0.86125
Exenatide v.s. Placebo	3.2	(2.,4.3)	4.7	(2.3,7.0)	3.3	(2.3,4.4)	0.25012

PEG-Loxenatide v.s. Placebo	0.13	(-2.5,2.8)	2.3	(-1.6,6.2)	0.81	(-1.4,3.)	0.36488
Liraglutide v.s. Placebo	4.4	(3.1,5.7)	2.	(0.35,3.6)	3.5	(2.4,4.5)	0.02130
Placebo v.s. Orforglipron	-8.6	(-10,-6.6)	-8	(-12,-3.9)	-8.5	(-10,-6.9)	0.79044
Semaglutide v.s. others	2.7	(1.3,4.1)	1.8	(0.22,3.5)	2.3	(1.2,3.4)	0.44617
Exenatide v.s. Others	4.2	(1.6,6.8)	2.4	(0.91,3.9)	2.8	(1.5,4.1)	0.24142
PEG-Loxenatide v.s. others	1.7	(-2.1,5.4)	-0.46	(-3.3,2.4)	0.33	(-1.9,2.6)	0.36487
Liraglutide v.s. Others	1.6	(-0.23,3.4)	4	(2.5,5.5)	3	(1.8,4.2)	0.04877

Supplementary 6: Network maps and forest plots of secondary outcome

Abbreviations: HDL, high density lipoprotein; LDL, low-density lipoprotein; TC, total cholesterol; TG, triglyceride; SBP, systolic blood pressure; DBP, diastolic blood pressure, ACM, all-cause mortality; NFS, non-fatal stroke; NFM, non-fatal myocardial infarction; CVM, cardiovascular mortality; MACE, major adverse cardiovascular events; GFR, glomerular filtration rate; UACR, urinary albumin/creatinine ratio; FIL, fasting insulin level; HOMA-β, homeostasis model assessment of β-cell function; HOMA-IR, homeostasis model assessment of insulin resistance.

Figure S6.1: Network map of the effect on the proportion of patients whose HbA_{1c} reaches the target of 7.0%, and forest plot of network effect sizes for compared with placebo.

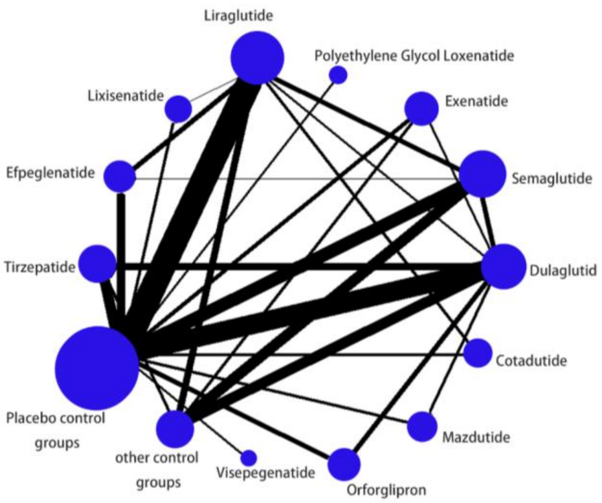

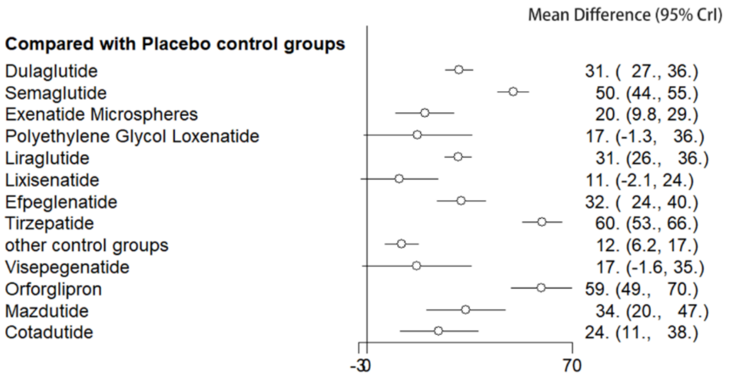

Figure S6.2: Network map of the effect on the proportion of patients whose HbA_{1c} reaches the target of 7.0%, and forest plot of network effect sizes for compared with placebo.

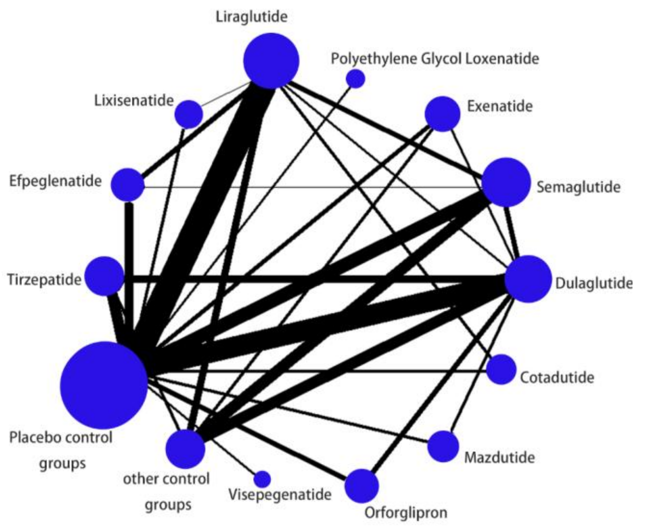

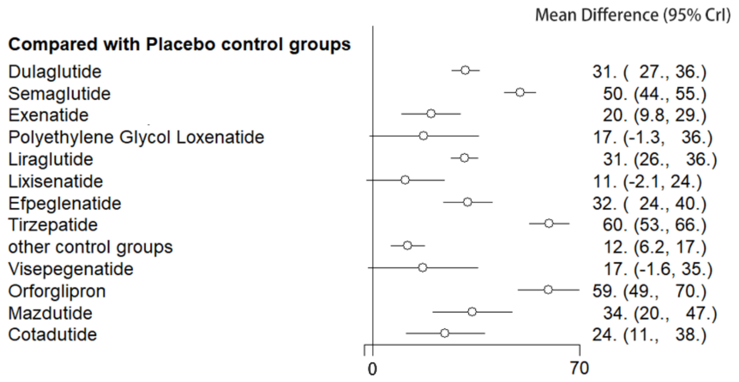

Figure S6.3: Network map of the effect on HDL, and forest plot of network effect sizes for compared with placebo.

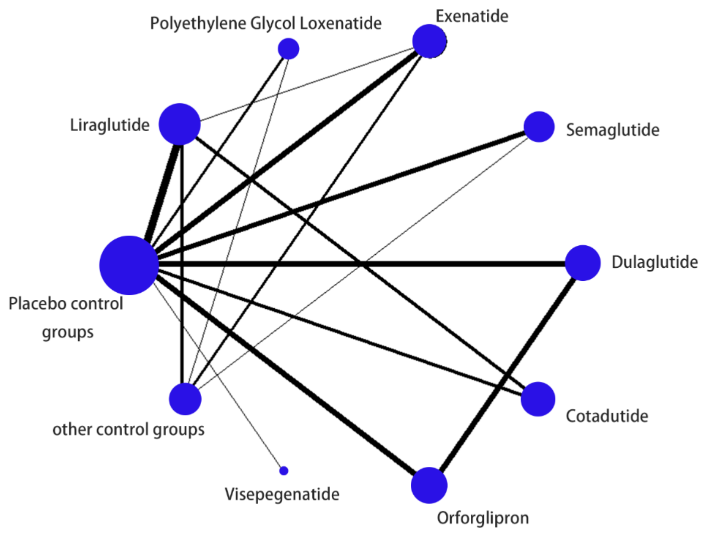

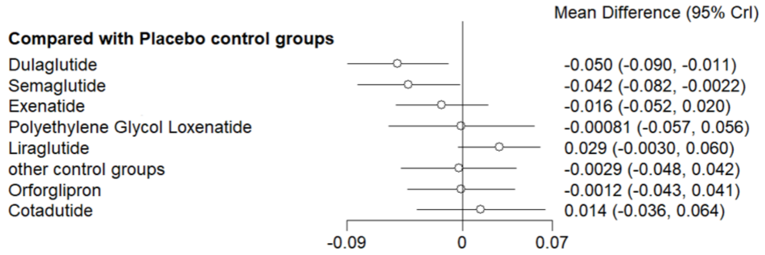

Figure S6.4: Network map of the effect on LDL, and forest plot of network effect sizes for compared with placebo.

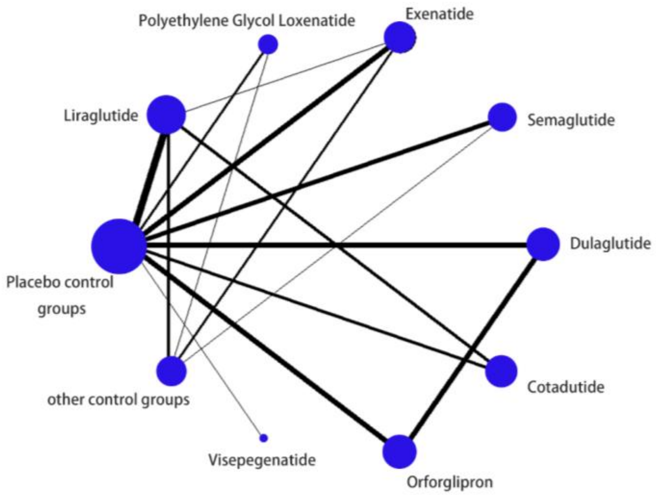

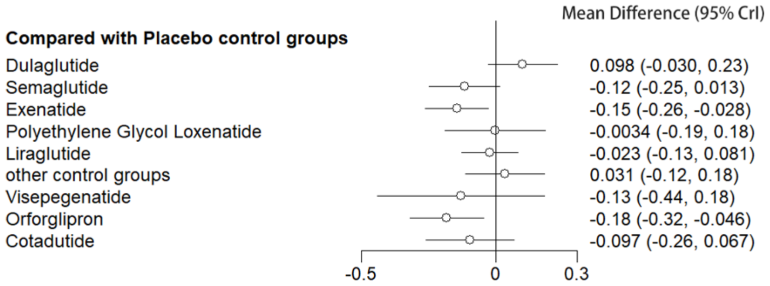

Figure S6.5: Network map of the effect on TC, and forest plot of network effect sizes for compared with placebo.

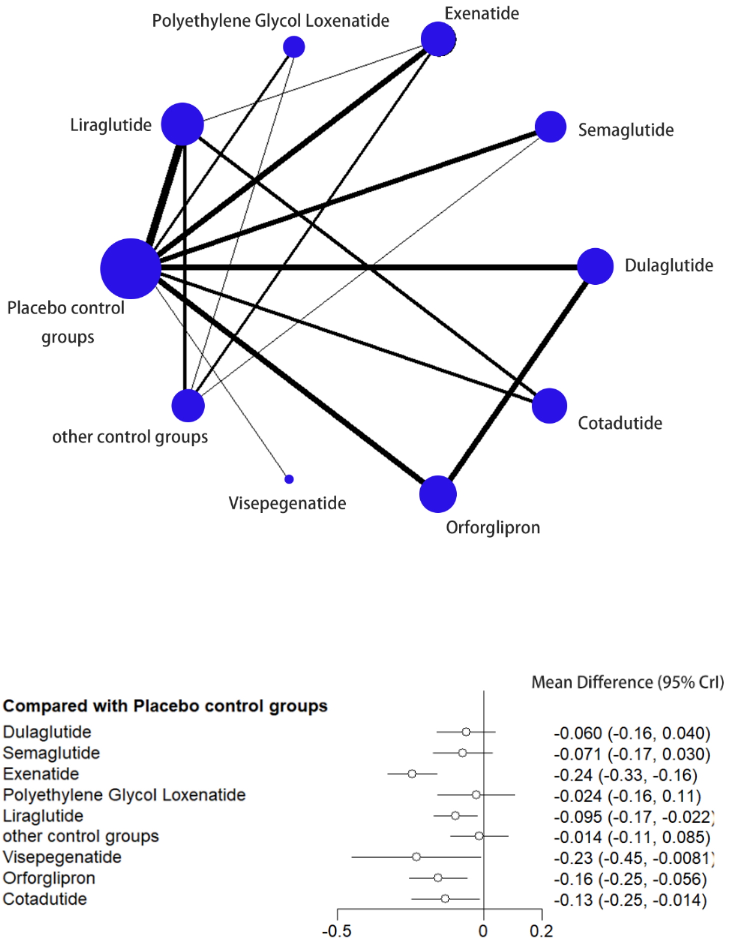

Figure S6.6: Network map of the effect on TG, and forest plot of network effect sizes for compared with placebo.

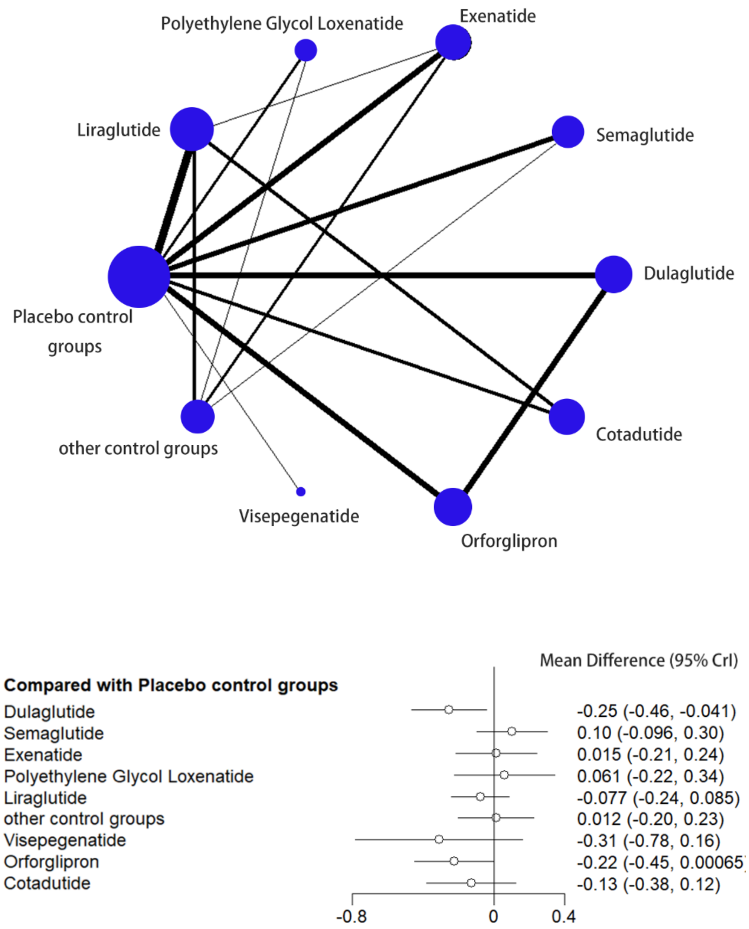

Figure S6.7: Network map of the effect on SBP, and forest plot of network effect sizes for compared with placebo.

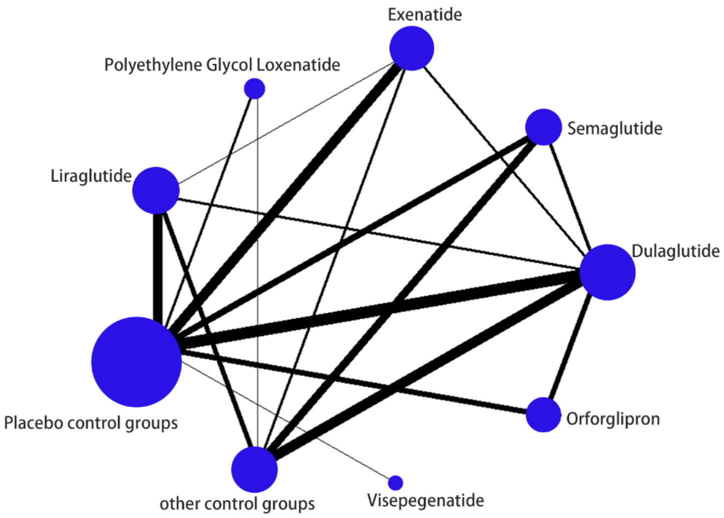

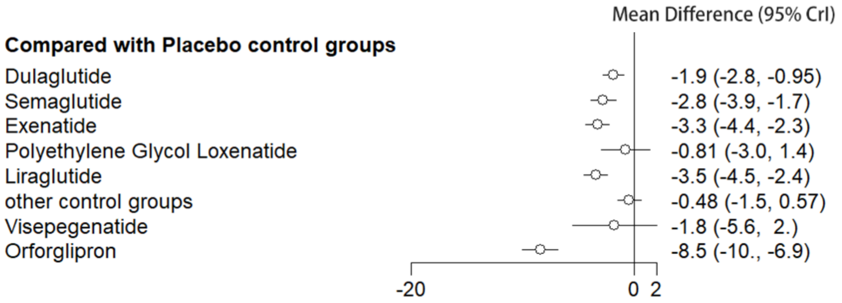

Figure S6.8: Network map of the effect on DBP and forest plot of network effect sizes for compared with placebo.

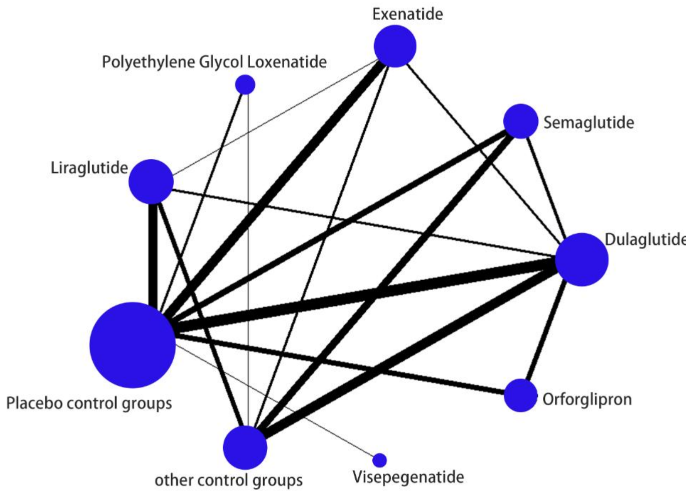

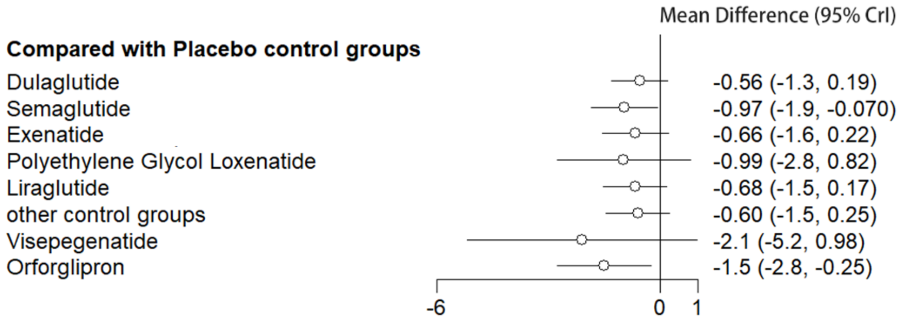

Figure S6.9: Network map of the effect on MACE, and forest plot of network effect sizes for compared with placebo.

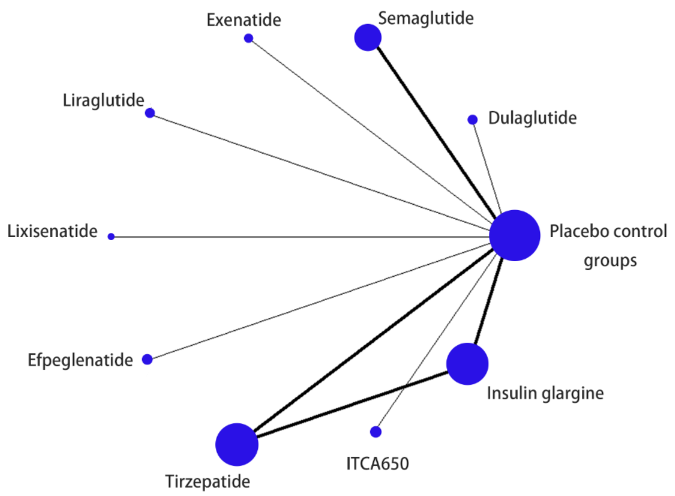

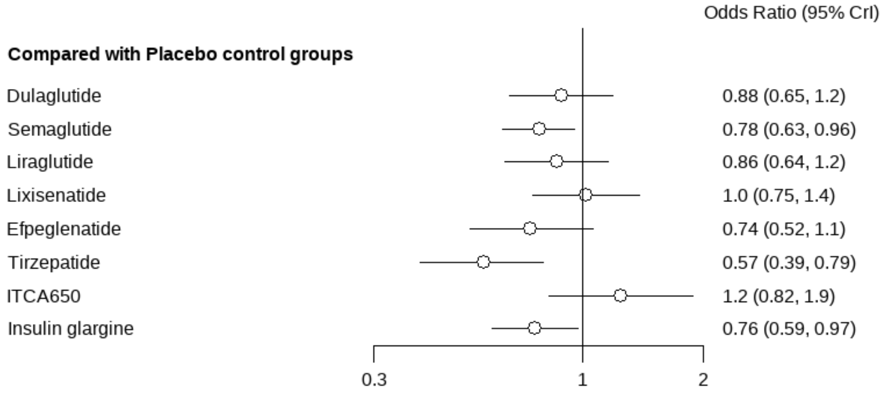

Figure S6.10: Network map of the effect on NFS, and forest plot of network effect sizes for compared with placebo.

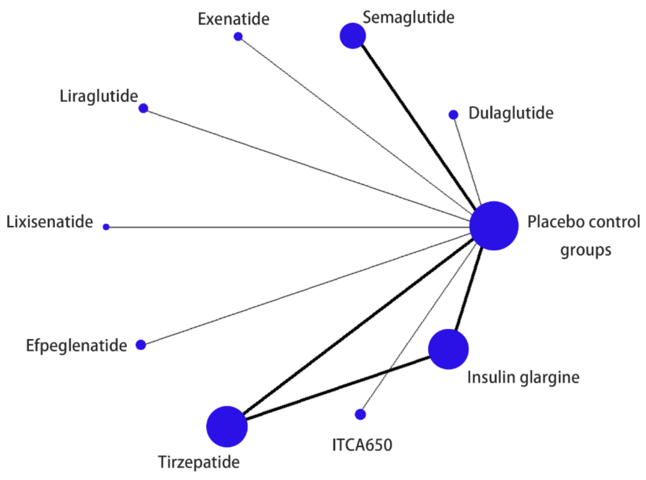

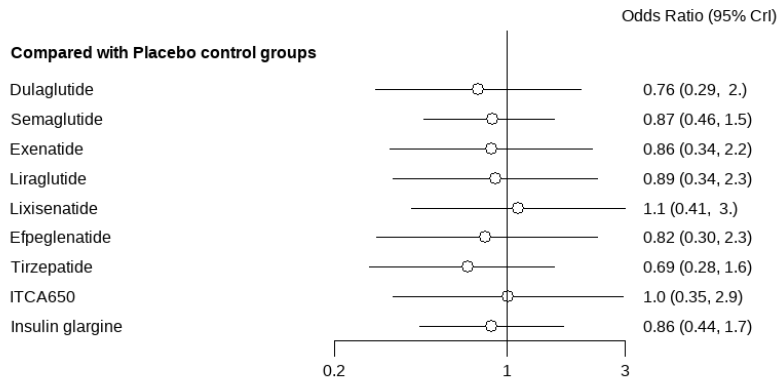

Figure S6.11 Network map of the effect on non-fatal myocardial infarction, and forest plot of network effect sizes for compared with placebo.

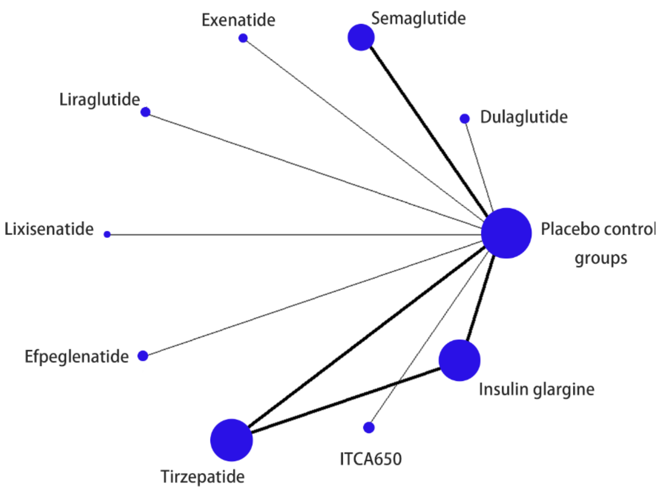

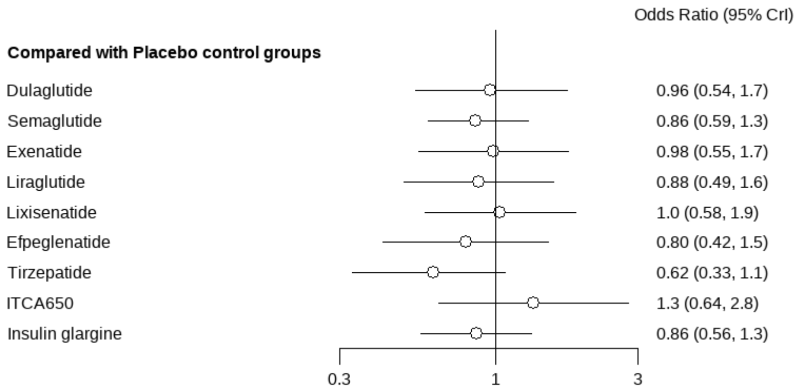

Figure S6.12: Network map of the effect on CVM, and forest plot of network effect sizes for compared with placebo.

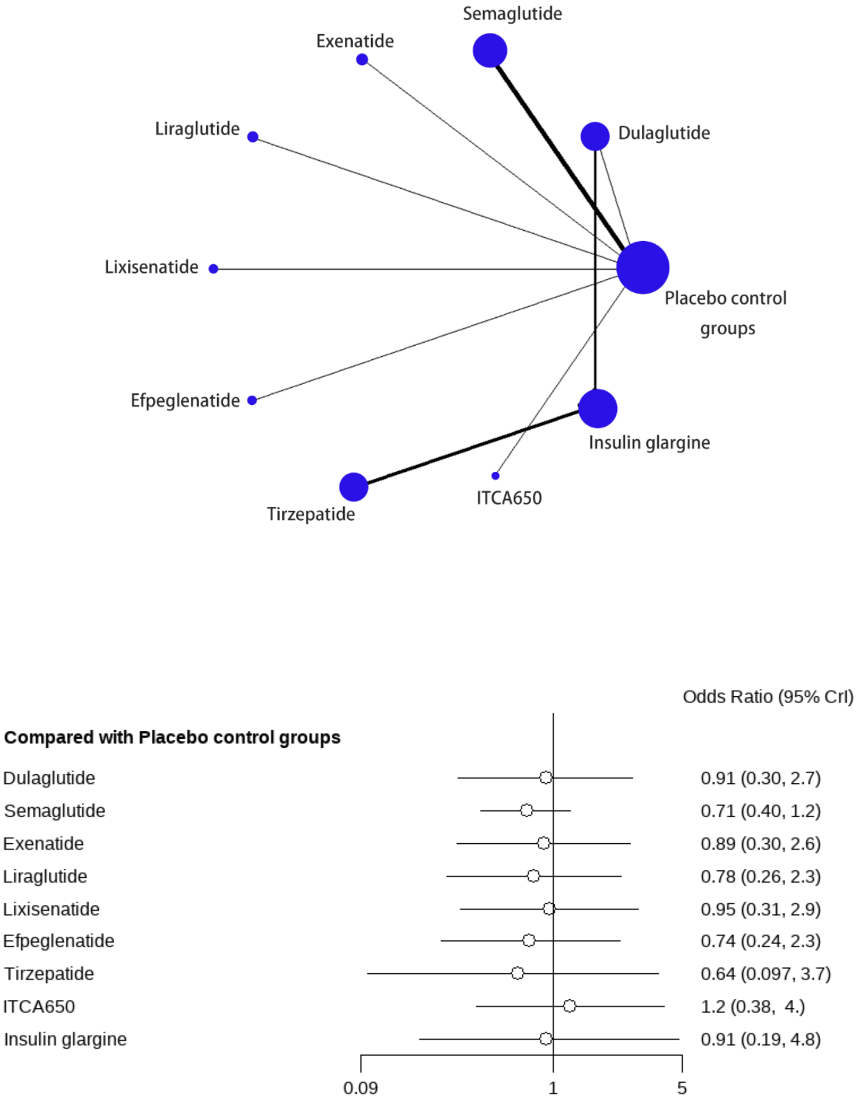

Figure S6.13 Network map of the effect on ACM, and forest plot of network effect sizes for compared with placebo.

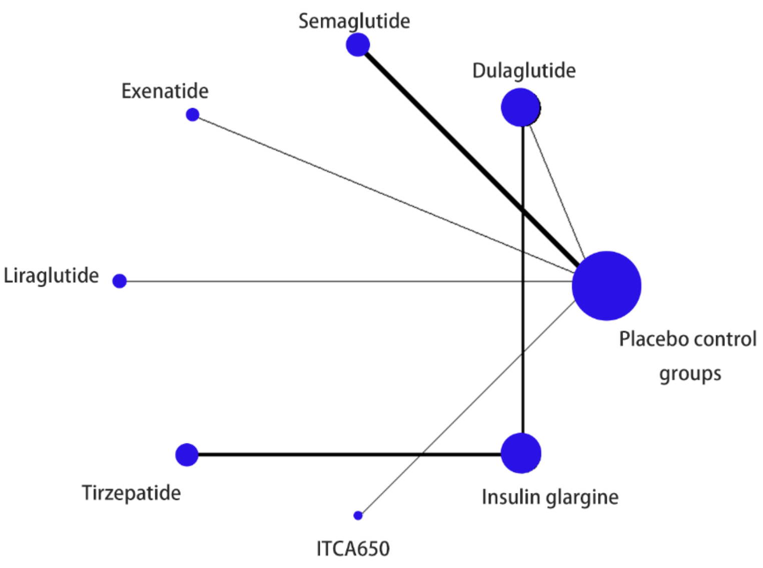

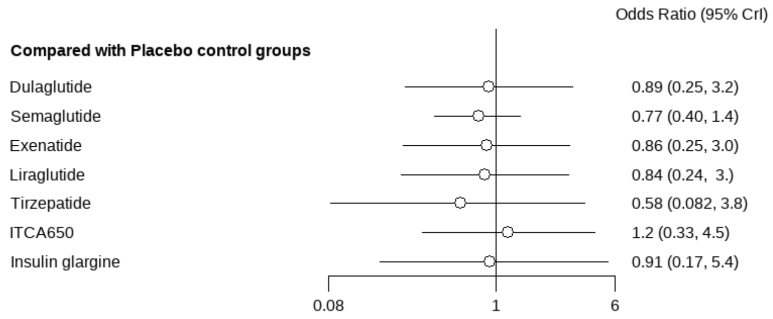

Figure S6.14 Network map of the effect on eGFR, and forest plot of network effect sizes for compared with placebo.

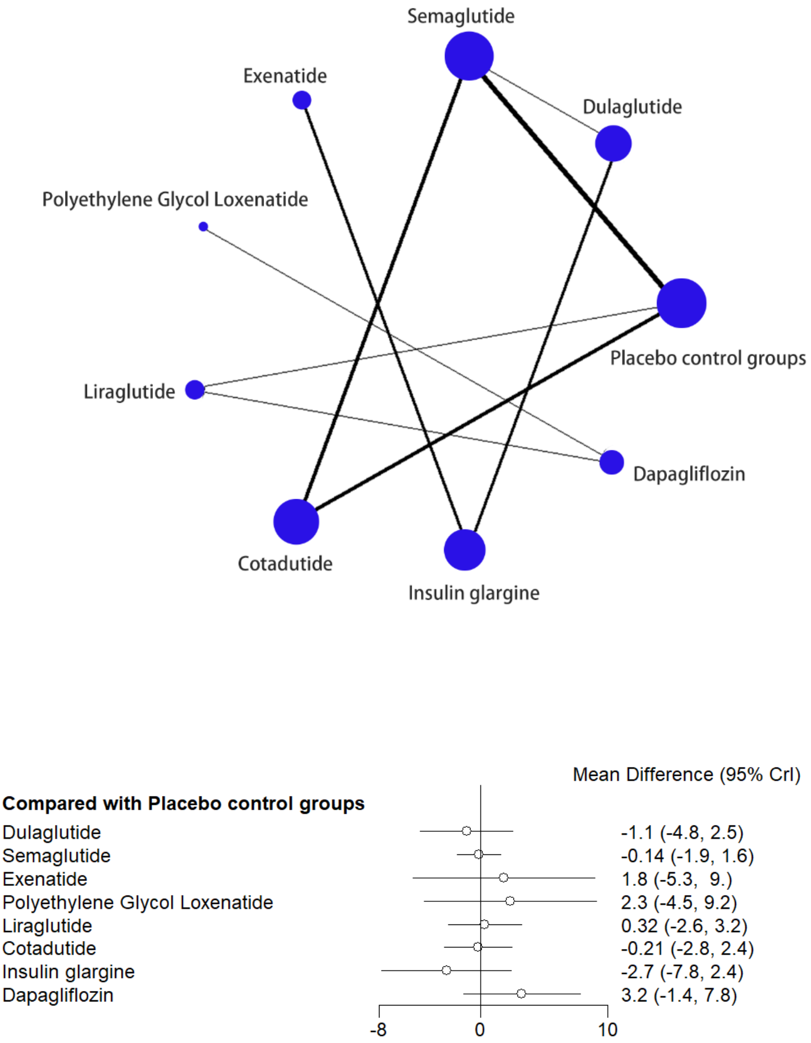

Figure S6.15 Network map of the effect on UACR, and forest plot of network effect sizes for compared with placebo.

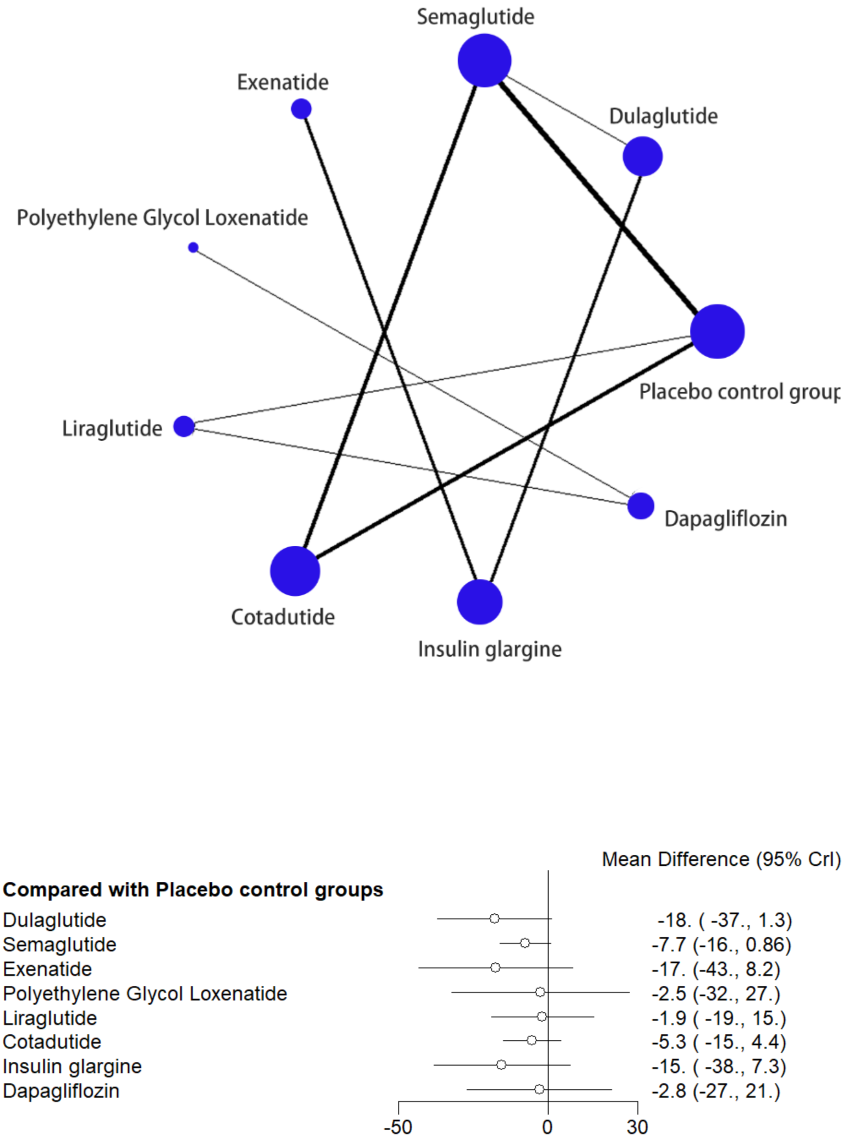

Figure S6.16 Network map of the effect on FIS, and forest plot of network effect sizes for compared with placebo.

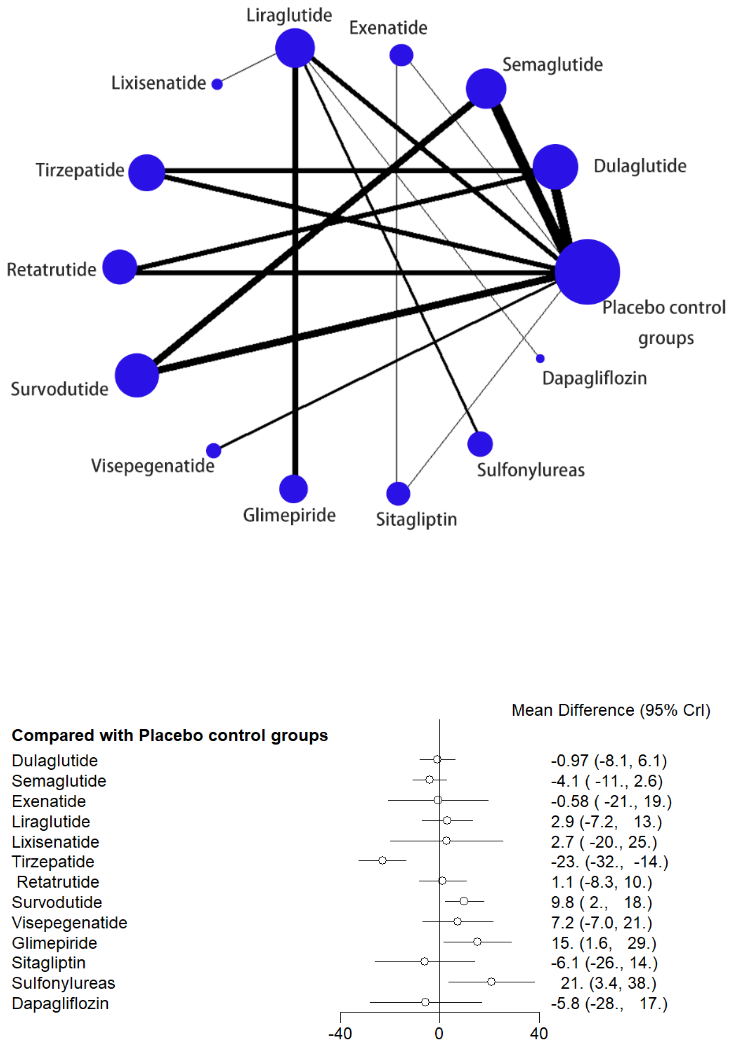

Figure S6.17 Network map of the effect on C-peptide, and forest plot of network effect sizes for compared with placebo.

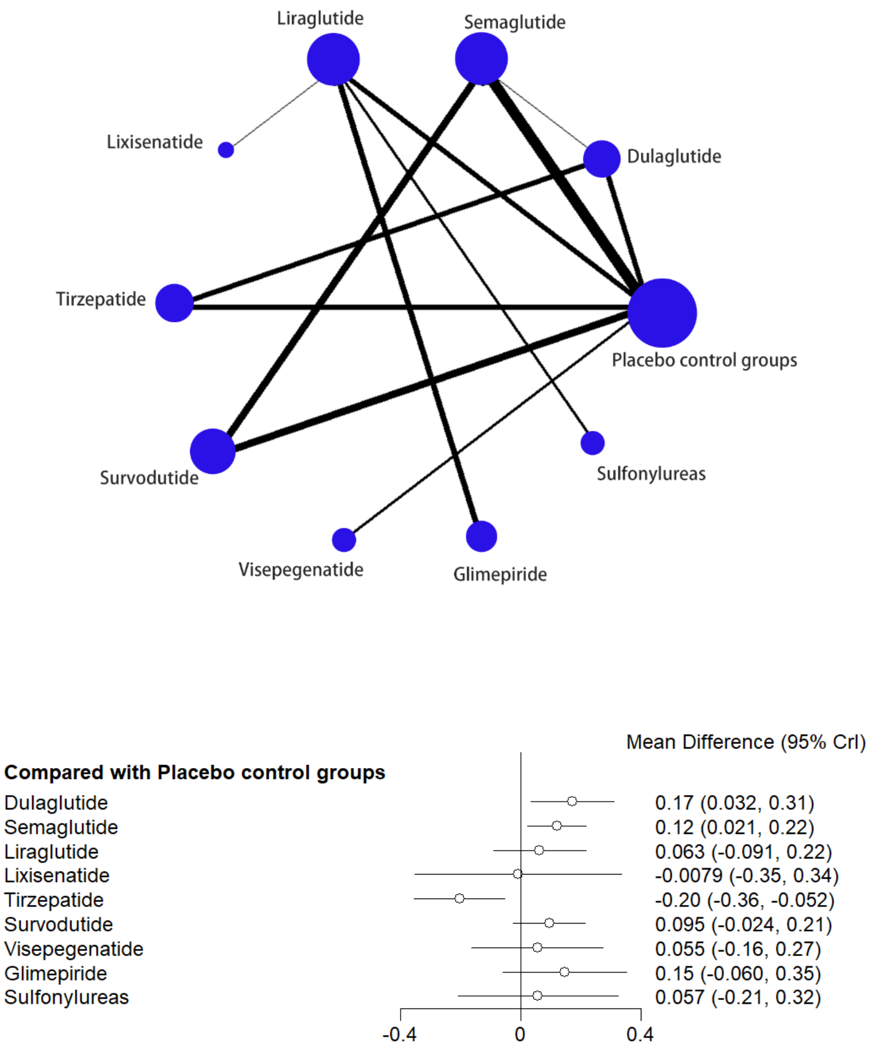

Figure S6.18 Network map of the effect on HOMA-IR, and forest plot of network effect sizes for compared with placebo.

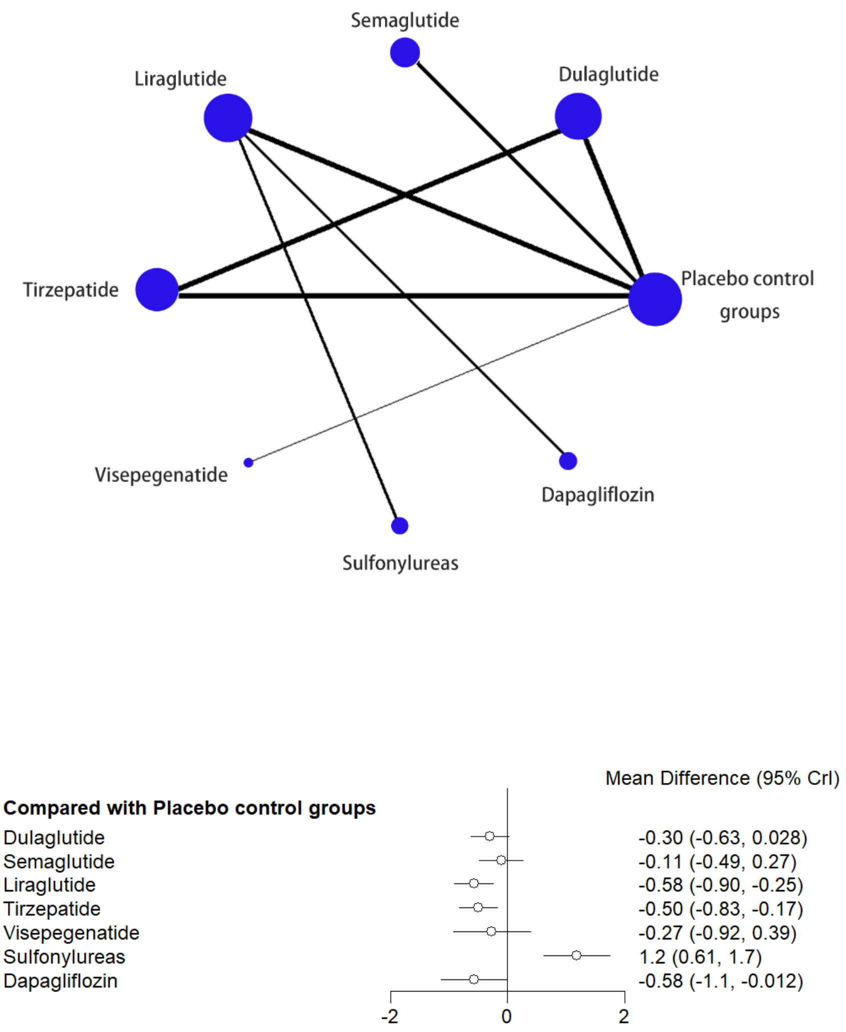

Figure S6.19 Network map of the effect on HOMA-β, and forest plot of network effect sizes for compared with placebo.

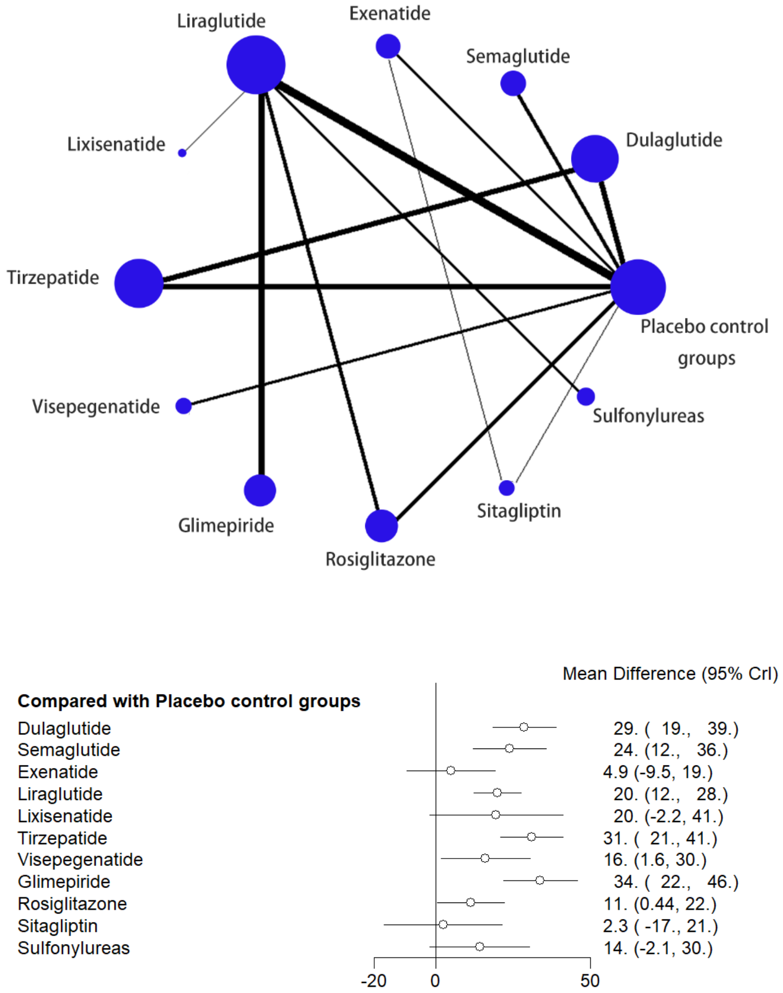

Figure S6.20 Network map of the effect on nausea, and forest plot of network effect sizes for compared with placebo.

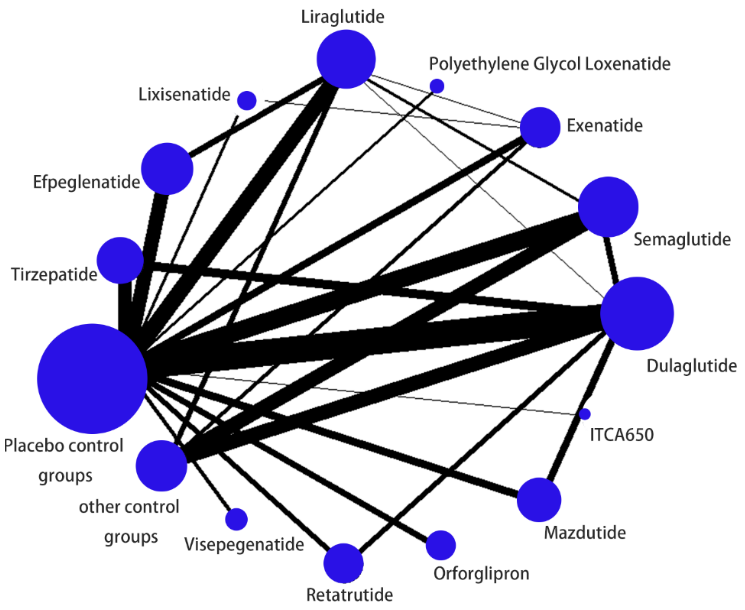

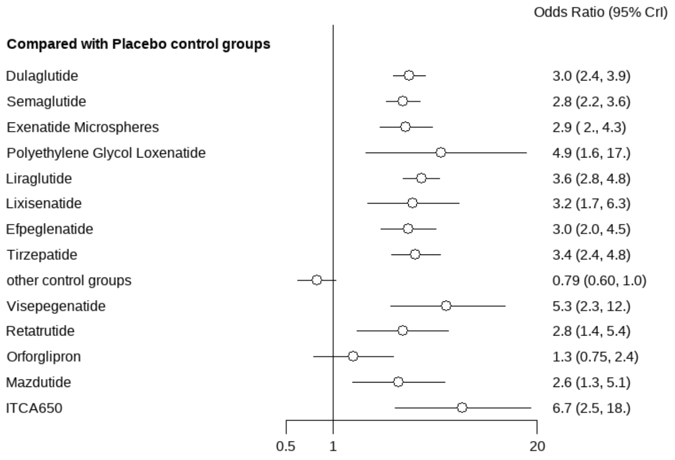

Figure S6.21 Network map of the effect on vomiting, and forest plot of network effect sizes for compared with placebo.

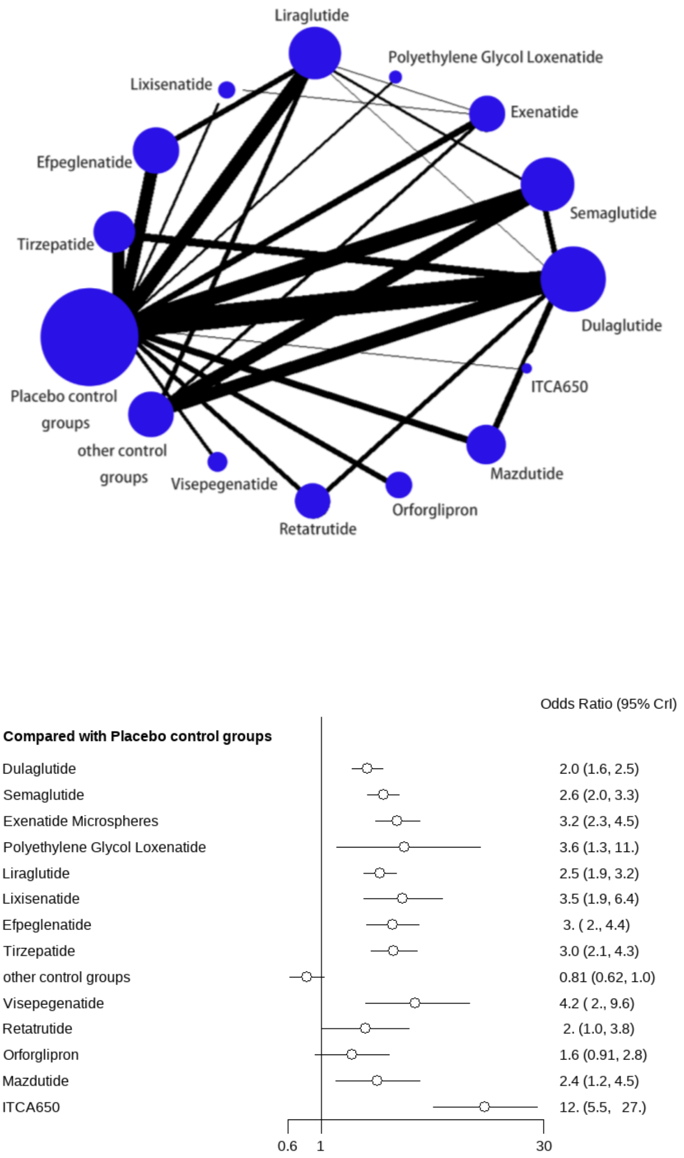

Figure S6.22 Network map of the effect on diarrhea, and forest plot of network effect sizes for compared with placebo.

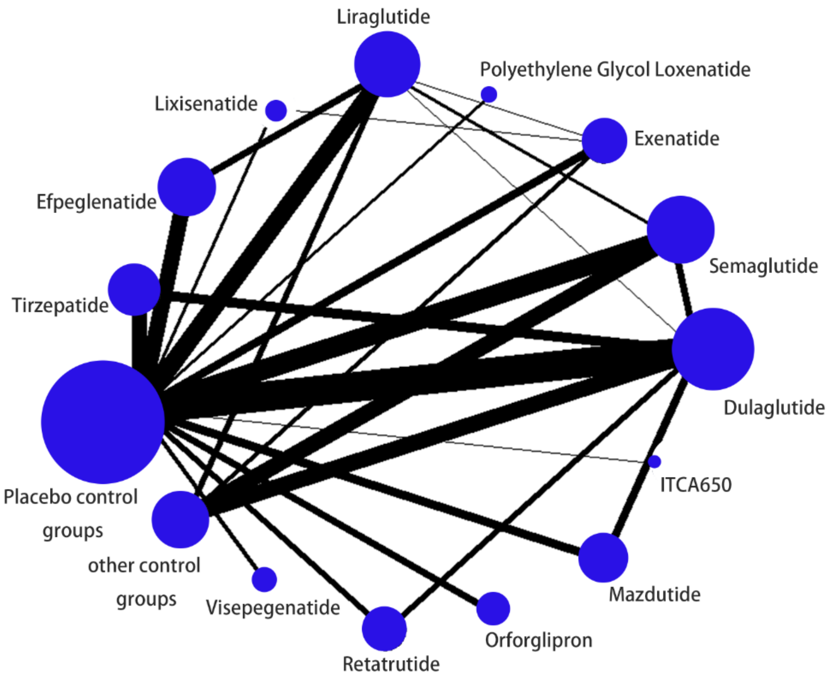

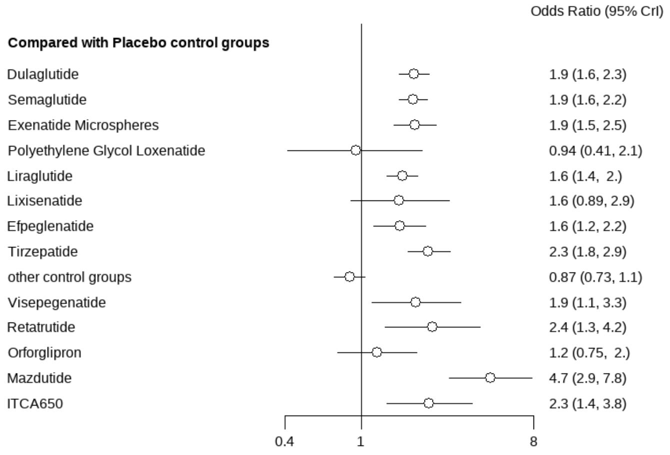

Figure S6.23 Network map of the effect on constipation, and forest plot of network effect sizes for compared with placebo.

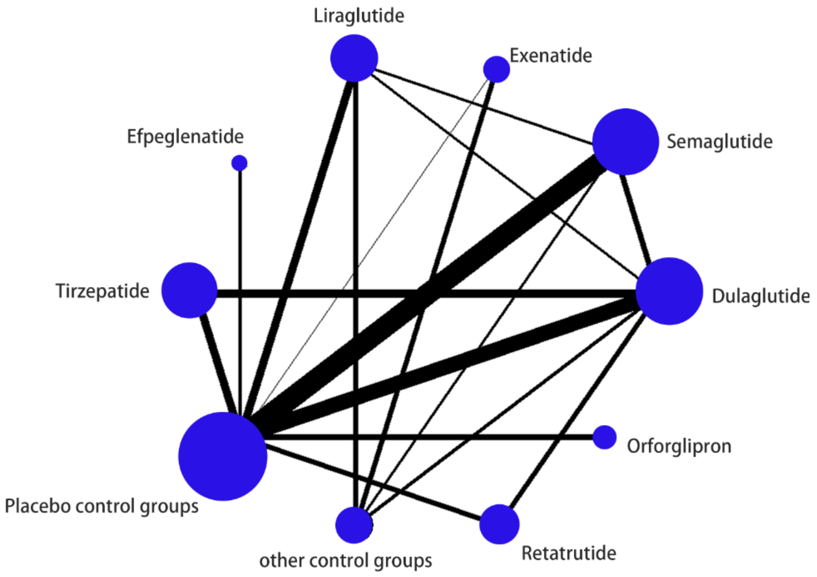
Liraglutide
Exenatide
Efpeglenatide
Semaglutide
Tirzepatide
Dulaglutide
Orforglipron
Placebo control groups
other control groups
Retatrutide

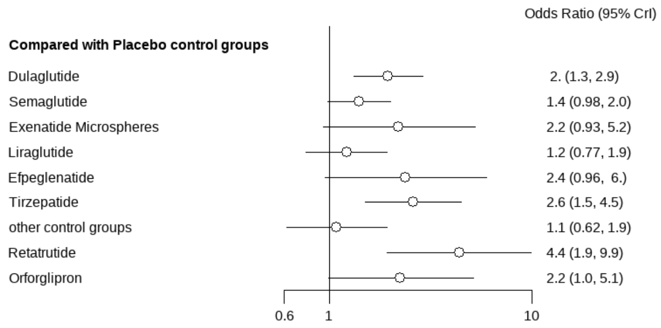
Odds Ratio (95% CrI)
Compared with Placebo control groups
Dulaglutide 2. (1.3, 2.9)
Semaglutide 1.4 (0.98, 2.0)
Exenatide Microspheres 2.2 (0.93, 5.2)
Liraglutide 1.2 (0.77, 1.9)
Efpeglenatide 2.4 (0.96, 6.)
Tirzepatide 2.6 (1.5, 4.5)
other control groups 1.1 (0.62, 1.9)
Retatrutide 4.4 (1.9, 9.9)
Orforglipron 2.2 (1.0, 5.1)
0.6
1
10

SUCRA of the effects of various GLP-1RAs

Supplementary 7: SUCRA and stacked sort charts

Abbreviations: FPG, fasting plasma glucose; HDL, highdensity lipoprotein; LDL, low-density lipoprotein; TC, total cholesterol; TG, triglyceride; SBP, systolic blood pressure; DBP, diastolic blood pressure, ACM, all-cause mortality; NFS, non-fatal stroke; NFM, non-fatal myocardial infarction; CVM, cardiovascular mortality; MACE, major adverse cardiovascular events; GFR, glomerular filtration rate; UACR, urinary albumin/creatinine ratio; FIL, fasting insulin level; HOMA-β, homeostasis model assessment of β-cell function; HOMA-IR, homeostasis model assessment of insulin resistance.

Figure S7.1 Stacked sort charts of GLP-1RAs for HbA_{1c} in network analysis.

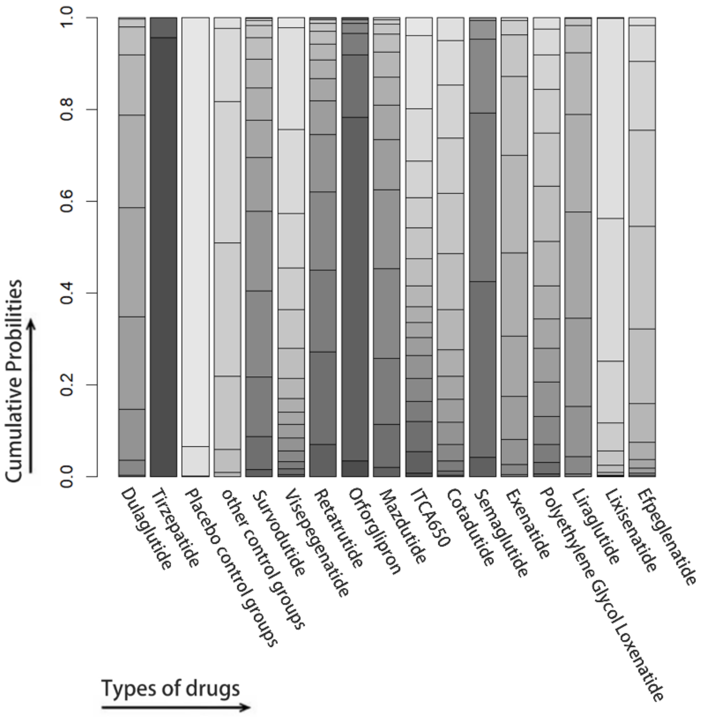

Table S7.1 SUCRA of the effects of various GLP-1RAs on HbA_{1c}**.**

Treatment	SUCRA
Dulaglutide	55.05
Semaglutide	82.54
Exenatide	47.57
Polyethylene Glycol Loxenatide	44.49
Liraglutide	55.13
Lixisenatide	12.74

Efpeglenatid	30.01
Tirzepatide	99.72
Placebo control groups	0.41
other control groups	22.42
Survodutide	65.37
Visepegenatide	26.62
Retatrutide	72.81
Orforglipron	91.71
Mazdutide	67.16
ITCA650	39.37
Cotadutide	36.89

Figure S7.2 Stacked sort charts of GLP-1RAs for FPG in network analysis.

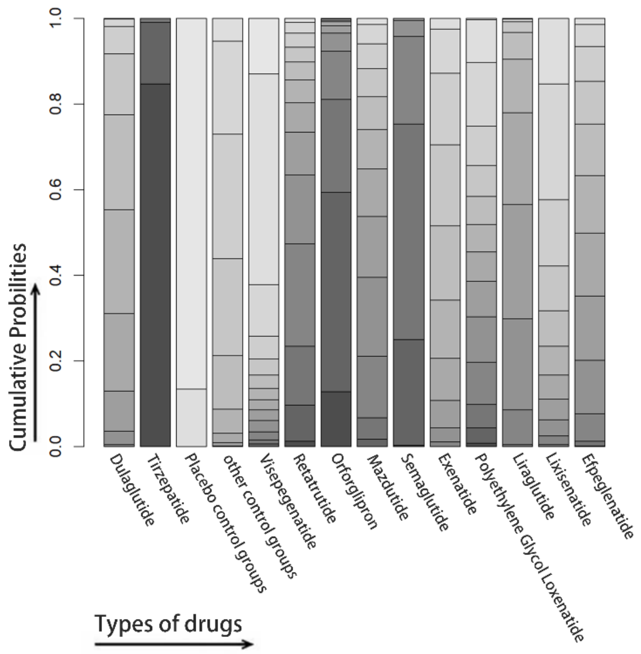

Table S7.2: SUCRA of the effects of various GLP-1RAs on FPG.

Treatment	SUCRA
Dulaglutide	43.78
Semaglutide	84.32
Exenatide	36.77

Polyethylene Glycol Loxenatide	45.37
Liraglutide	58.44
Lixisenatide	28.95
Efpeglenatid	48.47
Tirzepatide	98.73
Placebo control groups	1.07
other control groups	26.60
Visepegenatide	17.86
Retatrutide	66.36
Orforglipron	87.54
Mazdutide	55.74

Figure S7.3: Stacked sort charts of GLP-1RAs for weight loss in network analysis.

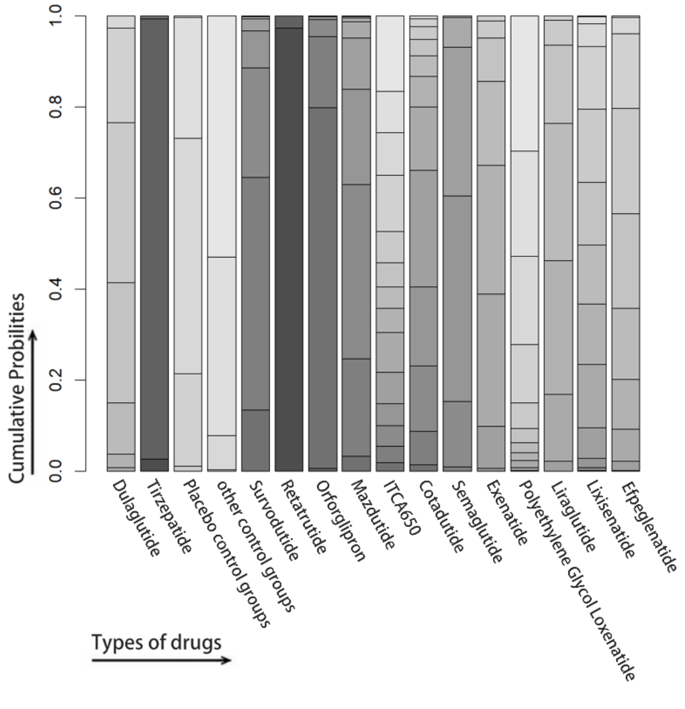

Table S7.3: SUCRA of the effects of various GLP-1RAs on body weight loss.

**Treatment**	**SUCRA**
Dulaglutide	28.59
Semaglutide	64.66

Exenatide	46.29
Polyethylene Glycol Loxenatide	14.98
Liraglutide	42.12
Lixisenatide	36.85
Efpeglenatide	32.99
Tirzepatide	93.47
Placebo control groups	12.42
other control groups	3.18
Survodutide	77.52
Retatrutide	99.81
Orforglipron	84.98
Mazdutide	71.14
ITCA650	31.77

Figure S7.4: Stacked sort charts of GLP-1RAs for the proportion of patients achieving the HbA_{1c} target of 7.0% in network analysis.

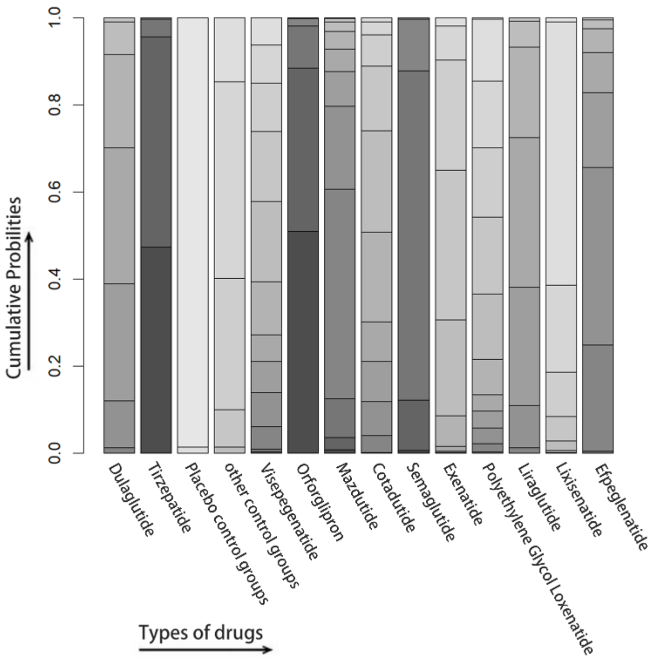

Table S7.4: SUCRA of the effects of various GLP-1RAs on the proportion of patients achieving the HbA_{1c} target of 7.0%.

Treatment	SUCRA
Dulaglutide	56.24
Semaglutide	86.17
Exenatide	31.65
Polyethylene Glycol Loxenatide	32.02
Liraglutide	56.45
Lixisenatide	14.08
Efpeglenatid	67.62
Tirzepatide	95.64
Placebo control groups	0.62
other control groups	19.58
Visepegenatide	41.38
Orforglipron	96.03
Mazdutide	73.21
Cotadutide	45.75

Figure S7.5: Stacked sort charts of GLP-1RAs for the proportion of patients achieving the HbA_{1c} target of 6.5% in network analysis.

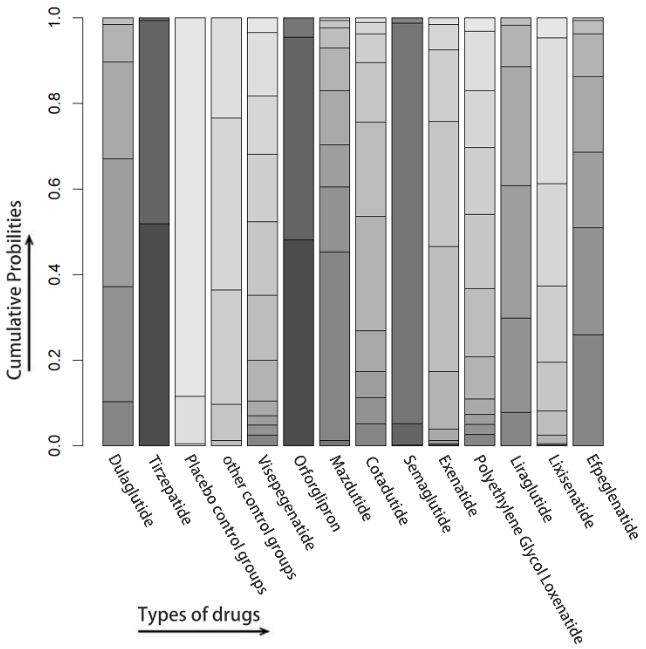

Table S7.5: SUCRA of the effects of various GLP-1RAs on the proportion of patients achieving the HbA_{1c} target of 6.5%.

Treatment	SUCRA
Dulaglutide	59.21
Semaglutide	85.21
Exenatide	31.44
Polyethylene Glycol Loxenatide	28.01
Liraglutide	57.74
Lixisenatide	16.09
Efpeglenatid	61.18
Tirzepatide	96.41
Placebo control groups	0.87
other control groups	16.02
Visepegenatide	27.51
Orforglipron	95.80
Mazdutide	63.36
Cotadutide	41.87

Figure S7.6: Stacked sort charts of GLP-1RAs for HDL in network analysis.

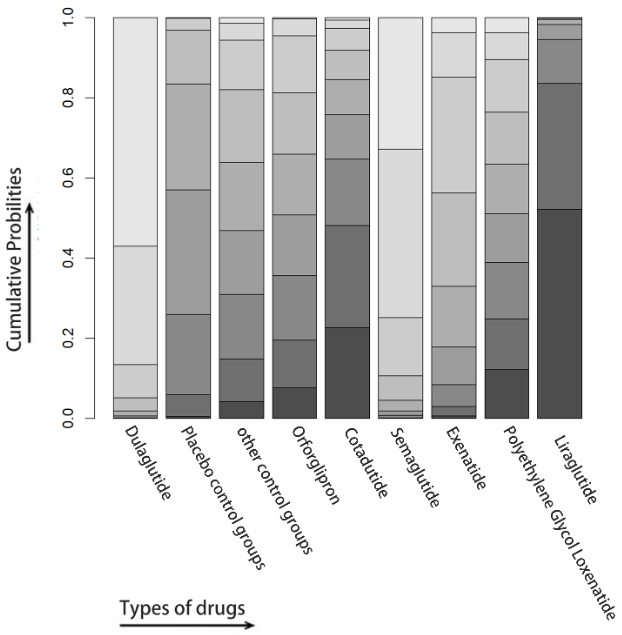

Table S7.6: SUCRA of the effects of various GLP-1RAs on HDL.

Treatment	SUCRA
Dulaglutide	8.07
Semaglutide	13.73
Exenatide	37.60
Polyethylene Glycol Loxenatide	56.45
Liraglutide	90.96
Placebo control groups	58.69
other control groups	54.43
Orforglipron	57.10
Cotadutide	72.98

Figure S7.7: Stacked sort charts of GLP-1RAs for LDL in network analysis.

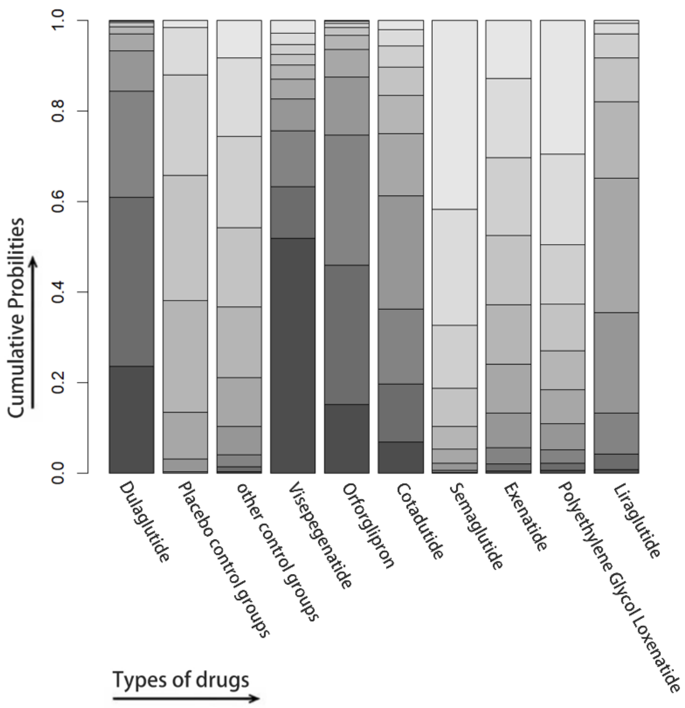

Table S7.7: SUCRA of the effects of various GLP-1RAs on LDL.

Treatment	SUCRA
**Dulaglutide**	7.65
**Semaglutide**	69.95
**Exenatide**	78.34
**Polyethylene Glycol Loxenatide**	34.79
**Liraglutide**	39.84
**Placebo control groups**	30.97
**other control groups**	22.79
**Visepegenatide**	66.55
**Orforglipron**	85.93
**Cotadutide**	63.19

Figure S7.8: Stacked sort charts of GLP-1RAs for TC in network analysis.

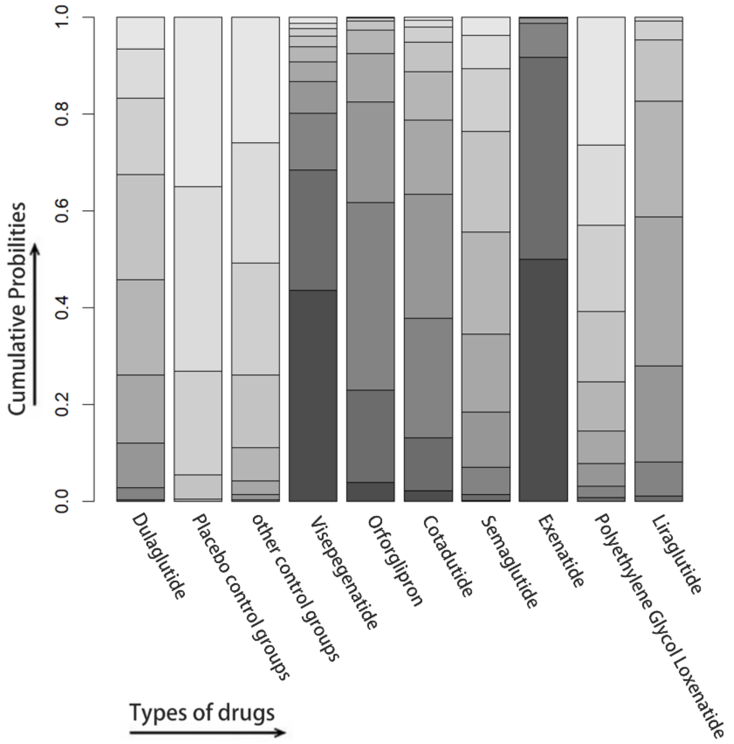

Table S7.8: SUCRA of the effects of various GLP-1RAs on TC.

Treatment	SUCRA
Dulaglutide	36.89
Semaglutide	42.07
Exenatide	93.33
Polyethylene Glycol Loxenatide	24.48
Liraglutide	52.52
Placebo control groups	10.88
other control groups	18.54
Visepegenatide	83.91
Orforglipron	73.37
Cotadutide	64.02

Figure S7.9: Stacked sort charts of GLP-1RAs for TG in network analysis.

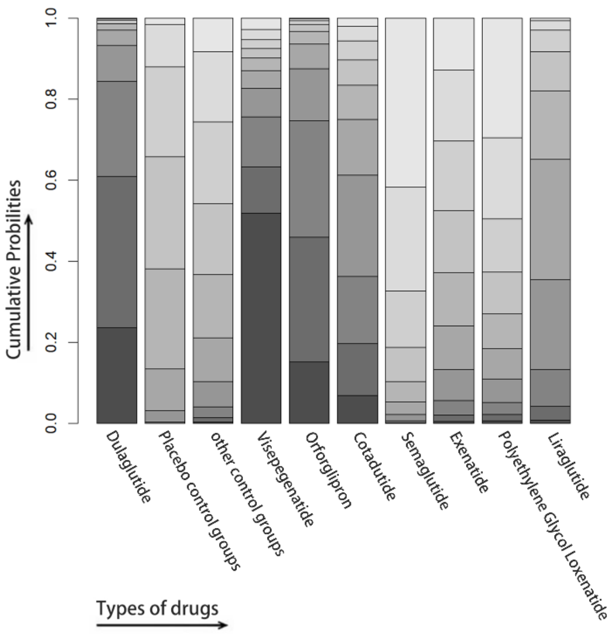

Table S7.9: SUCRA of the effects of various GLP-1RAs on TG.

Treatment	SUCRA
Dulaglutide	84.07
Semaglutide	14.30
Exenatide	32.36
Polyethylene Glycol Loxenatide	24.87
Liraglutide	54.38
Placebo control groups	34.06
other control groups	32.67
Visepegenatide	81.65
Orforglipron	78.97
Cotadutide	62.67

Figure S7.10: Stacked sort charts of GLP-1RAs for SBP in network analysis.

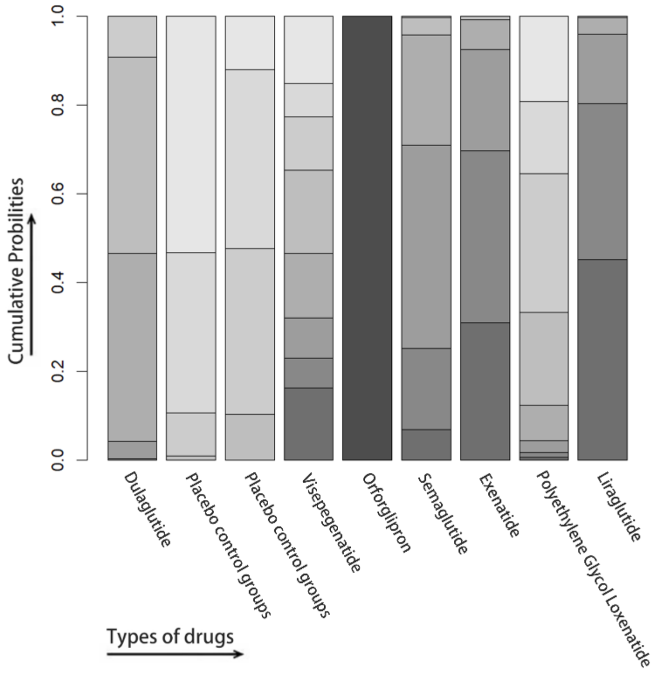

Table S7.10: SUCRA of the effects of various GLP-1RAs on SBP.

Treatment	SUCRA
Dulaglutide	42.67
Semaglutide	62.26
Exenatide	73.99
Polyethylene Glycol Loxenatide	24.72
Liraglutide	77.66
Placebo control groups	7.32
other control groups	18.14
Visepegenatide	43.27
Orforglipron	99.99

Figure S7.11: Stacked sort charts of GLP-1RAs for DBP in network analysis.

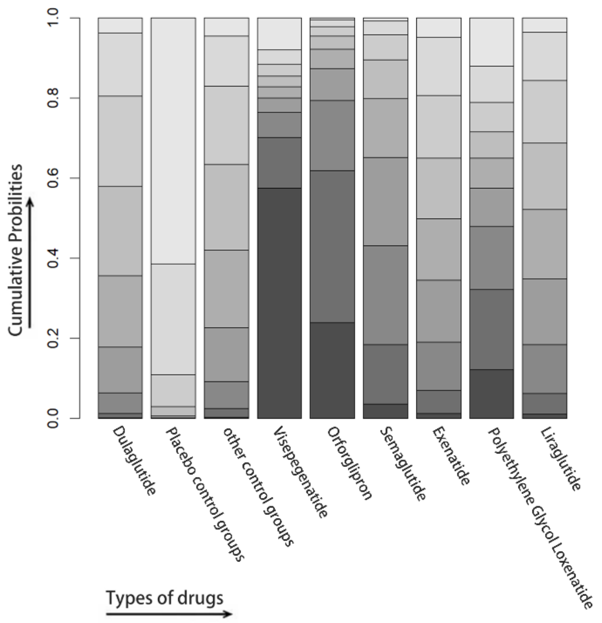

Table S7.11: SUCRA of the effects of various GLP-1RAs on DBP.

Treatment	SUCRA
Dulaglutide	36.90
Semaglutide	61.73
Exenatide	44.19
Polyethylene Glycol Loxenatide	56.61
Liraglutide	45.29
Placebo control groups	6.68
other control groups	39.75
Visepegenatide	79.08
Orforglipron	69.76

Figure S7.12: Stacked sort charts of GLP-1RAs for MACE in network analysis.

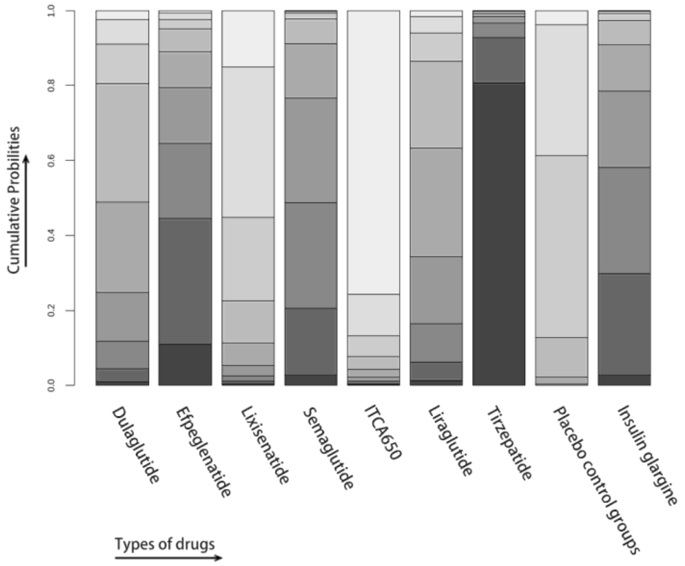

Table S7.12: SUCRA of the effects of various GLP-1RAs on MACE.

Treatment	SUCRA
Placebo control groups	21.60
Dulaglutide	45.04
Semaglutide	67.19
Liraglutide	49.65
Lixisenatide	21.53
Efpeglenatide	72.30
Tirzepatide	96.21
ITCA650	6.82
Insulin glargine	69.65

Figure S7.13: Stacked sort charts of GLP-1RAs for NFS in network analysis.

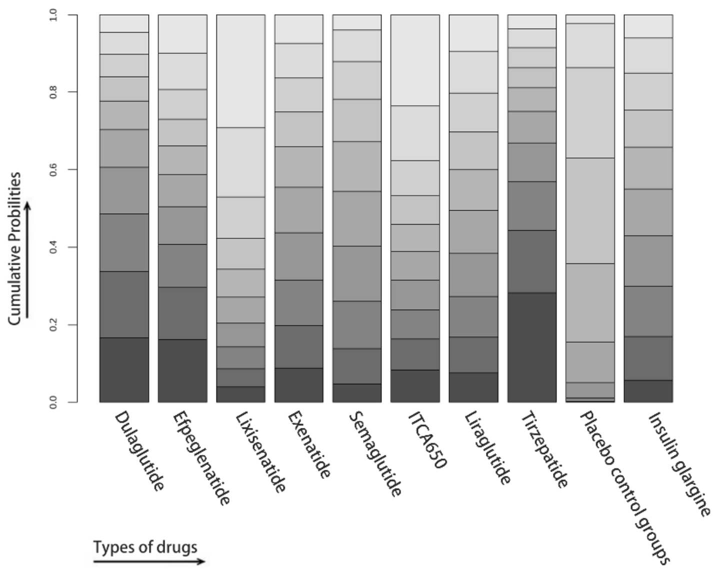

Table S7.13: SUCRA of the effects of various GLP-1RAs on NFS.

Treatment	SUCRA
Placebo control groups	33.87
Dulaglutide	64.08
Semaglutide	52.04
Exenatide	52.92
Liraglutide	48.84
Lixisenatide	30.53
Efpeglenatide	56.17
Tirzepatide	69.62
ITCA650	39.63
Insulin glargine	52.28

Figure S7.14: Stacked sort charts of GLP-1RAs for NFM in network analysis.

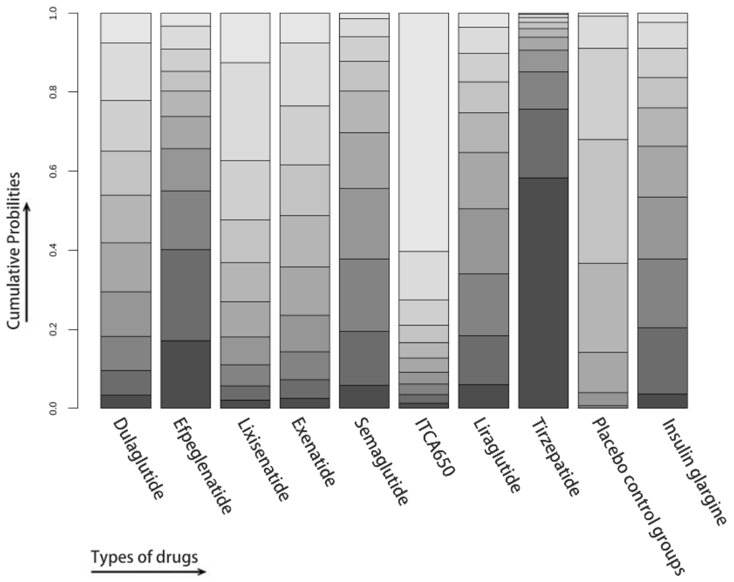

Table S7.14: SUCRA of the effects of various GLP-1RAs on NFM.

Treatment	SUCRA
Placebo control groups	34.85
Dulaglutide	43.52
Semaglutide	61.00
Exenatide	40.33
Liraglutide	57.43
Lixisenatide	33.15
Efpeglenatide	67.20
Tirzepatide	88.39
ITCA650	15.26
Insulin glargine	58.87

Figure S7.15: Stacked sort charts of GLP-1RAs for CVM in network analysis.

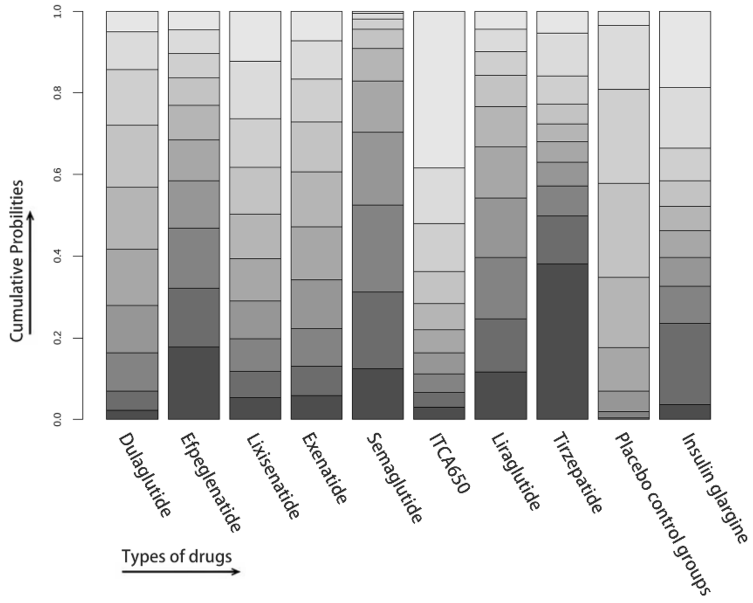

Table S7.15: SUCRA of the effects of various GLP-1RAs on CVM.

Treatment	SUCRA
Placebo control groups	32.97
Dulaglutide	44.97
Semaglutide	70.35
Exenatide	48.02
Liraglutide	60.39
Lixisenatide	42.05
Efpeglenatide	63.25
Tirzepatide	67.16
ITCA650	25.94
Insulin glargine	44.88

Figure S7.16: Stacked sort charts of GLP-1RAs for ACM in network analysis.

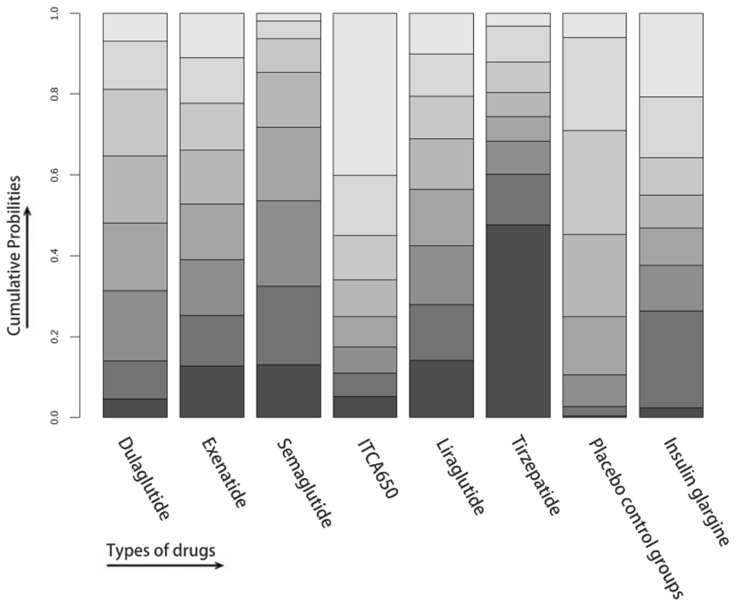

Table S7.16: SUCRA of the effects of various GLP-1RAs on ACM.

Treatment	SUCRA
Placebo control groups	35.53
Dulaglutide	48.09
Semaglutide	64.00
Exenatide	51.81
Liraglutide	54.16
Tirzepatide	73.65
ITCA650	28.20
Insulin glargine	44.55

Figure S7.17: Stacked sort charts of GLP-1RAs for eGFR in network analysis.

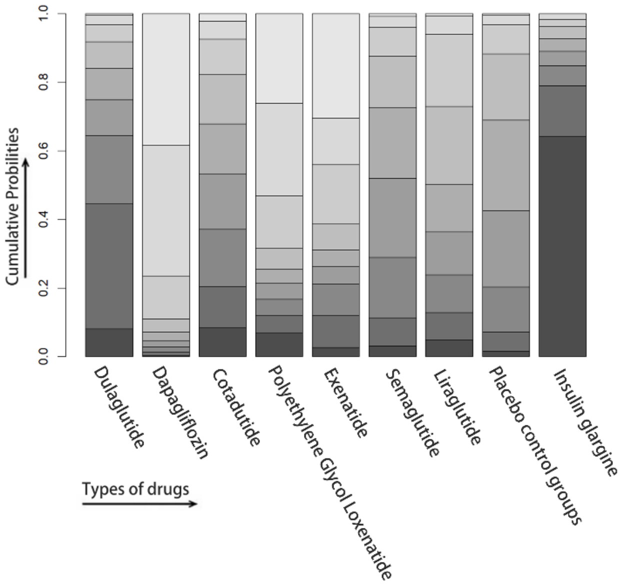

Table S7.17: SUCRA of the effects of various GLP-1RAs on Glomerular filtration rate.

Treatment	SUCRA
Placebo control groups	53.07
Dulaglutide	70.46
Semaglutide	56.35
Exenatide	32.12
Polyethylene Glycol Loxenatide	29.31
Liraglutide	49.25
Cotadutide	57.42
Insulin glargine	87.98
Dapagliflozin	14.03

Figure S7.18: Stacked sort charts of GLP-1RAs for UACR in network analysis.

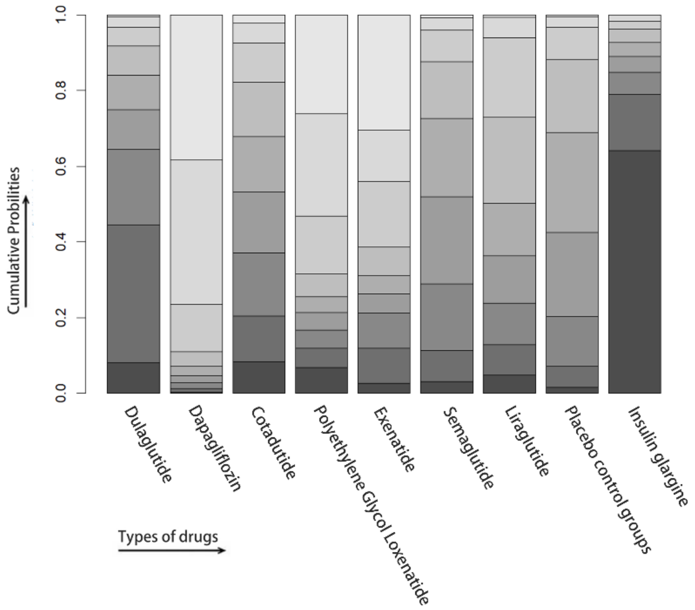

Table S7.18: SUCRA of the effects of various GLP-1RAs on UACR.

Treatment	SUCRA
Placebo control groups	19.28
Dulaglutide	82.21
Semaglutide	53.16
Exenatide	77.98
Polyethylene Glycol Loxenatide	36.54
Liraglutide	31.13
Cotadutide	42.27
Insulin glargine	70.96
Dapagliflozin	36.47

Figure S7.19: Stacked sort charts of GLP-1RAs for FIS in network analysis.

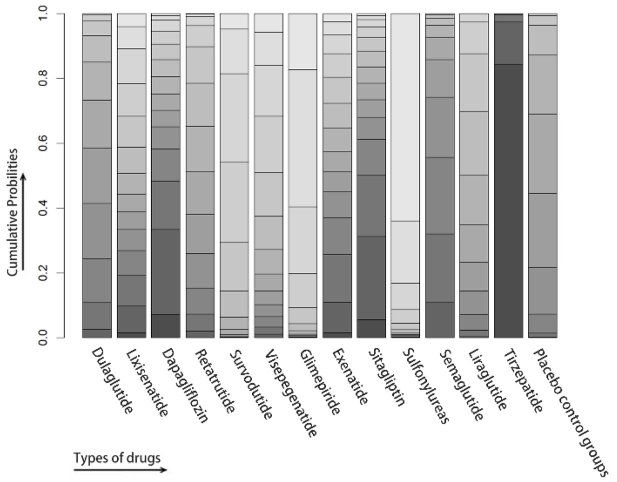

Table S7.19: SUCRA of the effects of various GLP-1RAs on FIS

Treatment	SUCRA
Placebo control groups	55.90
Dulaglutide	60.48
Semaglutide	72.73
Exenatide	55.72
Liraglutide	45.15
Lixisenatide	47.28
Tirzepatide	98.55
Retatrutide	51.39
Survodutide	21.92
Visepegenatid	32.12
Glimepiride	12.37
Sitagliptin	71.15
Sulfonylureas	5.55
Dapagliflozin	69.69

Figure S7.20: Stacked sort charts of GLP-1RAs for C-peptide in network analysis.

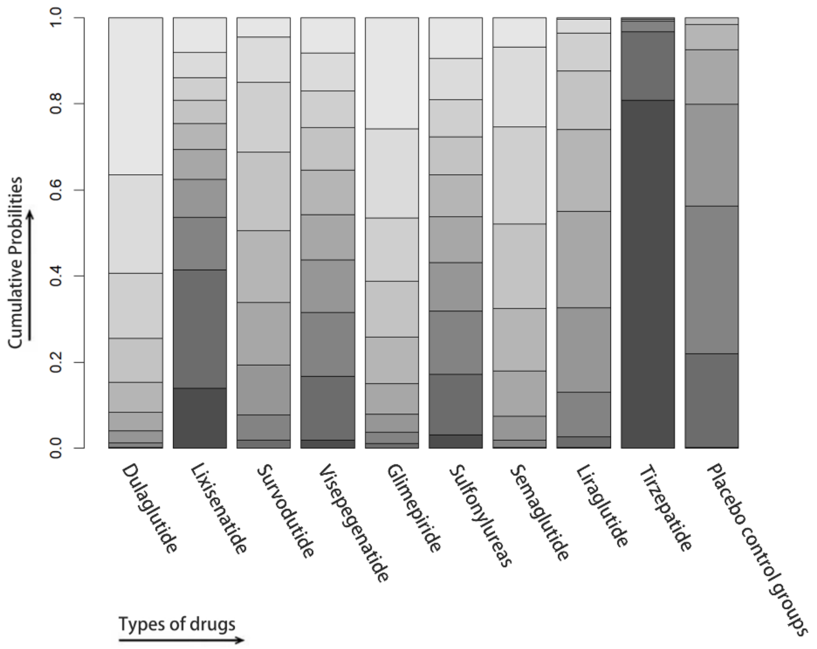

Table S7.20: SUCRA of the effects of various GLP-1RAs on C-peptide.

Treatment	SUCRA
Placebo control groups	72.12
Dulaglutide	17.66
Semaglutide	31.08
Liraglutide	51.20
Lixisenatide	63.85
Tirzepatide	97.33
Survodutide	40.26
Visepegenatid	51.33

Figure S7.21: Stacked sort charts of GLP-1RAs for HOMA-IR in network analysis.

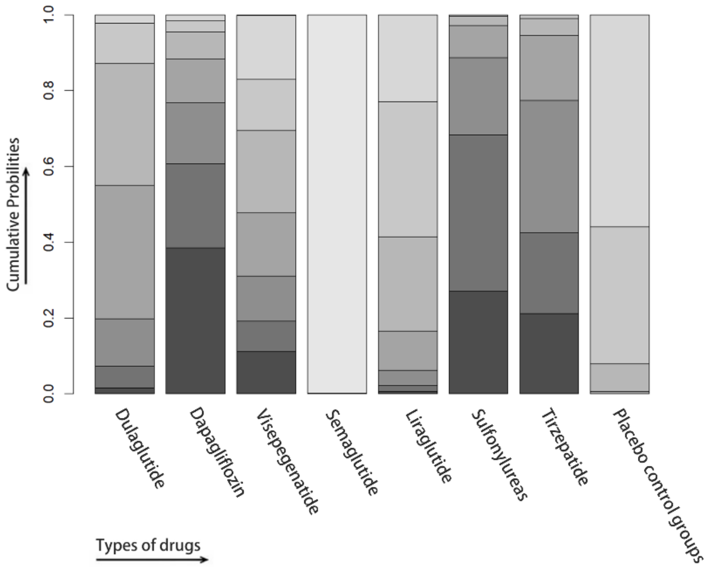

Table S7.21: SUCRA of the effects of various GLP-1RAs on HOMA-IR

Treatment	SUCRA
Placebo control groups	21.79
Dulaglutide	52.64
Semaglutide	34.86
Liraglutide	82.94
Tirzepatide	76.37
Visepegenatid	51.62
Dapagliflozin	79.75

Figure S7.22: Stacked sort charts of GLP-1RAs for HOMA-β in network analysis.

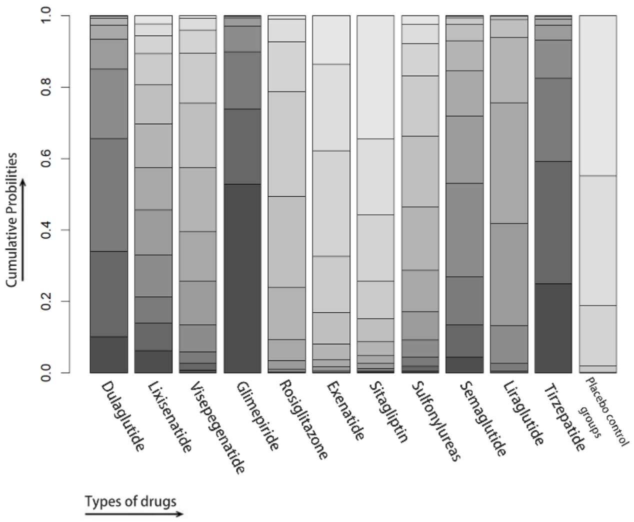

Table S7.22: SUCRA of the effects of various GLP-1RAs on HOMA-β

Treatment	SUCRA
Placebo control groups	6.93
Dulaglutide	80.38
Semaglutide	67.61
Exenatide	19.35
Liraglutide	56.96
Lixisenatide	55.35
Tirzepatide	86.86
Visepegenatid	45.95
Glimepiride	92.03
Rosiglitazone	32.53
Sitagliptin	15.40
Sulfonylureas	40.65

Figure S7.23: B

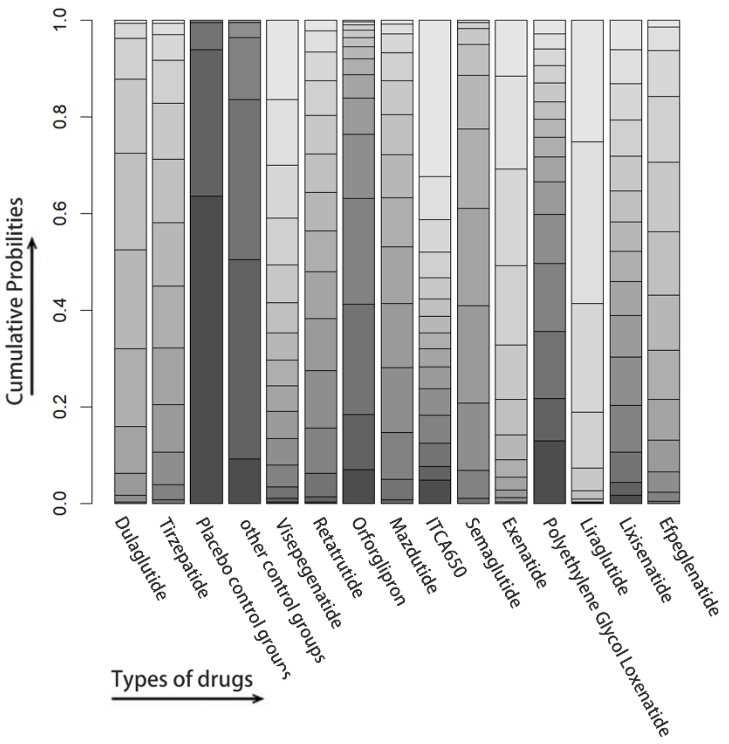

Table S7.23: SUCRA of the effects of various GLP-1RAs on nausea.

Treatment	SUCRA
Dulaglutide	47.39
Semaglutide	57.27
Exenatide	51.38
Polyethylene Glycol Loxenatide	23.12
Liraglutide	28.83
Lixisenatide	42.84
Efpeglenatid	48.37
Tirzepatide	37.53
Placebo control groups	92.04
other control groups	99.29

Visepegenatide	16.02
Retatrutide	52.83
Orforglipron	85.41
Mazdutide	57.48
ITCA650	10.19

Figure S7.24: Stacked sort charts of GLP-1RAs for vomiting in network analysis. The darker-colored segments indicate a higher stacking probability, reflecting a greater likelihood of association with vomiting.

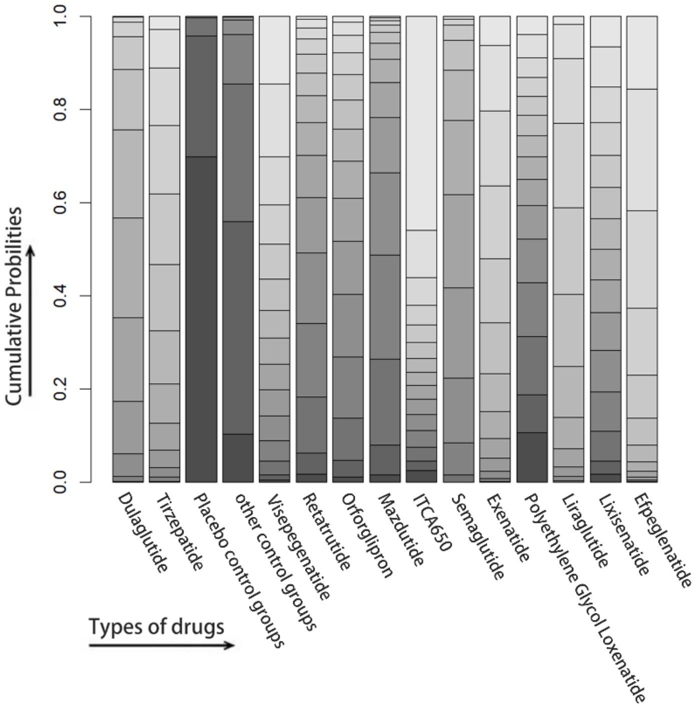

Table S7.24: SUCRA of the effects of various GLP-1RAs on vomiting.

**Treatment**	**SUCRA**
Dulaglutide	71.02
Semaglutide	49.07
Exenatide	30.10
Polyethylene Glycol Loxenatide	31.85
Liraglutide	54.27
Lixisenatide	28.16

Efpeglenatid	37.12
Tirzepatide	35.11
Placebo control groups	92.63
other control groups	99.41
Visepegenatide	20.85
Retatrutide	66.48
Orforglipron	78.31
Mazdutide	55.01
ITCA650	0.6

Figure S7.25: Stacked sort charts of GLP-1RAs for diarrhea in network analysis. The darker-colored segments indicate a higher stacking probability, reflecting a greater likelihood of association with diarrhea.

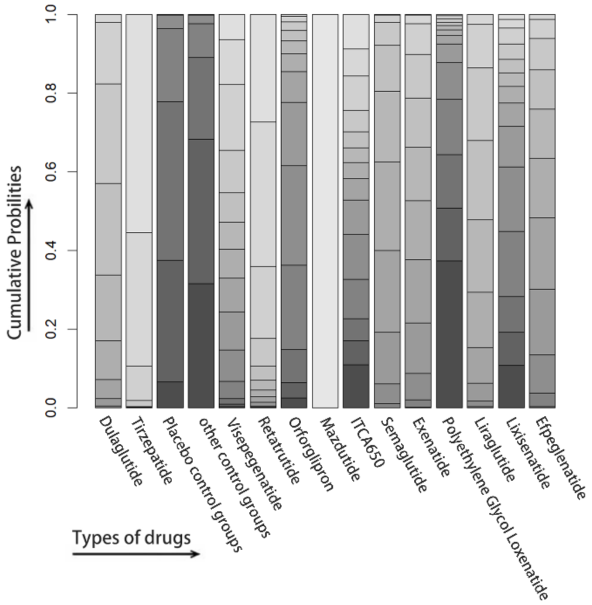

Table S7.25: SUCRA of the effects of various GLP-1RAs on diarrhea.

Treatment	SUCRA
Dulaglutide	40.58
Semaglutide	41.06
Exenatide	38.03

Polyethylene Glycol Loxenatide	85.92
Liraglutide	58.66
Lixisenatide	55.99
Efpeglenatid	59.12
Tirzepatide	21.48
Placebo control groups	87.34
other control groups	95.42
Visepegenatide	39.98
Retatrutide	23.23
Orforglipron	76.94
Mazdutide	0.55
ITCA650	25.69

Figure S7.26: Stacked sort charts of GLP-1RAs for constipation in network analysis. The darker-colored segments indicate a higher stacking probability, reflecting a greater likelihood of association with constipation.

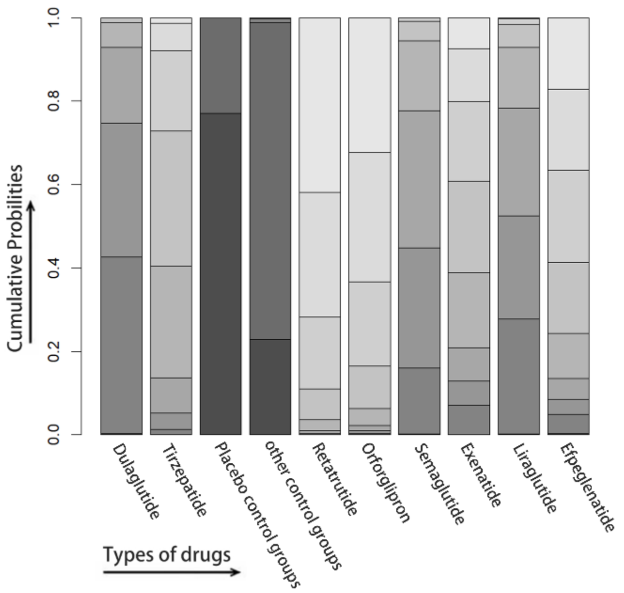

Table S7.26: B

Treatment	SUCRA
Dulaglutide	42.41
Semaglutide	66.57
Exenatide	36.39
Liraglutide	76.60
Efpeglenatid	33.55
Tirzepatide	24.94
Placebo control groups	92.19
other control groups	84.69
Retatrutide	6.48
Orforglipron	35.64

Supplementary 8: league table of Summary Estimates for GLP-1RAs on T2D Derived from Network Meta-analysis of 102 Trials

Abbreviations: FPG, fasting plasma glucose; HDL, highdensity lipoprotein; LDL, low-density lipoprotein; TC, total cholesterol; TG, triglyceride; SBP, systolic blood pressure; DBP, diastolic blood pressure, ACM, all-cause mortality; NFS, non-fatal stroke; NFM, non-fatal myocardial infarction; CVM, cardiovascular mortality; MACE, major adverse cardiovascular events; GFR, glomerular filtration rate; UACR, urinary albumin/creatinine ratio; FIL, fasting insulin level; HOMA-β, homeostasis model assessment of β-cell function; HOMA-IR, homeostasis model assessment of insulin resistance

Table S8.1: The league table of HbA_{1c}

**Dulaglutide**	-0.29 (-0.45, -0.13)	0.06 (-0.15, 0.27)	0.1 (-0.37, 0.57)	0 (-0.16, 0.17)	0.52 (0.16, 0.88)	0.24 (-0.04, 0.51)	-0.92 (-1.14, -0.7)	1 (0.87, 1.13)	0.32 (0.16, 0.48)	-0.11 (-0.45, 0.24)	0.36 (-0.3, 1)	-0.2 (-0.6, 0.2)	-0.55 (-0.9, -0.19)	-0.12 (-0.45, 0.21)	0.2 (-0.7, 1.12)	0.19 (-0.28, 0.66)
0.29 (0.13, 0.45)	**Semaglutide**	0.35 (0.14, 0.56)	0.39 (-0.08, 0.86)	0.29 (0.14, 0.45)	0.81 (0.45, 1.17)	0.53 (0.26, 0.79)	-0.63 (-0.86, -0.4)	1.29 (1.17, 1.42)	0.61 (0.45, 0.76)	0.18 (-0.14, 0.51)	0.64 (0, 1.29)	0.09 (-0.32, 0.5)	-0.26 (-0.63, 0.12)	0.17 (-0.18, 0.52)	0.49 (-0.41, 1.41)	0.48 (0.01, 0.94)
-0.06 (-0.27, 0.15)	-0.35 (-0.56, -0.14)	**Exenatide**	0.04 (-0.44, 0.53)	-0.06 (-0.27, 0.15)	0.46 (0.09, 0.83)	0.18 (-0.13, 0.48)	-0.98 (-1.25, -0.71)	0.94 (0.76, 1.12)	0.26 (0.05, 0.46)	-0.17 (-0.54, 0.2)	0.29 (-0.37, 0.95)	-0.26 (-0.7, 0.17)	-0.61 (-1, -0.21)	-0.18 (-0.56, 0.19)	0.14 (-0.77, 1.06)	0.13 (-0.36, 0.61)
-0.1 (-0.57, 0.37)	-0.39 (-0.86, 0.08)	-0.04 (-0.53, 0.44)	**Polyethylene Glycol Loxenatide**	-0.1 (-0.57, 0.37)	0.42 (-0.15, 0.98)	0.13 (-0.39, 0.65)	-1.02 (-1.52, -0.53)	0.9 (0.44, 1.36)	0.22 (-0.24, 0.67)	-0.21 (-0.77, 0.35)	0.25 (-0.53, 1.03)	-0.3 (-0.91, 0.3)	-0.65 (-1.23, -0.07)	-0.23 (-0.79, 0.33)	0.1 (-0.91, 1.11)	0.08 (-0.56, 0.73)
0 (-0.17, 0.16)	-0.29 (-0.45, -0.14)	0.06 (-0.15, 0.27)	0.1 (-0.37, 0.57)	**Liraglutide**	0.52 (0.16, 0.87)	0.23 (-0.03, 0.5)	-0.92 (-1.16, -0.69)	1 (0.88, 1.13)	0.32 (0.16, 0.47)	-0.11 (-0.45, 0.24)	0.35 (-0.3, 1)	-0.2 (-0.62, 0.21)	-0.55 (-0.92, -0.18)	-0.13 (-0.47, 0.22)	0.2 (-0.7, 1.11)	0.19 (-0.27, 0.64)
-0.52 (-0.88, -0.16)	-0.81 (-1.17, -0.45)	-0.46 (-0.83, -0.09)	-0.42 (-0.98, 0.15)	-0.52 (-0.87, -0.16)	**Lixisenatide**	-0.28 (-0.71, 0.14)	-1.44 (-1.84, -1.04)	0.48 (0.14, 0.83)	-0.2 (-0.56, 0.16)	-0.63 (-1.1, -0.16)	-0.17 (-0.89, 0.56)	-0.72 (-1.25, -0.2)	-1.07 (-1.56, -0.57)	-0.64 (-1.12, -0.17)	-0.32 (-1.27, 0.65)	-0.33 (-0.9, 0.24)
-0.24 (-0.51, 0.04)	-0.53 (-0.79, -0.26)	-0.18 (-0.48, 0.13)	-0.13 (-0.65, 0.39)	-0.23 (-0.5, 0.03)	0.28 (-0.14, 0.71)	**Efpeglenatide**	-1.16 (-1.48, -0.84)	0.77 (0.52, 1.01)	0.08 (-0.2, 0.36)	-0.34 (-0.75, 0.06)	0.12 (-0.56, 0.8)	-0.44 (-0.9, 0.03)	-0.78 (-1.21, -0.35)	-0.36 (-0.77, 0.05)	-0.03 (-0.96, 0.9)	-0.05 (-0.56, 0.46)
0.92 (0.7, 1.14)	0.63 (0.4, 0.86)	0.98 (0.71, 1.25)	1.02 (0.53, 1.52)	0.92 (0.69, 1.16)	1.44 (1.04, 1.84)	1.16 (0.84, 1.48)	**Tirzepatide**	1.92 (1.72, 2.13)	1.24 (1.01, 1.47)	0.81 (0.43, 1.2)	1.28 (0.61, 1.94)	0.72 (0.28, 1.16)	0.37 (-0.03, 0.78)	0.8 (0.42, 1.18)	1.12 (0.21, 2.05)	1.11 (0.61, 1.61)
-1 (-1.13, -0.87)	-1.29 (-1.42, -1.17)	-0.94 (-1.12, -0.76)	-0.9 (-1.36, -0.44)	-1 (-1.13, -0.88)	-0.48 (-0.83, -0.14)	-0.77 (-1.01, -0.52)	-1.92 (-2.13, -1.72)	**Placebo control groups**	-0.69 (-0.83, -0.54)	-1.11 (-1.44, -0.79)	-0.65 (-1.29, -0.01)	-1.2 (-1.6, -0.81)	-1.55 (-1.91, -1.2)	-1.13 (-1.46, -0.8)	-0.8 (-1.7, 0.1)	-0.82 (-1.27, -0.36)
-0.32 (-0.48, -0.16)	-0.61 (-0.76, -0.45)	-0.26 (-0.46, -0.05)	-0.22 (-0.67, 0.24)	-0.32 (-0.47, -0.16)	0.2 (-0.16, 0.56)	-0.08 (-0.36, 0.2)	-1.24 (-1.47, -1.01)	0.69 (0.54, 0.83)	**other control. groups**	-0.42 (-0.77, -0.08)	0.04 (-0.62, 0.69)	-0.52 (-0.93, -0.1)	-0.87 (-1.24, -0.49)	-0.44 (-0.79, -0.09)	-0.11 (-1.02, 0.8)	-0.13 (-0.6, 0.34)
0.11 (-0.24, 0.45)	-0.18 (-0.51, 0.14)	0.17 (-0.2, 0.54)	0.21 (-0.35, 0.77)	0.11 (-0.24, 0.45)	0.63 (0.16, 1.1)	0.34 (-0.06, 0.75)	-0.81 (-1.2, -0.43)	1.11 (0.79, 1.44)	0.42 (0.08, 0.77)	**Survodutide**	0.46 (-0.25, 1.17)	-0.09 (-0.61, 0.42)	-0.44 (-0.92, 0.04)	-0.02 (-0.48, 0.45)	0.31 (-0.64, 1.26)	0.29 (-0.26, 0.85)
-0.36 (-1, 0.3)	-0.64 (-1.29, 0)	-0.29 (-0.95, 0.37)	-0.25 (-1.03, 0.53)	-0.35 (-1, 0.3)	0.17 (-0.56, 0.89)	-0.12 (-0.8, 0.56)	-1.28 (-1.94, -0.61)	0.65 (0.01, 1.29)	-0.04 (-0.69, 0.62)	-0.46 (-1.17, 0.25)	**Visepegenatide**	-0.56 (-1.31, 0.19)	-0.9 (-1.63, -0.17)	-0.48 (-1.19, 0.24)	-0.15 (-1.25, 0.95)	-0.17 (-0.95, 0.62)
0.2 (-0.2, 0.6)	-0.09 (-0.5, 0.32)	0.26 (-0.17, 0.7)	0.3 (-0.3, 0.91)	0.2 (-0.21, 0.62)	0.72 (0.2, 1.25)	0.44 (-0.03, 0.9)	-0.72 (-1.16, -0.28)	1.2 (0.81, 1.6)	0.52 (0.1, 0.93)	0.09 (-0.42, 0.61)	0.56 (-0.19, 1.31)	**Retatrutide**	-0.35 (-0.87, 0.18)	0.08 (-0.43, 0.58)	0.4 (-0.58, 1.39)	0.39 (-0.21, 0.99)
0.55 (0.19, 0.9)	0.26 (-0.12, 0.63)	0.61 (0.21, 1)	0.65 (0.07, 1.23)	0.55 (0.18, 0.92)	1.07 (0.57, 1.56)	0.78 (0.35, 1.21)	-0.37 (-0.78, 0.03)	1.55 (1.2, 1.91)	0.87 (0.49, 1.24)	0.44 (-0.04, 0.92)	0.9 (0.17, 1.63)	0.35 (-0.18, 0.87)	**Orforglipron**	0.42 (-0.05, 0.9)	0.75 (-0.21, 1.72)	0.73 (0.16, 1.31)
0.12 (-0.21, 0.45)	-0.17 (-0.52, 0.18)	0.18 (-0.19, 0.56)	0.23 (-0.33, 0.79)	0.13 (-0.22, 0.47)	0.64 (0.17, 1.12)	0.36 (-0.05, 0.77)	-0.8 (-1.18, -0.42)	1.13 (0.8, 1.46)	0.44 (0.09, 0.79)	0.02 (-0.45, 0.48)	0.48 (-0.24, 1.19)	-0.08 (-0.58, 0.43)	-0.42 (-0.9, 0.05)	**Mazdutide**	0.33 (-0.63, 1.29)	0.31 (-0.25, 0.87)
-0.2 (-1.12, 0.7)	-0.49 (-1.41, 0.41)	-0.14 (-1.06, 0.77)	-0.1 (-1.11, 0.91)	-0.2 (-1.11, 0.7)	0.32 (-0.65, 1.27)	0.03 (-0.9, 0.96)	-1.12 (-2.05, -0.21)	0.8 (-0.1, 1.7)	0.11 (-0.8, 1.02)	-0.31 (-1.26, 0.64)	0.15 (-0.95, 1.25)	-0.4 (-1.39, 0.58)	-0.75 (-1.72, 0.21)	-0.33 (-1.29, 0.63)	**ITCA650**	-0.02 (-1.03, 0.98)
-0.19 (-0.66, 0.28)	-0.48 (-0.94, -0.01)	-0.13 (-0.61, 0.36)	-0.08 (-0.73, 0.56)	-0.19 (-0.64, 0.27)	0.33 (-0.24, 0.9)	0.05 (-0.46, 0.56)	-1.11 (-1.61, -0.61)	0.82 (0.36, 1.27)	0.13 (-0.34, 0.6)	-0.29 (-0.85, 0.26)	0.17 (-0.62, 0.95)	-0.39 (-0.99, 0.21)	-0.73 (-1.31, -0.16)	-0.31 (-0.87, 0.25)	0.02 (-0.98, 1.03)	**Cotadutide**

Table S8.2: The league table of FPG

**Dulaglutide**	-0.7 (-1.04, -0.36)	0.09 (-0.34, 0.52)	-0.01 (-1.02, 1)	-0.17 (-0.51, 0.18)	0.24 (-0.45, 0.93)	-0.06 (-0.58, 0.47)	-1.29 (-1.7, -0.87)	1.35 (1.1, 1.61)	0.2 (-0.11, 0.52)	0.67 (-0.56, 1.9)	-0.37 (-1.11, 0.38)	-0.86 (-1.53, -0.18)	-0.17 (-0.79, 0.45)
0.7 (0.36, 1.04)	**Semaglutide**	0.79 (0.34, 1.24)	0.69 (-0.32, 1.71)	0.53 (0.18, 0.88)	0.94 (0.24, 1.64)	0.64 (0.11, 1.17)	-0.59 (-1.05, -0.13)	2.05 (1.77, 2.33)	0.9 (0.58, 1.23)	1.37 (0.14, 2.6)	0.33 (-0.45, 1.12)	-0.15 (-0.87, 0.56)	0.53 (-0.14, 1.2)
-0.09 (-0.52, 0.34)	-0.79 (-1.24, -0.34)	**Exenatide**	-0.09 (-1.15, 0.95)	-0.25 (-0.7, 0.19)	0.15 (-0.56, 0.87)	-0.15 (-0.74, 0.44)	-1.38 (-1.91, -0.85)	1.26 (0.89, 1.64)	0.12 (-0.32, 0.55)	0.58 (-0.67, 1.84)	-0.46 (-1.28, 0.38)	-0.94 (-1.7, -0.18)	-0.26 (-0.97, 0.45)
0.01 (-1, 1.02)	-0.69 (-1.71, 0.32)	0.09 (-0.95, 1.15)	**Polyethylene Glycol Loxenatide**	-0.16 (-1.18, 0.86)	0.25 (-0.93, 1.42)	-0.05 (-1.14, 1.04)	-1.28 (-2.34, -0.23)	1.36 (0.37, 2.34)	0.21 (-0.79, 1.21)	0.68 (-0.88, 2.23)	-0.36 (-1.59, 0.87)	-0.85 (-2.04, 0.34)	-0.16 (-1.32, 0.99)
0.17 (-0.18, 0.51)	-0.53 (-0.88, -0.18)	0.25 (-0.19, 0.7)	0.16 (-0.86, 1.18)	**Liraglutide**	0.41 (-0.28, 1.09)	0.11 (-0.4, 0.61)	-1.12 (-1.58, -0.66)	1.52 (1.24, 1.8)	0.37 (0.03, 0.71)	0.84 (-0.4, 2.07)	-0.2 (-0.99, 0.59)	-0.69 (-1.4, 0.03)	0 (-0.67, 0.66)
-0.24 (-0.93, 0.45)	-0.94 (-1.64, -0.24)	-0.15 (-0.87, 0.56)	-0.25 (-1.42, 0.93)	-0.41 (-1.09, 0.28)	**Lixisenatide**	-0.3 (-1.1, 0.49)	-1.53 (-2.28, -0.78)	1.11 (0.46, 1.76)	-0.04 (-0.73, 0.65)	0.43 (-0.94, 1.79)	-0.61 (-1.6, 0.38)	-1.09 (-2.03, -0.16)	-0.41 (-1.3, 0.48)
0.06 (-0.47, 0.58)	-0.64 (-1.17, -0.11)	0.15 (-0.44, 0.74)	0.05 (-1.04, 1.14)	-0.11 (-0.61, 0.4)	0.3 (-0.49, 1.1)	**Efpeglenatide**	-1.23 (-1.83, -0.62)	1.41 (0.94, 1.88)	0.26 (-0.28, 0.8)	0.73 (-0.56, 2.02)	-0.31 (-1.19, 0.57)	-0.79 (-1.61, 0.02)	-0.11 (-0.88, 0.66)
1.29 (0.87, 1.7)	0.59 (0.13, 1.05)	1.38 (0.85, 1.91)	1.28 (0.23, 2.34)	1.12 (0.66, 1.58)	1.53 (0.78, 2.28)	1.23 (0.62, 1.83)	**Tirzepatide**	2.64 (2.25, 3.03)	1.49 (1.04, 1.94)	1.96 (0.7, 3.22)	0.92 (0.09, 1.75)	0.43 (-0.33, 1.2)	1.12 (0.4, 1.83)
-1.35 (-1.61, -1.1)	-2.05 (-2.33, -1.77)	-1.26 (-1.64, -0.89)	-1.36 (-2.34, -0.37)	-1.52 (-1.8, -1.24)	-1.11 (-1.76, -0.46)	-1.41 (-1.88, -0.94)	-2.64 (-3.03, -2.25)	**Placebo control groups**	-1.15 (-1.45, -0.84)	-0.68 (-1.88, 0.52)	-1.72 (-2.46, -0.97)	-2.21 (-2.88, -1.54)	-1.52 (-2.14, -0.91)
-0.2 (-0.52, 0.11)	-0.9 (-1.23, -0.58)	-0.12 (-0.55, 0.32)	-0.21 (-1.21, 0.79)	-0.37 (-0.71, -0.03)	0.04 (-0.65, 0.73)	-0.26 (-0.8, 0.28)	-1.49 (-1.94, -1.04)	1.15 (0.84, 1.45)	**other control groups**	0.47 (-0.77, 1.71)	-0.57 (-1.36, 0.22)	-1.06 (-1.78, -0.34)	-0.37 (-1.04, 0.29)
-0.67 (-1.9, 0.56)	-1.37 (-2.6, -0.14)	-0.58 (-1.84, 0.67)	-0.68 (-2.23, 0.88)	-0.84 (-2.07, 0.4)	-0.43 (-1.79, 0.94)	-0.73 (-2.02, 0.56)	-1.96 (-3.22, -0.7)	0.68 (-0.52, 1.88)	-0.47 (-1.71, 0.77)	**Visepegenatide**	-1.04 (-2.45, 0.38)	-1.53 (-2.9, -0.15)	-0.84 (-2.19, 0.51)
0.37 (-0.38, 1.11)	-0.33 (-1.12, 0.45)	0.46 (-0.38, 1.28)	0.36 (-0.87, 1.59)	0.2 (-0.59, 0.99)	0.61 (-0.38, 1.6)	0.31 (-0.57, 1.19)	-0.92 (-1.75, -0.09)	1.72 (0.97, 2.46)	0.57 (-0.22, 1.36)	1.04 (-0.38, 2.45)	**Retatrutide**	-0.49 (-1.48, 0.5)	0.19 (-0.76, 1.15)
0.86 (0.18, 1.53)	0.15 (-0.56, 0.87)	0.94 (0.18, 1.7)	0.85 (-0.34, 2.04)	0.69 (-0.03, 1.4)	1.09 (0.16, 2.03)	0.79 (-0.02, 1.61)	-0.43 (-1.2, 0.33)	2.21 (1.54, 2.88)	1.06 (0.34, 1.78)	1.53 (0.15, 2.9)	0.49 (-0.5, 1.48)	**Orforglipron**	0.68 (-0.21, 1.58)
0.17 (-0.45, 0.79)	-0.53 (-1.2, 0.14)	0.26 (-0.45, 0.97)	0.16 (-0.99, 1.32)	0 (-0.66, 0.67)	0.41 (-0.48, 1.3)	0.11 (-0.66, 0.88)	-1.12 (-1.83, -0.4)	1.52 (0.91, 2.14)	0.37 (-0.29, 1.04)	0.84 (-0.51, 2.19)	-0.19 (-1.15, 0.76)	-0.68 (-1.58, 0.21)	**Mazdutide**

Table S8.3: The league table of Weight loss

**Dulaglutide**	-1.97 (-2.69, -1.25)	-0.77 (-1.71, 0.18)	1.42 (-0.98, 3.83)	-0.56 (-1.31, 0.19)	-0.33 (-2.08, 1.42)	-0.15 (-1.39, 1.08)	-6.62 (-7.61, -5.64)	1.14 (0.56, 1.72)	1.92 (1.21, 2.64)	-3.16 (-4.71, -1.61)	-8.55 (-10.33, -6.78	-4.28 (-5.88, -2.69)	-2.54 (-4.01, -1.08)	0.14 (-3.96, 4.22)	-1.68 (-3.8, 0.42)
1.97 (1.25, 2.69)	**Semaglutide**	1.2 (0.25, 2.15)	3.39 (0.99, 5.79)	1.41 (0.7, 2.12)	1.64 (-0.11, 3.38)	1.82 (0.6, 3.03)	-4.65 (-5.7, -3.6)	3.11 (2.54, 3.68)	3.89 (3.19, 4.59)	-1.19 (-2.65, 0.27)	-6.59 (-8.43, -4.74)	-2.31 (-3.98, -0.64)	-0.57 (-2.12, 0.97)	2.1 (-1.99, 6.18)	0.29 (-1.82, 2.39)
0.77 (-0.18, 1.71)	-1.2 (-2.15, -0.25)	**Exenatide**	2.18 (-0.28, 4.66)	0.2 (-0.74, 1.15)	0.43 (-1.35, 2.21)	0.61 (-0.75, 1.97)	-5.86 (-7.06, -4.66)	1.9 (1.1, 2.71)	2.69 (1.76, 3.62)	-2.39 (-4.05, -0.74)	-7.79 (-9.73, -5.85)	-3.51 (-5.3, -1.75)	-1.78 (-3.44, -0.13)	0.9 (-3.24, 5.02)	-0.92 (-3.1, 1.27)
-1.42 (-3.83, 0.98)	-3.39 (-5.79, -0.99)	-2.18 (-4.66, 0.28)	**Polyethylene Glycol Loxenatide**	-1.98 (-4.39, 0.42)	-1.75 (-4.64, 1.1)	-1.58 (-4.17, 1.02)	-8.05 (-10.55, -5.53	-0.28 (-2.63, 2.06)	0.5 (-1.87, 2.88)	-4.59 (-7.33, -1.84)	-9.98 (-12.93, -7.04	-5.7 (-8.53, -2.89)	-3.96 (-6.72, -1.21)	-1.3 (-5.97, 3.37)	-3.1 (-6.21, -0.01)
0.56 (-0.19, 1.31)	-1.41 (-2.12, -0.7)	-0.2 (-1.15, 0.74)	1.98 (-0.42, 4.39)	**Liraglutide**	0.23 (-1.49, 1.95)	0.41 (-0.77, 1.58)	-6.06 (-7.12, -5)	1.7 (1.12, 2.28)	2.48 (1.77, 3.2)	-2.6 (-4.14, -1.06)	-7.99 (-9.85, -6.14)	-3.72 (-5.41, -2.05)	-1.98 (-3.54, -0.43)	0.69 (-3.39, 4.77)	-1.12 (-3.18, 0.92)
0.33 (-1.42, 2.08)	-1.64 (-3.38, 0.11)	-0.43 (-2.21, 1.35)	1.75 (-1.1, 4.64)	-0.23 (-1.95, 1.49)	**Lixisenatide**	0.18 (-1.81, 2.17)	-6.29 (-8.18, -4.41)	1.47 (-0.2, 3.14)	2.26 (0.53, 3.99)	-2.83 (-5.04, -0.63)	-8.22 (-10.66, -5.8)	-3.95 (-6.26, -1.65)	-2.21 (-4.43, 0)	0.47 (-3.9, 4.85)	-1.35 (-3.97, 1.28)
0.15 (-1.08, 1.39)	-1.82 (-3.03, -0.6)	-0.61 (-1.97, 0.75)	1.58 (-1.02, 4.17)	-0.41 (-1.58, 0.77)	-0.18 (-2.17, 1.81)	**Efpeglenatide**	-6.47 (-7.9, -5.02)	1.29 (0.19, 2.4)	2.08 (0.83, 3.34)	-3 (-4.83, -1.19)	-8.4 (-10.49, -6.31)	-4.13 (-6.07, -2.19)	-2.39 (-4.21, -0.56)	0.29 (-3.91, 4.46)	-1.53 (-3.83, 0.78)
6.62 (5.64, 7.61)	4.65 (3.6, 5.7)	5.86 (4.66, 7.06)	8.05 (5.53, 10.55)	6.06 (5, 7.12)	6.29 (4.41, 8.18)	6.47 (5.02, 7.9)	**Tirzepatide**	7.76 (6.83, 8.68)	8.55 (7.5, 9.59)	3.46 (1.75, 5.17)	-1.93 (-3.9, 0.04)	2.34 (0.52, 4.16)	4.08 (2.38, 5.78)	6.76 (2.6, 10.9)	4.94 (2.71, 7.17)
-1.14 (-1.72, -0.56)	-3.11 (-3.68, -2.54)	-1.9 (-2.71, -1.1)	0.28 (-2.06, 2.63)	-1.7 (-2.28, -1.12)	-1.47 (-3.14, 0.2)	-1.29 (-2.4, -0.19)	-7.76 (-8.68, -6.83)	**Placebo control groups**	0.79 (0.12, 1.45)	-4.3 (-5.75, -2.84)	-9.69 (-11.47, -7.92	-5.42 (-7.02, -3.83)	-3.68 (-5.14, -2.22)	-1.01 (-5.05, 3.04)	-2.82 (-4.87, -0.77)
-1.92 (-2.64, -1.21)	-3.89 (-4.59, -3.19)	-2.69 (-3.62, -1.76)	-0.5 (-2.88, 1.87)	-2.48 (-3.2, -1.77)	-2.26 (-3.99, -0.53)	-2.08 (-3.34, -0.83)	-8.55 (-9.59, -7.5)	-0.79 (-1.45, -0.12)	**other control groups**	-5.09 (-6.65, -3.53)	10.48 (-12.34, -8.61	-6.21 (-7.9, -4.52)	-4.46 (-6.04, -2.91)	-1.79 (-5.9, 2.3)	-3.6 (-5.73, -1.49)
3.16 (1.61, 4.71)	1.19 (-0.27, 2.65)	2.39 (0.74, 4.05)	4.59 (1.84, 7.33)	2.6 (1.06, 4.14)	2.83 (0.63, 5.04)	3 (1.19, 4.83)	-3.46 (-5.17, -1.75)	4.3 (2.84, 5.75)	5.09 (3.53, 6.65)	**Survodutide**	-5.39 (-7.68, -3.1)	-1.12 (-3.28, 1.03)	0.62 (-1.43, 2.68)	3.29 (-1.01, 7.59)	1.48 (-1.02, 3.99)
8.55 (6.78, 10.33)	6.59 (4.74, 8.43)	7.79 (5.85, 9.73)	9.98 (7.04, 12.93)	7.99 (6.14, 9.85)	8.22 (5.8, 10.66)	8.4 (6.31, 10.49)	1.93 (-0.04, 3.9)	9.69 (7.92, 11.47)	10.48 (8.61, 12.34)	5.39 (3.1, 7.68)	**Retatrutide**	4.27 (1.91, 6.63)	6.01 (3.76, 8.27)	8.69 (4.26, 13.1)	6.87 (4.17, 9.57)
4.28 (2.69, 5.88)	2.31 (0.64, 3.98)	3.51 (1.75, 5.3)	5.7 (2.89, 8.53)	3.72 (2.05, 5.41)	3.95 (1.65, 6.26)	4.13 (2.19, 6.07)	-2.34 (-4.16, -0.52)	5.42 (3.83, 7.02)	6.21 (4.52, 7.9)	1.12 (-1.03, 3.28)	-4.27 (-6.63, -1.91)	**Orforglipron**	1.74 (-0.38, 3.86)	4.41 (0.07, 8.76)	2.6 (0, 5.18)
2.54 (1.08, 4.01)	0.57 (-0.97, 2.12)	1.78 (0.13, 3.44)	3.96 (1.21, 6.72)	1.98 (0.43, 3.54)	2.21 (0, 4.43)	2.39 (0.56, 4.21)	-4.08 (-5.78, -2.38)	3.68 (2.22, 5.14)	4.46 (2.91, 6.04)	-0.62 (-2.68, 1.43)	-6.01 (-8.27, -3.76)	-1.74 (-3.86, 0.38)	**Mazdutide**	2.67 (-1.64, 6.97)	0.86 (-1.65, 3.36)
-0.14 (-4.22, 3.96)	-2.1 (-6.18, 1.99)	-0.9 (-5.02, 3.24)	1.3 (-3.37, 5.97)	-0.69 (-4.77, 3.39)	-0.47 (-4.85, 3.9)	-0.29 (-4.46, 3.91)	-6.76 (-10.9, -2.6)	1.01 (-3.04, 5.05)	1.79 (-2.3, 5.9)	-3.29 (-7.59, 1.01)	-8.69 (-13.1, -4.26)	-4.41 (-8.76, -0.07)	-2.67 (-6.97, 1.64)	**ITCA650**	-1.82 (-6.35, 2.72)
1.68 (-0.42, 3.8)	-0.29 (-2.39, 1.82)	0.92 (-1.27, 3.1)	3.1 (0.01, 6.21)	1.12 (-0.92, 3.18)	1.35 (-1.28, 3.97)	1.53 (-0.78, 3.83)	-4.94 (-7.17, -2.71)	2.82 (0.77, 4.87)	3.6 (1.49, 5.73)	-1.48 (-3.99, 1.02)	-6.87 (-9.57, -4.17)	-2.6 (-5.18, 0)	-0.86 (-3.36, 1.65)	1.82 (-2.72, 6.35)	**Cotadutide**

Table S8.4: The league table of the proportion of patients achieving the HbA_{1c} target of 7.0%

Dulaglutide	15.98 (10.67, 21.23)	-10.16 (-19.13, -1.24)	-10.82 (-29.48, 7.78)	0.04 (-5.48, 5.55)	-20.44 (-33.96, -6.84)	4.19 (-4.75, 13.16)	22.68 (15.96, 29.47)	-35.75 (-40.26, -31.24)	-15.76 (-21.1, -10.47)	-6.42 (-25.12, 12.28)	22.92 (12.82, 33.04)	7.78 (-5.19, 20.8)	-4.17 (-17.77, 9.43)
-15.98 (-21.23, -10.67)	Semaglutide	-26.13 (-35.23, -17.01)	-26.8 (-45.44, -8.16)	-15.94 (-21.14, -10.73)	-36.4 (-49.96, -22.89)	-11.78 (-20.56, -3)	6.71 (-0.63, 14.1)	-51.72 (-56.24, -47.21)	-31.74 (-36.77, -26.72)	-22.39 (-41.09, -3.71)	6.93 (-3.84, 17.76)	-8.21 (-21.7, 5.37)	-20.16 (-33.63, -6.59)
10.16 (1.24, 19.13)	26.13 (17.01, 35.23)	Exenatide	-0.64 (-20.69, 19.33)	10.19 (1.03, 19.3)	-10.3 (-25.62, 5.14)	14.34 (2.8, 25.88)	32.84 (22.58, 43.16)	-25.6 (-34.05, -17.18)	-5.61 (-14.53, 3.3)	3.75 (-16.22, 23.84)	33.09 (20.08, 46.03)	17.9 (2.65, 33.29)	5.98 (-9.41, 21.48)
10.82 (-7.78, 29.48)	26.8 (8.16, 45.44)	0.64 (-19.33, 20.69)	Polyethylene Glycol Loxenatide	10.88 (-7.67, 29.39)	-9.64 (-31.7, 12.65)	15.02 (-4.72, 34.81)	33.5 (14.28, 52.79)	-24.93 (-43.08, -6.84)	-4.93 (-23.8, 13.92)	4.41 (-21.11, 30.15)	33.73 (12.97, 54.49)	18.6 (-3.65, 40.82)	6.63 (-15.61, 28.93)
-0.04 (-5.55, 5.48)	15.94 (10.73, 21.14)	-10.19 (-19.3, -1.03)	-10.88 (-29.39, 7.67)	Liraglutide	-20.47 (-33.71, -7.27)	4.15 (-4.16, 12.48)	22.64 (15.28, 30.08)	-35.8 (-39.95, -31.63)	-15.81 (-21.42, -10.21)	-6.45 (-25.03, 12.15)	22.88 (12.14, 33.68)	7.72 (-5.75, 21.28)	-4.22 (-17.22, 8.75)
20.44 (6.84, 33.96)	36.4 (22.89, 49.96)	10.3 (-5.14, 25.62)	9.64 (-12.65, 31.7)	20.47 (7.27, 33.71)	Lixisenatide	24.62 (9.64, 39.68)	43.13 (28.69, 57.44)	-15.32 (-28.14, -2.45)	4.67 (-9.11, 18.49)	14.07 (-8.31, 36.17)	43.34 (27.03, 59.77)	28.21 (9.97, 46.55)	16.24 (-1.85, 34.38)
-4.19 (-13.16, 4.75)	11.78 (3, 20.56)	-14.34 (-25.88, -2.8)	-15.02 (-34.81, 4.72)	-4.15 (-12.48, 4.16)	-24.62 (-39.68, -9.64)	Efpeglenatide	18.5 (8.27, 28.65)	-39.95 (-47.94, -31.95)	-19.95 (-29.17, -10.74)	-10.58 (-30.41, 9.33)	18.71 (5.96, 31.57)	3.58 (-11.61, 18.85)	-8.34 (-23.36, 6.68)
-22.68 (-29.47, -15.96)	-6.71 (-14.1, 0.63)	-32.84 (-43.16, -22.58)	-33.5 (-52.79, -14.28)	-22.64 (-30.08, -15.28)	-43.13 (-57.44, -28.69)	-18.5 (-28.65, -8.27)	Tirzepatide	-58.44 (-64.94, -51.99)	-38.45 (-45.78, -31.15)	-29.09 (-48.42, -9.81)	0.23 (-11.46, 11.91)	-14.9 (-29.11, -0.69)	-26.84 (-41.22, -12.44)
35.75 (31.24, 40.26)	51.72 (47.21, 56.24)	25.6 (17.18, 34.05)	24.93 (6.84, 43.08)	35.8 (31.63, 39.95)	15.32 (2.45, 28.14)	39.95 (31.95, 47.94)	58.44 (51.99, 64.94)	Placebo control groups	19.99 (14.75, 25.21)	29.32 (11.21, 47.48)	58.66 (48.57, 68.83)	43.53 (30.54, 56.57)	31.58 (18.58, 44.57)
15.76 (10.47, 21.1)	31.74 (26.72, 36.77)	5.61 (-3.3, 14.53)	4.93 (-13.92, 23.8)	15.81 (10.21, 21.42)	-4.67 (-18.49, 9.11)	19.95 (10.74, 29.17)	38.45 (31.15, 45.78)	-19.99 (-25.21, -14.75)	other control groups	9.34 (-9.49, 28.28)	38.67 (27.74, 49.66)	23.53 (9.9, 37.28)	11.57 (-2.14, 25.34)
6.42 (-12.28, 25.12)	22.39 (3.71, 41.09)	-3.75 (-23.84, 16.22)	-4.41 (-30.15, 21.11)	6.45 (-12.15, 25.03)	-14.07 (-36.17, 8.31)	10.58 (-9.33, 30.41)	29.09 (9.81, 48.42)	-29.32 (-47.48, -11.21)	-9.34 (-28.28, 9.49)	Visepegenatide	29.33 (8.53, 50.11)	14.2 (-8.22, 36.54)	2.24 (-20.02, 24.49)
-22.92 (-33.04, -12.82)	-6.93 (-17.76, 3.84)	-33.09 (-46.03, -20.08)	-33.73 (-54.49, -12.97)	-22.88 (-33.68, -12.14)	-43.34 (-59.77, -27.03)	-18.71 (-31.57, -5.96)	-0.23 (-11.91, 11.46)	-58.66 (-68.83, -48.57)	-38.67 (-49.66, -27.74)	-29.33 (-50.11, -8.53)	Orforglipron	-15.15 (-31.29, 1.08)	-27.07 (-43.49, -10.72)
-7.78 (-20.8, 5.19)	8.21 (-5.37, 21.7)	-17.9 (-33.29, -2.65)	-18.6 (-40.82, 3.65)	-7.72 (-21.28, 5.75)	-28.21 (-46.55, -9.97)	-3.58 (-18.85, 11.61)	14.9 (0.69, 29.11)	-43.53 (-56.57, -30.54)	-23.53 (-37.28, -9.9)	-14.2 (-36.54, 8.22)	15.15 (-1.08, 31.29)	Mazdutide	-11.94 (-30.29, 6.31)
4.17 (-9.43, 17.77)	20.16 (6.59, 33.63)	-5.98 (-21.48, 9.41)	-6.63 (-28.93, 15.61)	4.22 (-8.75, 17.22)	-16.24 (-34.38, 1.85)	8.34 (-6.68, 23.36)	26.84 (12.44, 41.22)	-31.58 (-44.57, -18.58)	-11.57 (-25.34, 2.14)	-2.24 (-24.49, 20.02)	27.07 (10.72, 43.49)	11.94 (-6.31, 30.29)	Cotadutide

Table S8.5: The league table of the proportion of patients achieving the HbA_{1c} target of 6.5%

Dulaglutide	18.42 (12.47, 24.35)	-11.69 (-21.75, -1.59)	-14.09 (-33.14, 5.01)	-0.38 (-6.33, 5.53)	-20.32 (-34.18, -6.43)	0.82 (-8.43, 10.08)	28.41 (21.45, 35.35)	-31.3 (-36.07, -26.56)	-19.5 (-25.08, -13.94)	-14.44 (-33.54, 4.69)	28.12 (17.76, 38.55)	2.35 (-11, 15.67)	-6.86 (-20.85, 7.09)
-18.42 (-24.35, -12.47)	Semaglutide	-30.12 (-40.68, -19.57)	-32.52 (-51.83, -13.28)	-18.81 (-24.93, -12.7)	-38.74 (-52.77, -24.66)	-17.58 (-26.9, -8.3)	9.98 (2.05, 17.94)	-49.73 (-55.12, -44.35)	-37.92 (-43.64, -32.21)	-32.88 (-52.17, -13.57)	9.71 (-1.67, 21.06)	-16.08 (-30.17, -1.99)	-25.26 (-39.42, -11.15)
11.69 (1.59, 21.75)	30.12 (19.57, 40.68)	Exenatide	-2.39 (-23.35, 18.48)	11.31 (0.83, 21.72)	-8.63 (-24.9, 7.67)	12.52 (-0.08, 25.16)	40.11 (28.66, 51.53)	-19.61 (-29.41, -9.8)	-7.81 (-17.75, 2.16)	-2.74 (-23.71, 18.21)	39.83 (25.84, 53.71)	14.03 (-2.29, 30.3)	4.83 (-11.61, 21.24)
14.09 (-5.01, 33.14)	32.52 (13.28, 51.83)	2.39 (-18.48, 23.35)	Polyethylene Glycol Loxenatide	13.71 (-5.39, 32.78)	-6.18 (-28.94, 16.38)	14.93 (-5.31, 35.16)	42.5 (22.83, 62.2)	-17.21 (-35.71, 1.27)	-5.41 (-24.75, 13.96)	-0.34 (-26.46, 25.87)	42.22 (21.07, 63.47)	16.46 (-6.4, 39.3)	7.2 (-15.52, 29.95)
0.38 (-5.53, 6.33)	18.81 (12.7, 24.93)	-11.31 (-21.72, -0.83)	-13.71 (-32.78, 5.39)	Liraglutide	-19.93 (-33.4, -6.36)	1.21 (-7.36, 9.81)	28.8 (21.1, 36.51)	-30.93 (-35.5, -26.31)	-19.11 (-25.17, -13.04)	-14.05 (-33.14, 5.01)	28.51 (17.36, 39.72)	2.72 (-11.24, 16.74)	-6.48 (-19.73, 6.8)
20.32 (6.43, 34.18)	38.74 (24.66, 52.77)	8.63 (-7.67, 24.9)	6.18 (-16.38, 28.94)	19.93 (6.36, 33.4)	Lixisenatide	21.14 (5.77, 36.44)	48.71 (34.06, 63.33)	-11 (-24.14, 2.06)	0.81 (-13.4, 14.91)	5.87 (-16.84, 28.56)	48.42 (31.68, 65.16)	22.63 (3.97, 41.2)	13.45 (-5.16, 31.93)
-0.82 (-10.08, 8.43)	17.58 (8.3, 26.9)	-12.52 (-25.16, 0.08)	-14.93 (-35.16, 5.31)	-1.21 (-9.81, 7.36)	-21.14 (-36.44, -5.77)	Efpeglenatide	27.58 (17.16, 38.05)	-32.12 (-40.31, -23.92)	-20.31 (-29.91, -10.78)	-15.25 (-35.51, 5.01)	27.3 (14.15, 40.48)	1.51 (-14.04, 17.13)	-7.7 (-23.03, 7.66)
-28.41 (-35.35, -21.45)	-9.98 (-17.94, -2.05)	-40.11 (-51.53, -28.66)	-42.5 (-62.2, -22.83)	-28.8 (-36.51, -21.1)	-48.71 (-63.33, -34.06)	-27.58 (-38.05, -17.16)	Tirzepatide	-59.72 (-66.42, -53.06)	-47.92 (-55.52, -40.33)	-42.86 (-62.45, -23.26)	-0.29 (-12.29, 11.72)	-26.07 (-40.63, -11.5)	-35.29 (-50.12, -20.5)
31.3 (26.56, 36.07)	49.73 (44.35, 55.12)	19.61 (9.8, 29.41)	17.21 (-1.27, 35.71)	30.93 (26.31, 35.5)	11 (-2.06, 24.14)	32.12 (23.92, 40.31)	59.72 (53.06, 66.42)	Placebo control groups	11.79 (6.16, 17.48)	16.85 (-1.63, 35.35)	59.44 (49.06, 69.8)	33.64 (20.34, 46.98)	24.43 (11.13, 37.74)
19.5 (13.94, 25.08)	37.92 (32.21, 43.64)	7.81 (-2.16, 17.75)	5.41 (-13.96, 24.75)	19.11 (13.04, 25.17)	-0.81 (-14.91, 13.4)	20.31 (10.78, 29.91)	47.92 (40.33, 55.52)	-11.79 (-17.48, -6.16)	other control groups	5.06 (-14.32, 24.43)	47.64 (36.29, 58.93)	21.85 (7.76, 35.93)	12.63 (-1.52, 26.79)
14.44 (-4.69, 33.54)	32.88 (13.57, 52.17)	2.74 (-18.21, 23.71)	0.34 (-25.87, 26.46)	14.05 (-5.01, 33.14)	-5.87 (-28.56, 16.84)	15.25 (-5.01, 35.51)	42.86 (23.26, 62.45)	-16.85 (-35.35, 1.63)	-5.06 (-24.43, 14.32)	Visepegenatide	42.59 (21.4, 63.79)	16.8 (-6.01, 39.51)	7.55 (-15.14, 30.3)
-28.12 (-38.55, -17.76)	-9.71 (-21.06, 1.67)	-39.83 (-53.71, -25.84)	-42.22 (-63.47, -21.07)	-28.51 (-39.72, -17.36)	-48.42 (-65.16, -31.68)	-27.3 (-40.48, -14.15)	0.29 (-11.72, 12.29)	-59.44 (-69.8, -49.06)	-47.64 (-58.93, -36.29)	-42.59 (-63.79, -21.4)	Orforglipron	-25.79 (-42.31, -9.25)	-34.98 (-51.89, -18.27)
-2.35 (-15.67, 11)	16.08 (1.99, 30.17)	-14.03 (-30.3, 2.29)	-16.46 (-39.3, 6.4)	-2.72 (-16.74, 11.24)	-22.63 (-41.2, -3.97)	-1.51 (-17.13, 14.04)	26.07 (11.5, 40.63)	-33.64 (-46.98, -20.34)	-21.85 (-35.93, -7.76)	-16.8 (-39.51, 6.01)	25.79 (9.25, 42.31)	Mazdutide	-9.21 (-28.06, 9.51)
6.86 (-7.09, 20.85)	25.26 (11.15, 39.42)	-4.83 (-21.24, 11.61)	-7.2 (-29.95, 15.52)	6.48 (-6.8, 19.73)	-13.45 (-31.93, 5.16)	7.7 (-7.66, 23.03)	35.29 (20.5, 50.12)	-24.43 (-37.74, -11.13)	-12.63 (-26.79, 1.52)	-7.55 (-30.3, 15.14)	34.98 (18.27, 51.89)	9.21 (-9.51, 28.06)	Cotadutide

Table S8.6: The league table of SBP

**Dulaglutide**	-0.9 (-2.04, 0.23)	-1.43 (-2.69, -0.16)	1.07 (-1.25, 3.39)	-1.58 (-2.78, -0.38)	1.89 (0.95, 2.83)	1.41 (0.46, 2.37)	0.1 (-3.77, 3.98)	-6.56 (-8.12, -5.02)
0.9 (-0.23, 2.04)	**Semaglutide**	-0.52 (-1.95, 0.9)	1.98 (-0.4, 4.37)	-0.68 (-2.05, 0.69)	2.8 (1.7, 3.89)	2.32 (1.24, 3.39)	1 (-2.92, 4.93)	-5.66 (-7.47, -3.84)
1.43 (0.16, 2.69)	0.52 (-0.9, 1.95)	**Exenatide**	2.5 (0.1, 4.9)	-0.16 (-1.52, 1.21)	3.32 (2.27, 4.36)	2.84 (1.52, 4.15)	1.52 (-2.37, 5.43)	-5.14 (-7, -3.29)
-1.07 (-3.39, 1.25)	-1.98 (-4.37, 0.4)	-2.5 (-4.9, -0.1)	**Polyethylene Glycol Loxenatide**	-2.66 (-5.05, -0.27)	0.81 (-1.38, 3.02)	0.33 (-1.95, 2.62)	-0.97 (-5.36, 3.37)	-7.64 (-10.32, -4.95)
1.58 (0.38, 2.78)	0.68 (-0.69, 2.05)	0.16 (-1.21, 1.52)	2.66 (0.27, 5.05)	**Liraglutide**	3.47 (2.42, 4.52)	2.99 (1.79, 4.19)	1.68 (-2.21, 5.59)	-4.98 (-6.81, -3.17)
-1.89 (-2.83, -0.95)	-2.8 (-3.89, -1.7)	-3.32 (-4.36, -2.27)	-0.81 (-3.02, 1.38)	-3.47 (-4.52, -2.42)	**Placebo control groups**	-0.48 (-1.53, 0.57)	-1.8 (-5.56, 1.96)	-8.46 (-10.05, -6.86)
-1.41 (-2.37, -0.46)	-2.32 (-3.39, -1.24)	-2.84 (-4.15, -1.52)	-0.33 (-2.62, 1.95)	-2.99 (-4.19, -1.79)	0.48 (-0.57, 1.53)	**other control groups**	-1.31 (-5.21, 2.59)	-7.98 (-9.71, -6.24)
-0.1 (-3.98, 3.77)	-1 (-4.93, 2.92)	-1.52 (-5.43, 2.37)	0.97 (-3.37, 5.36)	-1.68 (-5.59, 2.21)	1.8 (-1.96, 5.56)	1.31 (-2.59, 5.21)	**Visepegenatide**	-6.66 (-10.74, -2.59)
6.56 (5.02, 8.12)	5.66 (3.84, 7.47)	5.14 (3.29, 7)	7.64 (4.95, 10.32)	4.98 (3.17, 6.81)	8.46 (6.86, 10.05)	7.98 (6.24, 9.71)	6.66 (2.59, 10.74)	**Orforglipro**

Table S8.7: The league table of DBP

**Dulaglutide**	-0.41 (-1.34, 0.51)	-0.1 (-1.15, 0.94)	-0.43 (-2.31, 1.46)	-0.12 (-1.09, 0.84)	0.56 (-0.19, 1.31)	-0.05 (-0.82, 0.73)	-1.54 (-4.72, 1.63)	-0.95 (-2.21, 0.3)
0.41 (-0.51, 1.34)	**Semaglutide**	0.31 (-0.88, 1.49)	-0.02 (-1.95, 1.93)	0.29 (-0.83, 1.42)	0.97 (0.07, 1.87)	0.37 (-0.51, 1.24)	-1.13 (-4.34, 2.08)	-0.54 (-2, 0.93)
0.1 (-0.94, 1.15)	-0.31 (-1.49, 0.88)	**Exenatide**	-0.32 (-2.29, 1.66)	-0.02 (-1.16, 1.12)	0.66 (-0.22, 1.56)	0.06 (-1.03, 1.15)	-1.44 (-4.65, 1.77)	-0.85 (-2.34, 0.65)
0.43 (-1.46, 2.31)	0.02 (-1.93, 1.95)	0.32 (-1.66, 2.29)	**Polyethylene Glycol Loxenatide**	0.3 (-1.65, 2.24)	0.99 (-0.82, 2.78)	0.38 (-1.49, 2.24)	-1.12 (-4.68, 2.45)	-0.52 (-2.7, 1.64)
0.12 (-0.84, 1.09)	-0.29 (-1.42, 0.83)	0.02 (-1.12, 1.16)	-0.3 (-2.24, 1.65)	**Liraglutide**	0.68 (-0.17, 1.54)	0.08 (-0.9, 1.05)	-1.42 (-4.62, 1.78)	-0.83 (-2.29, 0.63)
-0.56 (-1.31, 0.19)	-0.97 (-1.87, -0.07)	-0.66 (-1.56, 0.22)	-0.99 (-2.78, 0.82)	-0.68 (-1.54, 0.17)	**Placebo control groups**	-0.6 (-1.47, 0.25)	-2.1 (-5.18, 0.98)	-1.51 (-2.77, -0.25)
0.05 (-0.73, 0.82)	-0.37 (-1.24, 0.51)	-0.06 (-1.15, 1.03)	-0.38 (-2.24, 1.49)	-0.08 (-1.05, 0.9)	0.6 (-0.25, 1.47)	**Placebo control groups**	-1.5 (-4.69, 1.7)	-0.9 (-2.31, 0.5)
1.54 (-1.63, 4.72)	1.13 (-2.08, 4.34)	1.44 (-1.77, 4.65)	1.12 (-2.45, 4.68)	1.42 (-1.78, 4.62)	2.1 (-0.98, 5.18)	1.5 (-1.7, 4.69)	**Visepegenatide**	0.6 (-2.73, 3.92)
0.95 (-0.3, 2.21)	0.54 (-0.93, 2)	0.85 (-0.65, 2.34)	0.52 (-1.64, 2.7)	0.83 (-0.63, 2.29)	1.51 (0.25, 2.77)	0.9 (-0.5, 2.31)	-0.6 (-3.92, 2.73)	**Orforglipron**

Table S8.8: The league table of HDL

**Dulaglutide**	0.01 (-0.04, 0.06)	0.03 (-0.02, 0.09)	0.05 (-0.02, 0.12)	0.08 (0.03, 0.13)	0.05 (0.01, 0.09)	0.05 (-0.01, 0.11)	0.05 (0.01, 0.09)	0.06 (0, 0.13)
-0.01 (-0.06, 0.04)	**Semaglutide**	0.03 (-0.03, 0.08)	0.04 (-0.03, 0.11)	0.07 (0.02, 0.12)	0.04 (0, 0.08)	0.04 (-0.01, 0.09)	0.04 (-0.01, 0.1)	0.06 (-0.01, 0.12)
-0.03 (-0.09, 0.02)	-0.03 (-0.08, 0.03)	**Exenatide**	0.02 (-0.05, 0.08)	0.04 (0, 0.09)	0.02 (-0.02, 0.05)	0.01 (-0.03, 0.06)	0.01 (-0.04, 0.07)	0.03 (-0.03, 0.09)
-0.05 (-0.12, 0.02)	-0.04 (-0.11, 0.03)	-0.02 (-0.08, 0.05)	**Polyethylene Glycol Loxenatide**	0.03 (-0.03, 0.09)	0 (-0.06, 0.06)	0 (-0.06, 0.06)	0 (-0.07, 0.07)	0.02 (-0.06, 0.09)
-0.08 (-0.13, -0.03)	-0.07 (-0.12, -0.02)	-0.04 (-0.09, 0)	-0.03 (-0.09, 0.03)	**Liraglutide**	-0.03 (-0.06, 0)	-0.03 (-0.08, 0.01)	-0.03 (-0.08, 0.02)	-0.01 (-0.06, 0.04)
-0.05 (-0.09, -0.01)	-0.04 (-0.08, 0)	-0.02 (-0.05, 0.02)	0 (-0.06, 0.06)	0.03 (0, 0.06)	**Placebo control group**	0 (-0.05, 0.04)	0 (-0.04, 0.04)	0.01 (-0.04, 0.06)
-0.05 (-0.11, 0.01)	-0.04 (-0.09, 0.01)	-0.01 (-0.06, 0.03)	0 (-0.06, 0.06)	0.03 (-0.01, 0.08)	0 (-0.04, 0.05)	**other control groups**	0 (-0.06, 0.06)	0.02 (-0.05, 0.08)
-0.05 (-0.09, -0.01)	-0.04 (-0.1, 0.01)	-0.01 (-0.07, 0.04)	0 (-0.07, 0.07)	0.03 (-0.02, 0.08)	0 (-0.04, 0.04)	0 (-0.06, 0.06)	**Orforglipron**	0.02 (-0.05, 0.08)
-0.06 (-0.13, 0)	-0.06 (-0.12, 0.01)	-0.03 (-0.09, 0.03)	-0.02 (-0.09, 0.06)	0.01 (-0.04, 0.06)	-0.01 (-0.06, 0.04)	-0.02 (-0.08, 0.05)	-0.02 (-0.08, 0.05)	**Cotadutide**

Table S8.9: The league table of LDL

**Dulaglutide**	-0.22 (-0.39, -0.05)	-0.24 (-0.42, -0.07)	-0.1 (-0.33, 0.12)	-0.12 (-0.29, 0.04)	-0.1 (-0.23, 0.03)	-0.07 (-0.26, 0.13)	-0.23 (-0.57, 0.11)	-0.28 (-0.42, -0.15)	-0.2 (-0.4, 0.01)
0.22 (0.05, 0.39)	**Semaglutide**	-0.03 (-0.2, 0.14)	0.12 (-0.11, 0.34)	0.09 (-0.07, 0.26)	0.12 (-0.01, 0.25)	0.15 (-0.03, 0.33)	-0.01 (-0.35, 0.33)	-0.07 (-0.25, 0.12)	0.02 (-0.19, 0.23)
0.24 (0.07, 0.42)	0.03 (-0.14, 0.2)	**Exenatide**	0.14 (-0.07, 0.35)	0.12 (-0.02, 0.26)	0.15 (0.03, 0.26)	0.18 (0.02, 0.33)	0.02 (-0.32, 0.35)	-0.04 (-0.22, 0.14)	0.05 (-0.15, 0.24)
0.1 (-0.12, 0.33)	-0.12 (-0.34, 0.11)	-0.14 (-0.35, 0.07)	**Polyethylene Glycol Loxenatide**	-0.02 (-0.23, 0.19)	0 (-0.18, 0.19)	0.03 (-0.17, 0.24)	-0.13 (-0.49, 0.23)	-0.18 (-0.41, 0.05)	-0.09 (-0.34, 0.15)
0.12 (-0.04, 0.29)	-0.09 (-0.26, 0.07)	-0.12 (-0.26, 0.02)	0.02 (-0.19, 0.23)	**Liraglutide**	0.02 (-0.08, 0.13)	0.05 (-0.1, 0.21)	-0.11 (-0.43, 0.22)	-0.16 (-0.33, 0.01)	-0.07 (-0.24, 0.09)
0.1 (-0.03, 0.23)	-0.12 (-0.25, 0.01)	-0.15 (-0.26, -0.03)	0 (-0.19, 0.18)	-0.02 (-0.13, 0.08)	**Placebo control groups**	0.03 (-0.12, 0.18)	-0.13 (-0.44, 0.18)	-0.18 (-0.32, -0.05)	-0.1 (-0.26, 0.07)
0.07 (-0.13, 0.26)	-0.15 (-0.33, 0.03)	-0.18 (-0.33, -0.02)	-0.03 (-0.24, 0.17)	-0.05 (-0.21, 0.1)	-0.03 (-0.18, 0.12)	**other control groups**	-0.16 (-0.51, 0.18)	-0.22 (-0.41, -0.02)	-0.13 (-0.34, 0.08)
0.23 (-0.11, 0.57)	0.01 (-0.33, 0.35)	-0.02 (-0.35, 0.32)	0.13 (-0.23, 0.49)	0.11 (-0.22, 0.43)	0.13 (-0.18, 0.44)	0.16 (-0.18, 0.51)	**Visepegenatide**	-0.05 (-0.39, 0.29)	0.03 (-0.32, 0.38)
0.28 (0.15, 0.42)	0.07 (-0.12, 0.25)	0.04 (-0.14, 0.22)	0.18 (-0.05, 0.41)	0.16 (-0.01, 0.33)	0.18 (0.05, 0.32)	0.22 (0.02, 0.41)	0.05 (-0.29, 0.39)	**Orforglipron**	0.09 (-0.13, 0.3)
0.2 (-0.01, 0.4)	-0.02 (-0.23, 0.19)	-0.05 (-0.24, 0.15)	0.09 (-0.15, 0.34)	0.07 (-0.09, 0.24)	0.1 (-0.07, 0.26)	0.13 (-0.08, 0.34)	-0.03 (-0.38, 0.32)	-0.09 (-0.3, 0.13)	**Cotadutide**

Table S8.10: The league table of TC

**Dulaglutide**	-0.01 (-0.15, 0.13)	-0.18 (-0.32, -0.05)	0.04 (-0.13, 0.2)	-0.04 (-0.16, 0.09)	0.06 (-0.04, 0.16)	0.05 (-0.1, 0.19)	-0.17 (-0.41, 0.07)	-0.1 (-0.19, 0)	-0.07 (-0.22, 0.08)
0.01 (-0.13, 0.15)	**Semaglutide**	-0.17 (-0.3, -0.05)	0.05 (-0.11, 0.21)	-0.02 (-0.14, 0.1)	0.07 (-0.03, 0.17)	0.06 (-0.07, 0.18)	-0.16 (-0.4, 0.08)	-0.08 (-0.23, 0.06)	-0.06 (-0.21, 0.09)
0.18 (0.05, 0.32)	0.17 (0.05, 0.3)	**Exenatide**	0.22 (0.07, 0.37)	0.15 (0.05, 0.25)	0.24 (0.16, 0.33)	0.23 (0.12, 0.34)	0.01 (-0.22, 0.25)	0.09 (-0.04, 0.22)	0.12 (-0.02, 0.25)
-0.04 (-0.2, 0.13)	-0.05 (-0.21, 0.11)	-0.22 (-0.37, -0.07)	**Polyethylene Glycol Loxenatide**	-0.07 (-0.22, 0.07)	0.02 (-0.11, 0.16)	0.01 (-0.13, 0.15)	-0.21 (-0.46, 0.05)	-0.13 (-0.3, 0.03)	-0.1 (-0.28, 0.07)
0.04 (-0.09, 0.16)	0.02 (-0.1, 0.14)	-0.15 (-0.25, -0.05)	0.07 (-0.07, 0.22)	**Liraglutide**	0.1 (0.02, 0.17)	0.08 (-0.02, 0.18)	-0.13 (-0.37, 0.1)	-0.06 (-0.18, 0.06)	-0.03 (-0.15, 0.08)
-0.06 (-0.16, 0.04)	-0.07 (-0.17, 0.03)	-0.24 (-0.33, -0.16)	-0.02 (-0.16, 0.11)	-0.1 (-0.17, -0.02)	**Placebo control groups**	-0.01 (-0.11, 0.08)	-0.23 (-0.45, -0.01)	-0.16 (-0.25, -0.06)	-0.13 (-0.25, -0.01)
-0.05 (-0.19, 0.1)	-0.06 (-0.18, 0.07)	-0.23 (-0.34, -0.12)	-0.01 (-0.15, 0.13)	-0.08 (-0.18, 0.02)	0.01 (-0.08, 0.11)	**other control groups**	-0.22 (-0.46, 0.03)	-0.14 (-0.28, 0)	-0.12 (-0.26, 0.03)
0.17 (-0.07, 0.41)	0.16 (-0.08, 0.4)	-0.01 (-0.25, 0.22)	0.21 (-0.05, 0.46)	0.13 (-0.1, 0.37)	0.23 (0.01, 0.45)	0.22 (-0.03, 0.46)	**Visepegenatide**	0.07 (-0.17, 0.32)	0.1 (-0.15, 0.35)
0.1 (0, 0.19)	0.08 (-0.06, 0.23)	-0.09 (-0.22, 0.04)	0.13 (-0.03, 0.3)	0.06 (-0.06, 0.18)	0.16 (0.06, 0.25)	0.14 (0, 0.28)	-0.07 (-0.32, 0.17)	**Orforglipron**	0.03 (-0.13, 0.18)
0.07 (-0.08, 0.22)	0.06 (-0.09, 0.21)	-0.12 (-0.25, 0.02)	0.1 (-0.07, 0.28)	0.03 (-0.08, 0.15)	0.13 (0.01, 0.25)	0.12 (-0.03, 0.26)	-0.1 (-0.35, 0.15)	-0.03 (-0.18, 0.13)	**Cotadutide**

Table S8.11: The league table of TG

**Dulaglutide**	0.36 (0.09, 0.62)	0.27 (-0.04, 0.58)	0.31 (-0.04, 0.66)	0.17 (-0.09, 0.44)	0.25 (0.04, 0.46)	0.26 (-0.03, 0.56)	-0.06 (-0.57, 0.46)	0.03 (-0.18, 0.24)	0.13 (-0.2, 0.45)
-0.36 (-0.62, -0.09)	**Semaglutide**	-0.09 (-0.38, 0.21)	-0.04 (-0.38, 0.29)	-0.18 (-0.43, 0.07)	-0.1 (-0.3, 0.1)	-0.09 (-0.36, 0.17)	-0.41 (-0.93, 0.1)	-0.33 (-0.61, -0.04)	-0.23 (-0.55, 0.09)
-0.27 (-0.58, 0.04)	0.09 (-0.21, 0.38)	**Exenatide**	0.05 (-0.3, 0.39)	-0.09 (-0.35, 0.17)	-0.01 (-0.24, 0.21)	0 (-0.25, 0.24)	-0.33 (-0.85, 0.2)	-0.24 (-0.56, 0.08)	-0.14 (-0.47, 0.19)
-0.31 (-0.66, 0.04)	0.04 (-0.29, 0.38)	-0.05 (-0.39, 0.3)	**Polyethylene Glycol Loxenatide**	-0.14 (-0.45, 0.17)	-0.06 (-0.34, 0.22)	-0.05 (-0.36, 0.26)	-0.37 (-0.92, 0.18)	-0.28 (-0.64, 0.07)	-0.19 (-0.56, 0.18)
-0.17 (-0.44, 0.09)	0.18 (-0.07, 0.43)	0.09 (-0.17, 0.35)	0.14 (-0.17, 0.45)	**Liraglutide**	0.08 (-0.08, 0.24)	0.09 (-0.12, 0.3)	-0.23 (-0.73, 0.27)	-0.15 (-0.42, 0.13)	-0.05 (-0.3, 0.2)
-0.25 (-0.46, -0.04)	0.1 (-0.1, 0.3)	0.01 (-0.21, 0.24)	0.06 (-0.22, 0.34)	-0.08 (-0.24, 0.08)	**Placebo control groups**	0.01 (-0.2, 0.23)	-0.31 (-0.78, 0.16)	-0.22 (-0.45, 0)	-0.13 (-0.38, 0.12)
-0.26 (-0.56, 0.03)	0.09 (-0.17, 0.36)	0 (-0.24, 0.25)	0.05 (-0.26, 0.36)	-0.09 (-0.3, 0.12)	-0.01 (-0.23, 0.2)	**other control group**	-0.32 (-0.84, 0.19)	-0.24 (-0.54, 0.07)	-0.14 (-0.45, 0.17)
0.06 (-0.46, 0.57)	0.41 (-0.1, 0.93)	0.33 (-0.2, 0.85)	0.37 (-0.18, 0.92)	0.23 (-0.27, 0.73)	0.31 (-0.16, 0.78)	0.32 (-0.19, 0.84)	**Visepegenatide**	0.09 (-0.44, 0.61)	0.18 (-0.35, 0.72)
-0.03 (-0.24, 0.18)	0.33 (0.04, 0.61)	0.24 (-0.08, 0.56)	0.28 (-0.07, 0.64)	0.15 (-0.13, 0.42)	0.22 (0, 0.45)	0.24 (-0.07, 0.54)	-0.09 (-0.61, 0.44)	**Orforglipron**	0.1 (-0.24, 0.43)
-0.13 (-0.45, 0.2)	0.23 (-0.09, 0.55)	0.14 (-0.19, 0.47)	0.19 (-0.18, 0.56)	0.05 (-0.2, 0.3)	0.13 (-0.12, 0.38)	0.14 (-0.17, 0.45)	-0.18 (-0.72, 0.35)	-0.1 (-0.43, 0.24)	**Cotadutide**

Table S8.12: The league table of MACE

**Placebo control groups**	-0.12 (-0.43, 0.17)	-0.25 (-0.46, -0.05)	-0.15 (-0.45, 0.14)	0.02 (-0.29, 0.33)	-0.3 (-0.65, 0.06)	-0.57 (-0.94, -0.23)	0.22 (-0.19, 0.63)	-0.28 (-0.53, -0.03)
0.12 (-0.17, 0.43)	**Dulaglutide**	-0.13 (-0.49, 0.24)	-0.03 (-0.45, 0.39)	0.15 (-0.28, 0.57)	-0.18 (-0.63, 0.29)	-0.44 (-0.91, 0)	0.34 (-0.16, 0.85)	-0.15 (-0.54, 0.24)
0.25 (0.05, 0.46)	0.13 (-0.24, 0.49)	**Semaglutide**	0.1 (-0.26, 0.46)	0.27 (-0.1, 0.65)	-0.05 (-0.46, 0.37)	-0.32 (-0.74, 0.08)	0.47 (0.01, 0.93)	-0.02 (-0.35, 0.3)
0.15 (-0.14, 0.45)	0.03 (-0.39, 0.45)	-0.1 (-0.46, 0.26)	**Liraglutide**	0.17 (-0.26, 0.6)	-0.15 (-0.61, 0.31)	-0.42 (-0.89, 0.02)	0.37 (-0.13, 0.88)	-0.12 (-0.51, 0.26)
-0.02 (-0.33, 0.29)	-0.15 (-0.57, 0.28)	-0.27 (-0.65, 0.1)	-0.17 (-0.6, 0.26)	**Lixisenatide**	-0.32 (-0.79, 0.15)	-0.59 (-1.06, -0.14)	0.2 (-0.31, 0.71)	-0.3 (-0.69, 0.1)
0.3 (-0.06, 0.65)	0.18 (-0.29, 0.63)	0.05 (-0.37, 0.46)	0.15 (-0.31, 0.61)	0.32 (-0.15, 0.79)	**Efpeglenatide**	-0.27 (-0.78, 0.21)	0.52 (-0.02, 1.06)	0.03 (-0.41, 0.46)
0.57 (0.23, 0.94)	0.44 (0, 0.91)	0.32 (-0.08, 0.74)	0.42 (-0.02, 0.89)	0.59 (0.14, 1.06)	0.27 (-0.21, 0.78)	**Tirzepatide**	0.79 (0.26, 1.35)	0.29 (-0.05, 0.67)
-0.22 (-0.63, 0.19)	-0.34 (-0.85, 0.16)	-0.47 (-0.93, -0.01)	-0.37 (-0.88, 0.13)	-0.2 (-0.71, 0.31)	-0.52 (-1.06, 0.02)	-0.79 (-1.35, -0.26)	**ITCA650**	-0.49 (-0.98, -0.02)
0.28 (0.03, 0.53)	0.15 (-0.24, 0.54)	0.02 (-0.3, 0.35)	0.12 (-0.26, 0.51)	0.3 (-0.1, 0.69)	-0.03 (-0.46, 0.41)	-0.29 (-0.67, 0.05)	0.49 (0.02, 0.98)	**Insulin glargine**

Table S8.13: The league table of NFS

**Placebo control groups**	-0.27 (-1.22, 0.69)	-0.14 (-0.77, 0.43)	-0.15 (-1.09, 0.79)	-0.11 (-1.07, 0.84)	0.1 (-0.89, 1.09)	-0.2 (-1.22, 0.83)	-0.37 (-1.29, 0.44)	0.01 (-1.06, 1.08)	-0.15 (-0.82, 0.52)
0.27 (-0.69, 1.22)	**Dulaglutide**	0.14 (-1.04, 1.22)	0.12 (-1.23, 1.46)	0.16 (-1.19, 1.5)	0.37 (-1.01, 1.75)	0.07 (-1.33, 1.48)	-0.1 (-1.42, 1.1)	0.28 (-1.16, 1.7)	0.12 (-1.04, 1.28)
0.14 (-0.43, 0.77)	-0.14 (-1.22, 1.04)	**Semaglutide**	-0.02 (-1.09, 1.14)	0.03 (-1.07, 1.18)	0.24 (-0.89, 1.44)	-0.06 (-1.21, 1.17)	-0.22 (-1.3, 0.78)	0.15 (-1.05, 1.41)	-0.01 (-0.88, 0.92)
0.15 (-0.79, 1.09)	-0.12 (-1.46, 1.23)	0.02 (-1.14, 1.09)	**Exenatide**	0.04 (-1.3, 1.38)	0.25 (-1.11, 1.62)	-0.05 (-1.43, 1.35)	-0.21 (-1.53, 0.98)	0.16 (-1.26, 1.57)	0 (-1.15, 1.15)
0.11 (-0.84, 1.07)	-0.16 (-1.5, 1.19)	-0.03 (-1.18, 1.07)	-0.04 (-1.38, 1.3)	**Liraglutide**	0.21 (-1.16, 1.6)	-0.09 (-1.48, 1.32)	-0.25 (-1.59, 0.96)	0.12 (-1.3, 1.54)	-0.04 (-1.19, 1.12)
-0.1 (-1.09, 0.89)	-0.37 (-1.75, 1.01)	-0.24 (-1.44, 0.89)	-0.25 (-1.62, 1.11)	-0.21 (-1.6, 1.16)	**Lixisenatide**	-0.3 (-1.72, 1.14)	-0.47 (-1.82, 0.77)	-0.1 (-1.56, 1.37)	-0.25 (-1.45, 0.95)
0.2 (-0.83, 1.22)	-0.07 (-1.48, 1.33)	0.06 (-1.17, 1.21)	0.05 (-1.35, 1.43)	0.09 (-1.32, 1.48)	0.3 (-1.14, 1.72)	**Efpeglenatide**	-0.17 (-1.56, 1.1)	0.2 (-1.28, 1.68)	0.05 (-1.18, 1.28)
0.37 (-0.44, 1.29)	0.1 (-1.1, 1.42)	0.22 (-0.78, 1.3)	0.21 (-0.98, 1.53)	0.25 (-0.96, 1.59)	0.47 (-0.77, 1.82)	0.17 (-1.1, 1.56)	**Tirzepatide**	0.38 (-0.94, 1.78)	0.22 (-0.59, 1.14)
-0.01 (-1.08, 1.06)	-0.28 (-1.7, 1.16)	-0.15 (-1.41, 1.05)	-0.16 (-1.57, 1.26)	-0.12 (-1.54, 1.3)	0.1 (-1.37, 1.56)	-0.2 (-1.68, 1.28)	-0.38 (-1.78, 0.94)	**ITCA650**	-0.16 (-1.42, 1.1)
0.15 (-0.52, 0.82)	-0.12 (-1.28, 1.04)	0.01 (-0.92, 0.88)	0 (-1.15, 1.15)	0.04 (-1.12, 1.19)	0.25 (-0.95, 1.45)	-0.05 (-1.28, 1.18)	-0.22 (-1.14, 0.59)	0.16 (-1.1, 1.42)	**Insulin glargine**

Table S8.14: The league table of NFM

**Placebo control groups**	-0.04 (-0.62, 0.55)	-0.15 (-0.52, 0.25)	-0.02 (-0.59, 0.56)	-0.13 (-0.71, 0.45)	0.04 (-0.54, 0.62)	-0.23 (-0.87, 0.41)	-0.48 (-1.11, 0.07)	0.29 (-0.44, 1.02)	-0.15 (-0.58, 0.28)
0.04 (-0.55, 0.62)	**Dulaglutide**	-0.12 (-0.8, 0.61)	0.02 (-0.8, 0.84)	-0.09 (-0.91, 0.73)	0.07 (-0.75, 0.9)	-0.19 (-1.06, 0.67)	-0.44 (-1.29, 0.33)	0.32 (-0.61, 1.25)	-0.11 (-0.84, 0.61)
0.15 (-0.25, 0.52)	0.12 (-0.61, 0.8)	**Semaglutide**	0.14 (-0.58, 0.8)	0.03 (-0.69, 0.7)	0.19 (-0.52, 0.86)	-0.08 (-0.84, 0.65)	-0.33 (-1.07, 0.33)	0.44 (-0.4, 1.24)	0 (-0.59, 0.57)
0.02 (-0.56, 0.59)	-0.02 (-0.84, 0.8)	-0.14 (-0.8, 0.58)	**Exenatide**	-0.11 (-0.92, 0.7)	0.05 (-0.76, 0.87)	-0.21 (-1.07, 0.65)	-0.46 (-1.3, 0.3)	0.3 (-0.61, 1.22)	-0.13 (-0.85, 0.58)
0.13 (-0.45, 0.71)	0.09 (-0.73, 0.91)	-0.03 (-0.7, 0.69)	0.11 (-0.7, 0.92)	**Liraglutide**	0.16 (-0.66, 0.99)	-0.1 (-0.96, 0.76)	-0.35 (-1.19, 0.42)	0.42 (-0.51, 1.33)	-0.02 (-0.74, 0.69)
-0.04 (-0.62, 0.54)	-0.07 (-0.9, 0.75)	-0.19 (-0.86, 0.52)	-0.05 (-0.87, 0.76)	-0.16 (-0.99, 0.66)	**Lixisenatide**	-0.26 (-1.13, 0.6)	-0.51 (-1.36, 0.26)	0.25 (-0.67, 1.17)	-0.18 (-0.91, 0.53)
0.23 (-0.41, 0.87)	0.19 (-0.67, 1.06)	0.08 (-0.65, 0.84)	0.21 (-0.65, 1.07)	0.1 (-0.76, 0.96)	0.26 (-0.6, 1.13)	**Efpeglenatide**	-0.25 (-1.15, 0.57)	0.52 (-0.45, 1.48)	0.08 (-0.68, 0.84)
0.48 (-0.07, 1.11)	0.44 (-0.33, 1.29)	0.33 (-0.33, 1.07)	0.46 (-0.3, 1.3)	0.35 (-0.42, 1.19)	0.51 (-0.26, 1.36)	0.25 (-0.57, 1.15)	**Tirzepatide**	0.77 (-0.13, 1.73)	0.33 (-0.22, 0.96)
-0.29 (-1.02, 0.44)	-0.32 (-1.25, 0.61)	-0.44 (-1.24, 0.4)	-0.3 (-1.22, 0.61)	-0.42 (-1.33, 0.51)	-0.25 (-1.17, 0.67)	-0.52 (-1.48, 0.45)	-0.77 (-1.73, 0.13)	**ITCA650**	-0.44 (-1.28, 0.41)
0.15 (-0.28, 0.58)	0.11 (-0.61, 0.84)	0 (-0.57, 0.59)	0.13 (-0.58, 0.85)	0.02 (-0.69, 0.74)	0.18 (-0.53, 0.91)	-0.08 (-0.84, 0.68)	-0.33 (-0.96, 0.22)	0.44 (-0.41, 1.28)	**Insulin glargine**

Table S8.15: The league table of CVM

**Placebo control groups**	-0.09 (-1.19, 0.98)	-0.34 (-0.92, 0.21)	-0.12 (-1.22, 0.96)	-0.25 (-1.33, 0.85)	-0.05 (-1.16, 1.05)	-0.3 (-1.41, 0.84)	-0.45 (-2.33, 1.31)	0.2 (-0.97, 1.38)	-0.09 (-1.68, 1.57)
0.09 (-0.98, 1.19)	**Dulaglutide**	-0.24 (-1.48, 0.98)	-0.03 (-1.57, 1.5)	-0.16 (-1.71, 1.39)	0.04 (-1.52, 1.59)	-0.21 (-1.75, 1.37)	-0.36 (-1.9, 1.12)	0.3 (-1.3, 1.89)	0 (-1.21, 1.29)
0.34 (-0.21, 0.92)	0.24 (-0.98, 1.48)	**Semaglutide**	0.21 (-1, 1.45)	0.09 (-1.13, 1.34)	0.28 (-0.94, 1.54)	0.04 (-1.19, 1.31)	-0.1 (-2.08, 1.75)	0.54 (-0.75, 1.86)	0.25 (-1.43, 2.01)
0.12 (-0.96, 1.22)	0.03 (-1.5, 1.57)	-0.21 (-1.45, 1)	**Exenatide**	-0.13 (-1.66, 1.41)	0.07 (-1.49, 1.61)	-0.18 (-1.73, 1.4)	-0.32 (-2.52, 1.71)	0.32 (-1.27, 1.92)	0.03 (-1.87, 2.01)
0.25 (-0.85, 1.33)	0.16 (-1.39, 1.71)	-0.09 (-1.34, 1.13)	0.13 (-1.41, 1.66)	**Liraglutide**	0.2 (-1.35, 1.75)	-0.05 (-1.6, 1.52)	-0.19 (-2.4, 1.83)	0.46 (-1.15, 2.05)	0.16 (-1.75, 2.15)
0.05 (-1.05, 1.16)	-0.04 (-1.59, 1.52)	-0.28 (-1.54, 0.94)	-0.07 (-1.61, 1.49)	-0.2 (-1.75, 1.35)	**Lixisenatide**	-0.25 (-1.81, 1.35)	-0.39 (-2.61, 1.65)	0.26 (-1.36, 1.86)	-0.04 (-1.96, 1.95)
0.3 (-0.84, 1.41)	0.21 (-1.37, 1.75)	-0.04 (-1.31, 1.19)	0.18 (-1.4, 1.73)	0.05 (-1.52, 1.6)	0.25 (-1.35, 1.81)	**Efpeglenatide**	-0.14 (-2.35, 1.9)	0.5 (-1.12, 2.11)	0.2 (-1.73, 2.2)
0.45 (-1.31, 2.33)	0.36 (-1.12, 1.9)	0.1 (-1.75, 2.08)	0.32 (-1.71, 2.52)	0.19 (-1.83, 2.4)	0.39 (-1.65, 2.61)	0.14 (-1.9, 2.35)	**Tirzepatide**	0.64 (-1.45, 2.89)	0.34 (-0.38, 1.33)
-0.2 (-1.38, 0.97)	-0.3 (-1.89, 1.3)	-0.54 (-1.86, 0.75)	-0.32 (-1.92, 1.27)	-0.46 (-2.05, 1.15)	-0.26 (-1.86, 1.36)	-0.5 (-2.11, 1.12)	-0.64 (-2.89, 1.45)	**ITCA650**	-0.29 (-2.25, 1.74)
0.09 (-1.57, 1.68)	0 (-1.29, 1.21)	-0.25 (-2.01, 1.43)	-0.03 (-2.01, 1.87)	-0.16 (-2.15, 1.75)	0.04 (-1.95, 1.96)	-0.2 (-2.2, 1.73)	-0.34 (-1.33, 0.38)	0.29 (-1.74, 2.25)	**Insulin glargine**

Table S8.16: The league table of ACM

**Placebo control groups**	-0.11 (-1.37, 1.16)	-0.26 (-0.93, 0.37)	-0.15 (-1.4, 1.11)	-0.17 (-1.44, 1.09)	-0.54 (-2.5, 1.33)	0.19 (-1.12, 1.49)	-0.1 (-1.75, 1.69)
0.11 (-1.16, 1.37)	**Dulaglutide**	-0.14 (-1.6, 1.25)	-0.04 (-1.82, 1.75)	-0.06 (-1.84, 1.72)	-0.43 (-1.93, 1.01)	0.3 (-1.51, 2.1)	0.02 (-1.14, 1.27)
0.26 (-0.37, 0.93)	0.14 (-1.25, 1.6)	**Semaglutide**	0.11 (-1.28, 1.55)	0.08 (-1.31, 1.51)	-0.28 (-2.34, 1.72)	0.45 (-1, 1.93)	0.16 (-1.6, 2.09)
0.15 (-1.11, 1.4)	0.04 (-1.75, 1.82)	-0.11 (-1.55, 1.28)	**Exenatide**	-0.03 (-1.8, 1.76)	-0.39 (-2.74, 1.84)	0.33 (-1.47, 2.15)	0.05 (-2.02, 2.23)
0.17 (-1.09, 1.44)	0.06 (-1.72, 1.84)	-0.08 (-1.51, 1.31)	0.03 (-1.76, 1.8)	**Liraglutide**	-0.37 (-2.69, 1.88)	0.36 (-1.45, 2.18)	0.08 (-1.99, 2.27)
0.54 (-1.33, 2.5)	0.43 (-1.01, 1.93)	0.28 (-1.72, 2.34)	0.39 (-1.84, 2.74)	0.37 (-1.88, 2.69)	**Tirzepatide**	0.72 (-1.54, 3.09)	0.44 (-0.31, 1.4)
-0.19 (-1.49, 1.12)	-0.3 (-2.1, 1.51)	-0.45 (-1.93, 1)	-0.33 (-2.15, 1.47)	-0.36 (-2.18, 1.45)	-0.72 (-3.09, 1.54)	**ITCA650**	-0.28 (-2.37, 1.93)
0.1 (-1.69, 1.75)	-0.02 (-1.27, 1.14)	-0.16 (-2.09, 1.6)	-0.05 (-2.23, 2.02)	-0.08 (-2.27, 1.99)	-0.44 (-1.4, 0.31)	0.28 (-1.93, 2.37)	**Insulin glargine**

Table S8.17: The league table of eGFR

**Placebo control groups**	-1.12 (-4.76, 2.53)	-0.14 (-1.87, 1.58)	1.83 (-5.35, 9)	2.31 (-4.48, 9.15)	0.32 (-2.57, 3.23)	-0.21 (-2.85, 2.44)	-2.71 (-7.83, 2.38)	3.23 (-1.36, 7.83)
1.12 (-2.53, 4.76)	**Dulaglutide**	0.97 (-2.69, 4.62)	2.95 (-3.2, 9.12)	3.43 (-4.29, 11.21)	1.44 (-3.22, 6.12)	0.91 (-3.43, 5.29)	-1.6 (-5.15, 1.94)	4.34 (-1.56, 10.23)
0.14 (-1.58, 1.87)	-0.97 (-4.62, 2.69)	**Semaglutide**	1.99 (-5.21, 9.11)	2.45 (-4.56, 9.5)	0.47 (-2.89, 3.85)	-0.06 (-2.71, 2.6)	-2.57 (-7.68, 2.52)	3.37 (-1.54, 8.29)
-1.83 (-9, 5.35)	-2.95 (-9.12, 3.2)	-1.99 (-9.11, 5.21)	**Exenatide**	0.49 (-9.4, 10.41)	-1.51 (-9.24, 6.21)	-2.05 (-9.56, 5.51)	-4.56 (-9.58, 0.49)	1.41 (-7.1, 9.95)
-2.31 (-9.15, 4.48)	-3.43 (-11.21, 4.29)	-2.45 (-9.5, 4.56)	-0.49 (-10.41, 9.4)	**Polyethylene Glycol Loxenatide**	-1.99 (-8.17, 4.16)	-2.52 (-9.84, 4.8)	-5.05 (-13.55, 3.51)	0.92 (-4.13, 5.91)
-0.32 (-3.23, 2.57)	-1.44 (-6.12, 3.22)	-0.47 (-3.85, 2.89)	1.51 (-6.21, 9.24)	1.99 (-4.16, 8.17)	**Liraglutide**	-0.53 (-4.47, 3.4)	-3.05 (-8.9, 2.81)	2.9 (-0.65, 6.46)
0.21 (-2.44, 2.85)	-0.91 (-5.29, 3.43)	0.06 (-2.6, 2.71)	2.05 (-5.51, 9.56)	2.52 (-4.8, 9.84)	0.53 (-3.4, 4.47)	**Cotadutide**	-2.51 (-8.14, 3.09)	3.43 (-1.87, 8.75)
2.71 (-2.38, 7.83)	1.6 (-1.94, 5.15)	2.57 (-2.52, 7.68)	4.56 (-0.49, 9.58)	5.05 (-3.51, 13.55)	3.05 (-2.81, 8.9)	2.51 (-3.09, 8.14)	**Insulin glargine**	5.96 (-0.94, 12.82)
-3.23 (-7.83, 1.36)	-4.34 (-10.23, 1.56)	-3.37 (-8.29, 1.54)	-1.41 (-9.95, 7.1)	-0.92 (-5.91, 4.13)	-2.9 (-6.46, 0.65)	-3.43 (-8.75, 1.87)	-5.96 (-12.82, 0.94)	**Dapagliflozin**

Table S8.18: The league table of FIS

Placebo control groups	-0.97 (-8.08, 6.12)	-4.12 (-10.82, 2.6)	-0.58 (-20.7, 19.46)	2.9 (-7.18, 12.99)	2.72 (-19.83, 25.2)	-23.09 (-32.48, -13.73)	1.08 (-8.32, 10.48)	9.83 (1.98, 17.72)	7.18 (-7.05, 21.41)	15.09 (1.56, 28.51)	-6.09 (-26.11, 14.09)	20.72 (3.4, 38.06)	-5.8 (-28.19, 16.7)
0.97 (-6.12, 8.08)	Dulaglutide	-3.14 (-12.95, 6.61)	0.41 (-21, 21.72)	3.88 (-8.47, 16.14)	3.68 (-20.02, 27.29)	-22.13 (-31.55, -12.66)	2.06 (-7.36, 11.43)	10.81 (0.25, 21.4)	8.14 (-7.75, 24.02)	16.07 (0.76, 31.21)	-5.15 (-26.42, 16.24)	21.69 (3, 40.45)	-4.83 (-28.36, 18.73)
4.12 (-2.6, 10.82)	3.14 (-6.61, 12.95)	Semaglutide	3.53 (-17.59, 24.66)	7.02 (-5.11, 19.11)	6.82 (-16.73, 30.33)	-18.99 (-30.52, -7.45)	5.19 (-6.37, 16.76)	13.94 (6.12, 21.84)	11.27 (-4.47, 26.97)	19.19 (4.11, 34.19)	-2.02 (-23.08, 19.18)	24.83 (6.17, 43.51)	-1.69 (-25.14, 21.89)
0.58 (-19.46, 20.7)	-0.41 (-21.72, 21)	-3.53 (-24.66, 17.59)	Exenatide	3.5 (-18.98, 26)	3.33 (-26.87, 33.59)	-22.5 (-44.64, -0.31)	1.65 (-20.48, 23.85)	10.41 (-11.08, 31.98)	7.75 (-16.75, 32.42)	15.67 (-8.55, 39.83)	-5.49 (-25.6, 14.61)	21.33 (-5.19, 47.93)	-5.21 (-35.32, 24.97)
-2.9 (-12.99, 7.18)	-3.88 (-16.14, 8.47)	-7.02 (-19.11, 5.11)	-3.5 (-26, 18.98)	Liraglutide	-0.17 (-20.34, 19.91)	-26.01 (-39.77, -12.2)	-1.85 (-15.63, 11.99)	6.95 (-5.88, 19.71)	4.28 (-13.15, 21.69)	12.17 (3.16, 21.13)	-9.02 (-31.49, 13.56)	17.81 (3.6, 32.03)	-8.71 (-28.79, 11.43)
-2.72 (-25.2, 19.83)	-3.68 (-27.29, 20.02)	-6.82 (-30.33, 16.73)	-3.33 (-33.59, 26.87)	0.17 (-19.91, 20.34)	Lixisenatide	-25.85 (-50.18, -1.38)	-1.63 (-25.99, 22.75)	7.13 (-16.64, 30.96)	4.48 (-22.21, 31.01)	12.36 (-9.69, 34.32)	-8.79 (-39.11, 21.37)	18.03 (-6.69, 42.59)	-8.5 (-36.96, 19.99)
23.09 (13.73, 32.48)	22.13 (12.66, 31.55)	18.99 (7.45, 30.52)	22.5 (0.31, 44.64)	26.01 (12.2, 39.77)	25.85 (1.38, 50.18)	Tirzepatide	24.18 (11.83, 36.46)	32.95 (20.68, 45.16)	30.28 (13.17, 47.3)	38.18 (21.73, 54.56)	16.99 (-5.15, 39.21)	43.84 (24.07, 63.56)	17.26 (-6.97, 41.54)
-1.08 (-10.48, 8.32)	-2.06 (-11.43, 7.36)	-5.19 (-16.76, 6.37)	-1.65 (-23.85, 20.48)	1.85 (-11.99, 15.63)	1.63 (-22.75, 25.99)	-24.18 (-36.46, -11.83)	Retatrutide	8.76 (-3.49, 21.04)	6.11 (-10.96, 23.08)	13.99 (-2.41, 30.39)	-7.14 (-29.41, 15.07)	19.64 (-0.06, 39.46)	-6.91 (-31.18, 17.54)
-9.83 (-17.72, -1.98)	-10.81 (-21.4, -0.25)	-13.94 (-21.84, -6.12)	-10.41 (-31.98, 11.08)	-6.95 (-19.71, 5.88)	-7.13 (-30.96, 16.64)	-32.95 (-45.16, -20.68)	-8.76 (-21.04, 3.49)	Survodutide	-2.66 (-18.91, 13.59)	5.26 (-10.4, 20.81)	-15.96 (-37.43, 5.59)	10.89 (-8.08, 30.06)	-15.62 (-39.34, 8.28)
-7.18 (-21.41, 7.05)	-8.14 (-24.02, 7.75)	-11.27 (-26.97, 4.47)	-7.75 (-32.42, 16.75)	-4.28 (-21.69, 13.15)	-4.48 (-31.01, 22.21)	-30.28 (-47.3, -13.17)	-6.11 (-23.08, 10.96)	2.66 (-13.59, 18.91)	Visepegenatide	7.9 (-11.61, 27.47)	-13.26 (-37.88, 11.43)	13.55 (-8.83, 36.11)	-13 (-39.45, 13.67)
-15.09 (-28.51, -1.56)	-16.07 (-31.21, -0.76)	-19.19 (-34.19, -4.11)	-15.67 (-39.83, 8.55)	-12.17 (-21.13, -3.16)	-12.36 (-34.32, 9.69)	-38.18 (-54.56, -21.73)	-13.99 (-30.39, 2.41)	-5.26 (-20.81, 10.4)	-7.9 (-27.47, 11.61)	Glimepiride	-21.16 (-45.45, 3.11)	5.67 (-11.16, 22.49)	-20.84 (-42.97, 1.12)
6.09 (-14.09, 26.11)	5.15 (-16.24, 26.42)	2.02 (-19.18, 23.08)	5.49 (-14.61, 25.6)	9.02 (-13.56, 31.49)	8.79 (-21.37, 39.11)	-16.99 (-39.21, 5.15)	7.14 (-15.07, 29.41)	15.96 (-5.59, 37.43)	13.26 (-11.43, 37.88)	21.16 (-3.11, 45.45)	Sitagliptin	26.81 (0.26, 53.4)	0.31 (-30.06, 30.56)
-20.72 (-38.06, -3.4)	-21.69 (-40.45, -3)	-24.83 (-43.51, -6.17)	-21.33 (-47.93, 5.19)	-17.81 (-32.03, -3.6)	-18.03 (-42.59, 6.69)	-43.84 (-63.56, -24.07)	-19.64 (-39.46, 0.06)	-10.89 (-30.06, 8.08)	-13.55 (-36.11, 8.83)	-5.67 (-22.49, 11.16)	-26.81 (-53.4, -0.26)	Sulfonylureas	-26.52 (-51.01, -1.91)
5.8 (-16.7, 28.19)	4.83 (-18.73, 28.36)	1.69 (-21.89, 25.14)	5.21 (-24.97, 35.32)	8.71 (-11.43, 28.79)	8.5 (-19.99, 36.96)	-17.26 (-41.54, 6.97)	6.91 (-17.54, 31.18)	15.62 (-8.28, 39.34)	13 (-13.67, 39.45)	20.84 (-1.12, 42.97)	-0.31 (-30.56, 30.06)	26.52 (1.91, 51.01)	Dapagliflozin

Table S8.19: The league table of C-peptide

**Placebo control groups**	0.17 (0.03, 0.31)	0.12 (0.02, 0.22)	0.06 (-0.09, 0.22)	-0.01 (-0.35, 0.34)	-0.2 (-0.36, -0.05)	0.1 (-0.02, 0.21)	0.06 (-0.16, 0.27)	0.15 (-0.06, 0.35)	0.06 (-0.21, 0.32)
-0.17 (-0.31, -0.03)	**Dulaglutide**	-0.05 (-0.21, 0.11)	-0.11 (-0.32, 0.1)	-0.18 (-0.55, 0.19)	-0.38 (-0.53, -0.23)	-0.08 (-0.26, 0.1)	-0.12 (-0.37, 0.14)	-0.03 (-0.27, 0.23)	-0.11 (-0.41, 0.19)
-0.12 (-0.22, -0.02)	0.05 (-0.11, 0.21)	**Semaglutide**	-0.06 (-0.24, 0.13)	-0.13 (-0.49, 0.23)	-0.32 (-0.5, -0.15)	-0.03 (-0.14, 0.09)	-0.06 (-0.3, 0.17)	0.03 (-0.2, 0.26)	-0.06 (-0.35, 0.22)
-0.06 (-0.22, 0.09)	0.11 (-0.1, 0.32)	0.06 (-0.13, 0.24)	**Liraglutide**	-0.07 (-0.38, 0.24)	-0.27 (-0.48, -0.05)	0.03 (-0.16, 0.23)	-0.01 (-0.27, 0.26)	0.08 (-0.05, 0.22)	-0.01 (-0.22, 0.21)
0.01 (-0.34, 0.35)	0.18 (-0.19, 0.55)	0.13 (-0.23, 0.49)	0.07 (-0.24, 0.38)	**Lixisenatide**	-0.2 (-0.57, 0.18)	0.1 (-0.26, 0.47)	0.06 (-0.34, 0.47)	0.15 (-0.18, 0.49)	0.07 (-0.31, 0.44)
0.2 (0.05, 0.36)	0.38 (0.23, 0.53)	0.32 (0.15, 0.5)	0.27 (0.05, 0.48)	0.2 (-0.18, 0.57)	**Tirzepatide**	0.3 (0.11, 0.49)	0.26 (0, 0.53)	0.35 (0.09, 0.61)	0.26 (-0.04, 0.57)
-0.1 (-0.21, 0.02)	0.08 (-0.1, 0.26)	0.03 (-0.09, 0.14)	-0.03 (-0.23, 0.16)	-0.1 (-0.47, 0.26)	-0.3 (-0.49, -0.11)	**Survodutide**	-0.04 (-0.29, 0.21)	0.05 (-0.19, 0.29)	-0.04 (-0.33, 0.26)
-0.06 (-0.27, 0.16)	0.12 (-0.14, 0.37)	0.06 (-0.17, 0.3)	0.01 (-0.26, 0.27)	-0.06 (-0.47, 0.34)	-0.26 (-0.53, 0)	0.04 (-0.21, 0.29)	**Visepegenatide**	0.09 (-0.21, 0.39)	0 (-0.34, 0.35)
-0.15 (-0.35, 0.06)	0.03 (-0.23, 0.27)	-0.03 (-0.26, 0.2)	-0.08 (-0.22, 0.05)	-0.15 (-0.49, 0.18)	-0.35 (-0.61, -0.09)	-0.05 (-0.29, 0.19)	-0.09 (-0.39, 0.21)	**Glimepiride**	-0.09 (-0.35, 0.17)
-0.06 (-0.32, 0.21)	0.11 (-0.19, 0.41)	0.06 (-0.22, 0.35)	0.01 (-0.21, 0.22)	-0.07 (-0.44, 0.31)	-0.26 (-0.57, 0.04)	0.04 (-0.26, 0.33)	0 (-0.35, 0.34)	0.09 (-0.17, 0.35)	**Sulfonylureas**

Table S8.20: The league table of HOMA-IR

**Placebo control groups**	-0.3 (-0.63, 0.03)	-0.11 (-0.49, 0.27)	-0.58 (-0.9, -0.25)	-0.5 (-0.83, -0.17)	-0.27 (-0.92, 0.39)	1.17 (0.61, 1.74)	-0.58 (-1.14, -0.01)
0.3 (-0.03, 0.63)	**Dulaglutide**	0.19 (-0.31, 0.69)	-0.28 (-0.74, 0.19)	-0.2 (-0.53, 0.13)	0.03 (-0.7, 0.76)	1.47 (0.82, 2.13)	-0.28 (-0.93, 0.37)
0.11 (-0.27, 0.49)	-0.19 (-0.69, 0.31)	**Semaglutide**	-0.47 (-0.97, 0.03)	-0.39 (-0.89, 0.11)	-0.16 (-0.92, 0.6)	1.28 (0.6, 1.97)	-0.47 (-1.15, 0.21)
0.58 (0.25, 0.9)	0.28 (-0.19, 0.74)	0.47 (-0.03, 0.97)	**Liraglutide**	0.08 (-0.39, 0.54)	0.31 (-0.43, 1.04)	1.75 (1.29, 2.21)	0 (-0.46, 0.46)
0.5 (0.17, 0.83)	0.2 (-0.13, 0.53)	0.39 (-0.11, 0.89)	-0.08 (-0.54, 0.39)	**Tirzepatide**	0.23 (-0.5, 0.97)	1.67 (1.02, 2.33)	-0.08 (-0.73, 0.58)
0.27 (-0.39, 0.92)	-0.03 (-0.76, 0.7)	0.16 (-0.6, 0.92)	-0.31 (-1.04, 0.43)	-0.23 (-0.97, 0.5)	**Visepegenatide**	1.44 (0.57, 2.31)	-0.31 (-1.17, 0.56)
-1.17 (-1.74, -0.61)	-1.47 (-2.13, -0.82)	-1.28 (-1.97, -0.6)	-1.75 (-2.21, -1.29)	-1.67 (-2.33, -1.02)	-1.44 (-2.31, -0.57)	**Sulfonylureas**	-1.75 (-2.4, -1.1)
0.58 (0.01, 1.14)	0.28 (-0.37, 0.93)	0.47 (-0.21, 1.15)	0 (-0.46, 0.46)	0.08 (-0.58, 0.73)	0.31 (-0.56, 1.17)	1.75 (1.1, 2.4)	**Dapagliflozin**

Table S8.21: The league table of HOMA-β

**Placebo control groups**	28.7 (18.51, 38.83)	23.85 (12.08, 35.63)	4.92 (-9.5, 19.26)	19.89 (12.18, 27.61)	19.51 (-2.16, 41.28)	31.02 (20.89, 41.26)	16.01 (1.59, 30.45)	33.84 (21.86, 45.75)	11.29 (0.44, 22.18)	2.29 (-16.81, 21.36)	14.14 (-2.12, 30.41)
-28.7 (-38.83, -18.51)	**Dulaglutide**	-4.85 (-20.43, 10.64)	-23.77 (-41.36, -6.24)	-8.81 (-21.56, 3.93)	-9.19 (-33.12, 14.89)	2.32 (-7.81, 12.56)	-12.65 (-30.37, 5.02)	5.12 (-10.5, 20.82)	-17.41 (-32.32, -2.47)	-26.38 (-47.97, -4.84)	-14.56 (-33.68, 4.67)
-23.85 (-35.63, -12.08)	4.85 (-10.64, 20.43)	**Semaglutide**	-18.92 (-37.58, -0.34)	-3.97 (-18.02, 10.15)	-4.35 (-29.01, 20.57)	7.18 (-8.37, 22.75)	-7.82 (-26.38, 10.86)	10.01 (-6.8, 26.65)	-12.56 (-28.58, 3.47)	-21.55 (-43.89, 1.01)	-9.71 (-29.78, 10.43)
-4.92 (-19.26, 9.5)	23.77 (6.24, 41.36)	18.92 (0.34, 37.58)	**Exenatide**	14.99 (-1.39, 31.25)	14.62 (-11.52, 40.62)	26.12 (8.55, 43.71)	11.13 (-9.29, 31.49)	28.94 (10.17, 47.52)	6.38 (-11.67, 24.44)	-2.62 (-21.57, 16.36)	9.21 (-12.39, 30.98)
-19.89 (-27.61, -12.18)	8.81 (-3.93, 21.56)	3.97 (-10.15, 18.02)	-14.99 (-31.25, 1.39)	**Liraglutide**	-0.38 (-20.64, 19.98)	11.14 (-1.59, 23.92)	-3.9 (-20.21, 12.46)	13.94 (4.8, 23.07)	-8.6 (-19.47, 2.32)	-17.58 (-38.16, 2.96)	-5.73 (-20.14, 8.63)
-19.51 (-41.28, 2.16)	9.19 (-14.89, 33.12)	4.35 (-20.57, 29.01)	-14.62 (-40.62, 11.52)	0.38 (-19.98, 20.64)	**Lixisenatide**	11.48 (-12.52, 35.45)	-3.49 (-29.65, 22.66)	14.32 (-7.95, 36.49)	-8.24 (-31.24, 14.71)	-17.25 (-46.15, 11.73)	-5.4 (-30.25, 19.48)
-31.02 (-41.26, -20.89)	-2.32 (-12.56, 7.81)	-7.18 (-22.75, 8.37)	-26.12 (-43.71, -8.55)	-11.14 (-23.92, 1.59)	-11.48 (-35.45, 12.52)	**Tirzepatide**	-15 (-32.78, 2.68)	2.81 (-12.92, 18.54)	-19.73 (-34.69, -4.83)	-28.71 (-50.35, -7.16)	-16.9 (-36.08, 2.31)
-16.01 (-30.45, -1.59)	12.65 (-5.02, 30.37)	7.82 (-10.86, 26.38)	-11.13 (-31.49, 9.29)	3.9 (-12.46, 20.21)	3.49 (-22.66, 29.65)	15 (-2.68, 32.78)	**Visepegenatide**	17.83 (-1.01, 36.53)	-4.74 (-22.85, 13.29)	-13.71 (-37.67, 10.23)	-1.89 (-23.65, 19.85)
-33.84 (-45.75, -21.86)	-5.12 (-20.82, 10.5)	-10.01 (-26.65, 6.8)	-28.94 (-47.52, -10.17)	-13.94 (-23.07, -4.8)	-14.32 (-36.49, 7.95)	-2.81 (-18.54, 12.92)	-17.83 (-36.53, 1.01)	**Glimepiride**	-22.55 (-36.77, -8.36)	-31.5 (-54.06, -9.04)	-19.7 (-36.71, -2.57)
-11.29 (-22.18, -0.44)	17.41 (2.47, 32.32)	12.56 (-3.47, 28.58)	-6.38 (-24.44, 11.67)	8.6 (-2.32, 19.47)	8.24 (-14.71, 31.24)	19.73 (4.83, 34.69)	4.74 (-13.29, 22.85)	22.55 (8.36, 36.77)	**Rosiglitazone**	-8.96 (-31.06, 12.93)	2.86 (-15.08, 20.82)
-2.29 (-21.36, 16.81)	26.38 (4.84, 47.97)	21.55 (-1.01, 43.89)	2.62 (-16.36, 21.57)	17.58 (-2.96, 38.16)	17.25 (-11.73, 46.15)	28.71 (7.16, 50.35)	13.71 (-10.23, 37.67)	31.5 (9.04, 54.06)	8.96 (-12.93, 31.06)	**Sitagliptin**	11.85 (-13.15, 37)
-14.14 (-30.41, 2.12)	14.56 (-4.67, 33.68)	9.71 (-10.43, 29.78)	-9.21 (-30.98, 12.39)	5.73 (-8.63, 20.14)	5.4 (-19.48, 30.25)	16.9 (-2.31, 36.08)	1.89 (-19.85, 23.65)	19.7 (2.57, 36.71)	-2.86 (-20.82, 15.08)	-11.85 (-37, 13.15)	**Sulfonylureas**

Table S8.22: The league table of Nausea

**Dulaglutide**	-2.29 (-6.73, 2.14)	3.72 (-3.72, 11.2)	-5.87 (-20.94, 9.32)	5.82 (0.49, 11.15)	-1.11 (-13.8, 11.59)	0.65 (-6.22, 7.51)	-0.45 (-6.34, 5.46)	-13.83 (-17.39, -10.27)	-11.06 (-15.59, -6.54)	2.35 (-10.19, 14.85)	-1.5 (-10.7, 7.71)	-7.39 (-17.39, 2.54)	-2.01 (-9.61, 5.55)	3.11 (-18.01, 24.32)
2.29 (-2.14, 6.73)	**Semaglutide**	6.02 (-1.52, 13.54)	-3.56 (-18.69, 11.65)	8.12 (2.71, 13.55)	1.17 (-11.53, 13.96)	2.94 (-4.03, 9.93)	1.85 (-4.75, 8.44)	-11.53 (-15.33, -7.73)	-8.75 (-13.34, -4.19)	4.65 (-7.97, 17.22)	0.8 (-8.99, 10.58)	-5.08 (-15.13, 4.94)	0.28 (-7.97, 8.56)	5.4 (-15.76, 26.64)
-3.72 (-11.2, 3.72)	-6.02 (-13.54, 1.52)	**Exenatide**	-9.57 (-25.79, 6.65)	2.1 (-5.93, 10.14)	-4.85 (-17.71, 8.03)	-3.07 (-12.11, 5.97)	-4.16 (-12.97, 4.61)	-17.56 (-24.41, -10.7)	-14.78 (-22.18, -7.36)	-1.37 (-15.19, 12.43)	-5.21 (-16.56, 6.11)	-11.11 (-22.65, 0.42)	-5.72 (-15.93, 4.39)	-0.61 (-22.67, 21.39)
5.87 (-9.32, 20.94)	3.56 (-11.65, 18.69)	9.57 (-6.65, 25.79)	**Polyethylene Glycol Loxenatide**	11.68 (-3.78, 27.08)	4.74 (-14.36, 23.85)	6.51 (-9.4, 22.41)	5.41 (-10.38, 21.06)	-7.98 (-22.68, 6.68)	-5.2 (-20.6, 10.22)	8.19 (-10.76, 27.22)	4.37 (-13.04, 21.68)	-1.53 (-18.91, 15.9)	3.84 (-12.78, 20.41)	8.96 (-16.52, 34.5)
-5.82 (-11.15, -0.49)	-8.12 (-13.55, -2.71)	-2.1 (-10.14, 5.93)	-11.68 (-27.08, 3.78)	**Liraglutide**	-6.94 (-20, 6.09)	-5.17 (-11.93, 1.6)	-6.27 (-13.4, 0.87)	-19.65 (-24.24, -15.08)	-16.88 (-22.43, -11.32)	-3.47 (-16.38, 9.3)	-7.32 (-17.46, 2.8)	-13.19 (-23.6, -2.84)	-7.83 (-16.56, 0.85)	-2.7 (-24.01, 18.67)
1.11 (-11.59, 13.8)	-1.17 (-13.96, 11.53)	4.85 (-8.03, 17.71)	-4.74 (-23.85, 14.36)	6.94 (-6.09, 20)	**Lixisenatide**	1.77 (-11.88, 15.44)	0.67 (-12.81, 14.17)	-12.7 (-24.96, -0.41)	-9.92 (-22.84, 2.98)	3.46 (-13.68, 20.61)	-0.36 (-15.68, 14.97)	-6.27 (-21.73, 9.14)	-0.89 (-15.27, 13.52)	4.22 (-19.98, 28.36)
-0.65 (-7.51, 6.22)	-2.94 (-9.93, 4.03)	3.07 (-5.97, 12.11)	-6.51 (-22.41, 9.4)	5.17 (-1.6, 11.93)	-1.77 (-15.44, 11.88)	**Efpeglenatide**	-1.09 (-9.28, 7.07)	-14.47 (-20.46, -8.49)	-11.7 (-19.06, -4.36)	1.7 (-11.7, 15.09)	-2.14 (-13.08, 8.73)	-8.03 (-19.07, 2.96)	-2.66 (-12.34, 6.96)	2.46 (-19.25, 24.2)
0.45 (-5.46, 6.34)	-1.85 (-8.44, 4.75)	4.16 (-4.61, 12.97)	-5.41 (-21.06, 10.38)	6.27 (-0.87, 13.4)	-0.67 (-14.17, 12.81)	1.09 (-7.07, 9.28)	**Tirzepatide**	-13.39 (-19.06, -7.72)	-10.62 (-17.54, -3.68)	2.79 (-10.5, 16.06)	-1.06 (-11.59, 9.52)	-6.93 (-17.81, 3.9)	-1.58 (-10.76, 7.63)	3.54 (-18.05, 25.16)
13.83 (10.27, 17.39)	11.53 (7.73, 15.33)	17.56 (10.7, 24.41)	7.98 (-6.68, 22.68)	19.65 (15.08, 24.24)	12.7 (0.41, 24.96)	14.47 (8.49, 20.46)	13.39 (7.72, 19.06)	**Placebo control groups**	2.77 (-1.84, 7.39)	16.18 (4.13, 28.19)	12.33 (3.15, 21.51)	6.45 (-2.86, 15.72)	11.82 (4.23, 19.37)	16.93 (-3.93, 37.83)
11.06 (6.54, 15.59)	8.75 (4.19, 13.34)	14.78 (7.36, 22.18)	5.2 (-10.22, 20.6)	16.88 (11.32, 22.43)	9.92 (-2.98, 22.84)	11.7 (4.36, 19.06)	10.62 (3.68, 17.54)	-2.77 (-7.39, 1.84)	**other control groups**	13.4 (0.52, 26.27)	9.56 (-0.39, 19.55)	3.68 (-6.71, 14.02)	9.04 (0.52, 17.52)	14.18 (-7.18, 35.53)
-2.35 (-14.85, 10.19)	-4.65 (-17.22, 7.97)	1.37 (-12.43, 15.19)	-8.19 (-27.22, 10.76)	3.47 (-9.3, 16.38)	-3.46 (-20.61, 13.68)	-1.7 (-15.09, 11.7)	-2.79 (-16.06, 10.5)	-16.18 (-28.19, -4.13)	-13.4 (-26.27, -0.52)	**Visepegenatide**	-3.84 (-18.94, 11.28)	-9.73 (-24.83, 5.45)	-4.37 (-18.51, 9.82)	0.78 (-23.29, 24.89)
1.5 (-7.71, 10.7)	-0.8 (-10.58, 8.99)	5.21 (-6.11, 16.56)	-4.37 (-21.68, 13.04)	7.32 (-2.8, 17.46)	0.36 (-14.97, 15.68)	2.14 (-8.73, 13.08)	1.06 (-9.52, 11.59)	-12.33 (-21.51, -3.15)	-9.56 (-19.55, 0.39)	3.84 (-11.28, 18.94)	**Retatrutide**	-5.87 (-18.96, 7.19)	-0.53 (-12.21, 11.12)	4.62 (-18.23, 27.45)
7.39 (-2.54, 17.39)	5.08 (-4.94, 15.13)	11.11 (-0.42, 22.65)	1.53 (-15.9, 18.91)	13.19 (2.84, 23.6)	6.27 (-9.14, 21.73)	8.03 (-2.96, 19.07)	6.93 (-3.9, 17.81)	-6.45 (-15.72, 2.86)	-3.68 (-14.02, 6.71)	9.73 (-5.45, 24.83)	5.87 (-7.19, 18.96)	**Orforglipron**	5.37 (-6.64, 17.39)	10.48 (-12.3, 33.37)
2.01 (-5.55, 9.61)	-0.28 (-8.56, 7.97)	5.72 (-4.39, 15.93)	-3.84 (-20.41, 12.78)	7.83 (-0.85, 16.56)	0.89 (-13.52, 15.27)	2.66 (-6.96, 12.34)	1.58 (-7.63, 10.76)	-11.82 (-19.37, -4.23)	-9.04 (-17.52, -0.52)	4.37 (-9.82, 18.51)	0.53 (-11.12, 12.21)	-5.37 (-17.39, 6.64)	**Mazdutide**	5.11 (-17.17, 27.34)
-3.11 (-24.32, 18.01)	-5.4 (-26.64, 15.76)	0.61 (-21.39, 22.67)	-8.96 (-34.5, 16.52)	2.7 (-18.67, 24.01)	-4.22 (-28.36, 19.98)	-2.46 (-24.2, 19.25)	-3.54 (-25.16, 18.05)	-16.93 (-37.83, 3.93)	-14.18 (-35.53, 7.18)	-0.78 (-24.89, 23.29)	-4.62 (-27.45, 18.23)	-10.48 (-33.37, 12.3)	-5.11 (-27.34, 17.17)	**ITCA650**

Table S8.23: The league table of Vomiting

**Dulaglutide**	-0.78 (-3.64, 2.09)	2.17 (-2.24, 6.54)	-2.11 (-11.38, 7.1)	1.75 (-1.63, 5.17)	0.2 (-7.56, 7.91)	3.4 (-0.85, 7.66)	1.54 (-2.08, 5.17)	-7.6 (-9.9, -5.32)	-5.88 (-8.69, -3.04)	1.98 (-5.71, 9.65)	-1.81 (-7.46, 3.8)	-1.18 (-7.29, 4.95)	-2.8 (-7.42, 1.84)	4.68 (-8.25, 17.59)
0.78 (-2.09, 3.64)	**Semaglutide**	2.94 (-1.58, 7.47)	-1.33 (-10.67, 8)	2.53 (-0.98, 6.05)	0.96 (-6.87, 8.77)	4.17 (-0.21, 8.57)	2.32 (-1.84, 6.48)	-6.83 (-9.44, -4.22)	-5.1 (-7.95, -2.23)	2.74 (-5.05, 10.53)	-1.03 (-7.09, 5.01)	-0.41 (-6.66, 5.85)	-2.02 (-7.14, 3.11)	5.46 (-7.48, 18.42)
-2.17 (-6.54, 2.24)	-2.94 (-7.47, 1.58)	**Exenatide**	-4.27 (-14.12, 5.51)	-0.42 (-5.03, 4.23)	-1.98 (-9.77, 5.78)	1.23 (-4.08, 6.57)	-0.63 (-5.83, 4.6)	-9.78 (-13.77, -5.78)	-8.05 (-12.43, -3.63)	-0.19 (-8.6, 8.14)	-3.98 (-10.81, 2.89)	-3.35 (-10.26, 3.59)	-4.97 (-10.98, 1.11)	2.5 (-10.81, 15.84)
2.11 (-7.1, 11.38)	1.33 (-8, 10.67)	4.27 (-5.51, 14.12)	**Polyethylene Glycol Loxenatide**	3.85 (-5.54, 13.28)	2.3 (-9.36, 13.96)	5.5 (-4.17, 15.18)	3.64 (-5.97, 13.26)	-5.5 (-14.47, 3.45)	-3.76 (-13.18, 5.69)	4.09 (-7.55, 15.65)	0.3 (-10.3, 10.91)	0.93 (-9.76, 11.55)	-0.69 (-10.75, 9.42)	6.79 (-8.72, 22.34)
-1.75 (-5.17, 1.63)	-2.53 (-6.05, 0.98)	0.42 (-4.23, 5.03)	-3.85 (-13.28, 5.54)	**Liraglutide**	-1.58 (-9.51, 6.36)	1.65 (-2.51, 5.77)	-0.21 (-4.66, 4.21)	-9.36 (-12.25, -6.49)	-7.63 (-11.18, -4.09)	0.21 (-7.69, 8.09)	-3.56 (-9.8, 2.66)	-2.93 (-9.33, 3.45)	-4.55 (-9.9, 0.82)	2.92 (-10.09, 15.96)
-0.2 (-7.91, 7.56)	-0.96 (-8.77, 6.87)	1.98 (-5.78, 9.77)	-2.3 (-13.96, 9.36)	1.58 (-6.36, 9.51)	**Lixisenatide**	3.2 (-5.02, 11.49)	1.35 (-6.84, 9.57)	-7.79 (-15.23, -0.34)	-6.06 (-13.94, 1.82)	1.78 (-8.65, 12.25)	-1.99 (-11.3, 7.32)	-1.36 (-10.71, 7.99)	-2.98 (-11.69, 5.75)	4.48 (-10.22, 19.15)
-3.4 (-7.66, 0.85)	-4.17 (-8.57, 0.21)	-1.23 (-6.57, 4.08)	-5.5 (-15.18, 4.17)	-1.65 (-5.77, 2.51)	-3.2 (-11.49, 5.02)	**Efpeglenatide**	-1.85 (-6.88, 3.16)	-11 (-14.66, -7.35)	-9.27 (-13.83, -4.72)	-1.43 (-9.62, 6.79)	-5.2 (-11.91, 1.49)	-4.57 (-11.34, 2.17)	-6.2 (-12.06, -0.33)	1.3 (-11.94, 14.49)
-1.54 (-5.17, 2.08)	-2.32 (-6.48, 1.84)	0.63 (-4.6, 5.83)	-3.64 (-13.26, 5.97)	0.21 (-4.21, 4.66)	-1.35 (-9.57, 6.84)	1.85 (-3.16, 6.88)	**Tirzepatide**	-9.15 (-12.62, -5.68)	-7.41 (-11.71, -3.13)	0.44 (-7.69, 8.52)	-3.35 (-9.78, 3.11)	-2.72 (-9.37, 3.95)	-4.33 (-9.93, 1.27)	3.13 (-10.01, 16.29)
7.6 (5.32, 9.9)	6.83 (4.22, 9.44)	9.78 (5.78, 13.77)	5.5 (-3.45, 14.47)	9.36 (6.49, 12.25)	7.79 (0.34, 15.23)	11 (7.35, 14.66)	9.15 (5.68, 12.62)	**Placebo control groups**	1.73 (-1.2, 4.67)	9.58 (2.28, 16.91)	5.79 (0.17, 11.42)	6.42 (0.73, 12.12)	4.81 (0.18, 9.45)	12.29 (-0.42, 24.96)
5.88 (3.04, 8.69)	5.1 (2.23, 7.95)	8.05 (3.63, 12.43)	3.76 (-5.69, 13.18)	7.63 (4.09, 11.18)	6.06 (-1.82, 13.94)	9.27 (4.72, 13.83)	7.41 (3.13, 11.71)	-1.73 (-4.67, 1.2)	**other control groups**	7.85 (-0.08, 15.74)	4.06 (-2.06, 10.16)	4.69 (-1.72, 11.09)	3.08 (-2.13, 8.27)	10.54 (-2.48, 23.58)
-1.98 (-9.65, 5.71)	-2.74 (-10.53, 5.05)	0.19 (-8.14, 8.6)	-4.09 (-15.65, 7.55)	-0.21 (-8.09, 7.69)	-1.78 (-12.25, 8.65)	1.43 (-6.79, 9.62)	-0.44 (-8.52, 7.69)	-9.58 (-16.91, -2.28)	-7.85 (-15.74, 0.08)	**Visepegenatide**	-3.78 (-13.02, 5.46)	-3.18 (-12.45, 6.13)	-4.78 (-13.42, 3.96)	2.71 (-11.96, 17.36)
1.81 (-3.8, 7.46)	1.03 (-5.01, 7.09)	3.98 (-2.89, 10.81)	-0.3 (-10.91, 10.3)	3.56 (-2.66, 9.8)	1.99 (-7.32, 11.3)	5.2 (-1.49, 11.91)	3.35 (-3.11, 9.78)	-5.79 (-11.42, -0.17)	-4.06 (-10.16, 2.06)	3.78 (-5.46, 13.02)	**Retatrutide**	0.62 (-7.35, 8.63)	-0.99 (-8.06, 6.14)	6.49 (-7.33, 20.38)
1.18 (-4.95, 7.29)	0.41 (-5.85, 6.66)	3.35 (-3.59, 10.26)	-0.93 (-11.55, 9.76)	2.93 (-3.45, 9.33)	1.36 (-7.99, 10.71)	4.57 (-2.17, 11.34)	2.72 (-3.95, 9.37)	-6.42 (-12.12, -0.73)	-4.69 (-11.09, 1.72)	3.18 (-6.13, 12.45)	-0.62 (-8.63, 7.35)	**Orforglipron**	-1.61 (-8.92, 5.74)	5.86 (-8.03, 19.8)
2.8 (-1.84, 7.42)	2.02 (-3.11, 7.14)	4.97 (-1.11, 10.98)	0.69 (-9.42, 10.75)	4.55 (-0.82, 9.9)	2.98 (-5.75, 11.69)	6.2 (0.33, 12.06)	4.33 (-1.27, 9.93)	-4.81 (-9.45, -0.18)	-3.08 (-8.27, 2.13)	4.78 (-3.96, 13.42)	0.99 (-6.14, 8.06)	1.61 (-5.74, 8.92)	**Mazdutide**	7.49 (-6.04, 20.93)
-4.68 (-17.59, 8.25)	-5.46 (-18.42, 7.48)	-2.5 (-15.84, 10.81)	-6.79 (-22.34, 8.72)	-2.92 (-15.96, 10.09)	-4.48 (-19.15, 10.22)	-1.3 (-14.49, 11.94)	-3.13 (-16.29, 10.01)	-12.29 (-24.96, 0.42)	-10.54 (-23.58, 2.48)	-2.71 (-17.36, 11.96)	-6.49 (-20.38, 7.33)	-5.86 (-19.8, 8.03)	-7.49 (-20.93, 6.04)	**ITCA650**

Table S8.24: The league table of Diarrhea

**Dulaglutide**	-1.12 (-3.25, 1)	-0.94 (-4.26, 2.38)	-6.94 (-14.1, 0.21)	-0.31 (-2.83, 2.2)	-3.96 (-11.28, 3.37)	-1.31 (-4.57, 1.96)	3.97 (1.17, 6.78)	-6.6 (-8.38, -4.84)	-7.34 (-9.48, -5.19)	-0.16 (-6.11, 5.82)	2.79 (-1.56, 7.15)	-3.65 (-8.41, 1.06)	20.36 (15.35, 25.34)	-2.35 (-12.34, 7.62)
1.12 (-1, 3.25)	**Semaglutide**	0.19 (-3.14, 3.51)	-5.83 (-13, 1.33)	0.82 (-1.71, 3.32)	-2.83 (-10.15, 4.47)	-0.19 (-3.48, 3.1)	5.09 (1.97, 8.2)	-5.49 (-7.29, -3.68)	-6.21 (-8.36, -4.07)	0.96 (-5.02, 6.94)	3.92 (-0.7, 8.54)	-2.53 (-7.3, 2.18)	21.49 (16.28, 26.68)	-1.23 (-11.19, 8.72)
0.94 (-2.38, 4.26)	-0.19 (-3.51, 3.14)	**Exenatide**	-6.01 (-13.58, 1.55)	0.62 (-2.81, 4.07)	-3.03 (-10.17, 4.09)	-0.37 (-4.43, 3.71)	4.9 (0.94, 8.88)	-5.67 (-8.68, -2.65)	-6.4 (-9.6, -3.18)	0.79 (-5.67, 7.19)	3.73 (-1.51, 8.97)	-2.72 (-8.06, 2.6)	21.3 (15.51, 27.07)	-1.42 (-11.71, 8.82)
6.94 (-0.21, 14.1)	5.83 (-1.33, 13)	6.01 (-1.55, 13.58)	**Polyethylene Glycol Loxenatide**	6.65 (-0.63, 13.86)	2.98 (-6.96, 12.92)	5.64 (-1.83, 13.13)	10.92 (3.44, 18.33)	0.34 (-6.59, 7.26)	-0.4 (-7.67, 6.88)	6.79 (-2.14, 15.75)	9.74 (1.57, 17.93)	3.29 (-4.91, 11.48)	27.31 (18.78, 35.81)	4.58 (-7.44, 16.64)
0.31 (-2.2, 2.83)	-0.82 (-3.32, 1.71)	-0.62 (-4.07, 2.81)	-6.65 (-13.86, 0.63)	**Liraglutide**	-3.66 (-11.01, 3.74)	-1.01 (-4.17, 2.18)	4.28 (0.93, 7.63)	-6.3 (-8.42, -4.17)	-7.03 (-9.61, -4.45)	0.14 (-5.93, 6.21)	3.1 (-1.68, 7.87)	-3.36 (-8.21, 1.52)	20.67 (15.29, 26.01)	-2.05 (-12.07, 7.97)
3.96 (-3.37, 11.28)	2.83 (-4.47, 10.15)	3.03 (-4.09, 10.17)	-2.98 (-12.92, 6.96)	3.66 (-3.74, 11.01)	**Lixisenatide**	2.66 (-5.02, 10.31)	7.93 (0.31, 15.53)	-2.64 (-9.77, 4.49)	-3.38 (-10.72, 3.96)	3.81 (-5.31, 12.94)	6.75 (-1.59, 15.09)	0.3 (-8.06, 8.71)	24.33 (15.62, 32.97)	1.61 (-10.54, 13.67)
1.31 (-1.96, 4.57)	0.19 (-3.1, 3.48)	0.37 (-3.71, 4.43)	-5.64 (-13.13, 1.83)	1.01 (-2.18, 4.17)	-2.66 (-10.31, 5.02)	**Efpeglenatide**	5.28 (1.39, 9.17)	-5.29 (-8.12, -2.48)	-6.02 (-9.49, -2.56)	1.16 (-5.22, 7.52)	4.1 (-1.05, 9.26)	-2.35 (-7.57, 2.85)	21.67 (15.94, 27.39)	-1.04 (-11.31, 9.15)
-3.97 (-6.78, -1.17)	-5.09 (-8.2, -1.97)	-4.9 (-8.88, -0.94)	-10.92 (-18.33, -3.44)	-4.28 (-7.63, -0.93)	-7.93 (-15.53, -0.31)	-5.28 (-9.17, -1.39)	**Tirzepatide**	-10.57 (-13.27, -7.89)	-11.3 (-14.57, -8.05)	-4.13 (-10.44, 2.18)	-1.18 (-6.19, 3.81)	-7.63 (-12.78, -2.49)	16.39 (10.82, 21.91)	-6.33 (-16.53, 3.85)
6.6 (4.84, 8.38)	5.49 (3.68, 7.29)	5.67 (2.65, 8.68)	-0.34 (-7.26, 6.59)	6.3 (4.17, 8.42)	2.64 (-4.49, 9.77)	5.29 (2.48, 8.12)	10.57 (7.89, 13.27)	**Placebo control groups**	-0.73 (-2.89, 1.45)	6.45 (0.74, 12.16)	9.4 (5.05, 13.75)	2.95 (-1.46, 7.34)	26.97 (21.98, 31.94)	4.26 (-5.56, 14.05)
7.34 (5.19, 9.48)	6.21 (4.07, 8.36)	6.4 (3.18, 9.6)	0.4 (-6.88, 7.67)	7.03 (4.45, 9.61)	3.38 (-3.96, 10.72)	6.02 (2.56, 9.49)	11.3 (8.05, 14.57)	0.73 (-1.45, 2.89)	**other control groups**	7.17 (1.08, 13.28)	10.13 (5.44, 14.85)	3.68 (-1.22, 8.56)	27.7 (22.4, 32.97)	4.97 (-5.04, 15.04)
0.16 (-5.82, 6.11)	-0.96 (-6.94, 5.02)	-0.79 (-7.19, 5.67)	-6.79 (-15.75, 2.14)	-0.14 (-6.21, 5.93)	-3.81 (-12.94, 5.31)	-1.16 (-7.52, 5.22)	4.13 (-2.18, 10.44)	-6.45 (-12.16, -0.74)	-7.17 (-13.28, -1.08)	**Visepegenatide**	2.96 (-4.26, 10.12)	-3.5 (-10.69, 3.72)	20.52 (12.93, 28.07)	-2.22 (-13.56, 9.15)
-2.79 (-7.15, 1.56)	-3.92 (-8.54, 0.7)	-3.73 (-8.97, 1.51)	-9.74 (-17.93, -1.57)	-3.1 (-7.87, 1.68)	-6.75 (-15.09, 1.59)	-4.1 (-9.26, 1.05)	1.18 (-3.81, 6.19)	-9.4 (-13.75, -5.05)	-10.13 (-14.85, -5.44)	-2.96 (-10.12, 4.26)	**Retatrutide**	-6.45 (-12.65, -0.25)	17.57 (11.05, 24.05)	-5.16 (-15.89, 5.59)
3.65 (-1.06, 8.41)	2.53 (-2.18, 7.3)	2.72 (-2.6, 8.06)	-3.29 (-11.48, 4.91)	3.36 (-1.52, 8.21)	-0.3 (-8.71, 8.06)	2.35 (-2.85, 7.57)	7.63 (2.49, 12.78)	-2.95 (-7.34, 1.46)	-3.68 (-8.56, 1.22)	3.5 (-3.72, 10.69)	6.45 (0.25, 12.65)	**Orforglipron**	24.02 (17.37, 30.65)	1.3 (-9.47, 12.06)
-20.36 (-25.34, -15.35)	-21.49 (-26.68, -16.28)	-21.3 (-27.07, -15.51)	-27.31 (-35.81, -18.78)	-20.67 (-26.01, -15.29)	-24.33 (-32.97, -15.62)	-21.67 (-27.39, -15.94)	-16.39 (-21.91, -10.82)	-26.97 (-31.94, -21.98)	-27.7 (-32.97, -22.4)	-20.52 (-28.07, -12.93)	-17.57 (-24.05, -11.05)	-24.02 (-30.65, -17.37)	**Mazdutide**	-22.71 (-33.71, -11.74)
2.35 (-7.62, 12.34)	1.23 (-8.72, 11.19)	1.42 (-8.82, 11.71)	-4.58 (-16.64, 7.44)	2.05 (-7.97, 12.07)	-1.61 (-13.67, 10.54)	1.04 (-9.15, 11.31)	6.33 (-3.85, 16.53)	-4.26 (-14.05, 5.56)	-4.97 (-15.04, 5.04)	2.22 (-9.15, 13.56)	5.16 (-5.59, 15.89)	-1.3 (-12.06, 9.47)	22.71 (11.74, 33.71)	**ITCA650**

Table S8.25: The league table of Constipation

**Dulaglutide**	0.46 (-1.24, 2.17)	2.13 (-1.38, 5.65)	0.33 (-1.81, 2.49)	2.89 (-1.12, 6.91)	1.94 (-0.13, 3.99)	-4.03 (-5.49, -2.57)	-3.16 (-5.51, -0.8)	4.21 (1.31, 7.09)	3.89 (0.64, 7.14)
-0.46 (-2.17, 1.24)	**Semaglutide**	1.67 (-1.87, 5.22)	-0.13 (-2.27, 2.01)	2.42 (-1.55, 6.42)	1.47 (-0.88, 3.82)	-4.49 (-5.86, -3.13)	-3.62 (-6.04, -1.22)	3.74 (0.63, 6.85)	3.43 (0.21, 6.65)
-2.13 (-5.65, 1.38)	-1.67 (-5.22, 1.87)	**Exenatide**	-1.79 (-5.38, 1.77)	0.75 (-4.32, 5.83)	-0.19 (-4.13, 3.7)	-6.17 (-9.62, -2.72)	-5.29 (-8.23, -2.36)	2.07 (-2.35, 6.49)	1.76 (-2.73, 6.27)
-0.33 (-2.49, 1.81)	0.13 (-2.01, 2.27)	1.79 (-1.77, 5.38)	**Liraglutide**	2.55 (-1.66, 6.75)	1.6 (-1.11, 4.32)	-4.37 (-6.29, -2.43)	-3.49 (-5.85, -1.11)	3.87 (0.47, 7.26)	3.56 (0.07, 7.07)
-2.89 (-6.91, 1.12)	-2.42 (-6.42, 1.55)	-0.75 (-5.83, 4.32)	-2.55 (-6.75, 1.66)	**Efpeglenatide**	-0.96 (-5.22, 3.3)	-6.92 (-10.65, -3.18)	-6.05 (-10.46, -1.64)	1.32 (-3.42, 6.05)	1 (-3.74, 5.75)
-1.94 (-3.99, 0.13)	-1.47 (-3.82, 0.88)	0.19 (-3.7, 4.13)	-1.6 (-4.32, 1.11)	0.96 (-3.3, 5.22)	**Tirzepatide**	-5.97 (-8.02, -3.91)	-5.09 (-8.05, -2.14)	2.27 (-1.13, 5.67)	1.96 (-1.6, 5.51)
4.03 (2.57, 5.49)	4.49 (3.13, 5.86)	6.17 (2.72, 9.62)	4.37 (2.43, 6.29)	6.92 (3.18, 10.65)	5.97 (3.91, 8.02)	**Placebo control groups**	0.88 (-1.47, 3.21)	8.24 (5.34, 11.13)	7.92 (5.02, 10.83)
3.16 (0.8, 5.51)	3.62 (1.22, 6.04)	5.29 (2.36, 8.23)	3.49 (1.11, 5.85)	6.05 (1.64, 10.46)	5.09 (2.14, 8.05)	-0.88 (-3.21, 1.47)	**other control groups**	7.36 (3.79, 10.94)	7.06 (3.33, 10.77)
-4.21 (-7.09, -1.31)	-3.74 (-6.85, -0.63)	-2.07 (-6.49, 2.35)	-3.87 (-7.26, -0.47)	-1.32 (-6.05, 3.42)	-2.27 (-5.67, 1.13)	-8.24 (-11.13, -5.34)	-7.36 (-10.94, -3.79)	**Retatrutide**	-0.32 (-4.42, 3.8)
-3.89 (-7.14, -0.64)	-3.43 (-6.65, -0.21)	-1.76 (-6.27, 2.73)	-3.56 (-7.07, -0.07)	-1 (-5.75, 3.74)	-1.96 (-5.51, 1.6)	-7.92 (-10.83, -5.02)	-7.06 (-10.77, -3.33)	0.32 (-3.8, 4.42)	**Orforglipron**

Suplementary 9: Funnel plots and CINeMA

The figures show the assessment of small study effect bias in studies on the effects of various GLP-1RAs on various measures of T2D. The funnel plots pertain to all trials comparing at least one GLP-1 receptor agonist versus placebo or other hypoglycemic. In the case of multi- arm trials which compared, for example, placebo vs Liraglutide vs Tirzepatide, we plotted placebo vs Liraglutide as well as placebo vs Tirzepatide. We also used Begg's test and Egger's test to assess the symmetry of the funnel plot, if $P > 0.05$, the funnel plot is proved to be symmetrical and may not have publication bias; and if $P < 0.05$, the results were tested for robustness using the "Metatrim" method.

Abbreviations: FPG, fasting plasma glucose; HDL, high density lipoprotein; LDL, low-density lipoprotein; TC, total cholesterol; TG, triglyceride; SBP, systolic blood pressure; DBP, diastolic blood pressure.

Figure S10.1: Funnel plot of HbA_{1c}

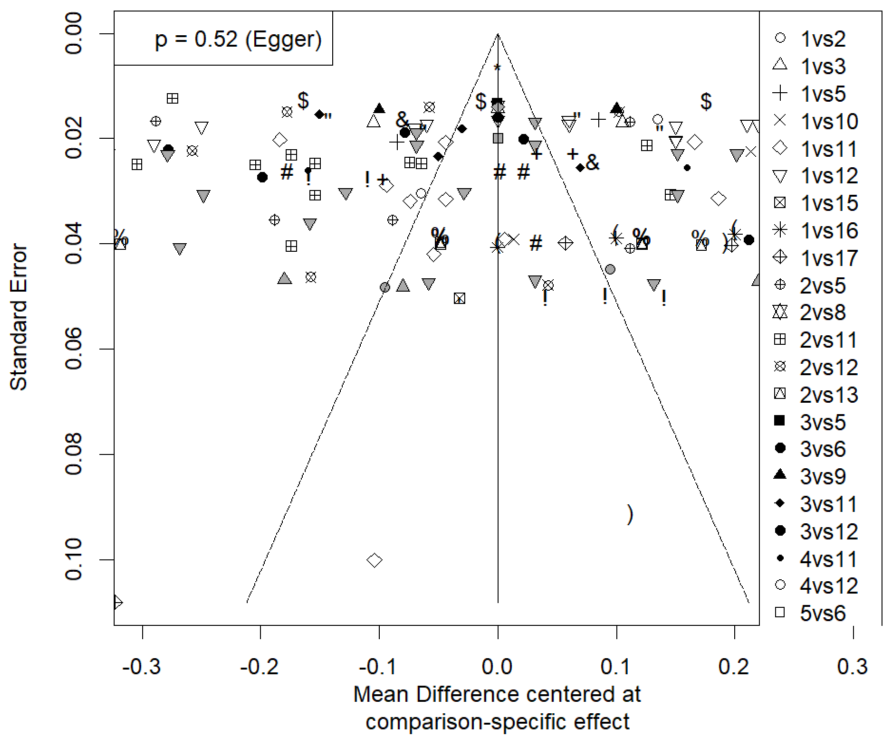

Figure S10.2: Funnel plot of FPG

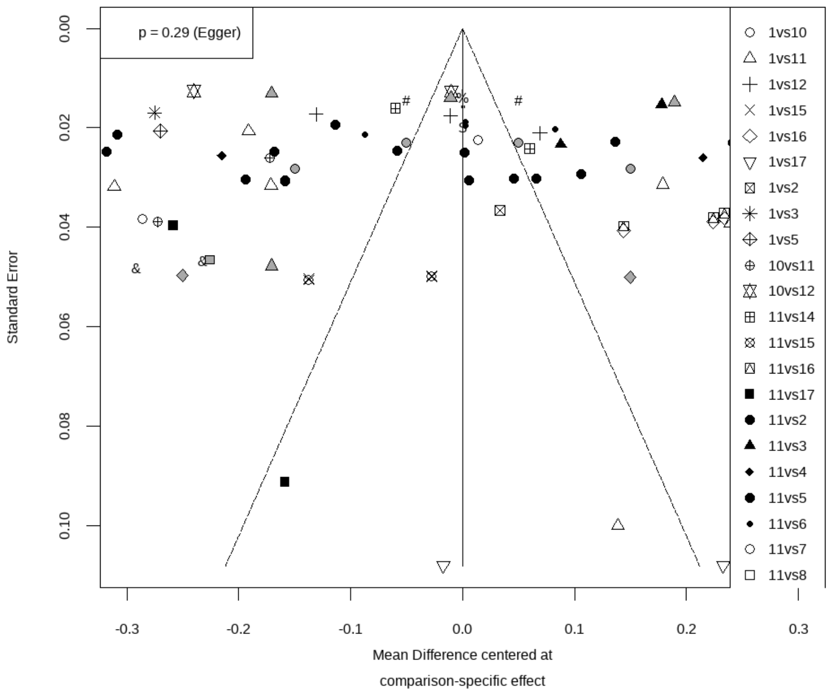

Figure S10.3: Funnel plot of weight loss

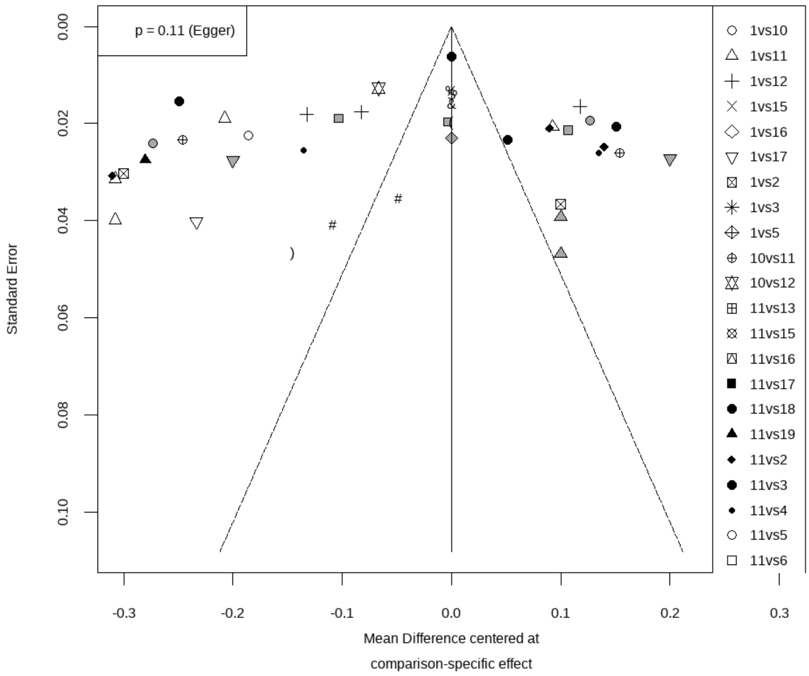

Figure S10.4: Funnel plot of HDL

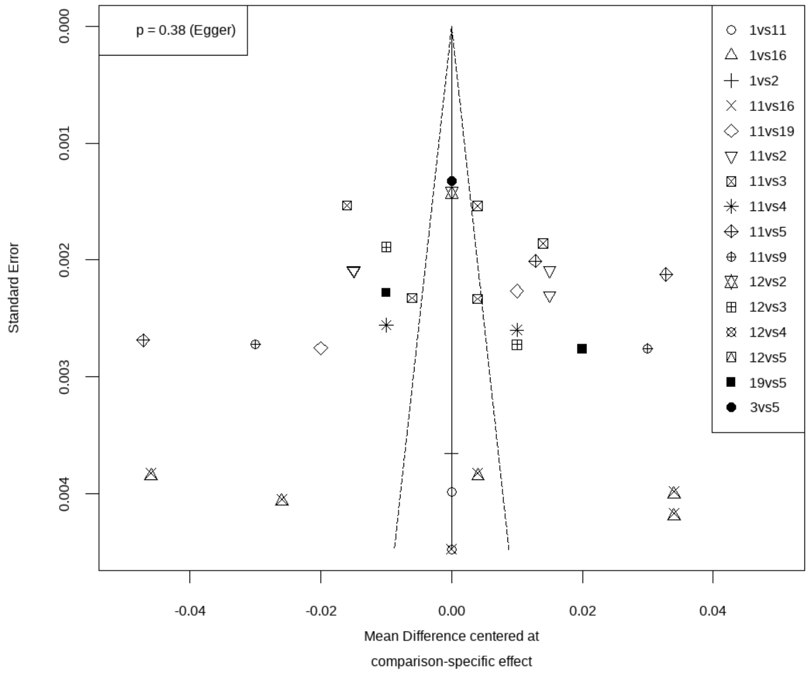

Figure S10.5: Funnel plot of LDL

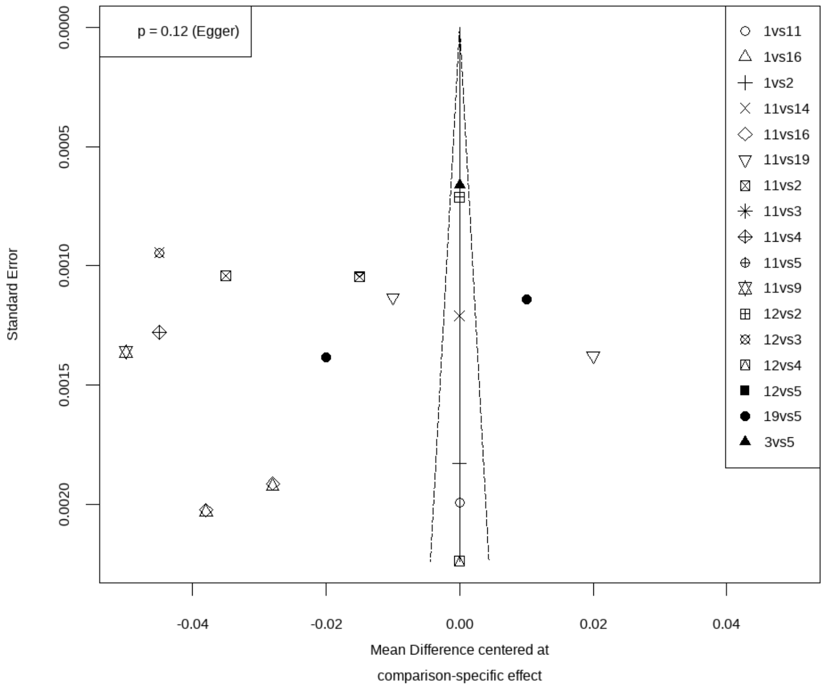

Figure S10.6: Funnel plot of TC

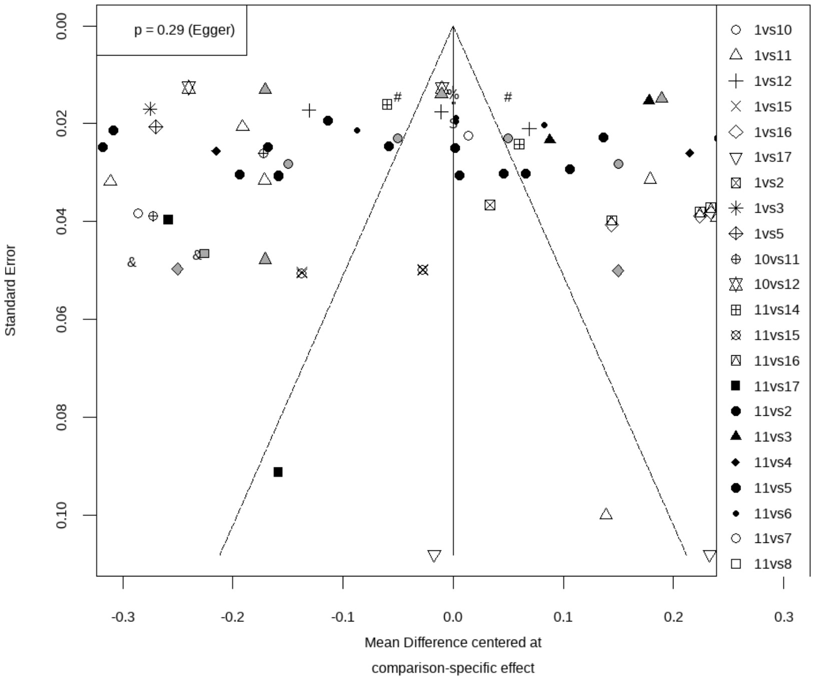

Figure S10.7: Funnel plot of TG

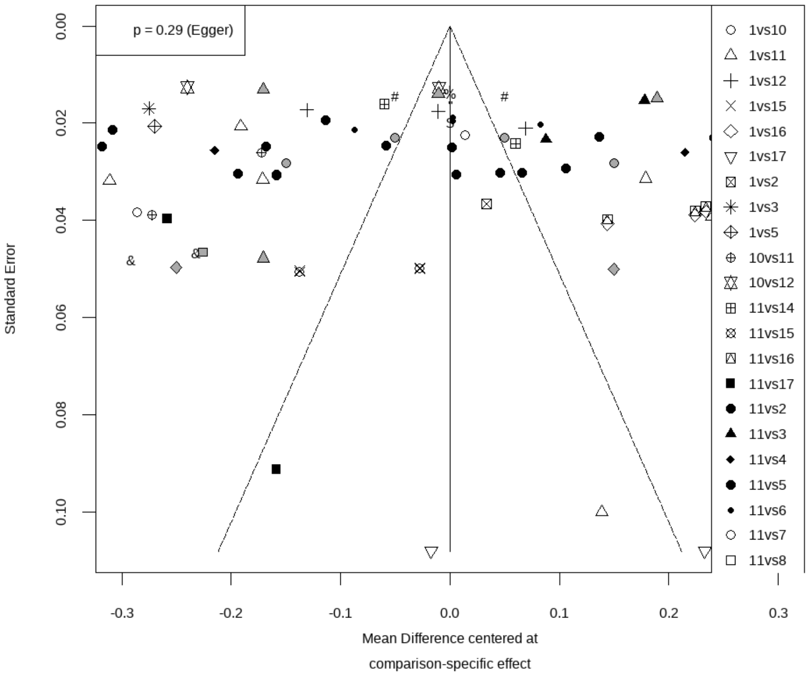

Figure S10.8: Funnel plot of SBP

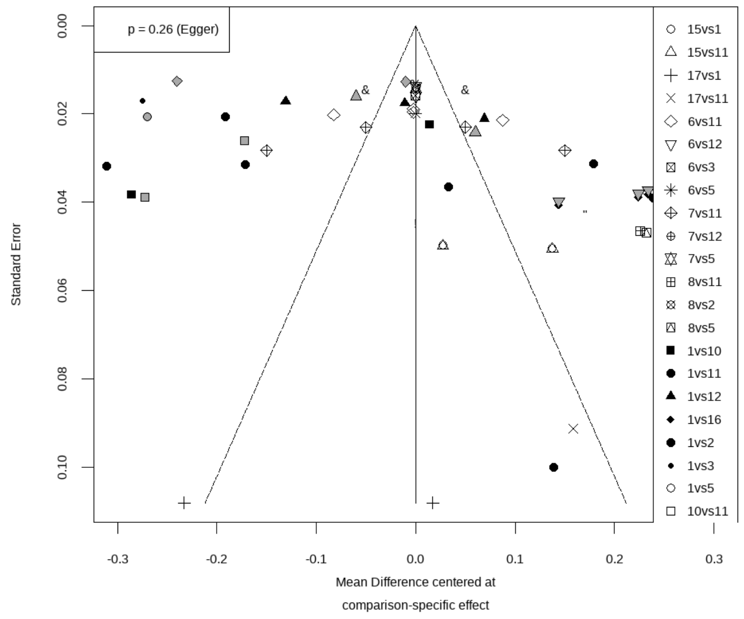

Figure S10.9: Funnel plot of DBP

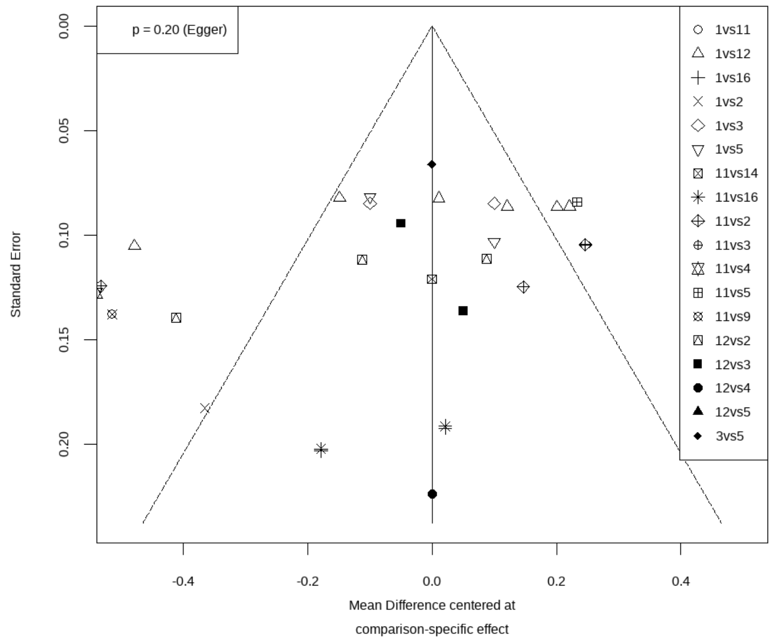

We use the CINeMA framework to evidence certainty, assessing it for each network estimate based on the following criteria:

A: Within study bias: We classified the overall risk of bias for each study as low risk of bias, the risk of bias as moderate when none of the four assessed risk of bias items were rated as high risk, and the risk of bias as high when one or both items were rated as high risk. See Appendix 4 for the bias assessment.
Reporting bias: We judged it visually by a funnel plot (Appendix 10).

Indirectness: Transferability assumptions were assessed by reporting baseline glycated hemoglobin levels in the included study population and by comparing age at baseline concordance between groups.

Table S9.1: Transitivity (Indirectness) Assessment

	Baseline variable (Mean ± SD)	
Intervention	Age(year)	HbA1c（%）
Albiglutide	54.60 ±10.81	8.20 ± 0.87
Tirzepatide	56.99 ± 10.38	8.22 ± 0.95
Liraglutide	56.53 ± 10.37	8.35 ± 1.01
Exenatide	55.73 ± 10.23	8.31 ± 0.97
Semaglutide	56.81 ±10.55	8.16 ± 0.89
Dulaglutide	56.59 ± 9.94	8.28 ± 0.99
Efpeglenatide	55.85 ± 9.51	7.80 ± 0.73
Lixisenatide	56.52 ± 9.65	8.05 ± 0.88
ITCA 650	55.10 ± 9.95	8.50 ± 0.80
Mazdutide	53.70 ± 9.34	8.73 ± 0.93
PEGylated exenatide	50.90 ± 9.62	8.66 ± 0.86
PEG-loxenatide	52.33 ± 10.62	8.47 ± 0.88
Placebo	55.88 ± 10.25	8.15 ± 0.86
CagriSema	56.00 ± 10.00	8.50 ± 0.80
Orforglipron	58.90 ± 8.86	8.09 ± 0.85
Retatrutide	56.59 ± 8.93	8.33 ± 1.09
Pooled average	56.79 ± 9.59	8.13 ± 0.93

Imprecision: We use the CINeMA website to grade the accuracy of each comparison.

Heterogeneity: We assessed the degree of worry by comparing clinical reasoning based on 95% confidence intervals (CIs) while applying the same clinical reasoning framework as for inaccuracy. In particular, we judged the consistency of our findings based on the confidence and prediction intervals associated with clinically important effect sizes. And we used the same thresholds of clinical significance as described above and followed the recommendations automatically provided by CINeMA (https://cinema.ispm.unibe.ch/).

Inconsistency: For inconsistency, we looked at the results for node splitting (Appendix 5) and we saw major problems when $p<0.10$, but otherwise no problems.

Table S10.2: CINeMA Results of HbA1c

Comparison	Within-study bias	Reporting bias	Indirectness	Imprecision	Heterogeneity	Inconsistency	Confidence rating
Dulaglutide:Liraglutide	No concerns	Low risk	No concerns	Some concerns	No concerns	No concerns	Moderate
Dulaglutide:Mazdutide	No concerns	Low risk	No concerns	No concerns	No concerns	No concerns	High
Dulaglutide:Orforglipron	No concerns	Low risk	No concerns	No concerns	Major concerns	No concerns	Low
Dulaglutide:Placebo	No concerns	Low risk	No concerns	No concerns	No concerns	No concerns	High
Dulaglutide:Retatrutide	No concerns	Low risk	No concerns	Major concerns	No concerns	No concerns	Low
Dulaglutide:Semaglutide	No concerns	Low risk	No concerns	No concerns	Major concerns	No concerns	Low
Dulaglutide:Tirzepatide	No concerns	Low risk	No concerns	No concerns	No concerns	No concerns	High
Efpeglenatide:Liraglutide	No concerns	Low risk	No concerns	Major concerns	No concerns	No concerns	Low
Efpeglenatide:Placebo	Some concerns	Low risk	No concerns	No concerns	No concerns	No concerns	High
Exenatide:Liraglutide	Some concerns	Low risk	No concerns	Some concerns	No concerns	No concerns	High
Exenatide:Placebo	Some concerns	Low risk	No concerns	No concerns	No concerns	No concerns	Moderate
Exenatide:Semaglutide	Some concerns	Low risk	No concerns	No concerns	Major concerns	No concerns	Low
ITCA 650:Placebo	No concerns	Low risk	No concerns	No concerns	No concerns	No concerns	High
Liraglutide:Placebo	No concerns	Low risk	No concerns	No concerns	No concerns	No concerns	High
Liraglutide:Semaglutide	No concerns	Low risk	No concerns	No concerns	Major concerns	No concerns	Low

Lixisenatide:Placebo	Some concerns	Low risk	No concerns	No concerns	No concerns	No concerns	Moderate
Mazdutide:Placebo	No concerns	Low risk	No concerns	No concerns	No concerns	No concerns	High
Orforglipron:Placebo	No concerns	Low risk	No concerns	No concerns	No concerns	No concerns	High
PEG-Loxenatide:Placebo	Some concerns	Low risk	No concerns	No concerns	No concerns	No concerns	Moderate
Placebo:Retatrutide	Some concerns	Low risk	No concerns	No concerns	No concerns	No concerns	Moderate
Placebo:Semaglutide	No concerns	Low risk	No concerns	No concerns	No concerns	No concerns	High
Placebo:Tirzepatide	No concerns	Low risk	No concerns	No concerns	No concerns	No concerns	High
Semaglutide:Tirzepatide	No concerns	Low risk	No concerns	No concerns	Major concerns	No concerns	Low
Dulaglutide:Efpeglenatide	No concerns	Low risk	No concerns	Major concerns	No concerns	No concerns	Low
Dulaglutide:Exenatide	Some concerns	Low risk	No concerns	Major concerns	No concerns	No concerns	Low
Dulaglutide:ITCA 650	No concerns	Low risk	No concerns	Major concerns	No concerns	No concerns	Low
Dulaglutide:Lixisenatide	Some concerns	Low risk	No concerns	No concerns	Major concerns	No concerns	Low
Dulaglutide:PEG-Loxenatide	Some concerns	Low risk	No concerns	Major concerns	No concerns	No concerns	Low
Efpeglenatide:Exenatide	Some concerns	Low risk	No concerns	Major concerns	No concerns	No concerns	Low
Efpeglenatide:ITCA 650	No concerns	Low risk	No concerns	Major concerns	No concerns	No concerns	Low

Efpeglenatide:Lixisenatide	Some concerns	Low risk	No concerns	Major concerns	No concerns	No concerns	Low
Efpeglenatide:Mazdutide	No concerns	Low risk	No concerns	No concerns	No concerns	No concerns	High
Efpeglenatide:Orforglipron	No concerns	Low risk	No concerns	No concerns	No concerns	No concerns	High
Efpeglenatide:PEG-Loxenatide	Some concerns	Low risk	No concerns	Major concerns	No concerns	No concerns	Low
Efpeglenatide:Retatrutide	Some concerns	Low risk	No concerns	Major concerns	No concerns	No concerns	Low
Efpeglenatide:Semaglutide	No concerns	Low risk	No concerns	No concerns	Major concerns	No concerns	Low
Efpeglenatide:Tirzepatide	No concerns	Low risk	No concerns	No concerns	No concerns	No concerns	High
Exenatide:ITCA 650	No concerns	Low risk	No concerns	Some concerns	No concerns	No concerns	Moderate
Exenatide:Lixisenatide	Some concerns	Low risk	No concerns	Major concerns	No concerns	No concerns	Low
Exenatide:Mazdutide	No concerns	Low risk	No concerns	No concerns	No concerns	No concerns	High
Exenatide:Orforglipron	Some concerns	Low risk	No concerns	No concerns	No concerns	No concerns	Moderate
Exenatide:PEG-Loxenatide	Some concerns	Low risk	No concerns	Major concerns	No concerns	No concerns	Low
Exenatide:Retatrutide	Some concerns	Low risk	No concerns	Major concerns	No concerns	No concerns	Low
Exenatide:Tirzepatide	No concerns	Low risk	No concerns	No concerns	No concerns	No concerns	High
ITCA 650:Liraglutide	No concerns	Low risk	No concerns	Major concerns	No concerns	No concerns	Low
ITCA 650:Lixisenatide	No concerns	Low risk	No concerns	Major concerns	No concerns	No concerns	Low

ITCA 650:Mazdutide	No concerns	Low risk	No concerns	No concerns	No concerns	No concerns	High
ITCA 650:Orforglipron	No concerns	Low risk	No concerns	Major concerns	No concerns	No concerns	Low
ITCA 650:PEG-Loxenatide	Some concerns	Low risk	No concerns	Major concerns	No concerns	No concerns	Low
ITCA 650:Retatrutide	No concerns	Low risk	No concerns	Major concerns	No concerns	No concerns	Low
ITCA 650:Semaglutide	No concerns	Low risk	No concerns	Major concerns	No concerns	No concerns	Low
ITCA 650:Tirzepatide	No concerns	Low risk	No concerns	No concerns	No concerns	No concerns	High
Liraglutide:Lixisenatide	Some concerns	Low risk	No concerns	No concerns	Major concerns	No concerns	Low
Liraglutide:Mazdutide	No concerns	Low risk	No concerns	No concerns	No concerns	No concerns	High
Liraglutide:Orforglipron	No concerns	Low risk	No concerns	No concerns	Major concerns	No concerns	Low
Liraglutide:PEG-Loxenatide	Some concerns	Low risk	No concerns	Major concerns	No concerns	No concerns	Low
Liraglutide:Retatrutide	No concerns	Low risk	No concerns	Major concerns	No concerns	No concerns	Low
Liraglutide:Tirzepatide	No concerns	Low risk	No concerns	No concerns	No concerns	No concerns	High
Lixisenatide:Mazdutide	No concerns	Low risk	No concerns	No concerns	No concerns	No concerns	High
Lixisenatide:Orforglipron	No concerns	Low risk	No concerns	No concerns	No concerns	No concerns	High
Lixisenatide:PEG-Loxenatide	Some concerns	Low risk	No concerns	Major concerns	No concerns	No concerns	Low
Lixisenatide:Retatrutide	Some concerns	Low risk	No concerns	No concerns	Major concerns	No concerns	Low

Lixisenatide:Semaglutide	Some concerns	Low risk	No concerns	No concerns	No concerns	No concerns	Moderate
Lixisenatide:Tirzepatide	No concerns	Low risk	No concerns	No concerns	No concerns	No concerns	High
Mazdutide:Orforglipron	No concerns	Low risk	No concerns	Major concerns	No concerns	No concerns	Low
Mazdutide:PEG-Loxenatide	Some concerns	Low risk	No concerns	No concerns	No concerns	No concerns	Moderate
Mazdutide:Retatrutide	No concerns	Low risk	No concerns	Major concerns	No concerns	No concerns	Low
Mazdutide:Semaglutide	No concerns	Low risk	No concerns	No concerns	Major concerns	No concerns	Low
Mazdutide:Tirzepatide	No concerns	Low risk	No concerns	Major concerns	No concerns	No concerns	Low
Orforglipron:PEG-Loxenatide	Some concerns	Low risk	No concerns	Major concerns	No concerns	No concerns	Low
Orforglipron:Retatrutide	No concerns	Low risk	No concerns	Major concerns	No concerns	No concerns	Low
Orforglipron:Semaglutide	No concerns	Low risk	No concerns	Major concerns	No concerns	No concerns	Low
Orforglipron:Tirzepatide	No concerns	Low risk	No concerns	Major concerns	No concerns	No concerns	Low
PEG-Loxenatide:Retatrutide	Some concerns	Low risk	No concerns	Major concerns	No concerns	No concerns	Low
PEG-Loxenatide:Semaglutide	Some concerns	Low risk	No concerns	Major concerns	No concerns	No concerns	Low

PEG-Loxenatide:Tirzepatide	Some concerns	Low risk	No concerns	No concerns	No concerns	No concerns	High
Retatrutide:Semaglutide	No concerns	Low risk	No concerns	Major concerns	No concerns	No concerns	Low
Retatrutide:Tirzepatide	No concerns	Low risk	No concerns	No concerns	Major concerns	No concerns	Low
Visepegenatid : Placebo	No concerns	Low risk	No concerns	No concerns	No concerns	No concerns	High
Visepegenatid : Cotadutide	No concerns	Low risk	No concerns	Major concerns	No concerns	No concerns	Low
Survodutide: Placebo	No· concerns	Low risk	No concerns	No concerns	No concerns	No concerns	High
Cotadutide : Liraglutide	No concerns	Low risk	No concerns	No concerns	No concerns	No concerns	Low
Cotadutide : Placebo	No concerns	Low risk	No concerns	No concerns	No concerns	No concerns	High
Cotadutide : Dulaglutide	No concerns	Low risk	No concerns	Major concerns	No concerns	No concerns	Low
Cotadutide : Orforglipron	No concerns	Low risk	No concerns	Major concerns	No concerns	No concerns	Low
Cotadutide : Mazdutide	No concerns	Low risk	No concerns	Major concerns	No concerns	No concerns	Low
Cotadutide : Tirzepatide	No concerns	Low risk	No concerns	Major concerns	No concerns	No concerns	Low
Cotadutide : Retatrutide	No concerns	Low risk	No concerns	Major concerns	No concerns	No concerns	Low
Cotadutide : Semaglutide	No concerns	Low risk	No concerns	Major concerns	No concerns	No concerns	Low
Cotadutide :PEG-Loxenatide	No concerns	Low risk	No concerns	Major concerns	No concerns	No concerns	Low

Cotadutide :Lixisenatide	No concerns	Low risk	No concerns	Major concerns	No concerns	No concerns	Low
Cotadutide :Exenatide	No concerns	Low risk	No concerns	Major concerns	No concerns	No concerns	Low
Cotadutide :Efpeglenatide	No concerns	Low risk	No concerns	Major concerns	No concerns	No concerns	Low
Cotadutide :Survodutide	No concerns	Low risk	No concerns	Major concerns	No concerns	No concerns	Low
Cotadutide :ITCA650	No concerns	Low risk	No concerns	Major concerns	No concerns	No concerns	Low

Table S10.3: CINeMA Results of FPG

Comparison	Within-study bias	Reporting bias	Indirectness	Imprecision	Heterogeneity	Inconsistency	Confidence rating
Dulaglutide:Liraglutide	No concerns	Low risk	No concerns	Some concerns	No concerns	No concerns	Moderate
Dulaglutide:Mazdutide	No concerns	Low risk	No concerns	No concerns	No concerns	No concerns	High
Dulaglutide:Orforglipron	No concerns	Low risk	No concerns	No concerns	Major concerns	No concerns	Low
Dulaglutide:Placebo	No concerns	Low risk	No concerns	No concerns	No concerns	No concerns	High
Dulaglutide:Retatrutide	No concerns	Low risk	No concerns	Major concerns	No concerns	No concerns	Low
Dulaglutide:Semaglutide	No concerns	Low risk	No concerns	No concerns	Major concerns	No concerns	Low
Dulaglutide:Tirzepatide	No concerns	Low risk	No concerns	No concerns	No concerns	No concerns	High

Efpeglenatide:Liraglutide	No concerns	Low risk	No concerns	Major concerns	No concerns	No concerns	Low
Efpeglenatide:Placebo	Some concerns	Low risk	No concerns	No concerns	No concerns	No concerns	High
Exenatide:Liraglutide	Some concerns	Low risk	No concerns	Some concerns	No concerns	No concerns	High
Exenatide:Placebo	Some concerns	Low risk	No concerns	No concerns	No concerns	No concerns	Moderate
Exenatide:Semaglutide	Some concerns	Low risk	No concerns	No concerns	Major concerns	No concerns	Low
Liraglutide:Placebo	No concerns	Low risk	No concerns	No concerns	No concerns	No concerns	High
Liraglutide:Semaglutide	No concerns	Low risk	No concerns	No concerns	Major concerns	No concerns	Low
Lixisenatide:Placebo	Some concerns	Low risk	No concerns	No concerns	No concerns	No concerns	Moderate
Mazdutide:Placebo	No concerns	Low risk	No concerns	No concerns	No concerns	No concerns	High
Orforglipron:Placebo	No concerns	Low risk	No concerns	No concerns	No concerns	No concerns	High
PEG-Loxenatide:Placebo	Some concerns	Low risk	No concerns	No concerns	No concerns	No concerns	Moderate
Placebo:Retatrutide	Some concerns	Low risk	No concerns	No concerns	No concerns	No concerns	Moderate
Placebo:Semaglutide	No concerns	Low risk	No concerns	No concerns	No concerns	No concerns	High
Placebo:Tirzepatide	No concerns	Low risk	No concerns	No concerns	No concerns	No concerns	High
Semaglutide:Tirzepatide	No concerns	Low risk	No concerns	No concerns	Major concerns	No concerns	Low
Dulaglutide:Efpeglenatide	No concerns	Low risk	No concerns	Major concerns	No concerns	No concerns	Low

Dulaglutide:Exenatide	Some concerns	Low risk	No concerns	Major concerns	No concerns	No concerns	Low
Dulaglutide:Lixisenatide	Some concerns	Low risk	No concerns	No concerns	Major concerns	No concerns	Low
Dulaglutide:PEG-Loxenatide	Some concerns	Low risk	No concerns	Major concerns	No concerns	No concerns	Low
Efpeglenatide:Exenatide	Some concerns	Low risk	No concerns	Major concerns	No concerns	No concerns	Low
Efpeglenatide:Lixisenatide	Some concerns	Low risk	No concerns	Major concerns	No concerns	No concerns	Low
Efpeglenatide:Mazdutide	No concerns	Low risk	No concerns	No concerns	No concerns	No concerns	High
Efpeglenatide:Orforglipron	No concerns	Low risk	No concerns	No concerns	No concerns	No concerns	High
Efpeglenatide:PEG-Loxenatide	Some concerns	Low risk	No concerns	Major concerns	No concerns	No concerns	Low
Efpeglenatide:Retatrutide	Some concerns	Low risk	No concerns	Major concerns	No concerns	No concerns	Low
Efpeglenatide:Semaglutide	No concerns	Low risk	No concerns	No concerns	Major concerns	No concerns	Low
Efpeglenatide:Tirzepatide	No concerns	Low risk	No concerns	No concerns	No concerns	No concerns	High
Exenatide:Lixisenatide	Some concerns	Low risk	No concerns	Major concerns	No concerns	No concerns	Low
Exenatide:Mazdutide	No concerns	Low risk	No concerns	No concerns	No concerns	No concerns	High
Exenatide:Orforglipron	Some concerns	Low risk	No concerns	No concerns	No concerns	No concerns	Moderate
Exenatide:PEG-Loxenatide	Some concerns	Low risk	No concerns	Major concerns	No concerns	No concerns	Low

Exenatide:Retatrutide	Some concerns	Low risk	No concerns	Major concerns	No concerns	No concerns	Low
Exenatide:Tirzepatide	No concerns	Low risk	No concerns	No concerns	No concerns	No concerns	High
Liraglutide:Lixisenatide	Some concerns	Low risk	No concerns	No concerns	Major concerns	No concerns	Low
Liraglutide:Mazdutide	No concerns	Low risk	No concerns	No concerns	No concerns	No concerns	High
Liraglutide:Orforglipron	No concerns	Low risk	No concerns	No concerns	Major concerns	No concerns	Low
Liraglutide:PEG-Loxenatide	Some concerns	Low risk	No concerns	Major concerns	No concerns	No concerns	Low
Liraglutide:Retatrutide	No concerns	Low risk	No concerns	Major concerns	No concerns	No concerns	Low
Liraglutide:Tirzepatide	No concerns	Low risk	No concerns	No concerns	No concerns	No concerns	High
Lixisenatide:Mazdutide	No concerns	Low risk	No concerns	No concerns	No concerns	No concerns	High
Lixisenatide:Orforglipron	No concerns	Low risk	No concerns	No concerns	No concerns	No concerns	High
Lixisenatide:PEG-Loxenatide	Some concerns	Low risk	No concerns	Major concerns	No concerns	No concerns	Low
Lixisenatide:Retatrutide	Some concerns	Low risk	No concerns	No concerns	Major concerns	No concerns	Low
Lixisenatide:Semaglutide	Some concerns	Low risk	No concerns	No concerns	No concerns	No concerns	Moderate
Lixisenatide:Tirzepatide	No concerns	Low risk	No concerns	No concerns	No concerns	No concerns	High
Mazdutide:Orforglipron	No concerns	Low risk	No concerns	Major concerns	No concerns	No concerns	Low
Mazdutide:PEG-Loxenatide	Some concerns	Low risk	No concerns	No concerns	No concerns	No concerns	Moderate

Mazdutide:Retatrutide	No concerns	Low risk	No concerns	Major concerns	No concerns	No concerns	Low
Mazdutide:Semaglutide	No concerns	Low risk	No concerns	No concerns	Major concerns	No concerns	Low
Mazdutide:Tirzepatide	No concerns	Low risk	No concerns	Major concerns	No concerns	No concerns	Low
Orforglipron:PEG-Loxenatide	Some concerns	Low risk	No concerns	Major concerns	No concerns	No concerns	Low
Orforglipron:Retatrutide	No concerns	Low risk	No concerns	Major concerns	No concerns	No concerns	Low
Orforglipron:Semaglutide	No concerns	Low risk	No concerns	Major concerns	No concerns	No concerns	Low
Orforglipron:Tirzepatide	No concerns	Low risk	No concerns	Major concerns	No concerns	No concerns	Low
PEG-Loxenatide:Retatrutide	Some concerns	Low risk	No concerns	Major concerns	No concerns	No concerns	Low
PEG-Loxenatide:Semaglutide	Some concerns	Low risk	No concerns	Major concerns	No concerns	No concerns	Low
PEG-Loxenatide:Tirzepatide	Some concerns	Low risk	No concerns	No concerns	No concerns	No concerns	High
Retatrutide:Semaglutide	No concerns	Low risk	No concerns	Major concerns	No concerns	No concerns	Low
Retatrutide:Tirzepatide	No concerns	Low risk	No concerns	No concerns	Major concerns	No concerns	Low
Visepegenatid : Placebo	No concerns	Low risk	No concerns	No concerns	No concerns	No concerns	High

Table S10.4: CINeMA Results of Weight

Comparison	Within-study bias	Reporting bias	Indirectness	Imprecision	Heterogeneity	Inconsistency	Confidence rating
Dulaglutide:Liraglutide	No concerns	Low risk	No concerns	Some concerns	No concerns	No concerns	Moderate
Dulaglutide:Mazdutide	No concerns	Low risk	No concerns	No concerns	No concerns	No concerns	High
Dulaglutide:Orforglipron	No concerns	Low risk	No concerns	No concerns	Major concerns	No concerns	Low
Dulaglutide:Placebo	No concerns	Low risk	No concerns	No concerns	No concerns	No concerns	High
Dulaglutide:Retatrutide	No concerns	Low risk	No concerns	Major concerns	No concerns	No concerns	Low
Dulaglutide:Semaglutide	No concerns	Low risk	No concerns	No concerns	Major concerns	No concerns	Low
Dulaglutide:Tirzepatide	No concerns	Low risk	No concerns	No concerns	No concerns	No concerns	High
Efpeglenatide:Liraglutide	No concerns	Low risk	No concerns	Major concerns	No concerns	No concerns	Low
Efpeglenatide:Placebo	Some concerns	Low risk	No concerns	No concerns	No concerns	No concerns	High
Exenatide:Liraglutide	Some concerns	Low risk	No concerns	Some concerns	No concerns	No concerns	High
Exenatide:Placebo	Some concerns	Low risk	No concerns	No concerns	No concerns	No concerns	Moderate
Exenatide:Semaglutide	Some concerns	Low risk	No concerns	No concerns	Major concerns	No concerns	Low
ITCA 650:Placebo	No concerns	Low risk	No concerns	No concerns	No concerns	No concerns	High
Liraglutide:Placebo	No concerns	Low risk	No concerns	No concerns	No concerns	No concerns	High
Liraglutide:Semaglutide	No concerns	Low risk	No concerns	No concerns	Major concerns	No concerns	Low

Lixisenatide:Placebo	Some concerns	Low risk	No concerns	No concerns	No concerns	No concerns	Moderate
Mazdutide:Placebo	No concerns	Low risk	No concerns	No concerns	No concerns	No concerns	High
Orforglipron:Placebo	No concerns	Low risk	No concerns	No concerns	No concerns	No concerns	High
PEG-Loxenatide:Placebo	Some concerns	Low risk	No concerns	No concerns	No concerns	No concerns	Moderate
Placebo:Retatrutide	Some concerns	Low risk	No concerns	No concerns	No concerns	No concerns	Moderate
Placebo:Semaglutide	No concerns	Low risk	No concerns	No concerns	No concerns	No concerns	High
Placebo:Tirzepatide	No concerns	Low risk	No concerns	No concerns	No concerns	No concerns	High
Semaglutide:Tirzepatide	No concerns	Low risk	No concerns	No concerns	Major concerns	No concerns	Low
Dulaglutide:Efpeglenatide	No concerns	Low risk	No concerns	Major concerns	No concerns	No concerns	Low
Dulaglutide:Exenatide	Some concerns	Low risk	No concerns	Major concerns	No concerns	No concerns	Low
Dulaglutide:ITCA 650	No concerns	Low risk	No concerns	Major concerns	No concerns	No concerns	Low
Dulaglutide:Lixisenatide	Some concerns	Low risk	No concerns	No concerns	Major concerns	No concerns	Low
Dulaglutide:PEG-Loxenatide	Some concerns	Low risk	No concerns	Major concerns	No concerns	No concerns	Low
Efpeglenatide:Exenatide	Some concerns	Low risk	No concerns	Major concerns	No concerns	No concerns	Low
Efpeglenatide:ITCA 650	No concerns	Low risk	No concerns	Major concerns	No concerns	No concerns	Low

Efpeglenatide:Lixisenatide	Some concerns	Low risk	No concerns	Major concerns	No concerns	No concerns	Low
Efpeglenatide:Mazdutide	No concerns	Low risk	No concerns	No concerns	No concerns	No concerns	High
Efpeglenatide:Orforglipron	No concerns	Low risk	No concerns	No concerns	No concerns	No concerns	High
Efpeglenatide:PEG-Loxenatide	Some concerns	Low risk	No concerns	Major concerns	No concerns	No concerns	Low
Efpeglenatide:Retatrutide	Some concerns	Low risk	No concerns	Major concerns	No concerns	No concerns	Low
Efpeglenatide:Semaglutide	No concerns	Low risk	No concerns	No concerns	Major concerns	No concerns	Low
Efpeglenatide:Tirzepatide	No concerns	Low risk	No concerns	No concerns	No concerns	No concerns	High
Exenatide:ITCA 650	No concerns	Low risk	No concerns	Some concerns	No concerns	No concerns	Moderate
Exenatide:Lixisenatide	Some concerns	Low risk	No concerns	Major concerns	No concerns	No concerns	Low
Exenatide:Mazdutide	No concerns	Low risk	No concerns	No concerns	No concerns	No concerns	High
Exenatide:Orforglipron	Some concerns	Low risk	No concerns	No concerns	No concerns	No concerns	Moderate
Exenatide:PEG-Loxenatide	Some concerns	Low risk	No concerns	Major concerns	No concerns	No concerns	Low
Exenatide:Retatrutide	Some concerns	Low risk	No concerns	Major concerns	No concerns	No concerns	Low
Exenatide:Tirzepatide	No concerns	Low risk	No concerns	No concerns	No concerns	No concerns	High
ITCA 650:Liraglutide	No concerns	Low risk	No concerns	Major concerns	No concerns	No concerns	Low
ITCA 650:Lixisenatide	No concerns	Low risk	No concerns	Major concerns	No concerns	No concerns	Low

ITCA 650:Mazdutide	No concerns	Low risk	No concerns	No concerns	No concerns	No concerns	High
ITCA 650:Orforglipron	No concerns	Low risk	No concerns	Major concerns	No concerns	No concerns	Low
ITCA 650:PEG-Loxenatide	Some concerns	Low risk	No concerns	Major concerns	No concerns	No concerns	Low
ITCA 650:Retatrutide	No concerns	Low risk	No concerns	Major concerns	No concerns	No concerns	Low
ITCA 650:Semaglutide	No concerns	Low risk	No concerns	Major concerns	No concerns	No concerns	Low
ITCA 650:Tirzepatide	No concerns	Low risk	No concerns	No concerns	No concerns	No concerns	High
Liraglutide:Lixisenatide	Some concerns	Low risk	No concerns	No concerns	Major concerns	No concerns	Low
Liraglutide:Mazdutide	No concerns	Low risk	No concerns	No concerns	No concerns	No concerns	High
Liraglutide:Orforglipron	No concerns	Low risk	No concerns	No concerns	Major concerns	No concerns	Low
Liraglutide:PEG-Loxenatide	Some concerns	Low risk	No concerns	Major concerns	No concerns	No concerns	Low
Liraglutide:Retatrutide	No concerns	Low risk	No concerns	Major concerns	No concerns	No concerns	Low
Liraglutide:Tirzepatide	No concerns	Low risk	No concerns	No concerns	No concerns	No concerns	High
Lixisenatide:Mazdutide	No concerns	Low risk	No concerns	No concerns	No concerns	No concerns	High
Lixisenatide:Orforglipron	No concerns	Low risk	No concerns	No concerns	No concerns	No concerns	High
Lixisenatide:PEG-Loxenatide	Some concerns	Low risk	No concerns	Major concerns	No concerns	No concerns	Low
Lixisenatide:Retatrutide	Some concerns	Low risk	No concerns	No concerns	Major concerns	No concerns	Low

Lixisenatide:Semaglutide	Some concerns	Low risk	No concerns	No concerns	No concerns	No concerns	Moderate
Lixisenatide:Tirzepatide	No concerns	Low risk	No concerns	No concerns	No concerns	No concerns	High
Mazdutide:Orforglipron	No concerns	Low risk	No concerns	Major concerns	No concerns	No concerns	Low
Mazdutide:PEG-Loxenatide	Some concerns	Low risk	No concerns	No concerns	No concerns	No concerns	Moderate
Mazdutide:Retatrutide	No concerns	Low risk	No concerns	Major concerns	No concerns	No concerns	Low
Mazdutide:Semaglutide	No concerns	Low risk	No concerns	No concerns	Major concerns	No concerns	Low
Mazdutide:Tirzepatide	No concerns	Low risk	No concerns	Major concerns	No concerns	No concerns	Low
Orforglipron:PEG-Loxenatide	Some concerns	Low risk	No concerns	Major concerns	No concerns	No concerns	Low
Orforglipron:Retatrutide	No concerns	Low risk	No concerns	Major concerns	No concerns	No concerns	Low
Orforglipron:Semaglutide	No concerns	Low risk	No concerns	Major concerns	No concerns	No concerns	Low
Orforglipron:Tirzepatide	No concerns	Low risk	No concerns	Major concerns	No concerns	No concerns	Low
PEG-Loxenatide:Retatrutide	Some concerns	Low risk	No concerns	Major concerns	No concerns	No concerns	Low
PEG-Loxenatide:Semaglutide	Some concerns	Low risk	No concerns	Major concerns	No concerns	No concerns	Low

PEG-Loxenatide:Tirzepatide	Some concerns	Low risk	No concerns	No concerns	No concerns	No concerns	High
Retatrutide:Semaglutide	No concerns	Low risk	No concerns	Major concerns	No concerns	No concerns	Low
Retatrutide:Tirzepatide	No concerns	Low risk	No concerns	No concerns	Major concerns	No concerns	Low
Survodutide: Placebo	No· concerns	Low risk	No concerns	No concerns	No concerns	No concerns	High
Cotadutide : Liraglutide	No concerns	Low risk	No concerns	No concerns	No concerns	No concerns	Low
Cotadutide : Placebo	No concerns	Low risk	No concerns	No concerns	No concerns	No concerns	High
Cotadutide : Dulaglutide	No concerns	Low risk	No concerns	Major concerns	No concerns	No concerns	Low
Cotadutide : Orforglipron	No concerns	Low risk	No concerns	Major concerns	No concerns	No concerns	Low
Cotadutide : Mazdutide	No concerns	Low risk	No concerns	Major concerns	No concerns	No concerns	Low
Cotadutide : Tirzepatide	No concerns	Low risk	No concerns	Major concerns	No concerns	No concerns	Low
Cotadutide : Retatrutide	No concerns	Low risk	No concerns	Major concerns	No concerns	No concerns	Low
Cotadutide : Semaglutide	No concerns	Low risk	No concerns	Major concerns	No concerns	No concerns	Low
Cotadutide :PEG-Loxenatide	No concerns	Low risk	No concerns	Major concerns	No concerns	No concerns	Low
Cotadutide :Lixisenatide	No concerns	Low risk	No concerns	Major concerns	No concerns	No concerns	Low

Cotadutide :Exenatide	No concerns	Low risk	No concerns	Major concerns	No concerns	No concerns	Low
Cotadutide :Efpeglenatide	No concerns	Low risk	No concerns	Major concerns	No concerns	No concerns	Low
Cotadutide :Survodutide	No concerns	Low risk	No concerns	Major concerns	No concerns	No concerns	Low
Cotadutide :ITCA650	No concerns	Low risk	No concerns	Major concerns	No concerns	No concerns	Low

Table S10.5: CINeMA Results of the proportion of patients whose HbA1c reaches the target of 7.0%

Comparison	Within-study bias	Reporting bias	Indirectness	Imprecision	Heterogeneity	Inconsistency	Confidence rating
Dulaglutide:Liraglutide	No concerns	Low risk	No concerns	Some concerns	No concerns	No concerns	Moderate
Dulaglutide:Mazdutide	No concerns	Low risk	No concerns	No concerns	No concerns	No concerns	High
Dulaglutide:Orforglipron	No concerns	Low risk	No concerns	No concerns	Major concerns	No concerns	Low
Dulaglutide:Placebo	No concerns	Low risk	No concerns	No concerns	No concerns	No concerns	High
Dulaglutide:Retatrutide	No concerns	Low risk	No concerns	Major concerns	No concerns	No concerns	Low
Dulaglutide:Semaglutide	No concerns	Low risk	No concerns	No concerns	Major concerns	No concerns	Low
Dulaglutide:Tirzepatide	No concerns	Low risk	No concerns	No concerns	No concerns	No concerns	High
Efpeglenatide:Liraglutide	No concerns	Low risk	No concerns	Major concerns	No concerns	No concerns	Low
Efpeglenatide:Placebo	Some concerns	Low risk	No concerns	No concerns	No concerns	No concerns	High

Exenatide:Liraglutide	Some concerns	Low risk	No concerns	Some concerns	No concerns	No concerns	High
Exenatide:Placebo	Some concerns	Low risk	No concerns	No concerns	No concerns	No concerns	Moderate
Exenatide:Semaglutide	Some concerns	Low risk	No concerns	No concerns	Major concerns	No concerns	Low
ITCA 650:Placebo	No concerns	Low risk	No concerns	No concerns	No concerns	No concerns	High
Liraglutide:Placebo	No concerns	Low risk	No concerns	No concerns	No concerns	No concerns	High
Liraglutide:Semaglutide	No concerns	Low risk	No concerns	No concerns	Major concerns	No concerns	Low
Lixisenatide:Placebo	Some concerns	Low risk	No concerns	No concerns	No concerns	No concerns	Moderate
Mazdutide:Placebo	No concerns	Low risk	No concerns	No concerns	No concerns	No concerns	High
Orforglipron:Placebo	No concerns	Low risk	No concerns	No concerns	No concerns	No concerns	High
PEG-Loxenatide:Placebo	Some concerns	Low risk	No concerns	No concerns	No concerns	No concerns	Moderate
Placebo:Retatrutide	Some concerns	Low risk	No concerns	No concerns	No concerns	No concerns	Moderate
Placebo:Semaglutide	No concerns	Low risk	No concerns	No concerns	No concerns	No concerns	High
Placebo:Tirzepatide	No concerns	Low risk	No concerns	No concerns	No concerns	No concerns	High
Semaglutide:Tirzepatide	No concerns	Low risk	No concerns	No concerns	Major concerns	No concerns	Low
Dulaglutide:Efpeglenatide	No concerns	Low risk	No concerns	Major concerns	No concerns	No concerns	Low
Dulaglutide:Exenatide	Some concerns	Low risk	No concerns	Major concerns	No concerns	No concerns	Low

Dulaglutide:ITCA 650	No concerns	Low risk	No concerns	Major concerns	No concerns	No concerns	Low
Dulaglutide:Lixisenatide	Some concerns	Low risk	No concerns	No concerns	Major concerns	No concerns	Low
Dulaglutide:PEG-Loxenatide	Some concerns	Low risk	No concerns	Major concerns	No concerns	No concerns	Low
Efpeglenatide:Exenatide	Some concerns	Low risk	No concerns	Major concerns	No concerns	No concerns	Low
Efpeglenatide:ITCA 650	No concerns	Low risk	No concerns	Major concerns	No concerns	No concerns	Low
Efpeglenatide:Lixisenatide	Some concerns	Low risk	No concerns	Major concerns	No concerns	No concerns	Low
Efpeglenatide:Mazdutide	No concerns	Low risk	No concerns	No concerns	No concerns	No concerns	High
Efpeglenatide:Orforglipron	No concerns	Low risk	No concerns	No concerns	No concerns	No concerns	High
Efpeglenatide:PEG-Loxenatide	Some concerns	Low risk	No concerns	Major concerns	No concerns	No concerns	Low
Efpeglenatide:Retatrutide	Some concerns	Low risk	No concerns	Major concerns	No concerns	No concerns	Low
Efpeglenatide:Semaglutide	No concerns	Low risk	No concerns	No concerns	Major concerns	No concerns	Low
Efpeglenatide:Tirzepatide	No concerns	Low risk	No concerns	No concerns	No concerns	No concerns	High
Exenatide:ITCA 650	No concerns	Low risk	No concerns	Some concerns	No concerns	No concerns	Moderate
Exenatide:Lixisenatide	Some concerns	Low risk	No concerns	Major concerns	No concerns	No concerns	Low
Exenatide:Mazdutide	No concerns	Low risk	No concerns	No concerns	No concerns	No concerns	High

Exenatide:Orforglipron	Some concerns	Low risk	No concerns	No concerns	No concerns	No concerns	Moderate
Exenatide:PEG-Loxenatide	Some concerns	Low risk	No concerns	Major concerns	No concerns	No concerns	Low
Exenatide:Retatrutide	Some concerns	Low risk	No concerns	Major concerns	No concerns	No concerns	Low
Exenatide:Tirzepatide	No concerns	Low risk	No concerns	No concerns	No concerns	No concerns	High
ITCA 650:Liraglutide	No concerns	Low risk	No concerns	Major concerns	No concerns	No concerns	Low
ITCA 650:Lixisenatide	No concerns	Low risk	No concerns	Major concerns	No concerns	No concerns	Low
ITCA 650:Mazdutide	No concerns	Low risk	No concerns	No concerns	No concerns	No concerns	High
ITCA 650:Orforglipron	No concerns	Low risk	No concerns	Major concerns	No concerns	No concerns	Low
ITCA 650:PEG-Loxenatide	Some concerns	Low risk	No concerns	Major concerns	No concerns	No concerns	Low
ITCA 650:Retatrutide	No concerns	Low risk	No concerns	Major concerns	No concerns	No concerns	Low
ITCA 650:Semaglutide	No concerns	Low risk	No concerns	Major concerns	No concerns	No concerns	Low
ITCA 650:Tirzepatide	No concerns	Low risk	No concerns	No concerns	No concerns	No concerns	High
Liraglutide:Lixisenatide	Some concerns	Low risk	No concerns	No concerns	Major concerns	No concerns	Low
Liraglutide:Mazdutide	No concerns	Low risk	No concerns	No concerns	No concerns	No concerns	High
Liraglutide:Orforglipron	No concerns	Low risk	No concerns	No concerns	Major concerns	No concerns	Low

Liraglutide:PEG-Loxenatide	Some concerns	Low risk	No concerns	Major concerns	No concerns	No concerns	Low
Liraglutide:Retatrutide	No concerns	Low risk	No concerns	Major concerns	No concerns	No concerns	Low
Liraglutide:Tirzepatide	No concerns	Low risk	No concerns	No concerns	No concerns	No concerns	High
Lixisenatide:Mazdutide	No concerns	Low risk	No concerns	No concerns	No concerns	No concerns	High
Lixisenatide:Orforglipron	No concerns	Low risk	No concerns	No concerns	No concerns	No concerns	High
Lixisenatide:PEG-Loxenatide	Some concerns	Low risk	No concerns	Major concerns	No concerns	No concerns	Low
Lixisenatide:Retatrutide	Some concerns	Low risk	No concerns	No concerns	Major concerns	No concerns	Low
Lixisenatide:Semaglutide	Some concerns	Low risk	No concerns	No concerns	No concerns	No concerns	Moderate
Lixisenatide:Tirzepatide	No concerns	Low risk	No concerns	No concerns	No concerns	No concerns	High
Mazdutide:Orforglipron	No concerns	Low risk	No concerns	Major concerns	No concerns	No concerns	Low
Mazdutide:PEG-Loxenatide	Some concerns	Low risk	No concerns	No concerns	No concerns	No concerns	Moderate
Mazdutide:Retatrutide	No concerns	Low risk	No concerns	Major concerns	No concerns	No concerns	Low
Mazdutide:Semaglutide	No concerns	Low risk	No concerns	No concerns	Major concerns	No concerns	Low
Mazdutide:Tirzepatide	No concerns	Low risk	No concerns	Major concerns	No concerns	No concerns	Low
Orforglipron:PEG-Loxenatide	Some concerns	Low risk	No concerns	Major concerns	No concerns	No concerns	Low

Orforglipron:Retatrutide	No concerns	Low risk	No concerns	Major concerns	No concerns	No concerns	Low
Orforglipron:Semaglutide	No concerns	Low risk	No concerns	Major concerns	No concerns	No concerns	Low
Orforglipron:Tirzepatide	No concerns	Low risk	No concerns	Major concerns	No concerns	No concerns	Low
PEG-Loxenatide:Retatrutide	Some concerns	Low risk	No concerns	Major concerns	No concerns	No concerns	Low
PEG-Loxenatide:Semaglutide	Some concerns	Low risk	No concerns	Major concerns	No concerns	No concerns	Low
PEG-Loxenatide:Tirzepatide	Some concerns	Low risk	No concerns	No concerns	No concerns	No concerns	High
Retatrutide:Semaglutide	No concerns	Low risk	No concerns	Major concerns	No concerns	No concerns	Low
Retatrutide:Tirzepatide	No concerns	Low risk	No concerns	No concerns	Major concerns	No concerns	Low
Visepegenatid : Placebo	No concerns	Low risk	No concerns	No concerns	No concerns	No concerns	High
Visepegenatid : Cotadutide	No concerns	Low risk	No concerns	Major concerns	No concerns	No concerns	Low
Survodutide: Placebo	No· concerns	Low risk	No concerns	No concerns	No concerns	No concerns	High
Cotadutide : Liraglutide	No concerns	Low risk	No concerns	No concerns	No concerns	No concerns	Low
Cotadutide : Placebo	No concerns	Low risk	No concerns	No concerns	No concerns	No concerns	High
Cotadutide : Dulaglutide	No concerns	Low risk	No concerns	Major concerns	No concerns	No concerns	Low
Cotadutide : Orforglipron	No concerns	Low risk	No concerns	Major concerns	No concerns	No concerns	Low

Cotadutide : Mazdutide	No concerns	Low risk	No concerns	Major concerns	No concerns	No concerns	Low
Cotadutide : Tirzepatide	No concerns	Low risk	No concerns	Major concerns	No concerns	No concerns	Low
Cotadutide : Retatrutide	No concerns	Low risk	No concerns	Major concerns	No concerns	No concerns	Low
Cotadutide : Semaglutide	No concerns	Low risk	No concerns	Major concerns	No concerns	No concerns	Low
Cotadutide :PEG-Loxenatide	No concerns	Low risk	No concerns	Major concerns	No concerns	No concerns	Low
Cotadutide :Lixisenatide	No concerns	Low risk	No concerns	Major concerns	No concerns	No concerns	Low
Cotadutide :Exenatide	No concerns	Low risk	No concerns	Major concerns	No concerns	No concerns	Low
Cotadutide :Efpeglenatide	No concerns	Low risk	No concerns	Major concerns	No concerns	No concerns	Low
Cotadutide :Survodutide	No concerns	Low risk	No concerns	Major concerns	No concerns	No concerns	Low
Cotadutide :ITCA650	No concerns	Low risk	No concerns	Major concerns	No concerns	No concerns	Low

Table S10.6: CINeMA Results of the proportion of patients whose HbA1c reaches the target of 6.5%

Comparison	Within-study bias	Reporting bias	Indirectness	Imprecision	Heterogeneity	Inconsistency	Confidence rating
Dulaglutide:Liraglutide	No concerns	Low risk	No concerns	Some concerns	No concerns	No concerns	Moderate
Dulaglutide:Mazdutide	No concerns	Low risk	No concerns	No concerns	No concerns	No concerns	High
Dulaglutide:Orforglipron	No concerns	Low risk	No concerns	No concerns	Major concerns	No concerns	Low
Dulaglutide:Placebo	No concerns	Low risk	No concerns	No concerns	No concerns	No concerns	High
Dulaglutide:Retatrutide	No concerns	Low risk	No concerns	Major concerns	No concerns	No concerns	Low
Dulaglutide:Semaglutide	No concerns	Low risk	No concerns	No concerns	Major concerns	No concerns	Low
Dulaglutide:Tirzepatide	No concerns	Low risk	No concerns	No concerns	No concerns	No concerns	High
Efpeglenatide:Liraglutide	No concerns	Low risk	No concerns	Major concerns	No concerns	No concerns	Low
Efpeglenatide:Placebo	Some concerns	Low risk	No concerns	No concerns	No concerns	No concerns	High
Exenatide:Liraglutide	Some concerns	Low risk	No concerns	Some concerns	No concerns	No concerns	High
Exenatide:Placebo	Some concerns	Low risk	No concerns	No concerns	No concerns	No concerns	Moderate
Exenatide:Semaglutide	Some concerns	Low risk	No concerns	No concerns	Major concerns	No concerns	Low
ITCA 650:Placebo	No concerns	Low risk	No concerns	No concerns	No concerns	No concerns	High
Liraglutide:Placebo	No concerns	Low risk	No concerns	No concerns	No concerns	No concerns	High
Liraglutide:Semaglutide	No concerns	Low risk	No concerns	No concerns	Major concerns	No concerns	Low
Lixisenatide:Placebo	Some concerns	Low risk	No concerns	No concerns	No concerns	No concerns	Moderate

Mazdutide:Placebo	No concerns	Low risk	No concerns	No concerns	No concerns	No concerns	High
Orforglipron:Placebo	No concerns	Low risk	No concerns	No concerns	No concerns	No concerns	High
PEG-Loxenatide:Placebo	Some concerns	Low risk	No concerns	No concerns	No concerns	No concerns	Moderate
Placebo:Retatrutide	Some concerns	Low risk	No concerns	No concerns	No concerns	No concerns	Moderate
Placebo:Semaglutide	No concerns	Low risk	No concerns	No concerns	No concerns	No concerns	High
Placebo:Tirzepatide	No concerns	Low risk	No concerns	No concerns	No concerns	No concerns	High
Semaglutide:Tirzepatide	No concerns	Low risk	No concerns	No concerns	Major concerns	No concerns	Low
Dulaglutide:Efpeglenatide	No concerns	Low risk	No concerns	Major concerns	No concerns	No concerns	Low
Dulaglutide:Exenatide	Some concerns	Low risk	No concerns	Major concerns	No concerns	No concerns	Low
Dulaglutide:ITCA 650	No concerns	Low risk	No concerns	Major concerns	No concerns	No concerns	Low
Dulaglutide:Lixisenatide	Some concerns	Low risk	No concerns	No concerns	Major concerns	No concerns	Low
Dulaglutide:PEG-Loxenatide	Some concerns	Low risk	No concerns	Major concerns	No concerns	No concerns	Low
Efpeglenatide:Exenatide	Some concerns	Low risk	No concerns	Major concerns	No concerns	No concerns	Low
Efpeglenatide:ITCA 650	No concerns	Low risk	No concerns	Major concerns	No concerns	No concerns	Low
Efpeglenatide:Lixisenatide	Some concerns	Low risk	No concerns	Major concerns	No concerns	No concerns	Low

Efpeglenatide:Mazdutide	No concerns	Low risk	No concerns	No concerns	No concerns	No concerns	High
Efpeglenatide:Orforglipron	No concerns	Low risk	No concerns	No concerns	No concerns	No concerns	High
Efpeglenatide:PEG-Loxenatide	Some concerns	Low risk	No concerns	Major concerns	No concerns	No concerns	Low
Efpeglenatide:Retatrutide	Some concerns	Low risk	No concerns	Major concerns	No concerns	No concerns	Low
Efpeglenatide:Semaglutide	No concerns	Low risk	No concerns	No concerns	Major concerns	No concerns	Low
Efpeglenatide:Tirzepatide	No concerns	Low risk	No concerns	No concerns	No concerns	No concerns	High
Exenatide:ITCA 650	No concerns	Low risk	No concerns	Some concerns	No concerns	No concerns	Moderate
Exenatide:Lixisenatide	Some concerns	Low risk	No concerns	Major concerns	No concerns	No concerns	Low
Exenatide:Mazdutide	No concerns	Low risk	No concerns	No concerns	No concerns	No concerns	High
Exenatide:Orforglipron	Some concerns	Low risk	No concerns	No concerns	No concerns	No concerns	Moderate
Exenatide:PEG-Loxenatide	Some concerns	Low risk	No concerns	Major concerns	No concerns	No concerns	Low
Exenatide:Retatrutide	Some concerns	Low risk	No concerns	Major concerns	No concerns	No concerns	Low
Exenatide:Tirzepatide	No concerns	Low risk	No concerns	No concerns	No concerns	No concerns	High
ITCA 650:Liraglutide	No concerns	Low risk	No concerns	Major concerns	No concerns	No concerns	Low
ITCA 650:Lixisenatide	No concerns	Low risk	No concerns	Major concerns	No concerns	No concerns	Low
ITCA 650:Mazdutide	No concerns	Low risk	No concerns	No concerns	No concerns	No concerns	High

ITCA 650:Orforglipron	No concerns	Low risk	No concerns	Major concerns	No concerns	No concerns	Low
ITCA 650:PEG-Loxenatide	Some concerns	Low risk	No concerns	Major concerns	No concerns	No concerns	Low
ITCA 650:Retatrutide	No concerns	Low risk	No concerns	Major concerns	No concerns	No concerns	Low
ITCA 650:Semaglutide	No concerns	Low risk	No concerns	Major concerns	No concerns	No concerns	Low
ITCA 650:Tirzepatide	No concerns	Low risk	No concerns	No concerns	No concerns	No concerns	High
Liraglutide:Lixisenatide	Some concerns	Low risk	No concerns	No concerns	Major concerns	No concerns	Low
Liraglutide:Mazdutide	No concerns	Low risk	No concerns	No concerns	No concerns	No concerns	High
Liraglutide:Orforglipron	No concerns	Low risk	No concerns	No concerns	Major concerns	No concerns	Low
Liraglutide:PEG-Loxenatide	Some concerns	Low risk	No concerns	Major concerns	No concerns	No concerns	Low
Liraglutide:Retatrutide	No concerns	Low risk	No concerns	Major concerns	No concerns	No concerns	Low
Liraglutide:Tirzepatide	No concerns	Low risk	No concerns	No concerns	No concerns	No concerns	High
Lixisenatide:Mazdutide	No concerns	Low risk	No concerns	No concerns	No concerns	No concerns	High
Lixisenatide:Orforglipron	No concerns	Low risk	No concerns	No concerns	No concerns	No concerns	High
Lixisenatide:PEG-Loxenatide	Some concerns	Low risk	No concerns	Major concerns	No concerns	No concerns	Low
Lixisenatide:Retatrutide	Some concerns	Low risk	No concerns	No concerns	Major concerns	No concerns	Low

Lixisenatide:Semaglutide	Some concerns	Low risk	No concerns	No concerns	No concerns	No concerns	Moderate
Lixisenatide:Tirzepatide	No concerns	Low risk	No concerns	No concerns	No concerns	No concerns	High
Mazdutide:Orforglipron	No concerns	Low risk	No concerns	Major concerns	No concerns	No concerns	Low
Mazdutide:PEG-Loxenatide	Some concerns	Low risk	No concerns	No concerns	No concerns	No concerns	Moderate
Mazdutide:Retatrutide	No concerns	Low risk	No concerns	Major concerns	No concerns	No concerns	Low
Mazdutide:Semaglutide	No concerns	Low risk	No concerns	No concerns	Major concerns	No concerns	Low
Mazdutide:Tirzepatide	No concerns	Low risk	No concerns	Major concerns	No concerns	No concerns	Low
Orforglipron:PEG-Loxenatide	Some concerns	Low risk	No concerns	Major concerns	No concerns	No concerns	Low
Orforglipron:Retatrutide	No concerns	Low risk	No concerns	Major concerns	No concerns	No concerns	Low
Orforglipron:Semaglutide	No concerns	Low risk	No concerns	Major concerns	No concerns	No concerns	Low
Orforglipron:Tirzepatide	No concerns	Low risk	No concerns	Major concerns	No concerns	No concerns	Low
PEG-Loxenatide:Retatrutide	Some concerns	Low risk	No concerns	Major concerns	No concerns	No concerns	Low
PEG-Loxenatide:Semaglutide	Some concerns	Low risk	No concerns	Major concerns	No concerns	No concerns	Low

PEG-Loxenatide:Tirzepatide	Some concerns	Low risk	No concerns	No concerns	No concerns	No concerns	High
Retatrutide:Semaglutide	No concerns	Low risk	No concerns	Major concerns	No concerns	No concerns	Low
Retatrutide:Tirzepatide	No concerns	Low risk	No concerns	No concerns	Major concerns	No concerns	Low
Visepegenatid : Placebo	No concerns	Low risk	No concerns	No concerns	No concerns	No concerns	High
Visepegenatid : Cotadutide	No concerns	Low risk	No concerns	Major concerns	No concerns	No concerns	Low
Survodutide: Placebo	No· concerns	Low risk	No concerns	No concerns	No concerns	No concerns	High
Cotadutide : Liraglutide	No concerns	Low risk	No concerns	No concerns	No concerns	No concerns	Low
Cotadutide : Placebo	No concerns	Low risk	No concerns	No concerns	No concerns	No concerns	High
Cotadutide : Dulaglutide	No concerns	Low risk	No concerns	Major concerns	No concerns	No concerns	Low
Cotadutide : Orforglipron	No concerns	Low risk	No concerns	Major concerns	No concerns	No concerns	Low
Cotadutide : Mazdutide	No concerns	Low risk	No concerns	Major concerns	No concerns	No concerns	Low
Cotadutide : Tirzepatide	No concerns	Low risk	No concerns	Major concerns	No concerns	No concerns	Low
Cotadutide : Retatrutide	No concerns	Low risk	No concerns	Major concerns	No concerns	No concerns	Low
Cotadutide : Semaglutide	No concerns	Low risk	No concerns	Major concerns	No concerns	No concerns	Low
Cotadutide :PEG-Loxenatide	No concerns	Low risk	No concerns	Major concerns	No concerns	No concerns	Low

Cotadutide :Lixisenatide	No concerns	Low risk	No concerns	Major concerns	No concerns	No concerns	Low
Cotadutide :Exenatide	No concerns	Low risk	No concerns	Major concerns	No concerns	No concerns	Low
Cotadutide :Efpeglenatide	No concerns	Low risk	No concerns	Major concerns	No concerns	No concerns	Low
Cotadutide :Survodutide	No concerns	Low risk	No concerns	Major concerns	No concerns	No concerns	Low
Cotadutide :ITCA650	No concerns	Low risk	No concerns	Major concerns	No concerns	No concerns	Low

Table S10.7: CINeMA Results of Blood lipid.

Comparison	Within-study bias	Reporting bias	Indirectness	Imprecision	Heterogeneity	Inconsistency	Confidence rating
Dulaglutide:Liraglutide	No concerns	Low risk	No concerns	Some concerns	No concerns	No concerns	Moderate
Dulaglutide:Orforglipron	No concerns	Low risk	No concerns	No concerns	Major concerns	No concerns	Low
Dulaglutide:Placebo	No concerns	Low risk	No concerns	No concerns	No concerns	No concerns	High
Dulaglutide:Semaglutide	No concerns	Low risk	No concerns	No concerns	Major concerns	No concerns	Low
Exenatide:Liraglutide	Some concerns	Low risk	No concerns	Some concerns	No concerns	No concerns	High
Exenatide:Placebo	Some concerns	Low risk	No concerns	No concerns	No concerns	No concerns	Moderate

Exenatide:Semaglutide	Some concerns	Low risk	No concerns	No concerns	Major concerns	No concerns	Low
Liraglutide:Placebo	No concerns	Low risk	No concerns	No concerns	No concerns	No concerns	High
Liraglutide:Semaglutide	No concerns	Low risk	No concerns	No concerns	Major concerns	No concerns	Low
Orforglipron:Placebo	No concerns	Low risk	No concerns	No concerns	No concerns	No concerns	High
PEG-Loxenatide:Placebo	Some concerns	Low risk	No concerns	No concerns	No concerns	No concerns	Moderate
Placebo:Semaglutide	No concerns	Low risk	No concerns	No concerns	No concerns	No concerns	High
Dulaglutide:Exenatide	Some concerns	Low risk	No concerns	Major concerns	No concerns	No concerns	Low
Dulaglutide:PEG-Loxenatide	Some concerns	Low risk	No concerns	Major concerns	No concerns	No concerns	Low
Exenatide:Orforglipron	Some concerns	Low risk	No concerns	No concerns	No concerns	No concerns	Moderate
Exenatide:PEG-Loxenatide	Some concerns	Low risk	No concerns	Major concerns	No concerns	No concerns	Low
Liraglutide:Orforglipron	No concerns	Low risk	No concerns	No concerns	Major concerns	No concerns	Low
Liraglutide:PEG-Loxenatide	Some concerns	Low risk	No concerns	Major concerns	No concerns	No concerns	Low
Orforglipron:PEG-Loxenatide	Some concerns	Low risk	No concerns	Major concerns	No concerns	No concerns	Low
Orforglipron:Semaglutide	No concerns	Low risk	No concerns	Major concerns	No concerns	No concerns	Low
PEG-Loxenatide:Semaglutide	Some concerns	Low risk	No concerns	Major concerns	No concerns	No concerns	Low

Visepegenatid : Placebo	No concerns	Low risk	No concerns	No concerns	No concerns	No concerns	High
Cotadutide : Liraglutide	No concerns	Low risk	No concerns	No concerns	No concerns	No concerns	Low
Cotadutide : Placebo	No concerns	Low risk	No concerns	No concerns	No concerns	No concerns	High
Cotadutide : Dulaglutide	No concerns	Low risk	No concerns	Major concerns	No concerns	No concerns	Low
Cotadutide : Orforglipron	No concerns	Low risk	No concerns	Major concerns	No concerns	No concerns	Low
Cotadutide : Semaglutide	No concerns	Low risk	No concerns	Major concerns	No concerns	No concerns	Low
Cotadutide :PEG-Loxenatide	No concerns	Low risk	No concerns	Major concerns	No concerns	No concerns	Low
Cotadutide :Lixisenatide	No concerns	Low risk	No concerns	Major concerns	No concerns	No concerns	Low
Cotadutide :Exenatide	No concerns	Low risk	No concerns	Major concerns	No concerns	No concerns	Low

Table S10.8: CINeMA Results of blood pressure。

Comparison	Within-study bias	Reporting bias	Indirectness	Imprecision	Heterogeneity	Inconsistency	Confidence rating
Dulaglutide:Liraglutide	No concerns	Low risk	No concerns	Some concerns	No concerns	No concerns	Moderate
Dulaglutide:Orforglipron	No concerns	Low risk	No concerns	No concerns	Major concerns	No concerns	Low
Dulaglutide:Placebo	No concerns	Low risk	No concerns	No concerns	No concerns	No concerns	High
Dulaglutide:Semaglutide	No concerns	Low risk	No concerns	No concerns	Major concerns	No concerns	Low

Exenatide:Liraglutide	Some concerns	Low risk	No concerns	Some concerns	No concerns	No concerns	High
Exenatide:Placebo	Some concerns	Low risk	No concerns	No concerns	No concerns	No concerns	Moderate
Exenatide:Semaglutide	Some concerns	Low risk	No concerns	No concerns	Major concerns	No concerns	Low
Liraglutide:Placebo	No concerns	Low risk	No concerns	No concerns	No concerns	No concerns	High
Liraglutide:Semaglutide	No concerns	Low risk	No concerns	No concerns	Major concerns	No concerns	Low
Orforglipron:Placebo	No concerns	Low risk	No concerns	No concerns	No concerns	No concerns	High
PEG-Loxenatide:Placebo	Some concerns	Low risk	No concerns	No concerns	No concerns	No concerns	Moderate
Placebo:Semaglutide	No concerns	Low risk	No concerns	No concerns	No concerns	No concerns	High
Dulaglutide:Exenatide	Some concerns	Low risk	No concerns	Major concerns	No concerns	No concerns	Low
Dulaglutide:PEG-Loxenatide	Some concerns	Low risk	No concerns	Major concerns	No concerns	No concerns	Low
Exenatide:Orforglipron	Some concerns	Low risk	No concerns	No concerns	No concerns	No concerns	Moderate
Exenatide:PEG-Loxenatide	Some concerns	Low risk	No concerns	Major concerns	No concerns	No concerns	Low
Liraglutide:Orforglipron	No concerns	Low risk	No concerns	No concerns	Major concerns	No concerns	Low
Liraglutide:PEG-Loxenatide	Some concerns	Low risk	No concerns	Major concerns	No concerns	No concerns	Low
Orforglipron:PEG-Loxenatide	Some concerns	Low risk	No concerns	Major concerns	No concerns	No concerns	Low

Orforglipron:Semaglutide	No concerns	Low risk	No concerns	Major concerns	No concerns	No concerns	Low
PEG-Loxenatide:Semaglutide	Some concerns	Low risk	No concerns	Major concerns	No concerns	No concerns	Low
Visepegenatid : Placebo	No concerns	Low risk	No concerns	No concerns	No concerns	No concerns	High

Suplementary 10: Sensitivity analyses

Sensitivity analyses were performed by excluding all trials that compare GLP-1 receptor agonists with other classes of glucose-lowering drugs.

Table S10.1: Sensitivity analyses of primary outcomes

GLP-1 RA	HbA_{1c}		FPG		Weight	
	Main estimate	Sensitivity analyses	Main estimate	Sensitivity analyses	Main estimate	Sensitivity analyses
Dulaglutide	-1.0 (-1.1, -0.88)	-0.95 (-1.1, -0.79)	-1.4 (-1.6, -1.1)	-1.4 (-1.6, -1.1)	-1.1 (-1.7, -0.53)	-0.98 (-1.7, -0.27)
Semaglutide	-1.3 (-1.4, -1.2)	-1.2 (-1.4, -1.0)	-2.1 (-2.4, -2.8)	-1.8 (-2.1, -1.5)	-3.1 (-3.6, -2.5)	-3.3 (-4.1, -2.6)
Exenatide	-0.94 (-1.1, -0.77)	-0.69 (-0.98, -0.4)	-1.2 (-1.6, -0.87)	-0.68 (-1.1, -0.21)	-1.9 (-2.7, -1.2)	-1.5 (-2.8, -0.14)
PEG-Loxenatide	-0.91 (-1.4, -0.47)	-0.97 (-1.5, -0.45)	-1.4 (-2.4, -0.4)	-1.5 (-2.5, -0.41)	0.34 (-2, 2.7)	-0.28 (-2.7, 2.1)
Liraglutide	-1.0 (-1.1, -0.89)	-1.0 (-1.2, -0.88)	-1.6 (-1.8, -1.3)	-1.4 (-1.7, -1.1)	-1.7 (-2.2, -1.1)	-1.5 (-2.2, -0.70)
Lixisenatide	-0.49 (-0.82, -0.15)	-0.52 (-0.93 ,-0.1)	-1.1 (-1.8, -0.47)	-0.48 (-1.2, -0.20)	-1.5 (-3.1, 0.19)	-1.1 (-3, 0.78)
Albiglutide	-0.83 (-1.1 -0.52)	-1.1 (-2, -0.14)	-1.4 (-2, -0.75)	-1.6 (-3.1, -0.12)	-1.8 (-4.1, 0.56)	-0.2 (-4.4,4)
Efpeglenatide	-0.77 (-1.0, -0.53)	-0.77 (-1.1, -0.52)	-1.4 (-1.9, -0.96)	-1.4 (-1.80, -0.95)	-1.3 (-2.4, -0.19)	-1.3 (-2.4, -0.1)
Taspoglutide	-0.78 (-1.1, -0.51)	-0.78 (-1.3, -0.24)	-0.95 (-1.5, -0.42)	-1.3 (-2.1 -0.39)	-2.3 (-3.7, -0.78)	-1.1 (-3.6 1.3)
Tirzepatide	-1.9 (-2.1, -1.7)	-1.8 (-2.0, -1.5)	-2.7 (-3.0, -2.3)	-2.7 (-3.1, -2.2)	-7.7 (-8.6, -6.8)	-7.2 (-8.4, -6.1)
Survodutide	-1.1 (-1.4, -0.8)	-1.1 (-1.4, -0.72)	NA	NA	-4.3 (-5.7, -2.8)	-4.4 (-5.9, -2.9)
Visepegenatide	-0.65 (-1.3, -0.031)	-0.65 (-1.3, -0.0047)	-0.68 (-1.9, -0.51)	-0.68 (-1.7, -0.38)	NA	NA
Retatrutide	-1.2 (-1.6, -0.82)	-1.2 (-1.6, -0.77)	-1.7 (-2.5, -0.98)	-1.7 (-2.4 -1.1)	-9.7 (-11, -7.9)	-9.6 (-11, -7.8)
Orforglipron	-1.6 (-1.9, -1.2)	-1.5 (-1.9, -1.2)	-2.2 (-2.9 -1.6)	-2.2 (-2.8, -1.6)	-5.4 (-7, -3.8)	-5.3 (-7, -3.7)
Mazdutide	-1.1 (-1.5, -0.81)	-1.1 (-1.4, -0.76)	-1.5 (-2.1, -0.92)	-1.5 (-2.1, -0.98)	-3.7 (-5.1, -2.2)	-3.6 (-5.1, -2.1)

ITCA 650	-0.8 (-1.7, -0.076)	-0.8 (-1.7, 0.12)	NA	NA	-1.0 (-5.0, -3.0)	-1.0 (-5.2, 3.2)
Cotadutide	-0.82 (-1.3, -0.38)	-1.1 (-1.7, -0.60)	NA	NA	-2.8 (-4.8, -0.79)	-3.0 (-5.4, -0.62)

Appendix 11: The meta-regression of the factors that may lead to differences to the main outcome indicators

Factors	HbA_{1c}			FPG			Body weight		
	Coefficient	95% CrI	P value	Coefficient	95% CrI	P value	Coefficient	95% CrI	P value
Diabetes duration	0.07	-0.17,0.10	0.11	0.04	-0.01, 0.06	0.22	0.04	-0.07, 0.14	0.31
Age	0.14	-0.03,0.18	0.06	0.06	-0.01, 0.14	0.16	-0.01	-0.13, 0.22	0.72
Gender	0.06	-0.03,0.11	0.12	0.05	-0.02, 0.12	0.18	-0.02	-0.10, 0.06	0.65
BMI	0.12	-0.02, 0.15	0.09	0.08	-0.04, 0.2	0.19	-0.05	-0.13, 0.03	0.21
Baseline HbA1c	-0.25	-0.40, 0.10	0.08	-0.30	-0.45, 0	0.09	-0.10	-0.20, 0.00	0.05
Whether background hypoglycemic therapy was combined	-0.13	-0.89, -0.06	0.03	-0.21	-0.58, -0.14	0.04	0.05	0.01,0.09	0.03

Suplementary 12: Effects of different doses of GLP-1RAs on various indicators of T2DM.

12.1 Forest plot of different doses of GLP-1RAs in reducing glycated hemoglobin

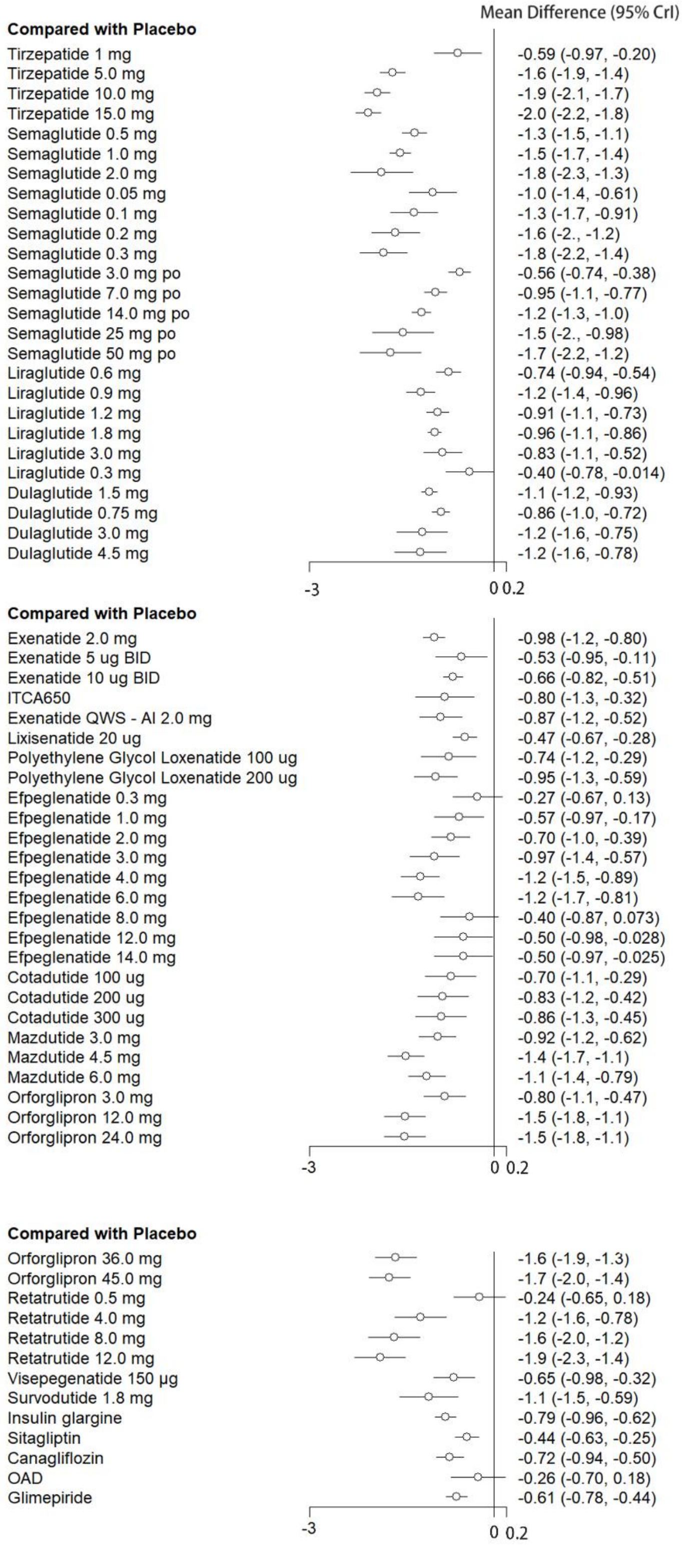

12.2 Forest plot of different doses of GLP-1RAs in reducing fasting blood glucose

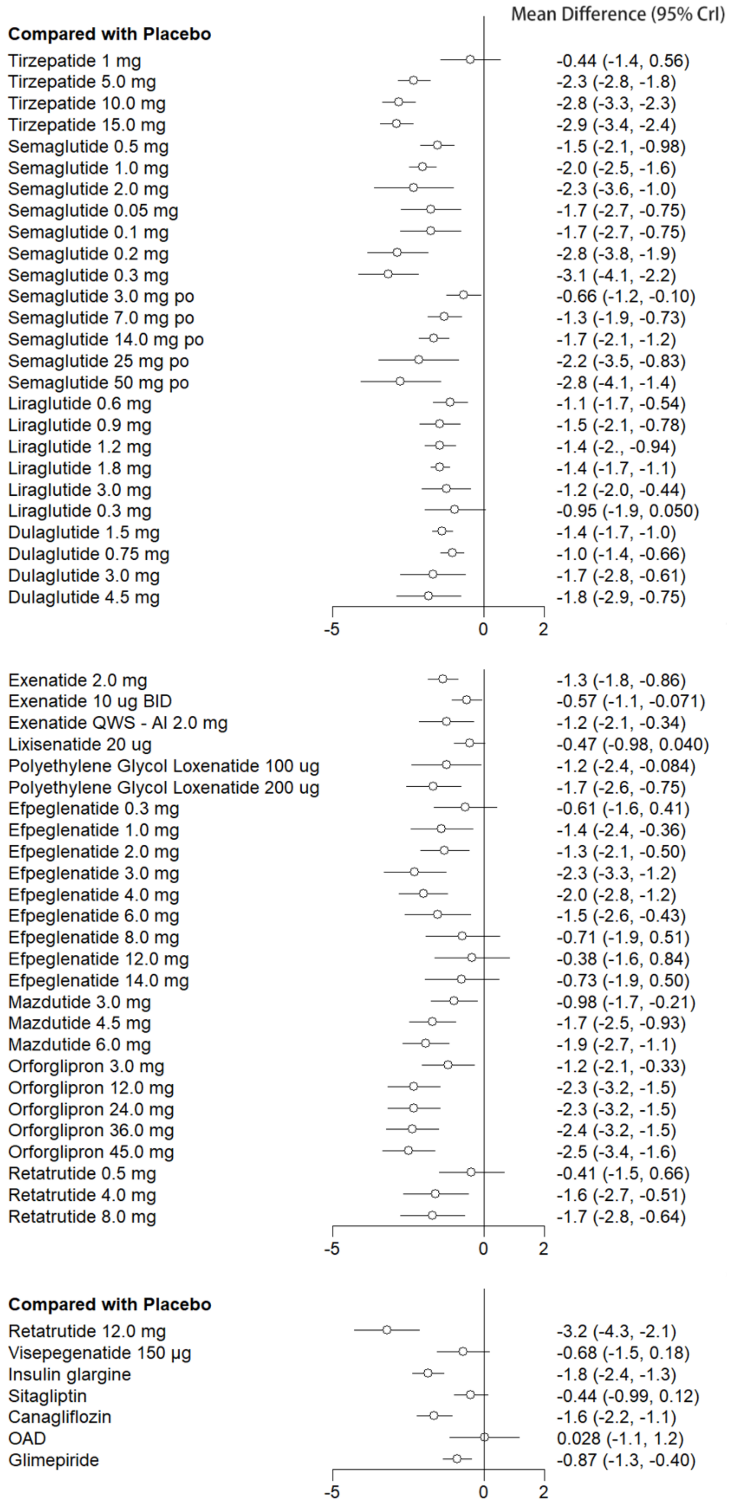

12.3 Forest plot of different doses of GLP-1RAs in reducing body weight

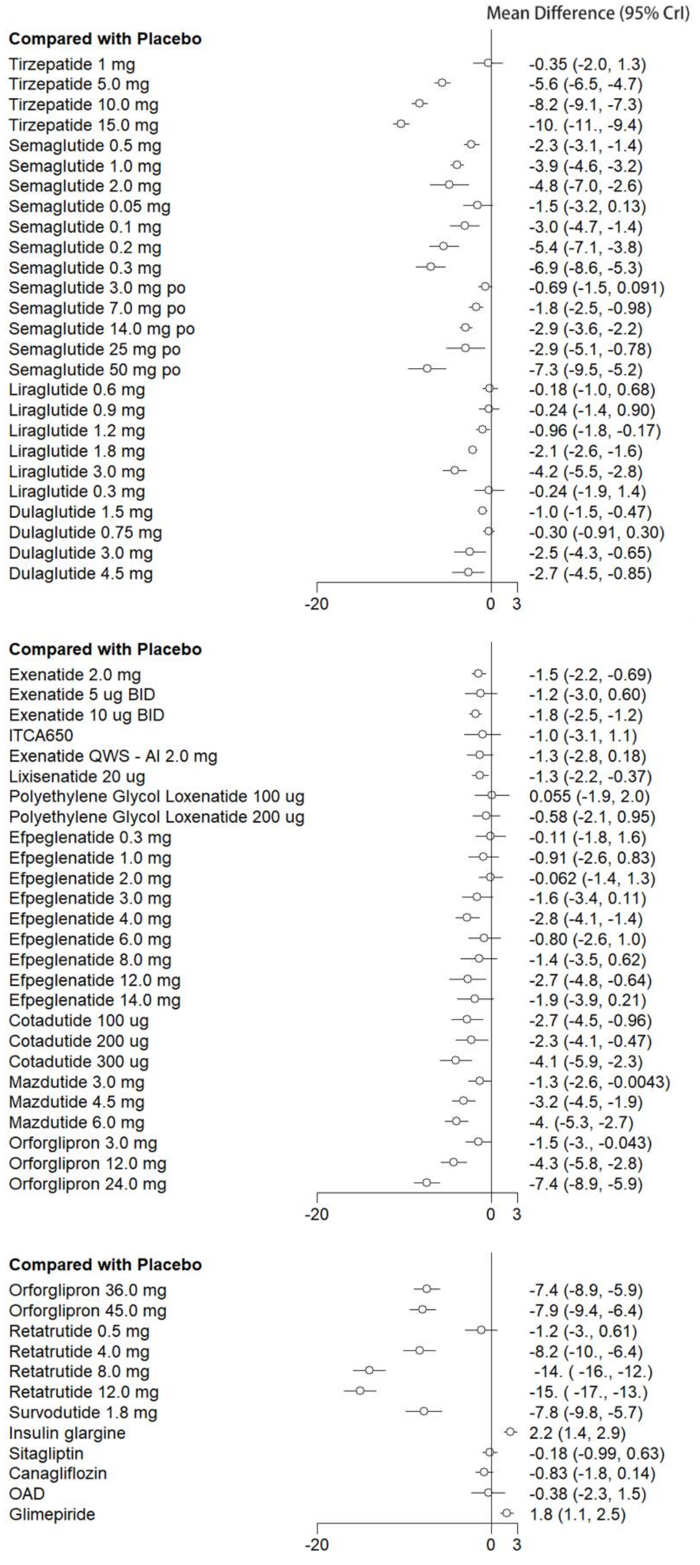

12.4 Forest plot of different doses of GLP-1RAs in terms of inducing nausea (incidence rate)

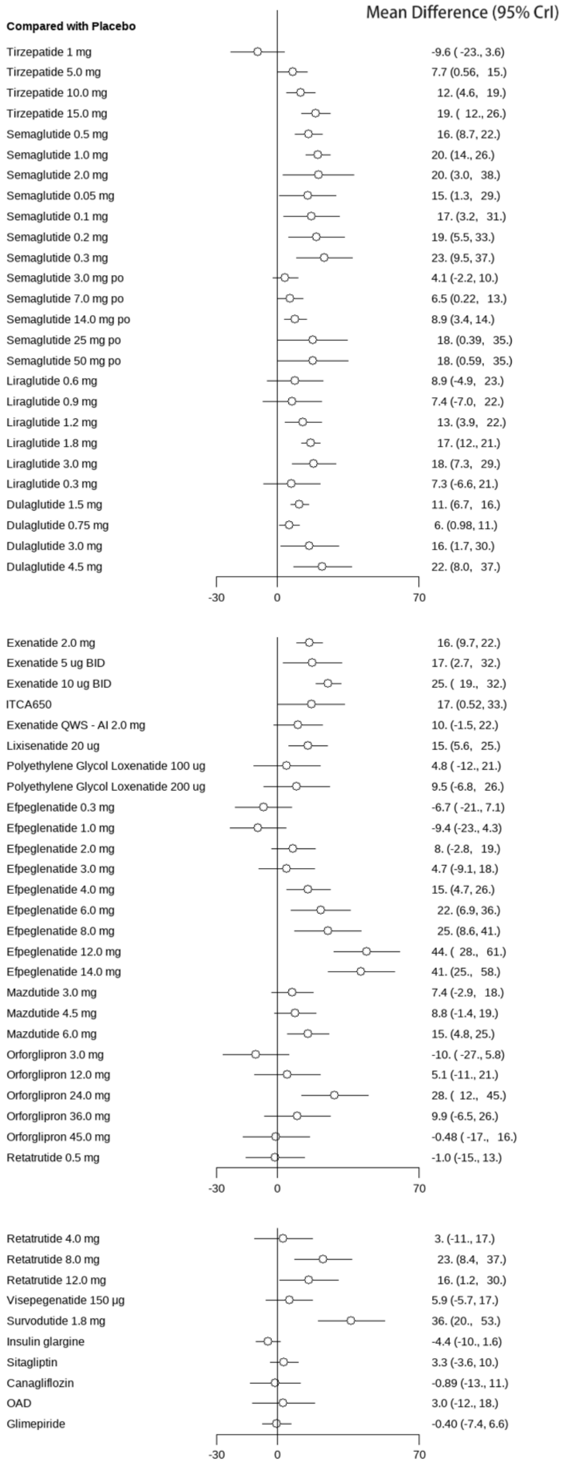

12.5 Forest plot of different doses of GLP-1RAs in inducing vomiting (incidence rate)

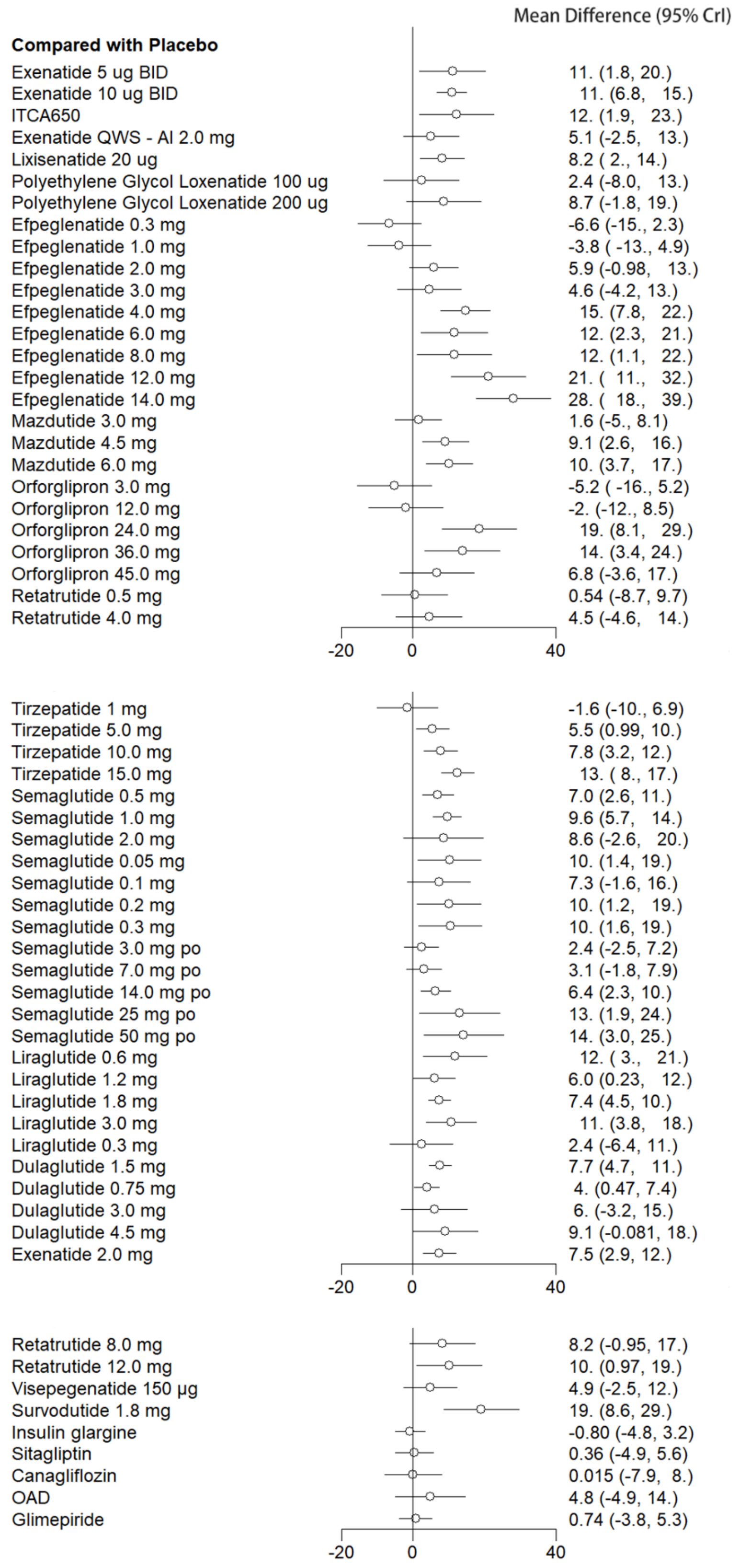

12.6 Forest plot of different doses of GLP-1RAs in terms of inducing diarrhea (incidence rate)

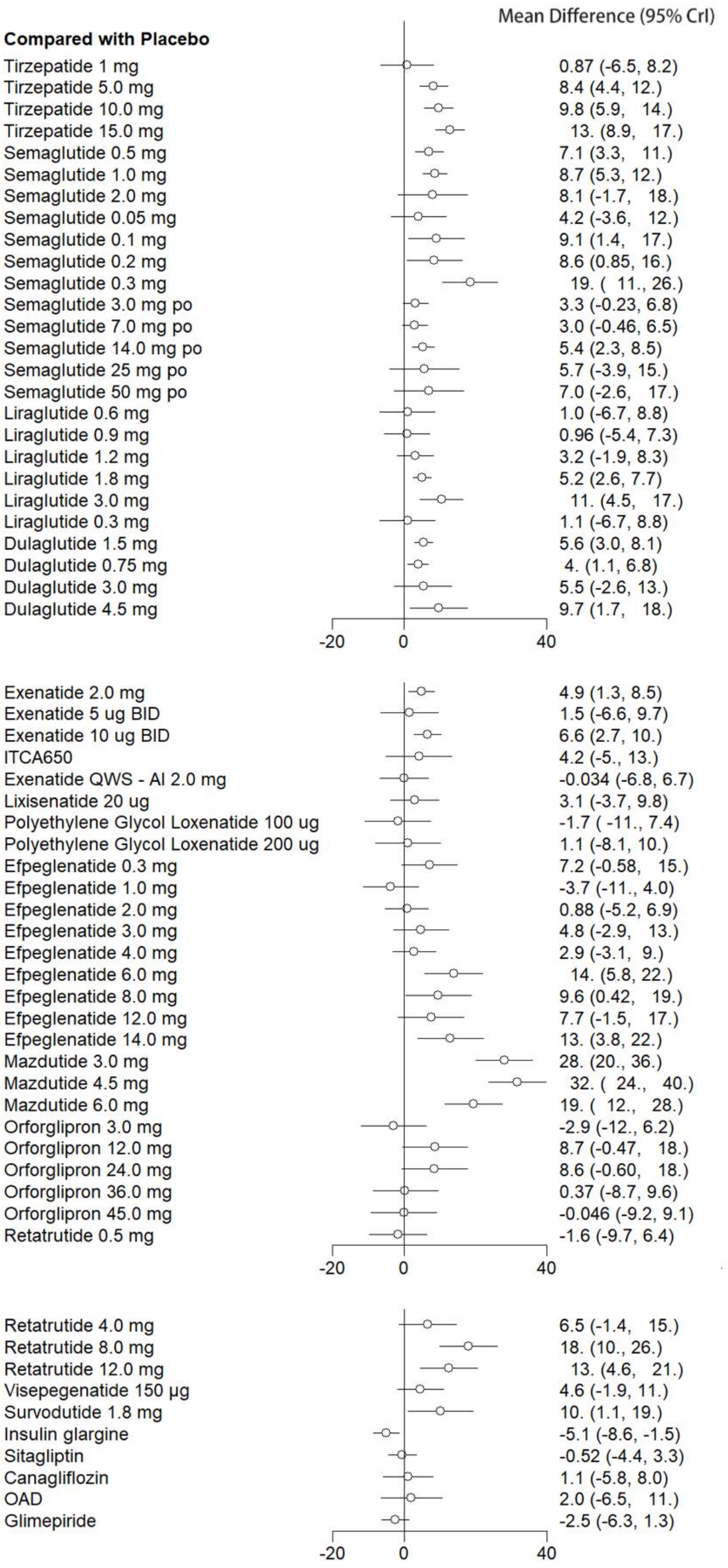

12.7 Forest plot of different doses of GLP-1RAs in inducing constipation (incidence rate)

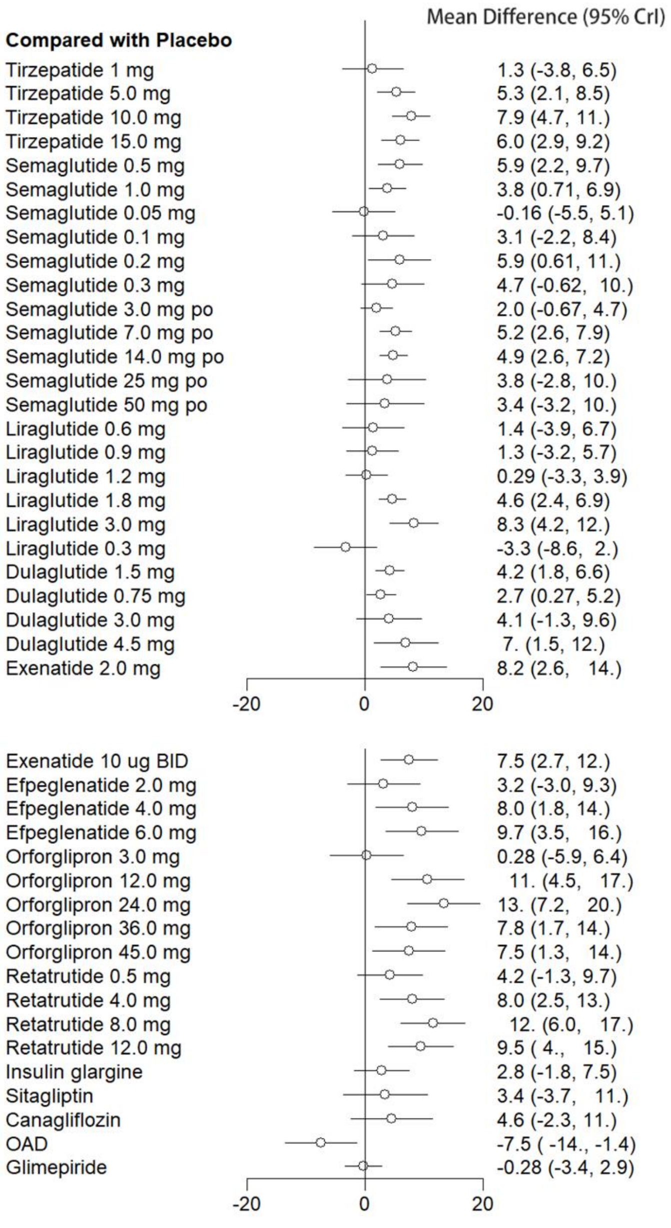

Suplementary 13: Effects of treatment durations of different GLP-1 receptor agonists on various indicators in T2DM.

13.1 Forest plot of GLP-1RAs with different treatment courses in reducing HbA_{1c}
In the following forest plot, A, B, C, D, E, and F represent: A: Dulaglutide; B: Semaglutide; C: Exenatide; D: Liraglutide; E: Tirzepatide; F: Efpeglenatide

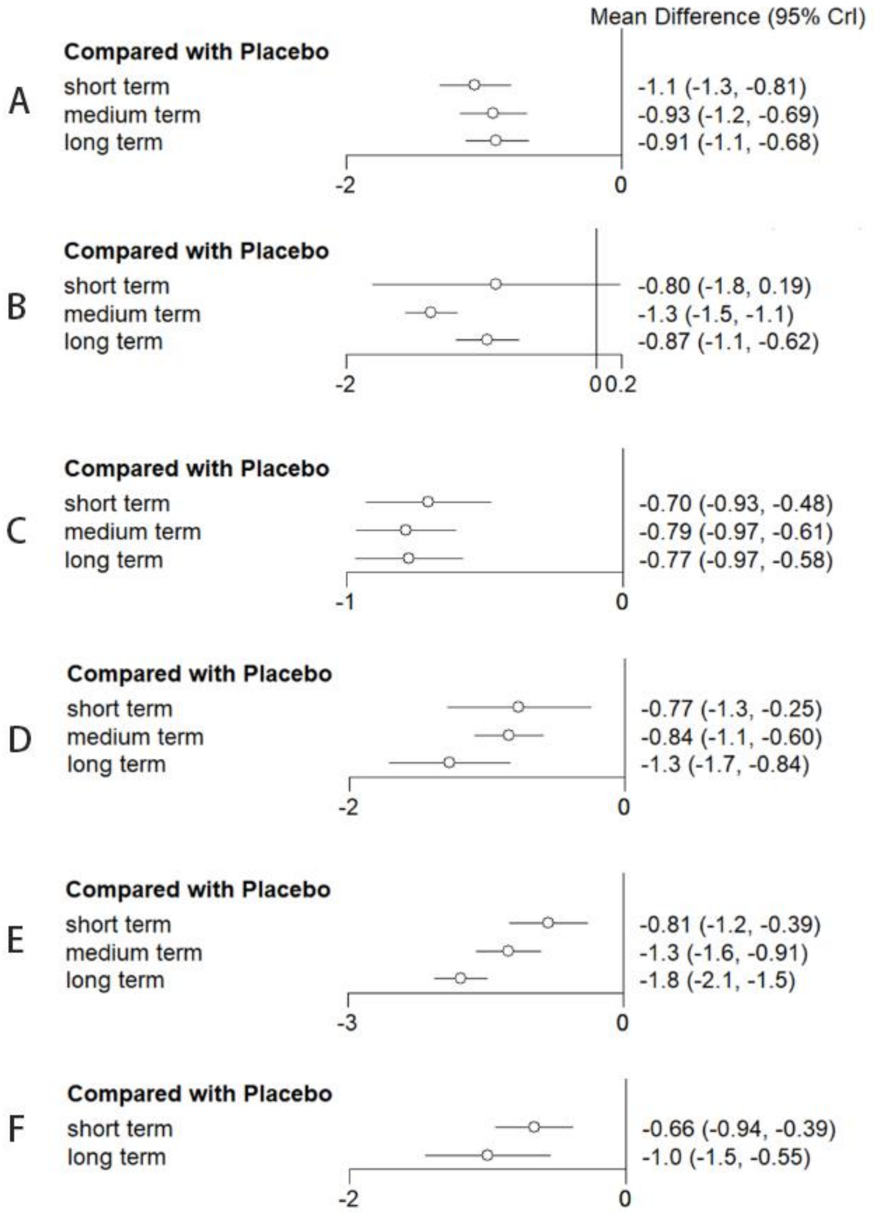

13.2 Forest plot of GLP-1RAs with different treatment courses in reducing FPG
In the following forest plot, A, B, C, D, E, and F represent: A: Dulaglutide; B: Semaglutide; C: Exenatide; D: Liraglutide; E: Tirzepatide; F: Efpeglenatide

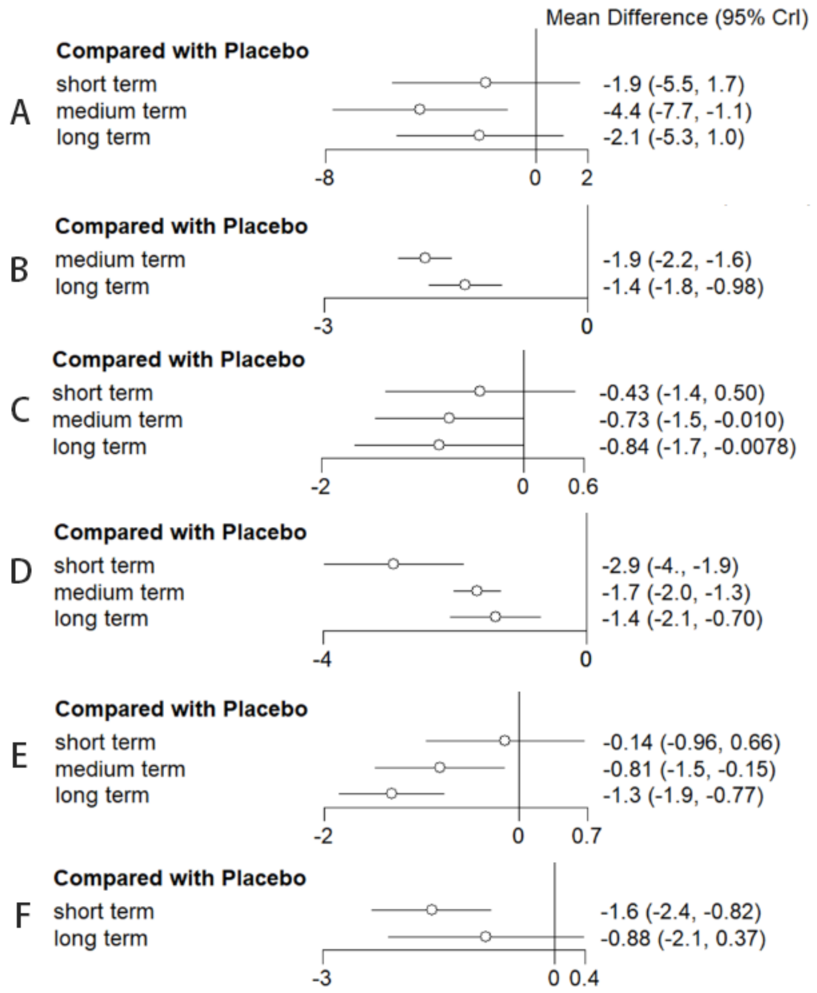

13.3 b

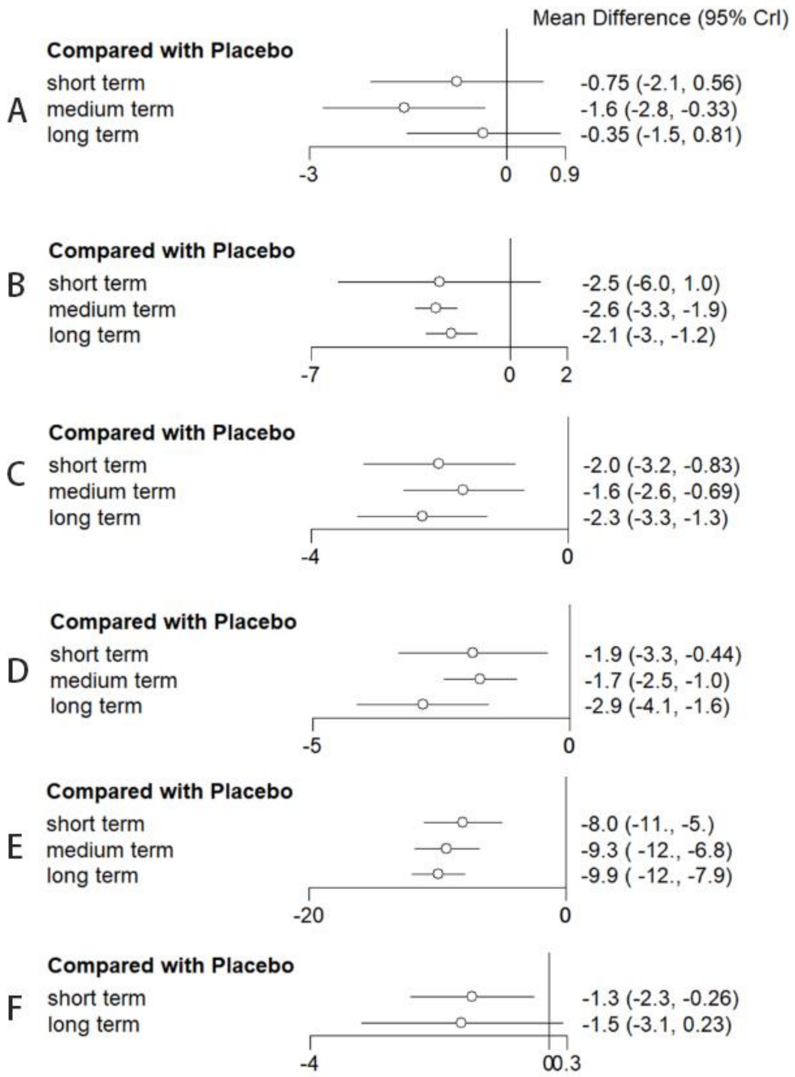

13.4 Forest plot of GLP-1RAs with different treatment courses in inducing nausea In the following forest plot, A, B, C, D, E, and F represent: A: Dulaglutide; B:

Semaglutide; C: Exenatide; D: Liraglutide; E: Tirzepatide; F: Efpeglenatide

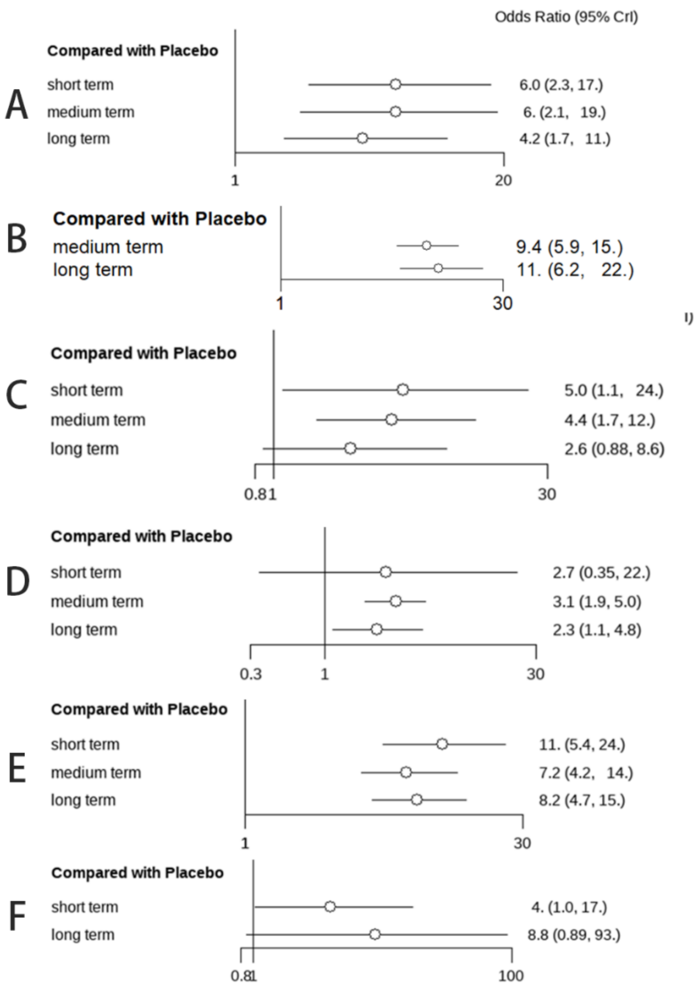

13.5 Forest plot of GLP-1RAs with different courses of treatment in inducing vomiting In the following forest plot, A, B, C, D, E, and F represent: A: Dulaglutide;

B: Semaglutide; C: Exenatide; D: Liraglutide; E: Tirzepatide; F: Efpeglenatide

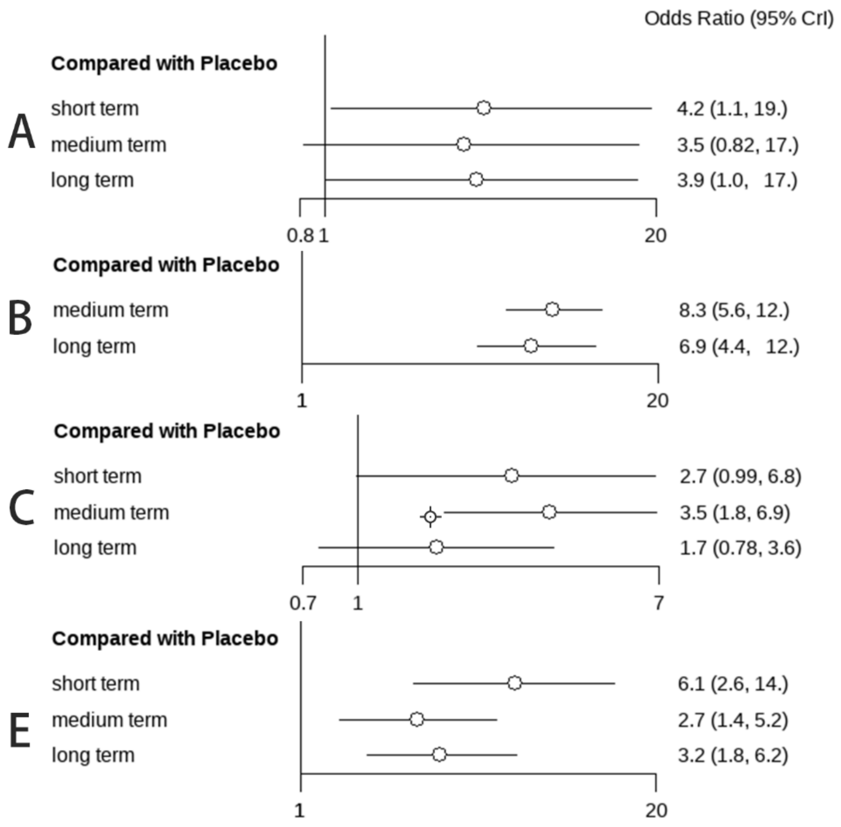

13.6 Forest plot of GLP-1RAs with different treatment courses in inducing diarrhea

In the following forest plot, A, B, C, D, E, and F represent: A: Dulaglutide; B: Semaglutide; C: Exenatide; D: Liraglutide; E: Tirzepatide; F: Efpeglenatide

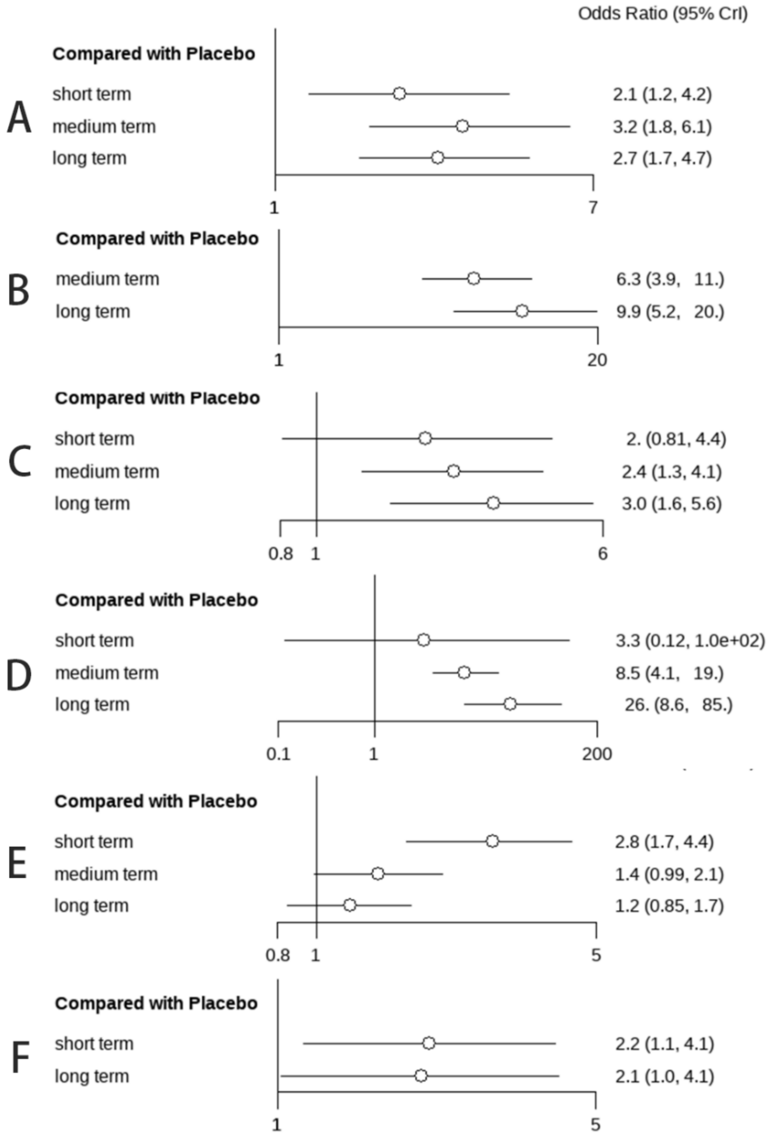

13.7 Forest plot of GLP-1RAs with different treatment courses in inducing constipation

In the following forest plot, A, B, C, D, E, and F represent: A: Dulaglutide; B: Semaglutide; C: Exenatide; D: Liraglutide; E: Tirzepatide; F: Efpeglenatide

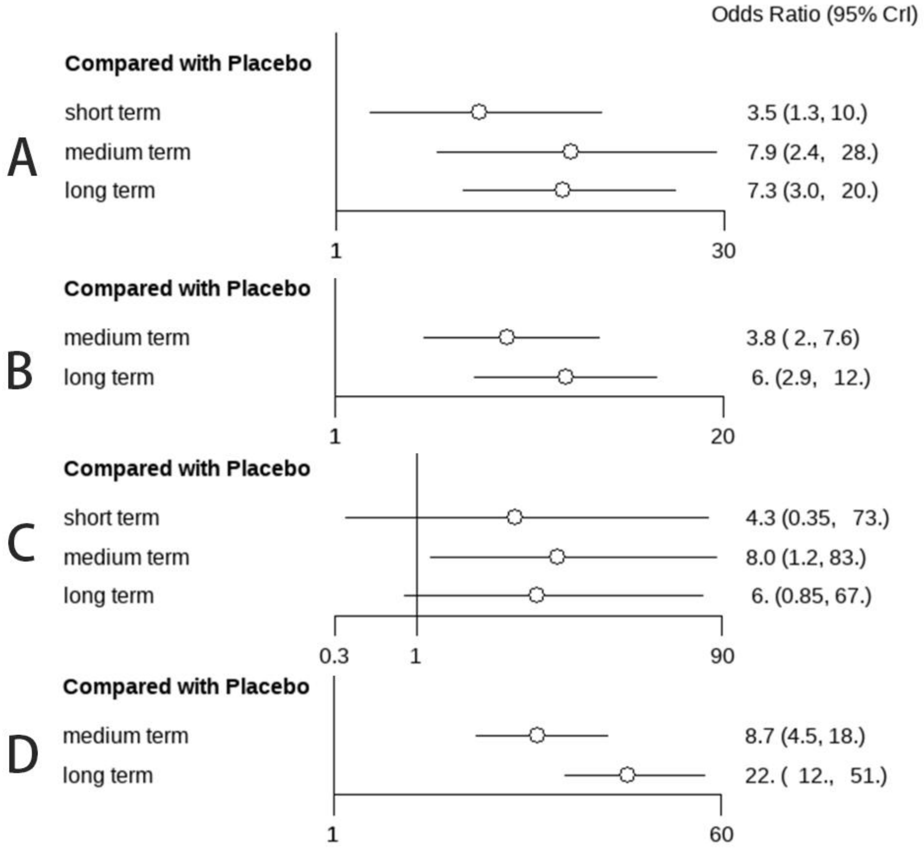

Suplementary14: Effects of different combination therapy regimens of GLP-1 receptor agonists on various indicators of T2DM.

14.1 Forest plot of GLP-1RAs in reducing HbA_{1c} under different combination therapy regimens

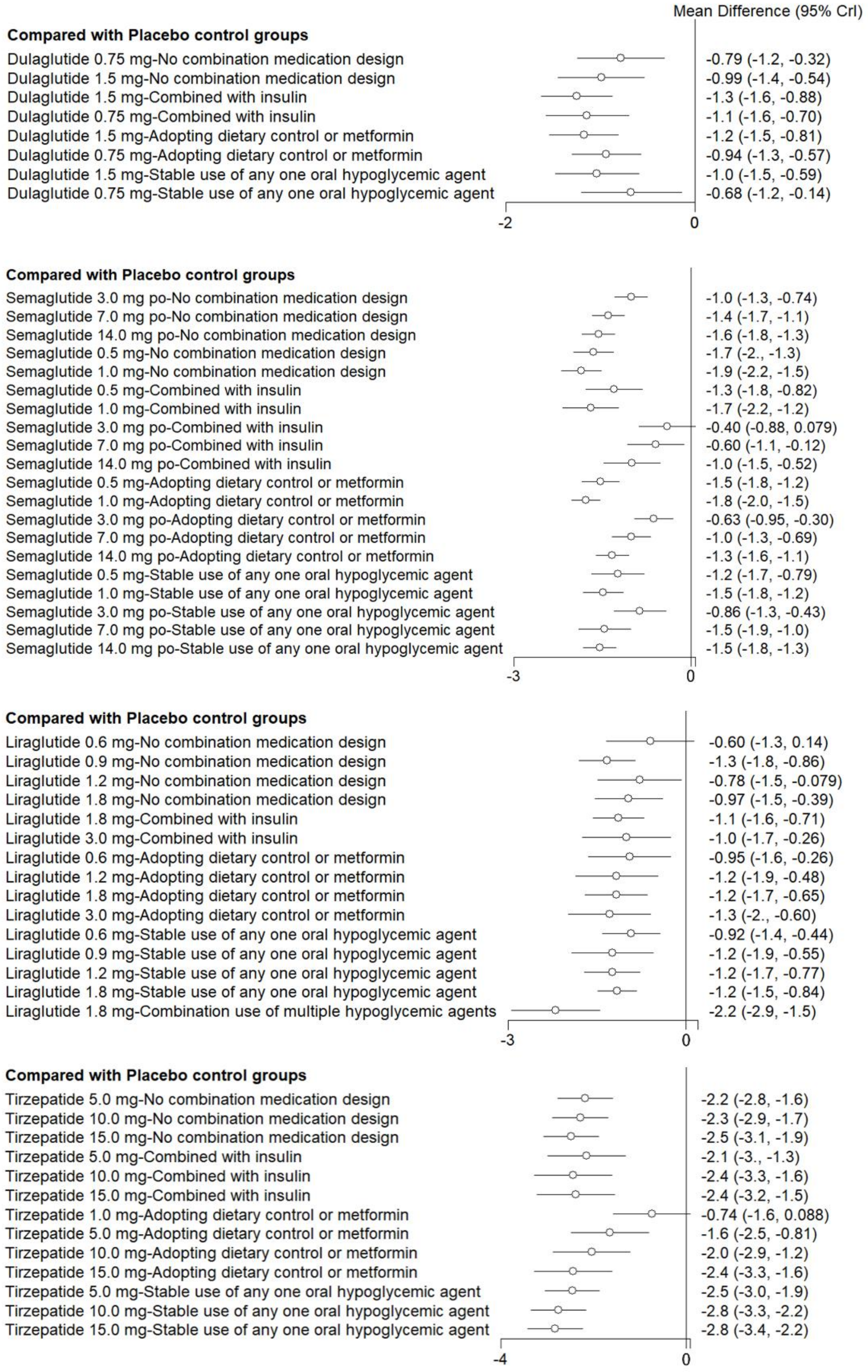

14.2 Forest plot of GLP-1RAs in reducing FPG under different combined medication regimens

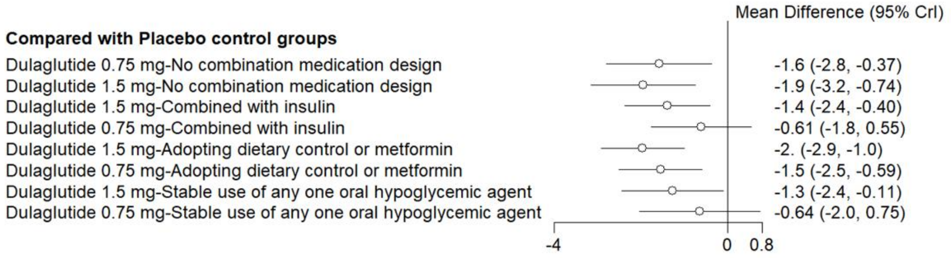

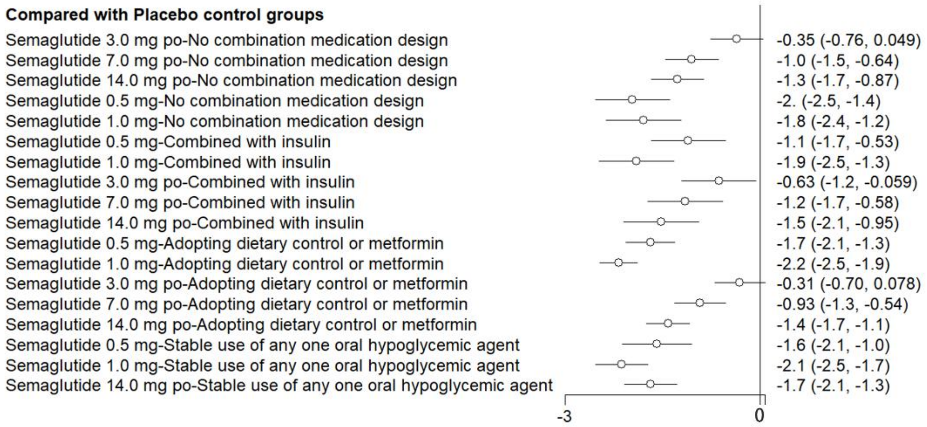

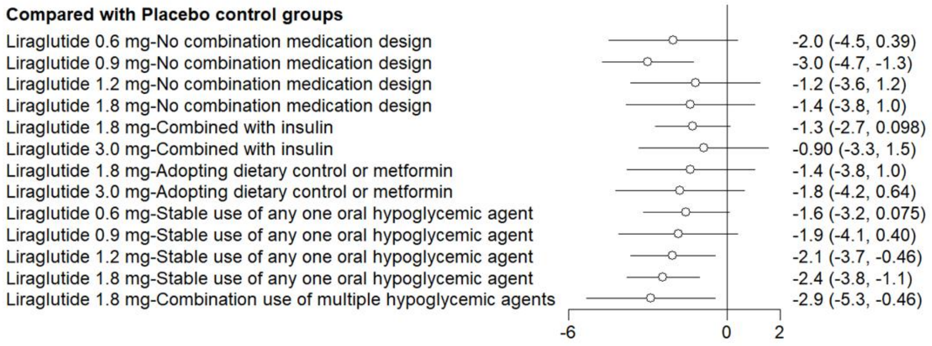

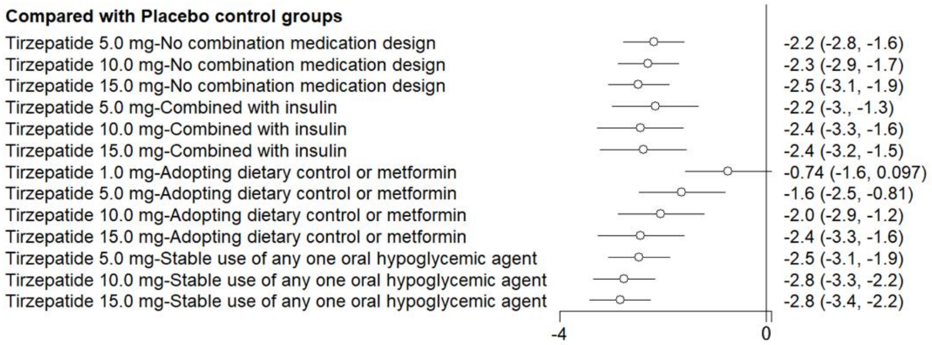

14.3 Forest plot of GLP-1RAs in reducing weight under different combination medication regimens

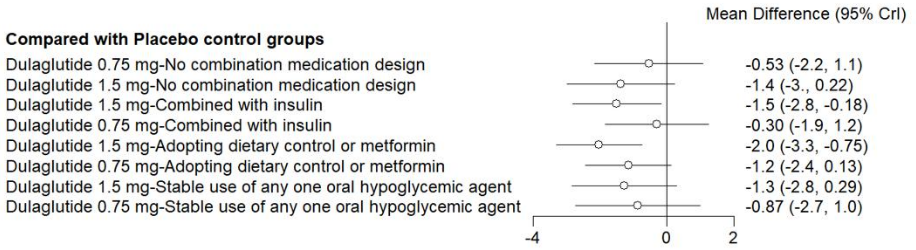

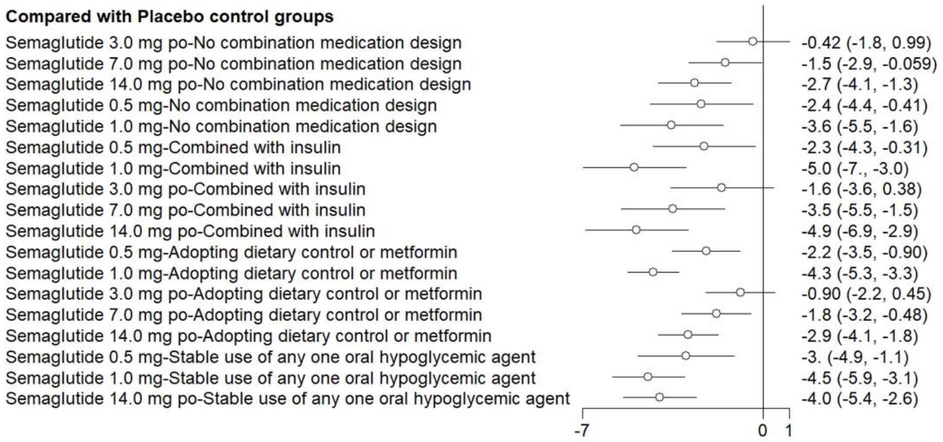

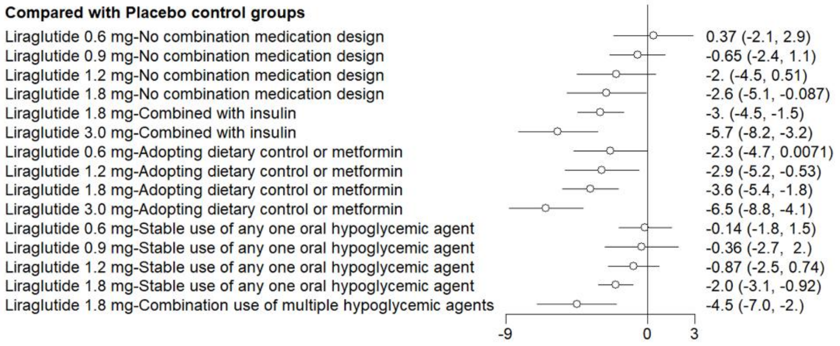

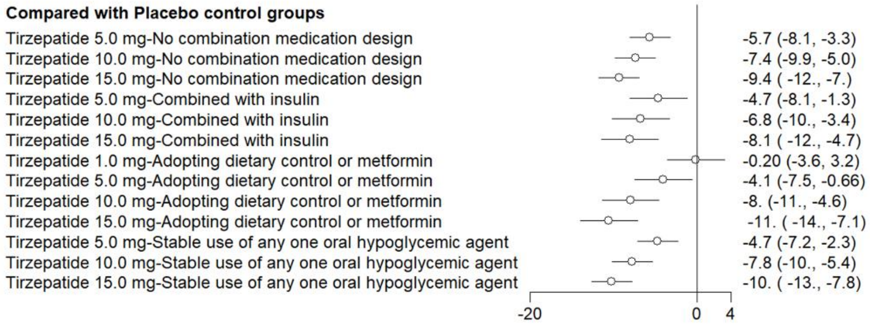

Supplement: Supplementary file 1 [file DataSheet1.pdf]
